# Supplementary material for: Remission of type 2 diabetes and improved diastolic function by combining structured exercise with meal replacement and food reintroduction among young adults: the RESET for REMISSION randomised controlled trial protocol
Source: BMJ Open. 2022 Sep 21;12(9):e063888. doi: 10.1136/bmjopen-2022-063888 (PMC9494595; doi:10.1136/bmjopen-2022-063888)
Supplement: Supplementary data [file bmjopen-2022-063888supp001.pdf]

**Appendix 1**. Interview guide for in-depth interviews with intervention arm participants at completion of final evaluations

This topic guide is a flexible tool and may be revised as new areas of interest arise during the process of data collection. The wording of the question is for guidance only and can be varied to suit the natural style of the interviewer and the level of understanding of the participant. This topic guide will be adapted to use for those who are involved in this study.

The interviewer will be a trial team member who has not interacted with the participant during the course of the trial. The interview may be conducted in person, by video teleconference, or by telephone, as preferred by the participant.

Introductory remarks

The interviewer will iterate that the purpose of the interview is to:

- Explore the participant’s knowledge, views and experience of the RESET trial interventions

Participants will be assured that:

- They are not being ‘tested’ – there are no right or wrong answers.
- They are free to say as much or as little as they want.
- Their specific comments will be anonymously quoted in manuscripts and reports. Individual comments will not be shared with anyone external to the research team.

Recording will be started after introductory remarks.

Questions to discuss

1. What made you want to participate in this study?

2. When you started, did you feel you were you prepared to invest time and effort to make the intervention work?

3. When you were assigned to the 'active intervention' group, what was your understanding of how the intervention could benefit you?

4. What are your thoughts about this programme overall? Probe: What did you think about the format? The length of the sessions? What did you find easy to do? What was difficult?

5. Were you able to attend the sessions? Probe: if participants respond no, probe reasons for not being able to attend.

6. Was the venue convenient for the exercise training? (i.e. easy to attend) If not, what would have helped to make the venue convenient to you?

7. Dietary component:

   7a) What was your experience with the dietary support sessions? Prompt: meal replacement products, dietary support sessions, limited amounts of food

   7b) How did you find the reintroduction to food? Prompt: questions around pace, was it manageable, did they require more support etc.

   7c) Can you tell us if you felt the sessions helped in any way to manage your diet? Probe in what way do you feel this has helped, or has not helped?

8. Exercise component:

8a) You took part in exercise training (combined aerobic and resistance activities) – Can you tell me a little bit about this? Prompt: difficulty, convenience, motivation, challenges in combination with diet

8b) How well were you able to exercise independently once the close supervision of the first 12 weeks ended? Prompt: Challenges, need for return to supervision

8c) Can you tell us if you felt the sessions helped in any way to manage your exercise training? Probe: in what way do you feel this has helped, or has not helped?

9. Medication adjustments, glucose and blood pressure:

9a) How did you feel about stopping some medications at the beginning of the program? Probe: What concerns, if any, did you have?

9b) How did you feel about your glucose levels and blood pressure levels during the program? Probe: Did you feel that you were well-monitored during this period?

10. Did you make any changes in your eating? Probe: If yes, when you were making your diet changes, did you feel you were making a difference to your health?

11. Did you make any changes in your exercise? Probe: If yes, when you were making your exercise changes, did you feel you were making a difference to your health?

12. What skills do you feel you have taken from taking part in the intervention?

13. Diet component

13a) Were there any tasks or time constraints that would influence your ability in making changes to your diet? Elaborate

13b) What factors would you say influenced whether you were able to maintain your diet plan?

14. Exercise component

14a) Were there any tasks or time constraints that would influence your ability in making changes to you exercise? Elaborate

14b) What factors would you say influenced whether you were able to maintain your exercise plan?

15. Did you ever discuss the programme with your family members or friends? Probe: What did they think of your participation? Were they supportive? Concerned? Were they sceptical?

16. Would your family members or friends influence what you did as part of the programme? If so, to what extent? Prompt: i.e. was their influence positive? Was it negative?

17. Do you feel the intervention affected your relationships in any way? Prompt: i.e. by not eating regular food based meals with the family

18. If you made changes to your eating or physical activity, how important do you think it is for you to maintain these changes to your health? (elaborate why this is or isn't important to the participants)

19. How optimistic are you about maintaining these changes to your health? Prompt: if not optimistic, explore the reasons why

20. If there was additional support available in the intervention, what would this be?

21. What would you do differently if you were asked to take part again?

22. Do you have any suggestions for improving the programme?
