# Supplementary material for: Remission of type 2 diabetes and improved diastolic function by combining structured exercise with meal replacement and food reintroduction among young adults: the RESET for REMISSION randomised controlled trial protocol
Source: BMJ Open. 2022 Sep 21;12(9):e063888. doi: 10.1136/bmjopen-2022-063888 (PMC9494595; doi:10.1136/bmjopen-2022-063888)
Supplement: Supplementary data [file bmjopen-2022-063888supp002.pdf]

Centre universitaire de santé McGill McGill University Health Centre

Centre universitaire de santé McGill McGill University Health Centre

X HME MCH X HGM MGH X HRV RVH
X HNM MNH X ITM MCI X CL LC

FMU-9997

INFORMATION AND CONSENT FORM

**Research Study Title:**	REMISSION OF DIABETES AND IMPROVED DIASTOLIC FUNCTION BY COMBINING STRUCTURED EXERCISE WITH MEAL REPLACEMENT AND FOOD REINTRODUCTION IN ENGLAND AND CANADA: THE **RESET FOR REMISSION** TRIAL
**Protocol number:**	2021-7148
**Researcher responsible for the research study:**	Dr. Kaberi Dasgupta, Professor of Medicine and Physician, MUHC; Scientist, Research Institute of the MUHC
**Co-Investigators:**	Dr. Stéphanie Chevalier, Associate Professor of Medicine and Scientist, Research Institute of the MUHC<br>Dr. Deborah Da Costa, Associate Professor of Medicine and Scientist, Research Institute of the MUHC<br>Dr. Elham Rahme, Associate Professor of Medicine and Scientist, Research Institute of the MUHC<br>Dr. Matthias G. Friedrich, Professor of Medicine and Radiology and Physician, MUHC<br>Dr. Isabel Fortier, Assistant Professor of Medicine, MUHC; Scientist, Research Institute of the MUHC<br>Dr. Mark Sherman Associate Professor of Medicine and Physician, MUHC
**Funding source:**	The Canadian Institutes of Health Research (CIHR)
**Sponsor:**	The Research Institute of the MUHC

INTRODUCTION

We are inviting you to take part in this research study because you were diagnosed with Type 2 diabetes less than 6 years ago.

However, before you accept to take part in this study and sign this information and consent form, please take the time to read, understand and carefully examine the following information. You may also want to discuss this study with your doctor, a family member or a close friend.

We invite you to speak to the researcher responsible for this study ("the researcher") or to other

Dasgupta K, *et al. BMJ Open* 2022; 12:e063888. doi: 10.1136/bmjopen-2022-063888

members of the research team and ask them any questions you may have about this study. Please also ask a member of the research team about any parts of this consent form you do not understand.

BACKGROUND
More and more people are getting type 2 diabetes. They are also getting it at younger ages (aged between 18-40 years) than in the past. This is worrying because heart, kidney and physical fitness problems related to diabetes can therefore also happen at an earlier age than in the past.

Past studies show that diets with a low number of calories can make blood sugar levels go back to normal without diabetes medications in people who have had type 2 diabetes for just a few years. This is called 'reversing diabetes.' A low calorie diet means eating a lot less than normal for some weeks often with special shakes and meal bars, with the guidance of a dietitian. Once people have lost enough weight, they can start to eat more regular food again while trying to keep off the weight they may have lost.

Exercise, such as walking and simple muscle building activities, can also make your blood sugar levels better and help your heart and muscles work more efficiently. Putting a low calorie diet *together* with a supervised exercise program has not been studied before to look at how well it works in reversing diabetes and improving how the heart and muscles work.

PURPOSE OF THE RESEARCH STUDY
The main goal of this study is to see whether combining exercise and a low calorie diet can reverse diabetes and make the heart and muscles work better.

This study is a randomized trial. This means that if you agree to participate in this study, you will be randomly assigned to one of two groups based on chance, like rolling a dice or flipping a coin. The two groups in this trial are:

- Group 1: **usual standard care** (that means continuing to be treated as you are right now)
- Group 2: a combination of an **exercise program and a low calorie diet**.

Neither you nor the researchers can choose the group to which you will be assigned. We will not know in which group you will be assigned until the trial begins. Both groups will have a series of tests and questionnaires at the beginning, middle, and at the end of the study, so we can compare the groups and what happens to them.

For this research study, we will recruit 30 to 35 people in Montreal. They will be men and women, between 18 and 40 years of age.

DESCRIPTION OF THE RESEARCH PROCEDURES
This research study will take place at the McGill University Health Centre. During the study, the research staff may communicate with you via e-mail, cell phone texting (standard text message rates apply) or video call.

Dasgupta K, *et al. BMJ Open* 2022; 12:e063888. doi: 10.1136/bmjopen-2022-063888

Your participation in this research project will last 6 to 7 months and will include on-line questionnaires and at least 5 visits for testing (both Group 1 and Group 2). For those who are in the Group 2 (structured exercise with low calorie diet group), there will be more visits for counselling and supervision. Before we ask you to answer questionnaires or schedule visits, the study doctor will first go through your medical records and may talk to your doctor to make sure that you are eligible for the study. If you are eligible, we will ask you to complete the steps below.

1. Duration and number of visits

You will first complete an on-line questionnaire, an on-line 4-day food diary, and 1 week of physical activity monitoring with a device worn on your wrist. A paper-based alternative to the diary and questionnaires is also available, if you prefer. If you complete these procedures, you will be scheduled for the first two visits (visit 1 and visit 2 described in the table below). The study doctor will review the results of the first two visits to confirm that it is okay for you to continue the research. If it's okay, then we will do the randomization (like 'flipping a coin') to decide which of the two study groups you will be in.

2. Description of the trial groups and related procedures

GROUP 1- Standard care

If you are in the standard care group, you will receive your normal diabetes care. You will also be given a leaflet about the benefits of diet and exercise. You will come back for VISITS 3, 4, and 5, as described above. If you complete all visits, **you will be offered the low calorie diet at the end of the trial, in thanks for your participation.**

GROUP 2- Low calorie diet and structured exercise

<u>Glucose and blood pressure medications and study physician supervision</u>

We will stop your diabetes and most blood pressure medications at the beginning of the diet and exercise program. This is because the low calorie diet and the structured exercise will probably lower your blood sugar and blood pressure quite a lot. To be safe, we have to stop the medications.

A study doctor will see you at the beginning of the study and will stay in contact with you throughout the 24 weeks to monitor your blood sugar and blood pressure and decide with you if any medications need to be restarted. You will be asked to monitor your blood glucose more often. For study purposes, we will lend you a home blood pressure monitor and a weighing scale and provide you with glucometer strips if you do not have enough to measure your glucose levels at home.

You will measure your glucose levels, blood pressure, and body weight at home, and report these back to the study physician, dietitian, and exercise specialist, as they instruct. In general, you will be asked to check your glucose level every day before your first meal and 7 times the day before each time you meet with the study doctor (before and 2 hours after each 'meal' and at bedtime), your blood pressure when you wake up in the morning, and your body weight at least once each week. You will be measuring your glucose levels during the study with the usual way of pricking your finger with a lancet, placing a drop of blood on a test strip, and inserting the strip into a glucometer. You will measure your blood pressure with the monitor that we lend you during the study. You will sit comfortably with your back supported, attach the blood pressure cuff (like a band) on your upper arm, and rest for about 5 minutes. You will

Dasgupta K, *et al. BMJ Open* 2022; 12:e063888. doi: 10.1136/bmjopen-2022-063888

then press a button to start the reading. Once the reading is done, you will push the button for a second reading, and this is the reading you will write down. You will write the blood pressure (first number and second number), body weight, and glucose values into an online diary that you and the study physician, dietitian, and exercise physiologist will be able to access. If you prefer, we can give you a paper diary instead. We will give you a fibre-based laxative (Metamucil) to take when needed in case you become constipated while on the low calorie diet described below.

We emphasize that it is very important not to become pregnant during the study. With this in mind, if you are female, the study physician will review your use of reliable contraception throughout the study. If you become pregnant, all study interventions will be stopped.

The low calorie diet

This diet will be a major change in your life for 12 weeks. You have to be aware of what is involved. You will have regular support from a study dietitian. This will be face to face or through telephone calls or virtual meetings.

Part 1 (2 weeks). You will have a “total dietary replacement” where you only consume special shakes and lots of water. The shakes will give you around 900 calories each day. All of the shakes will be free of charge and will be give to you the first time you meet the dietitian, which will be face-to-face at least the first time.

Part 2 (10 weeks). After the first two weeks, you will eat one meal with regular food each day. The rest of what you eat will be meal replacement products like the shakes you had the first two weeks. To give you more variety, on top of shakes, you will also get special soups and bars. On most of the days each week (4 out of 7 days), you will still have about 900 calories each day (all of the meal products and the one meal each day all considered together).

During this ‘part 2’ time, you will also be exercising 3 days each week. On the days that you exercise, you will have some extra meal replacement product so on those days you will be taking in a bit more than 900 calories.

You and the dietitian will figure out together what kinds of meals you will eat to make sure that you are not taking in more calories than the limit set by the study. She will work with you to pick foods that you prefer. She will also help you figure out what combination of shakes, bars, and soups you might like best.

Part 3 (12 weeks). At this point, you will probably have lost weight and we will now try to help you keep it off. Even if you have not lost weight, we will help you transition back to a healthy food-based diet. The dietitian will help you bring back normal foods. She will calculate about how many calories you will need every day to keep your weight the same as it is at 12 weeks. We will support you to figure out portion sizes and food choices. The goal is to help you keep off the weight you have lost. If you gain weight, she will reintroduce or increase some meal replacement products and remove some regular meals from your plan, for a period of time.

Dasgupta K, *et al. BMJ Open* 2022; 12:e063888. doi: 10.1136/bmjopen-2022-063888

The structured exercise program

The exercise program will start after your first two weeks of shakes. We don't want to overwhelm you with too many changes at once, so we will start the exercise program at the same time that you get one food meal back in your diet at week 3.

Just as the dietitian supervises you in the low calorie diet part, an exercise specialist (exercise physiologist) will supervise you in the exercise part. The sessions are free of charge.

Part 1 – During the first 2 weeks when you are having the shakes only diet, you will continue with your usual activities.

Part 2 -- During the next 10 weeks you will exercise, you will have two supervised exercise sessions at the PERFORM Centre, located at 7272 Sherbrooke Street West. You will take part in lots of different exercises designed to increase your fitness and strength. The planned exercise will take into account your level of fitness and ability. You will use a treadmill, free weights, bands, and weight machines. The exercise specialist will help you use these safely and effectively. The specialist will design a third session for you to do each week. This 'unsupervised' session can be done outdoors, at another gym, or at home but you will also have the option to come to PERFORM for this, to access the equipment and facilities. Please note, however, that if there are any government-mandated COVID restrictions in place, the PERFORM gym will not be available for unsupervised exercise. You will wear a Fitbit device that we will lend you so that you and we can monitor your unsupervised exercise and activities of daily living.

Part 3 – During the next 12 weeks, you will exercise on your own. The goal is for you to keep exercising at the level and amount that you have been doing in part 2. The exercise specialist will make a plan with you and will be checking in with you by phone, text, or e-mail. If you are having difficulty or are not exercising regularly, you will be asked to return for some supervised sessions. When you are exercising on your own during these 12 weeks, you will still have the option of exercising at the PERFORM Centre. As we state above, however, if there are any government-mandated COVID restrictions in place, the PERFORM gym will not be available for unsupervised exercise. You will wear a Fitbit device that we will lend you to track your aerobic exercise and physical activity.

Additional glucose sensors

As we talked about above, you will be testing your blood sugar levels at least once a day at home and 7 times on the day before you have a visit with the study doctor. You and the study doctor will know all of these numbers. On top of that, we will measure your glucose levels throughout the day using a special device called a continuous blood glucose monitor (CGM). The study physician won't use the CGM numbers to guide you; she will stick to the numbers you measure yourself at home. We will use the CGM numbers for research analysis ('research math') to understand overall the patterns of blood sugar change in the two study groups. The research team will download and store these numbers for analyses.

We will show you how to place the CGM sensor on the skin of your upper arm. It has a small filament that is inserted under your skin with a small needle (less than 1 cm long). The needle is then removed and only the flexible filament remains under your skin. We will show you how to tape the sensor to

Dasgupta K, *et al. BMJ Open* 2022; 12:e063888. doi: 10.1136/bmjopen-2022-063888

your arm. You can shower with it. Once in place, you do not need to do anything else with it. The CGM sensor measures your blood glucose continuously and stores values every 15 minutes.

We will ask you to wear a CGM sensor at 4 different time points, each time for two weeks. These are the 4 time blocks:
(1) The week before you start the low energy diet and the first week of the low energy diet;
(2) The second week of the low energy diet and the first week supervised exercise is added;
(3) The 11th and 12th weeks after you start the diet, when we should start seeing the strongest effects of what you are doing;
(4) The 23rd and 24th weeks when you are finishing up the study.

Each time you put on one of these sensors, you will mail it back to us after two weeks in a stamped envelope we will give you or you will bring it to the PERFORM centre where we will collect it from you.

3. Tests and procedures
After you agree to join the study, we will ask you to do the following:

Accelerometer to monitor your activity & sleep) for 7 days	We will mail you a small machine to wear on your wrist called a research accelerometer. This measures how much time you spend sleeping, sitting and moving. It can be worn all day and night. We will ask you to wear it for 7 days. You will then mail it back to us in the postage paid envelope that we will give you.
Food diary	We will explain to you how to log onto an online food diary or mail you a paper one to complete. We will ask you to track what you eat over 4 days (three weekdays and one weekend day). If you complete a paper diary, you will mail it back with the accelerometer. This will help you understand what your food habits are.
Questionnaire – done at home	We will ask you to fill out an online questionnaire (or paper version, if you prefer). You can reach our study staff by phone or email to answer any questions you have. You also have the option of answering the questions by phone and the staff can fill in the answers for you.<br>Examples of what the questions will be about are feeling anxious or depressed, how well you function in your day to day life, history of illness in your family, your cultural or ethnic background, any trouble breathing, how much schooling you completed, the kind of work you do if you are employed, and whether or not you have a partner. We will also ask female participants about previous pregnancies. We estimate that these questions will take about 20 minutes to complete.

If you do not complete the above procedures, you will not be invited to continue in the study. Once you complete the above procedures at home, you will be scheduled for some face-to-face visits for evaluation.

Dasgupta K, *et al. BMJ Open* 2022; 12:e063888. doi: 10.1136/bmjopen-2022-063888

The study doctor or a member of the research team will conduct the following tests and procedures:

DESCRIPTION OF STUDY PROCEDURES	
**Procedures at visit 1, 3 and 4**	**Description**
Preparation for visits 1, 3, and 4 (fasting)	To make sure we have accurate results on the tests we are doing, we ask you not to exercise or drink alcohol for two days before you come to see us.<br>We also have to ask you to fast and not smoke the night and following early morning (about 10 hours total) before you come in to have accurate results on blood tests.<br>Do not take your diabetes medications until we give you a snack later in the morning before the exercise test.<br>You can take the other medications that you would normally take at that time of day
Urine collection	We will ask you to go to the washroom to empty your bladder into a container that we will give you. We will use the urine sample to check how your kidneys are working. If you are a woman who is able to become pregnant, we will do a urine test for pregnancy before you start participating in the study. We will ask you to put on a hospital gown.
Height, weight, neck, waist and hip measures	We will ask you change into a hospital gown. We will weigh you (in the gown *without* shoes) and measure your neck, waist, hip and height.
Indirect calorimetry	This test will tell us how much energy you use when you are still. It reflects the amount of energy your body uses to keep your blood flowing and your organs working. You will lie still on a table under a clear hood, staying awake. A machine connected to the hood will measure how much oxygen you are breathing in and how much carbon dioxide you are breathing out.
Review of your medications	You will bring in a list of your medications (with doses) or the medication containers themselves. These will be noted down by study staff. They will ask you some more questions about your medical history and current health.
Blood pressure	We will ask you to sit down quietly for 5 minutes. We will then take one measure from the left arm and then the right arm. Then six measures will be taken 1-minute apart in the arm that had the higher value on the first measurement.
Bioelectrical Impedance Analysis (BIA) test	This test will estimate your body fat and 'lean' mass (muscle) by running a small electrical current through the body that you will not feel. You will stand on the machine and hold onto the bars.
Blood collection	We will take some blood (about 34 mL or roughly 7 teaspoons) to measure the amount of sugar and fat in your blood and other blood tests related to type 2 diabetes and the heart.
Fitness test	We will give you a snack (or if this is visit 3, a lunch) before the fitness test.

	You will be walking/running on a treadmill. The speed will remain the same but the incline of the treadmill will slowly increase. We will ask you to keep going as long as you can to figure out how fit you are. It will feel feel like climbing up a hill that gradually becomes steeper and steeper. You will be asked to breathe through a mask in order to collect and measure the carbon dioxide you breathe out and the oxygen you breathe in.
**Procedures at visit 2 and 5**	
Preparation for Visits 2 and 5	Please do not smoke, consume caffeine-containing beverages or foods (coffee, tea, cocoa, chocolate, “energy drink” or cola) for 12 hours before MRI visit and do not eat for 4 hours before the MRI Visit. If you are on a type of medication for blood pressure called a beta-blocker, they study doctor will ask you to not take it 12 hours before the MRI.
MRI	Magnetic Resonance Imaging (MRI) is an imaging method that uses a magnetic field with radio frequency waves, which allows doctors to see your organs inside your body. Before your scan, we will figure out if you have been taking a medication called a beta-blocker for high blood pressure; if yes, on the day of the scan, we will ask you to delay taking it until after the can. During the scan, you will lie on your back inside the scanner. The inside of the machine is quite small. Some people may start to feel claustrophobic. When the scanner is working, it makes loud buzzing sounds. Therefore, you will wear headphones. We will use the scanner to take pictures of the heart. We will also measure how much fat you have in and around your organs. The MRI will produce images and the computer will collect this information. Some images will require for you to hold your breath for a short period of time (2-15sec) and the technologist will give you specific instructions. It is very important that you don’t move when the picture is taken, as this can blur the pictures. Your heart, blood pressure, and blood oxygen levels will be closely monitored during the test. Electrocardiogram (ECG) electrodes (stickers) will be placed on your chest. A blood pressure cuff will be placed around your upper arm. A pulse oximeter (a small device to measure the amount of oxygen in your blood) will be attached to one of your fingers.
DXA (Dual Energy X-ray Absorptiometry) scan	This test uses X-rays to measure the overall amount of fat and muscle you have in your body. We will also ask you to stay as still as possible. You will lie on a padded table. There will be a machine that makes X-rays below the table. There will be a moveable machine ‘arm’ above the table that detects the X-rays. The arm will move across your body above you.
Physical function tests	After a snack, you will do a few tests that will measure hand grip strength, balance, time it will take you to walk 4 metres and time it takes for you to rise from a chair.

Dasgupta K, *et al. BMJ Open* 2022; 12:e063888. doi: 10.1136/bmjopen-2022-063888

The schedule of procedures for each visit is listed below:

	For everyone in the study (Group 1 and Group 2)					
**Procedure**	**Prior to visit 1**	**Visit 1**	**Visit 2**	**Visit 3**	**Visit 4**	**Visit 5**
**Duration of in-person visit (hrs:min)**	**At home**	**4:00**	**2:10**	**5:30**	**4:30**	**2:10**
**Timeline**			Done within 14 days of visit 1	12 weeks after visit 1	24 weeks after visit 1	24 weeks after visit 1
Urine test		X		X	X	
Height, weight, neck, waist and hip measures		X		X	X	
Indirect calorimetry test		X		X	X	
Pregnancy test (if necessary)		X		X	X	
Medication list review		X		X	X	
Blood pressure		X		X	X	
Bioelectrical Impedance Analysis (BIA)		X		X	X	
Fasting, blood test		X		X	X	
Snack given to participant		X			X	
Lunch given to participant				X		
Fitness test		X		X	X	
Wear a device to monitor your activity & sleep	X			X	X	
Food diary	X			X	X	
Online questionnaire (at-home)	X			X	X	
MRI			X			X
DXA			X	X		X
Snack given to participant			X	X		X
Physical function test			X	X		X

**For those in the Group 2 (low calorie diet and exercise group)**													
**Week #**	**0**	**1**	**2**	**3**	**4**	**8**	**10**	**12**	**14**	**16**	**18**	**21**	**24**
Session with study physician or health care professional	x	x	x		x	x		x	x		x		x
Blood pressure, weight and fasting glucose measure		x	x		x	x		x	x		x		
Blood glucose monitoring (finger prick testing)	x	x	x	x	x	x	x	x	x	x	x	x	x
Sessions with the dietitian	x	x	x		x	x		x	x	x	x	x	x
Supervised exercise training*				x	x	x	x	x					
Home-based exercise**									x	x	x	x	x
CGM sensor	x	x	x	x				x***					x***

*= At least two supervised exercise sessions weekly from weeks 3 to 12.
** At least one unsupervised/home-based exercise session weekly from weeks 3 to 12 and three sessions weekly or equivalent from weeks 13 through 24
*** Each time you will wear it for two weeks (weeks 11 and 12; weeks 23 and 24)

BENEFITS ASSOCIATED WITH THE RESEARCH STUDY

You may or may not personally benefit from your participation in this research project. However, we hope that the study results will contribute to the advancement of scientific knowledge in the study field and help us find better treatments for patients.

RISKS ASSOCIATED WITH THE RESEARCH STUDY

A possible risk associated with this study is a breach of confidentiality or use of your personal information by a third party. To limit this risk, we will take the steps to protect your confidentiality described in the Confidentiality section, below.

1. **Risks associated with the research intervention (group 2 only)**

Physical activity and exercise: With exercise, there are risks of injury to joints, bones, and muscles. If you wind up in the low energy diet and structured exercise group, during the supervised exercise sessions, you will receive advice on how to be safely active. You will participate in some exercises and may try out some gym equipment. Becoming physically active and exercising may sometimes lead to injury or bring to light health problems that were not obvious. You must inform study personnel and physician of any symptoms of shortness of breath, chest discomfort, or other pain. The study physician may have to further evaluate you. Our exercise specialists will ask you about any joint, muscle, or back problems that you may have. They will help you to tailor your exercise accordingly. It is your responsibility to follow their advice and inform them of any concerns you may have.

Low energy diet: In past studies, the low energy diet has led to symptoms like constipation (18 to 47%), dizziness (4 to 32%), fatigue (11 to 25%), thirst (6%), and/or headache (8%). These tend to get better with fibre-based laxatives and time. We will be giving you a fibre-based laxative to use as needed. It is also important to drink as much water as needed when on the diet.

Stopping medications: We will be stopping your diabetes and blood pressure medications at the beginning of the diet and exercise program. This will help to prevent drops in blood pressure and/or glucose that would happen if the low energy diet were combined with medications. Diet and exercise may lead to low blood sugar and low blood pressure, but this is unlikely when not taking medication. We will monitor your blood sugar and blood pressure levels with you during the study to see if at any point medications need to be restarted.

2. **Risks associated with research procedures.**

Blood tests: The taking of blood samples may cause some discomfort, fainting, formation of a small blood clot or swelling of the vein on surrounding tissue, bleeding from the puncture site, and /or rarely an infection. There is a possibility that you may faint. However, precautions will be taken to ensure your safety should this occur.

Treadmill testing: As with any type of strenuous activity, there is a some risk of a serious event (e.g. heart attack, hypoglycemia) during the exercise tests that you will perform during the study. If at any time during the exercise tests you do not wish to continue for any reason, you may stop exercising. A health professional and physician will always be available during the treadmill test and emergency equipment will always be available in case of a serious event.

Body composition DXA scans: These procedures like X-rays use radiation energy to form images of your body and provide your doctor with other clinical information. Radiation can cause cell damage that may, after many years or decades, turn cancerous. We are all at risk of developing cancer during our lifetime. The normal risk is that this will happen to about 50% of people at some point in their life. Taking part in this study will add only a very small chance of this happening to you.

MRI: We will not perform an MRI on pregnant women. The magnetic field of the MRI may pose a risk to the developing fetus. The MRI table, on which you will lie on, will slide inside the MRI scanner. Less than 5% of people have anxiety or a feeling of claustrophobia because of the small space that they are in during the test. The scanner will emit radio frequencies that generate a loud noise, which will be lessened by the headphones we will give you to wear. During the exam, you can talk to the technologist or you can ask to stop the exam at any time. About 10% of people have headache or nausea related to the strong magnetic field of the MRI; these are usually mild and they go away. Some people may also experience a sensation of heat, tingling in the body, nausea and discomfort due to the loud noise.

Questionnaires: Some questions may upset some participants. For example, we will ask you about use of drugs and alcohol, past history of miscarriage, and symptoms of pain, anxiety, and depression. Please let us know if this upsets you and how we can help to support you. We may be able to help you find online resources or places to seek counselling, if necessary. We could also assist you in communicating any issues to your doctor.

Glucose sensors: There is a low risk of infection or bruising from the insertion of the sensor. These could last for a few days. The skin of some individuals is sensitive to the adhesive and can get red or itchy when the CGM is attached. There may be redness in the area where the tape was applied but this will usually disappear after a few days.

RISKS ASSOCIATED WITH PREGNANCY

The approach we are using has not been tested for safety in pregnant women. Therefore, it is important not to become pregnant during the study. In addition, if you are sexually active, and could become pregnant you must use a medically accepted contraceptive method throughout your participation in the study.

The medically accepted contraceptive methods are oral contraception, hormonal implants, hormonal patches, IUD, diaphragm and spermicide, cervical cap with spermicide, and condom with spermicide. The study doctor or the research team will discuss your contraceptive method with you to ensure that it is medically accepted.

If you suspect that you have become pregnant during your participation in the research study, you must inform the study staff immediately. The study doctor and staff will help arrange testing and

Dasgupta K, *et al. BMJ Open* 2022; 12:e063888. doi: 10.1136/bmjopen-2022-063888

referral, as appropriate. If you are in Group 2, we will stop the low energy diet and supervised exercise program. For either Group 1 or Group 2, we will not do the other test procedures like MRI or treadmill testing.

VOLUNTARY PARTICIPATION AND THE RIGHT TO WITHDRAW

Your participation in this research project is voluntary. Therefore, you may refuse to participate. You may also withdraw from the project at any time, without giving any reason, by informing the study doctor or a member of the research team.

Your decision not to participate in the study, or to withdraw from it, will have no impact on the quality of care and services to which you are otherwise entitled, or on your relationship with the study doctor or clinical team.

The study doctor, the Research Ethics Board, the funding agency, or the Sponsor may put an end to your participation without your consent. This may happen if new findings or information indicate that participation is no longer in your interest, if you do not follow study instructions, or if there are administrative reasons to terminate the project.

If you withdraw or are withdrawn from the study, the information and biological material already collected for the study will be stored, analyzed and used to ensure the integrity of the study.

Any new findings that could influence your decision to stay in the research project will be shared with you as soon as possible.

CONFIDENTIALITY

During your participation in this study, the study doctor and their team will collect and record information about you in a study file. They will only collect information required to meet the scientific goals of the study.

The study file may include information from your medical chart, including your identity, concerning your past and present state of health, your lifestyle, as well as the results of the tests, exams, and procedures that you will undergo during this research project. Your research file could also contain other information, such as your name, sex, date of birth and ethnic origin.

All the information collected during the research project will remain strictly confidential to the extent provided by law. You will only be identified by a code number. The key to the code linking your name to your study file will be kept by the study doctor.

To ensure your safety, a copy of this information and consent form will be placed in your medical chart. As a result, any person or company to whom you give access to your medical chart will have access to this information.

Dasgupta K, *et al. BMJ Open* 2022; 12:e063888. doi: 10.1136/bmjopen-2022-063888

We are conducting this study in collaboration with researchers in the United Kingdom (UK) and Canada. We may share the data collected with them, without any identifying information. This is so we can analyse the results from all the sites participating in this study. This will be done in such a way that you cannot be recognised from it. For example, shared data will not include names, addresses or dates of birth.

The study data will be stored for 25 years after completion of the trial.

The data may be published or shared during scientific meetings; however, precautions will be taken to ensure that it will not be possible to identify you.

You have the right to consult your study file in order to verify the information gathered, and to have it corrected if necessary.

However, in order to protect the scientific integrity of the research project, accessing certain information before the project is ended may require that you be withdrawn from the study.

For auditing purposes, the research study files which could include documents that may identify you may be examined by a person mandated by the study sponsor, the institution, or the Research Ethics Board. All these individuals and organizations adhere to policies on confidentiality.

INCIDENTAL FINDINGS

Material incidental findings are findings made in the course of the study that may have significant impacts on your current or future wellbeing or that of your family members. A material incidental finding concerning you in the course of this research will be communicated to you and to a health professional of your choice.

FUNDING OF THE RESEARCH PROJECT

The researcher and the institution have received funding from the Canadian Institutes of Health Research to conduct this research project.

COMPENSATION

You will receive an amount of $10 per **visit to the Glen hospital** (to cover parking and public transit costs) for each of the 5 study-related visits (see Table titled ***For everyone in the study (Group 1 and Group 2)*****).**

For Group 2, parking costs at PERFORM will be paid directly by the study team to the PERFORM centre. The meal replacements and supervised exercise sessions (where applicable) will be offered to you for free for the duration of this research study.

SHOULD YOU SUFFER ANY HARM

Should you suffer harm of any kind due to a procedure related to the research study, you will receive the appropriate care and services required by your state of health.

By agreeing to participate in this research project, you are not waiving any of your legal rights nor discharging the study doctor, the sponsor or the institution, of their civil and professional responsibilities.

Dasgupta K, *et al. BMJ Open* 2022; 12:e063888. doi: 10.1136/bmjopen-2022-063888

WHAT ABOUT COVID-19?

Your safety and that of our research team will be paramount. The study will comply with the latest recommendations and laws. This includes physical distancing and the use of personal protective equipment. If things change during the course of the study, we may need to change how we run the study. This may mean greater use of remote consultations and monitoring. The amount of exercise done at home may also need to be increased.

CLINICAL TRIAL REGISTRATION

A description of this clinical trial will be available on http://www.ClinicalTrials.gov. This Website will not include information that can identify you. At most, the Website will include a summary of the results. You can search this Website at any moment.

CONTACT INFORMATION

If you have questions or if you have a problem you think may be related to your participation in this research study, or if you would like to withdraw, you may communicate with:

- The Principal Investigator, Dr. Kaberi Dasgupta, at the McGill University Health Centre, at 514-934-1934, then 1 then ext. 44715; or
- The main research coordinator of the study, Ms. Debbie Chan, at 514-934-1934 then 1 then ext. 44835 or by email at debbie.chan@rimuhc.ca

For any question concerning your rights as a research participant taking part in this study, or if you have comments, or wish to file a complaint, you may communicate with McGill University Health Centre Ombudsman: 514-934-1934, then 1 then ext. 35655

OVERVIEW OF ETHICAL ASPECTS OF THE RESEARCH

The McGill University Health Centre Research Ethics Board reviewed this research and is responsible for monitoring the study.

Dasgupta K, *et al. BMJ Open* 2022; 12:e063888. doi: 10.1136/bmjopen-2022-063888

SIGNATURES

Signature of the participant

I have reviewed the information and consent form. Both the research study and the information and consent form were explained to me. My questions were answered, and I was given sufficient time to make a decision. After reflection, I consent to participate in this research study in accordance with the conditions stated above.

I authorize the research study team to have access to my medical record for the purposes of this study.

I authorize the study doctor to inform my treating physician that I am taking part in this study.

I authorize the doctor in charge of this research study to communicate with me directly to ask if I am interested in participating in other research

I understand that my doctor may be contacted to provide information about my health status to the research team for confirmation of eligibility.

Name and contact information of treating physician: ____________________________

Physician's Address: _______________________________

Physician's phone number:_______-________-___________

I understand that the study doctor will send my treating physician health information if it will be useful for my care.

____

Name of participant | Signature | Date (dd/mmm/yyyy)

Signature of the person obtaining consent

I have explained the research study and the terms of this information and consent form to the research participant, and I answered all his/her questions.

____

Name of the person obtaining consent | Signature | Date (dd/mmm/yyyy)

Dasgupta K, *et al. BMJ Open* 2022; 12:e063888. doi: 10.1136/bmjopen-2022-063888
