# Supplementary material for: Remission of type 2 diabetes and improved diastolic function by combining structured exercise with meal replacement and food reintroduction among young adults: the RESET for REMISSION randomised controlled trial protocol
Source: BMJ Open. 2022 Sep 21;12(9):e063888. doi: 10.1136/bmjopen-2022-063888 (PMC9494595; doi:10.1136/bmjopen-2022-063888)
Supplement: Supplementary data [file bmjopen-2022-063888supp003.pdf]

DMC Charter for R4R

Date: 15/01/2021

RESET FOR REMISSION

Author: Thomas Yates and Kaberi Dasgupta

Version: 1.0

RESET 4 REMISSION Trial DMC Charter

1. INTRODUCTION	
Name (and sponsor's ID) of trial plus ISRCTN and/or EUDRACT number	REmission of diabetes and improved diastolic function by combining Structured Exercise with meal replacemenT and food reintroduction: THE RESET FOR REMISSION TRIAL<br>Sponsor: University of Leicester and McGill University<br>Registration number: *To be included*
Objectives of trial, including interventions being investigated	**Primary objective**<br>The aim of this efficacy study is to investigate whether combining a low energy diet incorporating the phased use of meal replacement products, with structured exercise training leads to remission of T2DM in younger adults (18-40 years) over a 24-week period, in comparison with usual care. Remission is defined as an hba1c value less than 6.5% at 24 weeks and no antihyperglycemic medications during weeks 13 through 24 weeks of the 24 week study period.<br><br>**Secondary objectives**<br>Key secondary objectives will examine the effects of the combined intervention on cardiovascular and functional health, particularly MRI-assessed diastolic function and cardiometabolic risk factors such as lipid profile and peripheral blood pressure, along with cardiorespiratory fitness, physical function, lean mass and basal metabolic rate. We will also undertake a process evaluation, to ensure the findings from our research can be used to inform how the interventional components are refined and translated.<br><br>Figure 1 shows a flow chart of the trial design.
Outline of scope of charter	The purpose of this document is to describe membership, terms of reference, roles, responsibilities, authority, decision-making and relationships of the independent Data Monitoring Committee (DMC) for the RESET 4 REMISSION trial. This will include the timing of meetings, methods of providing information to and from the DMC, frequency and format of meetings and relationships with other trial committees such as the Trial Steering Committee (TSC).
**2. ROLES AND RESPONSIBILITIES**	
A broad statement of the aims of the Data Monitoring Committee	To safeguard the interests of trial participants, assess the safety of the interventions during the trial, and monitor the overall conduct of the clinical trial.
Terms of reference	The DMC should receive and review the progress and accruing data of this trial and provide advice on the conduct of the trial to the Trial Steering Committee.
Specific roles of DMC	To undertake reviews of the trial's progress, including updated figures on recruitment, data quality, and main outcomes and safety data, by:<br>• assess data completeness (and by so doing encourage collection of high-quality data)<br>• monitor recruitment figures and losses to follow-up<br>• monitor compliance with the protocol by participants and investigators<br>• monitor evidence for any potential treatment harm (e.g., Serious Adverse Events or deaths)

RESET 4 REMISSION Trial DMC Charter

	• suggest additional data analyses as part of the review of the statistical analysis plan<br>• advise on protocol modifications suggested by investigators or sponsors (e.g., to inclusion criteria, trial endpoints, or sample size)<br>• monitor planned sample size assumptions<br>• monitor continuing appropriateness of participant information sheets<br>• monitor compliance with previous DMC recommendations<br>• assess the impact and relevance of external evidence as requested by the TSC, Trial Sponsors (University of Leicester and Research Institute of the McGill University Health Centre [RI-MUHC]) or Trial Funders (The UK Medical Research Council [MRC] and The Canadian Institutes of Health Research [CIHR])<br>• if funding is required above the level originally requested, the DMC may be asked by the Principal (Chief) Investigators, TSC, Trial Sponsors or Trial Funders to provide advice and, where appropriate, information on the data gathered to date in a way that will not compromise the trial.
**3. BEFORE OR EARLY IN THE TRIAL**	
Whether the DMC will have input into the protocol	All potential DMC members should read the protocol/outline before agreeing to join the committee. Before recruitment begins the trial will have undergone review by the funders (UK Medical Research Council and Canadian Institutes of Health Research), the sponsors (University of Leicester and RI-MUHC), and other relevant trial committees including that of research ethics committees. Therefore, if a potential DMC member has major reservations about the trial (e.g., the protocol or the logistics) they should report these to the Chief Investigators and may decide not to accept their invitation to join. DMC members should be constructively critical of the ongoing trial, but also supportive of aims and methods of the trial.
Whether the DMC will meet before the start of the trial	It is recommended that, if possible, the DMC meets before the trial starts or early in the course of the trial, to discuss the protocol, the trial, any analysis plan, future meetings, and to have the opportunity to clarify any aspects with the principal investigators.
Any issues specific to the disease under study	The study population comprises younger (18 – 40 years) adults with type 2 diabetes. These individuals are at risk of developing chronic (e.g., cardiovascular disease) or acute (e.g. hyperglycaemia) complications related to their diabetes. The protocol lists the possible adverse events of study interventions within this population.
Any specific regulatory issues	None
Any other issues specific to the treatment under study	None
Whether members of the DMC will have a contract	DMC members will not be asked to formally sign a contract but should formally register their agreement to join the group by confirming; (1) that they agree to be a member of the DMC and (2) that they agree with the contents of this Charter. Any potential competing interests, real or potential, should be declared at the same time using a short competing interest form (displayed in **Annex 1**), which should be completed and returned by the DMC members to the trial coordinating centre. DMC members should sign and maintain this log of potential competing interests.

RESET 4 REMISSION Trial DMC Charter

**4. COMPOSITION**	
Membership and size of the DMC	Members of the DMC (including the DMC Chair) should be independent of the trial. The definition of independent is as follows:<br>• Not part of the same institution as any of the applicants or members of the project team<br>• Not part of the same institution that is acting as a recruitment or investigative centre<br>• Not related to any of the applicants or members of the project team<br>• For the chair only – not an applicant on a rival proposal<br>The membership will consist of three individuals including two clinicians experienced in the clinical area and one statistician. Members have been chosen because they are experienced in trials and/or the disease area.<br>The members of the DMC for the RESET 4 REMISSION Trial are:<br>(1) **Dr Janusz Kaczorowski** *(Chair)*, Professor of Family and Emergency Medicine, University of Montreal<br>(2) **Mr Stephen Sharp** (*Statistician*), MRC Epidemiology Unit, University of Cambridge School of Clinical Medicine<br>(3) **Dr Andrew Farmer** (*Clinical Representative*), Professor of General Practice, Nuffield Department of Primary Care Health Sciences, University of Oxford
The Chair, how they are chosen and the Chair's role. (Likewise, if relevant, the vice-Chairman)	The chair is Dr. Janusz Kaczorowski (Professor and Research Director in the Department of Family and Emergency Medicine – University of Montreal) who is serving on the DMC. The Chair is expected to facilitate and summarise discussions. There is no vice-Chair.
The responsibilities of the DMC statistician	The DMC membership includes a statistician (Mr Stephen Sharp) whose role it is to provide independent statistical expertise and to further guide the other DMC members through the report. The DMC statistician is not expected to prepare the DMC report.
The responsibilities of the trial statistician	The trial statistician (Dr Elham Rahme) will produce (or oversee the production of) the report to the DMC and will participate in DMC meetings, guide the DMC through the report, may participate in DMC discussions and, on some occasions, taking notes.
The responsibilities of the trial office team	The trial management team (e.g., Trial Managers/Coordinators) will input to the production of the DMC report.
The responsibilities of the PI and other members of the Trial Management Group (TMG)	The PI's (Professor Thomas Yates and Professor Kaberi Dasgupta), may be asked, and should be available, to attend open sessions of the DMC meeting. The other TMG members will not usually be expected to attend but can attend open sessions when necessary (see Section 6 regarding 'Organisation of DMC Meetings' below).
**5. RELATIONSHIPS**	
Relationships with Principal Investigators, other trial committees (eg Trial Steering Committee (TSC) or Executive Committee), sponsor and regulatory bodies	The DMC are advisory to the Trial Steering Committee (which includes the PI's). The TSC is the executive body for the trial.
Clarification of whether the DMC is	The DMC is advisory to the Trial Steering Committee. The TSC is the executive

RESET 4 REMISSION Trial DMC Charter

advisory (make recommendations) or executive (make decisions)	body for the trial.
Payments to DMC members	Members will be reimbursed for reasonable travel costs and other expenses incurred, but it is anticipated that the meetings with be virtual. No other payments or rewards would be given to professional members.
The need for DMC members to disclose information about any competing interests	Competing interests should be disclosed. These are not restricted to financial matters – involvement in other trials or intellectual investment could be relevant. Although members may well be able to act objectively despite such connections, complete disclosure enhances credibility (See **Annex 1** towards end of DMC Charter document).<br><br>DMC members should not use interim results to inform trading in pharmaceutical shares, and careful consideration should be given to trading in stock of companies with competing products. Changes in declarations of real or potential competing interests should be documented within the minutes at the start of each meeting.
**6. ORGANISATION OF DMC MEETINGS**	
Expected frequency of DMC meetings	The DMC should meet 6-monthly to annually, or more often as appropriate either in person or via teleconference. Some trial issues may need to be dealt with between meetings, by phone or by email. DMC members should be prepared for such instances. Additional *ad hoc* meetings will be scheduled with the occurrence of any severe adverse event including death, hospitalization, or emergency room consultation. During these meetings, reports from the PIs will be reviewed, regarding the SAE in question and the relatedness to trial procedures. The DSC will then determine whether further adjudication is warranted and what type of expertise is required; in these instances, two physicians from the relevant research site will be invited to review the event and medical records and comment on relatedness. If there is a difference of opinion, a third physician will be invited to review. The physicians may be trial investigators but they must be blinded to the trial arm and cannot have interacted with the participant during the course of the trial.<br><br>Regularly-scheduled meetings should be timed approximately 1 month prior to TSC meetings, so that reports can be fed to the TSC accordingly.
Whether meetings will be face-to-face or by teleconference	Given the international context, the default will be to hold meetings over videoconference. Effort will be made to ensure that all members can attend.
How DMC meetings will be organised, especially regarding open and closed sessions, including who will be present in each session	Upon request by the DMC, the format will be an open session throughout where all parts of the report are discussed and there is DMC discussion. The DMC members can ask for a closed session at any point in the DMC (± trial statistician at discretion of the DMC chair).
**7. TRIAL DOCUMENTATION AND PROCEDURES TO ENSURE CONFIDENTIALITY AND PROPER COMMUNICATION**	
Intended content of material to be available in sessions	Accumulating information relating to recruitment and retention, and data quality (e.g., data return rates, treatment compliance) will be presented by intervention arm. No formal interim analyses are planned A log of adverse events will be presented at each meeting and discussed. Outcome data will not be presented. However, if the DMC has any concerns related to safety, pooled outcome data may be presented, and, in discussion with the TSC, unblinding may be permitted.

RESET 4 REMISSION Trial DMC Charter

Will the DMC be blinded to the treatment allocation	The DMC will not be blinded to recruitment data by treatment arm. No formal interim analyses are planned. The DMC will remain blinded unless safety concerns arise. However, primary and secondary outcome data will be based on pooled data, and the DMC will remain blinded unless unblinding is requested related to concerns about safety.
Who will see the accumulating data	There are no planned interim analyses. The accumulating data presented to the DMC by intervention arm will relate only to attrition and to safety. If the DMC deems that safety concerns then unblinding may occur as indicated above. Interim data and analyses by intervention/control group (and the deliberations of the DMC) should be available only to those present in the DMC meetings when the discussions occurred: i.e., only members of the DMC, the trial statistician and other members of the trial team, as agreed by the DMC. DMC members must not share confidential information with people outside the DMC.
Who will be responsible for identifying and circulating external evidence (e.g., from other trials/ systematic reviews)	Identification and circulation of external evidence (e.g., from other trials/ systematic reviews) is not the responsibility of the DMC members. It is the responsibility of the TMG (Trial Management Group). However, the DMC should continue to be made aware of other data that may impact the trial.
To whom the DMC will communicate the decisions/ recommendations that are reached	The DMC will report its recommendations via the chair in writing to the Trial Steering Committee (TSC, please see **Annex 2**). This should be sent in time for consideration at a TSC meeting. If the trial is to continue largely unchanged then it is often useful for the report from the DMC to include a summary paragraph suitable for trial promotion purposes.
Whether reports to the DMC be available before the meeting or only at/during the meeting	It is planned that the DMC will receive data monitoring reports (i.e., recruitment, attrition, numbers in various study stages, numbers completed) from the TMG at least 2 weeks before scheduled meetings.
What will happen to the confidential papers after the meeting	The DMC members should store the papers safely after each meeting so they may check the next report against them. After the trial is reported, the DMC members should destroy all interim reports.
**8. DECISION MAKING**	
What decisions/recommendations will be open to the DMC	Possible recommendations to the TSC could include:<br>• No action needed; trial continues as planned<br>• Early stopping due, for example, to clear harm of a treatment or external evidence (this should generally involve a recommendation from the DMC to unblind the TSC to this data)<br>• Modifying target recruitment, or pre-analysis follow-up, based on any change to the assumptions underlying the original trial sample size calculation (but not on any emerging differences)<br>• Sanctioning and/or proposing protocol changes<br><br>Ultimately, decisions will be made by the TSC, as the DMC's role is advisory.
The role of formal statistical methods, specifically which methods will be used and whether they will be used as guidelines or rules	The DMC and TSC will be asked to comment on and approve the trial statistical analysis plan (SAP) before database lock.
How decisions or recommendations will be reached within the DMC	Every effort should be made to achieve consensus. The role of the DMC Chair is to summarise discussions and encourage consensus; therefore, it is usually best for the Chair to give their own opinion last.

RESET 4 REMISSION Trial DMC Charter

	It is important that the implications (e.g., ethical, statistical, practical, financial) for the trial be considered before any decision is made. Only appointed members will be entitled to vote and the chair will have a casting vote.
When the DMC is quorate for decision-making	Effort should be made for all DMC members to attend. The TMG will try to ensure that a date is chosen to enable this. Members who cannot attend in person should be encouraged to attend by teleconference. If, at short notice, any DMC members cannot attend at all then the DMC may still meet if at least two members will be present (including the Chair). If the DMC is considering recommending major action after such a meeting, the DMC Chair should talk with the absent members as soon after the meeting as possible to check whether they agree with such action. If they do not, a further teleconference should be arranged with the full DMC.
Can DMC members who cannot attend the meeting input	If the report is circulated before the meeting, DMC members who will not be able to attend the meeting may pass comments to the DMC Chair for consideration during the discussions.
What happens to members who do not attend meetings	If a member does not attend a meeting, it should be ensured that the member is available for the next meeting. If a member does not attend a second meeting, they should be asked if they wish to remain part of the DMC. If a member does not attend a third meeting, they should be replaced.
Whether different weight will be given to different endpoints (e.g., safety/efficacy)	This study is unlikely to result in safety issues, therefore greater weight should be given to efficacy considerations.
Any specific issues relating to the trial design that might influence the proceedings, eg cluster trials, equivalence trials, multi-arm trials	This is a 2-arm trial, occurring in the UK (Leicester) and Canada (Montreal, Edmonton). It is likely that the TSC/ DMC will need to consider issues specific to UK or Canadian sites in equivalent trials.
**9. REPORTING**	
To whom will the DMC report their recommendations/decisions, and in what form	The DMC Chair should report in writing to the Chair of the Trial Steering Committee, usually within 3 weeks after the meeting. Unless the DMC is recommending that the trial protocol be changed in some way, the letter to the TSC should not reveal any confidential information. An example of a letter from a DMC to the TSC recommending no action is presented in **Annex 2**. Additionally, the letter should be copied to the PIs, trial statistician and trial managers.
Whether minutes of the meeting be made and, if so, by whom and where they will be kept	Minutes including key points and actions will be made by a member of the trials' office team (to be decided prior to each meeting). This will include details of whether any competing interests have arisen/changed for any attendees since the previous meeting. The draft minutes will be initially circulated for comment to all DMC members who were present at the meeting. The DMC Chair will then sign off the final version of minutes. The final signed version will then be sent to all members, the sponsor, the funder and stored in the trial master file.
What will be done if there is disagreement between the DMC and the body to which it reports	The TSC has ultimate responsibility for the trial and assumes primacy. However, the TSC should report to the DMC regarding how they have acted upon the DMC's recommendations. If the DMC has serious problems or concerns with the TSC decision, a joint meeting of the TSC and DMC should be held. The information to be shown would depend upon the action proposed and each committee's concerns. The meeting would be Chaired by

RESET 4 REMISSION Trial DMC Charter

	a senior member of the trials office staff or an external expert who is not directly involved with the trial.
**10. AFTER THE TRIAL**	
Publication of results	The PIs have responsibility that trial results will be published in a correct and timely manner. The TSC is the committee that should oversee this process.
The information about the DMC that will be included in published trial reports	DMC members will be named and their affiliations listed in the main report, unless they explicitly request otherwise.
Whether the DMC will have the opportunity to approve publications, especially with respect to reporting of any DMC recommendation regarding termination of a trial	The DMC members should be given at least 2 weeks to read and comment on any draft publications that report the primary outcome measure and/or details of the DMC. This may be done simultaneously to other groups reviewing the draft manuscript (e.g. Trial Steering Committee, trial investigators).
Any constraints on DMC members divulging information about their deliberations after the trial has been published	The DMC should not discuss confidential issues from their involvement in the trial until 12 months after the primary trial results have been published, unless permission is agreed with the TSC. The TSC will also decide when it is appropriate for TMG and DMC members to trade in related stocks.

DMC Charter for R4R

Date: 15/01/2021

Author: Thomas Yates and Kaberi Dasgupta

Version: 1.0

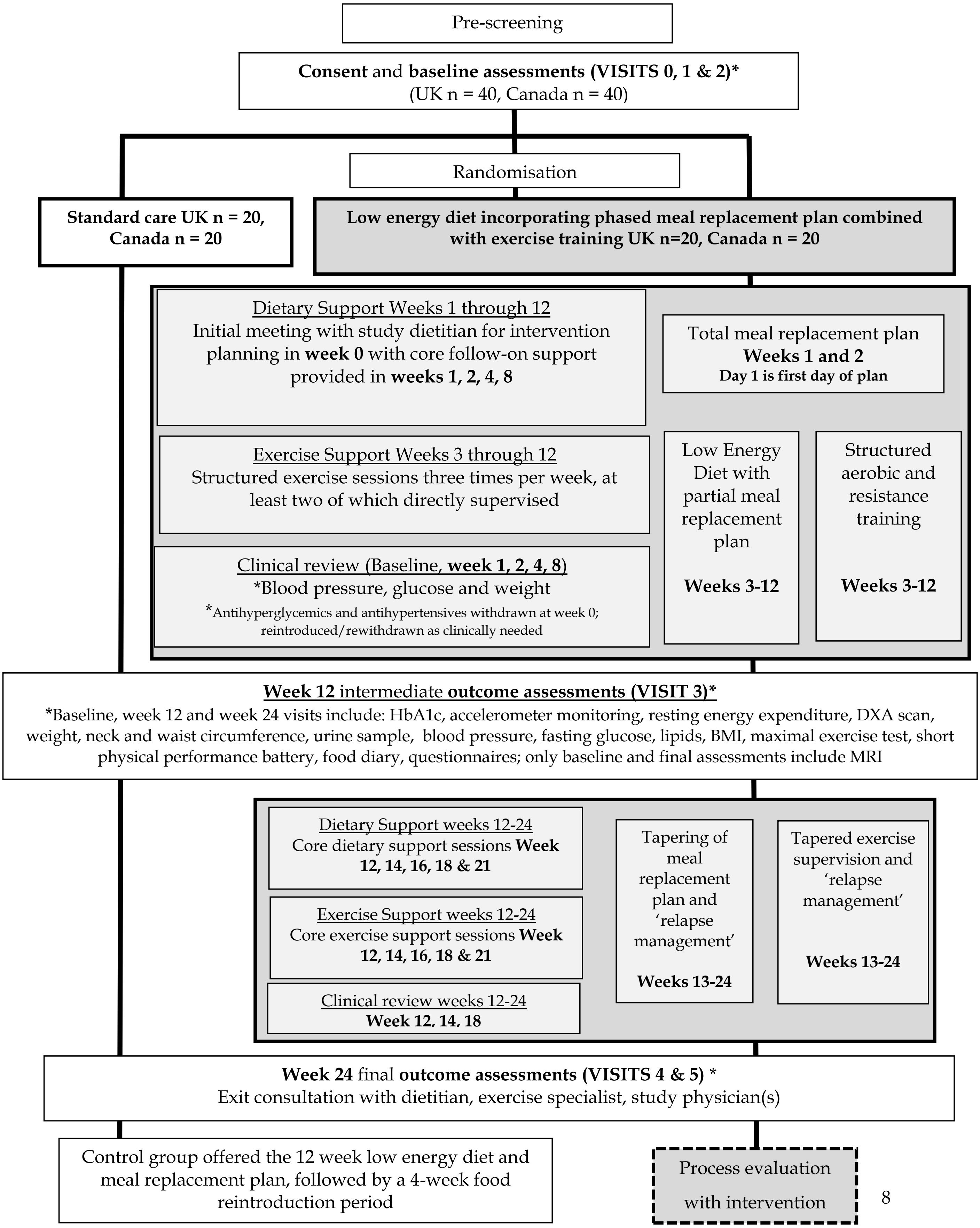

Dasgupta K, *et al. BMJ Open* 2022; 12:e063888. doi: 10.1136/bmjopen-2022-063888

Annex 1: Suggested competing interests form

RESET 4 REMISSION Trial (Sponsor: University of Leicester and McGill University): Agreement to join the Data Monitoring Committee as an independent member and disclosure of potential competing interests

Please complete the following document and return to the Trials Office

(Please initial box to agree)

☐	I have read and understood the DMC Charter version 1.0, dated 15th March 2021
☐	I agree to join the Data Monitoring Committee for this trial as an independent member
☐	I agree to treat all sensitive trial data and discussions confidentially

The avoidance of any perception that independent members of a DMC may be biased in some fashion is important for the credibility of the decisions made by the DMC and for the integrity of the trial.

Potential competing interests should be disclosed via the trials office. In many cases simple disclosure up front should be sufficient. Otherwise, the (potential) independent DMC member should remove the conflict or stop participating in the DMC. **Table 1** lists potential competing interests.

☐ **No,** I have no potential competing interests to declare
☐ **Yes,** I have potential competing interests to declare (please detail below)

Please provide details of any potential competing interests:

____

____

____

Name: ____________________________

Signed: ___________________________ Date: _______________

Table 1: Potential competing interests for independent members

- Stock ownership in any commercial companies involved
- Stock transaction in any commercial company involved (if previously holding stock)
- Consulting arrangements with the Sponsor/Funder
- Ongoing advisory role to a company providing drugs to the trial
- Frequent speaking engagements on behalf of the intervention
- Career tied up in a product or technique assessed by trial
- Hands-on participation in the trial
- Involvement in the running of the trial
- Emotional involvement in the trial
- Intellectual conflict e.g. strong prior belief in the trial’s experimental arm
- Involvement in regulatory issues relevant to the trial procedures
- Investment (financial or intellectual) or career tied up in competing products
- Involvement in the writing up of the main trial results in the form of authorship

Annex 2: Suggested report from DMC to TSC where no recommendations are being made

[Insert date]

To: Professor XX (Chair of Trial Steering Committee)

Dear Professor XX,

The Data Monitoring Committee (DMC) for the RESET 4 REMISSION Trial met on *[meeting date]* to review its progress and interim accumulating data. *[List members] attended* the meeting and reviewed the report.

We congratulate the trial organisers and collaborators on the progress and conduct of the trial and the presentation of the data. The trial question remains important and, on the basis of the data reviewed at this stage, we recommend continuation of the trial according to the current version of the protocol *[specify protocol version number and date]* with no changes.

We shall next review the progress and data *[provide approximate timing]*

Yours sincerely,

Professor XX
Chair of Data Monitoring Committee

On behalf of the DMC (all members listed below)

DMC members:
(1) xx
(2) xx
(3) xx

TSC Charter for R4R | Author: Kaberi Dasgupta and Thomas Yates

Date: 13/01/2021 | Version: 1.0

RESET FOR REMISSION TRIAL TSC CHARTER

**1. INTRODUCTION**	
Name (and sponsor's ID) of trial plus ISRCTN and/or EUDRACT number	REmission of diabetes and improved diastolic function by combining Structured Exercise with meal replacemenT and food reintroduction: THE RESET FOR REMISSION TRIAL<br>Sponsor: University of Leicester and McGill University<br>Registration number: *To be included*
Objectives of trial, including interventions being investigated	**Primary objective**<br>The aim of this efficacy study is to investigate whether combining a low energy diet incorporating the phased use of meal replacement products, with structured exercise training leads to remission of T2DM in younger adults (18-40 years) over a 24 week period. Remission is defined as an HbA1c value less than 6.5% without antihyperglycemic medications during weeks 13 through 24 weeks of the 24 week study period.<br><br>**Secondary objectives**<br>Key secondary objectives will examine the effects of the combined intervention on cardiovascular and functional health, particularly MRI-assessed diastolic function and cardiometabolic risk factors such as lipid profile and peripheral blood pressure, along with cardiorespiratory fitness, physical function, lean mass and basal metabolic rate. We will also undertake a process evaluation, to ensure the findings from our research can be used to inform how the interventional components are refined and translated.<br><br>Figure 1 shows a flow chart of the trial design.
Outline of scope of charter	The purpose of this document is to describe the membership, terms of reference, roles, responsibilities, authority, decision-making and relationships of the Trial Steering Committee (TSC) for this trial, including the timing of meetings, methods of providing information to and from the TSC, frequency and format of meetings and relationships with other trial committees.
**2. ROLES AND RESPONSIBILITIES**	
A broad statement of the aims of the TSC	To act as the oversight body for the Reset For Remission Trial on behalf of the Sponsor/Funder.
Terms of reference	The role of the TSC is to provide oversight for the trial. It should also provide advice, through its Chair, to the CIs (PIs), the Sponsors (University of Leicester and Research Institute of the McGill University Health Centre), the Funders (The Canadian Institutes of Health Research [CIHR] and the UK Medical Research Council [MRC]), and the Host Institution on all appropriate aspects of the trial but with a particular focus on:<br>• Progress of the trial, adherence to the protocol, participant safety and the consideration of new information of relevance to the research question.

BMJ Publishing Group Limited (BMJ) disclaims all liability and responsibility arising from any reliance placed on this supplemental material which has been supplied by the author(s)

**RESET FOR REMISSION TRIAL TSC CHARTER**	
	• The rights, safety and well-being of the trial participants are the most important considerations and should prevail over the interests of science and society.<br>• To ensure appropriate ethical and other approvals are obtained in line with the project plan<br>• To review proposals for substantial protocol amendments and provide advice to the sponsor and funder regarding approvals of such amendments, where appropriate.<br>• To provide advice to the investigators on all aspects of the trial.
Specific roles of TSC	The specific roles of the TSC as a body will be to:<br>• provide expert oversight of the trial<br>• make decisions as to the future continuation (or otherwise) of the trial<br>• monitor recruitment rates and encourage the Trial Management Group (TMG) to develop strategies to deal with any recruitment problems<br>• review regular reports of the trial from the trials coordinators (sent on behalf of the TMG)<br>• receive letters of feedback from the DMC (Data Monitoring Committee) and consider their recommendations<br>• assess the impact and relevance of any accumulating external evidence (provided by the TMG)<br>• monitor follow-up rates and review strategies from TMG to deal with problems<br>• ensure sites that are complying with the protocol<br>• approve major amendments to the protocol<br>• approve any proposals by the TMG concerning any change to the design of the trial, including additional sub studies<br>• oversee the timely reporting of trial results<br>• comment on the statistical analysis plan<br>• comment on the publication policy<br>• comment on the main trial manuscript<br>• approve and comment on any abstracts and presentations of any results *during* the running of the trial<br>• approve external or early internal requests for release of data or subsets of data or samples including clinical data and stored biological samples<br>It is the responsibility of individual members of the TSC to maintain confidentiality of all trial information that is not already in the public domain.
**3. BEFORE OR EARLY IN THE TRIAL**	
TSC input into the protocol	All potential independent TSC members should have opportunity to comment on the protocol as early as possible. If a potential independent TSC member has major reservations about the trial (e.g., the protocol, the logistics, ethical concerns) they should report these to the TMG and may decide not to accept the invitation to join. TSC members should be constructively critical of the protocol, but also supportive of aims and methods of the trial.
Any issues specific to the disease under study	The study population comprises younger (18 – 40 years) adults with type 2 diabetes. These individuals are at risk of developing chronic (e.g. cardiovascular disease) or acute (e.g. hyperglycaemia) complications related

RESET FOR REMISSION TRIAL TSC CHARTER

	to diabetes. The protocol lists the possible adverse events of study interventions within this population
Any specific regulatory issues	None
Any other issues specific to the treatment under study	None
Whether members of the TSC will have a contract	TSC members will not be asked to formally sign a contract but should formally register their agreement to join the group by confirming (1) that they agree to be a member of the TSC and (2) that they agree with the contents of this Charter. Any potential competing interests should be declared at the same time. Members should complete and return the form in Annex 1 or 2 by email. Any observers (attendees who are not members) will sign a confidentiality agreement on the first occasion they attend a meeting (Annex 3).
**4. COMPOSITION**	
Membership and size of the TSC	Apart from the studies PIs (Dasgupta, Yates), the members of the TSC, including the Chair, should be independent of the trial. Non-independent members will also be part of the TSC. The definition of independent is as follows:<br>• Not part of the same institution as any of the applicants or members of the project team<br>• Not part of the same institution that is acting as a recruitment or investigative centre<br>• Not related to any of the applicants or members of the project team<br>• For the chair only – not an applicant on a rival proposal<br><br>The membership will consist of three individuals including two clinicians experienced in the clinical area and one statistician. Members have been chosen because they are experienced in trials and/or the disease area.<br>The members of the TSC for this trial are:<br>(1) **Professor Jason Gill** (Chair), Professor of Cardiometabolic Health, University of Glasgow<br>(2) **Dr Alice Cheng** (Clinical representative), Physician and Associate Professor of Medicine, University of Toronto<br>(3) Dr Elham Rahme (Trial statistician)<br>(4) Professor Kaberi Dasgupta and Prof Tom Yates, PIs<br><br>Only appointed members will be entitled to vote. The PIs or their delegates are allocated one vote in total.
The Chair	The chair is Prof Jason Gill (Professor of Cardiometabolic Health at the University of Glasgow) who is serving on the TSC. The Chair is expected to facilitate and summarise discussions. There is no vice-Chair.

RESET FOR REMISSION TRIAL TSC CHARTER	
	The Chair is directly answerable to the Sponsor and Funders. The Chair's responsibilities include:<br>• Arranging, along with the PIs and trials office, an inaugural meeting to set up a schedule of meetings to align with the project plan<br>• Being familiar with relevant guidance documents and with the role of the DMC<br>• Providing an independent, experienced opinion if conflicts arise between the needs of the research team, the funder, the sponsor, the participating organisations and/or any other agencies<br>• Leading the TSC to provide regular, impartial oversight of the trial, especially to identify and pre-empt problems<br>• Ensuring that changes to the protocol are debated and endorsed by the TSC, letters of endorsement should be made available to the project team when requesting approval from the funder and sponsor for matters such as substantive changes to protocol<br>• Being available to provide independent advice as required, not just when TSC meetings are scheduled<br>• Commenting on any extension requests and, where appropriate, providing a letter of recommendation to accompany such a request<br>• Commenting in detail, when appropriate, regarding the continuation or termination of the project
The responsibilities of the trial office team	The trials office team will produce a short report on the trial before each meeting of the TSC. A template for the report will be followed.
The responsibilities of the PI and other members of the Trial Management Group (TMG)	The PIs is an important member of the TSC and no major decisions should be made without their involvement.
The responsibilities of the observers	Additional observers may be in attendance through (parts of) the TSC meetings in order to provide input on behalf of the trials office, the trial's Sponsor/Funder or to provide specific relevant expertise.
**5. RELATIONSHIPS**	
Advisory and executive bodies	The TSC is the oversight body (see *Roles and responsibilities* above). All substantial issues regarding the trial must go to the TSC for consideration. The DMC is advisory to the TSC.
Payments to TSC members	Members will be reimbursed for reasonable travel costs and other expenses incurred. No other payments or rewards would be given to professional members.
The need for TSC members to disclose information about any competing interests	Competing interests should be disclosed. These are not restricted to financial matters – involvement in other trials or intellectual investment could be relevant. Although members may well be able to act objectively despite such connections, complete disclosure enhances credibility (See Annex 1).<br>TSC members should not use interim results to inform trading in pharmaceutical shares, and careful consideration should be given to trading

RESET FOR REMISSION TRIAL TSC CHARTER	
	in stock of companies with competing products. Changes in declarations of real or potential competing interests should be minuted at the start of each meeting.
**6. ORGANISATION OF TSC MEETINGS**	
Expected frequency of TSC meetings	The TSC will meet at 6-monthly to annually depending on need as determined by the Chair, either in person or via teleconference. At the request of the TSC, interim meetings, in person or by teleconference, will be organised. Some trial issues may need to be dealt with between meetings, by phone or by email. TSC members should be prepared for such instances.<br>It is expected that the TSC will meet approximately one month after the DMC.
Attendance of TSC members at meetings	Given the international context, the default will be to hold meetings over videoconference. Effort will be made to ensure that all members can attend. At least one PI must attend all meeting. If, at short notice, any TSC members cannot attend then the TSC may still meet if at least two independent members will be present, along with a PI. There may be occasions when the Trial Sponsors or Funders will wish to organise and administer these meetings.
How TSC meetings will be organised, especially regarding open and closed sessions, including who will be present in each session	Presence will be usually limited to the TSC members, and observers from the TMG. Other attendees may be invited for all or part of the meeting by the TSC Chair. Meeting will be organised by Trials office, in coordination with the PIs and Chair.
Input from TSC members who cannot attend the meeting	If the report is circulated before the meeting, TSC members who will not be able to attend the meeting may pass comments to the TSC Chair or trials office contact for consideration during the discussions.
What happens to independent members who do not attend meetings	If an independent member does not attend a meeting or provide comments when requested between meetings, it should be ensured that the independent member is available for the next meeting. If an independent member does not attend the next meeting or provide comments when next requested, they should be asked if they wish to remain part of the TSC. If an independent member does not attend a third meeting, strong consideration should be given to replacing this member.
**7. TRIAL DOCUMENTATION AND PROCEDURES TO ENSURE CONFIDENTIALITY AND PROPER COMMUNICATION**	
Intended content of material to be considered during meetings	A short report will be prepared by the trial coordinators following a standard template. This will report on accrual and any matters affecting the trial. Additionally, the material may include a report *from* the DMC, requests *from* the TMG or draft publications. No trial outcome measure data will be presented by arm unless explicitly authorised by the DMC (e.g., safety outcomes). If specifically requested by the TSC, accrual, compliance with follow-up and adherence to treatment may be presented by site.
Whether reports to the TSC will be available before the meeting or only at/during the meeting	It is planned that the TSC will receive the report at least 1 week and preferably at least 2 weeks before any meetings.
Responsibility for identifying and	Identification and circulation of external evidence (e.g., from other trials/

RESET FOR REMISSION TRIAL TSC CHARTER	
circulating external evidence (e.g. from other trials/ systematic reviews)	systematic reviews) is not the responsibility of the TSC members; it is a responsibility of the TMG. However, the TSC should continue to be made aware of other data that may impact on a trial.
What will happen to the papers after the meeting	TSC members will be expected to securely store copies of the reports to and from the TSC, agenda and minutes, as well as copies of communications between meetings so they may check the next report against them. All documentation should be considered confidential. After the trial is reported, the TSC members should destroy all interim reports. The trials office will keep a central record of all minutes, reports and correspondence by the TSC.
**8. DECISION MAKING**	
What decisions will be open to the TSC	Based on recommendations from the DMC, possible decisions include:-<br>• No action needed; trial continues as planned.<br>• Early stopping due, for example, to clear harm of a treatment or external evidence (this should generally involve a recommendation from the DMC to unblind the TSC to this data).<br>• Modifying target recruitment, or pre-analysis follow-up, based on any change to the assumptions underlying the original trial sample size calculation (but not on any emerging differences).<br>• Sanctioning and/or proposing protocol changes.<br><br>Based on other factors, possible decisions include the decisions above and:<br>• Censuring centres for poor data quality.<br>• Approving proposed protocol amendments or new trial sub-studies.<br>• Approving requests for early release of (subsets of) data.<br>• Approving presentation of results during the trial or soon after closure.<br>• Approval of new centres or strategies to improve recruitment or follow-up.
The role of formal statistical methods	The TSC will be asked to comment on and approve trial statistical analysis plan (SAP) before database lock.
How decisions or recommendations will be reached within the TSC	Every effort should be made to achieve consensus. The role of the Chair is to summarise discussions and encourage consensus; therefore, it is usually best for the Chair to give their own opinion last.<br>It is important that the implications (e.g., ethical, statistical, practical, financial) for the trial be considered before any decision is made.
When the TSC is quorate for decision-making	At least two independent members of the TSC should be present including the Chair, along with at last one PI
Any specific issues relating to the trial design that might influence the proceedings, eg cluster trials, equivalence trials, multi-arm trials	This is a 2-arm trial, occurring in the UK (Leicester) and Canada (Montreal, Edmonton). It is likely that the TSC will need to consider issues specific to UK or Canadian sites
**9. REPORTING**	
To whom will the TSC report their recommendations/decisions, and in	Reports will be returned to the TMG who will be responsible for implementing any actions resulting. The TSC may also provide feedback to

RESET FOR REMISSION TRIAL TSC CHARTER	
what form	the DMC and, where appropriate, to the Sponsors or Funders.
Whether minutes of the meeting be made and, if so, by whom and where they will be kept	Notes of key points and actions will be made by a member of the trial management team. This will include details of whether potential competing interests have changed for any attendees since the previous meeting. The draft minutes will be initially circulated for comment to those TSC members who were present at the meeting. The TSC Chair will sign off the final version of minutes or notes. The final version will then be sent to all members, the sponsor, the funder and the trial master file.
What will be done if there is disagreement between the TSC and other trial committees	The TSC is the oversight body for the trial. However, the TSC should have good reason before deciding not to accept requests from the TMG and recommendations from the DMC. If there are serious problems or concerns with the TSC decision following a DMC recommendation, a joint meeting of the TSC and DMC should be held. The information to be shown would depend upon the action proposed and each committee's concerns. The meeting would be Chaired by a senior member of the trial management staff or an external expert who is not directly involved with the trial.
**10. AFTER THE TRIAL**	
Publication of results	The TSC will oversee the timely analysis, writing up and publication of the main trial results. The independent members of the TSC will have the opportunity to read and comment on the proposed main publications of trial data prior to submission and abstracts and presentations during the trial. This review may be concurrent to that of the trial investigators and DMC.
The information about the TSC that will be included in published trial reports	TSC members will be named and their affiliations listed in the main report, unless they explicitly request otherwise.
Any constraints on TSC members divulging information about their deliberations after the trial has been published	The TSC may discuss issues from their involvement in the trial 12 months after the primary trial results have been published, or sooner if permission is agreed with the other trial committees and trials office. Similarly, the TSC will decide when it is appropriate for TMG and DMC members to trade in related stocks.

RESET FOR REMISSION

TSC Charter for R4R | Author: Kaberi Dasgupta and Thomas Yates

Date: 13/01/2021 | Version: 1.0

Figure 1: Trial Design

Pre-screening

Consent and **baseline assessments (VISITS 0, 1 & 2)***
(UK n = 40, Canada n = 40)

Randomisation

Standard care UK n =20, Canada n = 20

Low energy diet incorporating phased meal replacement plan combined with exercise training UK n=20, Canada n = 20

Dietary Support Weeks 1 through 12
Initial meeting with study dietitian for intervention planning in **week 0** with core follow-on support provided in **weeks 1, 2, 4, 8**

Total meal replacement plan
Weeks 1 and 2
Day 1 is first day of plan

Exercise Support Weeks 3 through 12
Structured exercise sessions three times per week, at least two of which directly supervised

Low Energy Diet with partial meal replacement plan
Weeks 3-12

Structured aerobic and resistance training
Weeks 3-12

Clinical review (Baseline, **week 1, 2, 4, 8**)
*Blood pressure, glucose and weight
*Antihyperglycemics and antihypertensives withdrawn at week 0; reintroduced/rewithdrawn as clinically needed

Week 12 intermediate **outcome assessments (VISIT 3)***
*Baseline, week 12 and week 24 visits include: HbA1c, accelerometer monitoring, resting energy expenditure, DXA scan, weight, neck and waist circumference, urine sample, blood pressure, fasting glucose, lipids, BMI, maximal exercise test, short physical performance battery, food diary, questionnaires; only baseline and final assessments include MRI

Dietary Support weeks 12-24
Core dietary support sessions
Week 12, 14, 16, 18 & 21

Exercise Support weeks 12-24
Core exercise support sessions
Week 12, 14, 16, 18 & 21

Clinical review weeks 12-24
Week 12, 14, 18

Tapering of meal replacement plan and 'relapse management'
Weeks 13-24

Tapered exercise supervision and 'relapse management'
Weeks 13-24

Week 24 final **outcome assessments (VISITS 4 & 5)** *
Exit consultation with dietitian, exercise specialist, study physician(s)

Control group offered the 12 week low energy diet and meal replacement plan, followed by a 4-week food reintroduction period

Process evaluation with intervention

8

Annex 1: Agreement and competing interests form for independent members

RESET FOR REMISSION TRIAL: Agreement to join the Trial Steering Committee as an independent member and disclosure of potential competing interests

Please complete the following document and return to the Trials Office

(Please initial box to agree)

☐ I have read and understood the TSC Charter version 1.0, dated (will insert date pending REB approval)

☐ I agree to join the Trial Steering Committee for this trial as an independent member

☐ I agree to treat all sensitive trial data and discussions confidentially

The avoidance of any perception that independent members of a TSC may be biased in some fashion is important for the credibility of the decisions made by the TSC and for the integrity of the trial.

Potential competing interests should be disclosed via the trials office. In many cases simple disclosure up front should be sufficient. Otherwise, the (potential) independent TSC member should remove the conflict or stop participating in the TSC. **Table 1** lists potential competing interests.

☐ **No,** I have no potential competing interests to declare
☐ **Yes,** I have potential competing interests to declare (please detail below)

Please provide details of any potential competing interests:

____

____

____

Name: ____________________________

Signed: ___________________________ Date: ______________

Table 1: Potential competing interests for independent members

- Stock ownership in any commercial companies involved
- Stock transaction in any commercial company involved (if previously holding stock)
- Consulting arrangements with the Sponsor/Funder
- Ongoing advisory role to a company providing products to the trial
- Frequent speaking engagements on behalf of the intervention
- Career tied up in a product or technique assessed by trial
- Hands-on participation in the trial
- Involvement in the running of the trial
- Emotional involvement in the trial
- Intellectual conflict e.g., strong prior belief in the trial's experimental arm
- Involvement in regulatory issues relevant to the trial procedures
- Investment (financial or intellectual) or career tied up in competing products

• Involvement in the writing up of the main trial results in the form of authorship

Annex 2: Agreement and competing interests form for non-independent members

RESET FOR REMISSION TRIAL: Agreement to join the Trial Steering Committee as a non-independent member and disclosure of potential competing interests

Please complete the following document and return to the Trials Office.

(Please initial box to agree)

☐ I have read and understood the TSC Charter version 1.0, dated 24th April 2013

☐ I agree to join the Trial Steering Committee for this trial as a non-independent member

☐ I agree to treat all sensitive trial data and discussions confidentially

The avoidance of any perception that members of a TSC may be biased in some undisclosed fashion is important for the credibility of the decisions made by the TSC and for the integrity of the trial.

Possible competing interests should be disclosed via the trials office. In many cases simple disclosure up front should be sufficient. Otherwise, the (potential) independent TSC member should remove the conflict or stop participating in the TSC. **Table 1** lists potential competing interests.

☐ **No,** I have no competing interests to declare other than involvement in the trial

☐ **Yes,** I have competing interests to declare (please detail below)

Please provide details of any competing interests:

____

____

____

Name: ____________________________

Signed: ___________________________ Date: ______________

Table 1: Potential competing interests for non-independent members

• Stock ownership in any commercial companies involved<br>• Stock transaction in any commercial company involved (if previously holding stock)<br>• Consulting arrangements with the Sponsor/Funder<br>• Ongoing advisory role to a company providing drugs to the trial<br>• Frequent speaking engagements on behalf of the intervention<br>• Intellectual conflict e.g., strong prior belief in the trial's experimental arm<br>• Involvement in regulatory issues relevant to the trial procedures

- Investment (financial or intellectual) in competing products

Annex 3: Agreement and confidentiality agreement for observers

RESET FOR REMISSION TRIAL: Agreement to attend the Trial Steering Committee and treat all information confidentially

Please complete the following document and return to the Trials Office.

(Please initial box to agree)

☐	I have received a copy of the TSC Charter version 1.0 dated 24th April 2013
☐	I agree to attend the Trial Steering Committee meeting on ___/____/______
☐	I agree to treat as confidential any sensitive information gained during this meeting unless explicitly permitted

Name: ____________________________

Signed: ___________________________ Date: ______________

Dasgupta K, *et al. BMJ Open* 2022; 12:e063888. doi: 10.1136/bmjopen-2022-063888

Annex 4: Document History

Version	Date	Who	Comments
1.0	24 April 2013	DM	First draft
