# Supplementary material for: Remission of type 2 diabetes and improved diastolic function by combining structured exercise with meal replacement and food reintroduction among young adults: the RESET for REMISSION randomised controlled trial protocol
Source: BMJ Open. 2022 Sep 21;12(9):e063888. doi: 10.1136/bmjopen-2022-063888 (PMC9494595; doi:10.1136/bmjopen-2022-063888)
Supplement: Author's manuscript [file bmjopen-2022-063888.draft_revisions.pdf]

BMJ Open is committed to open peer review. As part of this commitment we make the peer review history of every article we publish publicly available.

When an article is published we post the peer reviewers' comments and the authors' responses online. We also post the versions of the paper that were used during peer review. These are the versions that the peer review comments apply to.

The versions of the paper that follow are the versions that were submitted during the peer review process. They are not the versions of record or the final published versions. They should not be cited or distributed as the published version of this manuscript.

BMJ Open is an open access journal and the full, final, typeset and author-corrected version of record of the manuscript is available on our site with no access controls, subscription charges or pay-per-view fees (http://bmjopen.bmj.com).

If you have any questions on BMJ Open's open peer review process please email info.bmjopen@bmj.com

BMJ Open

Remission of Type 2 Diabetes and Improved Diastolic Function by Combining Structured Exercise with Meal Replacement and Food Reintroduction among young adults: The RESET FOR REMISSION Randomized Controlled Trial Protocol

Journal:	*BMJ Open*
Manuscript ID	bmjopen-2022-063888
Article Type:	Protocol
Date Submitted by the Author:	21-Apr-2022

Complete List of Authors:	Dasgupta, Kaberi; Research Institute of the McGill University Health Centre, Department of Medicine, Faculty of Medicine<br>Boulé , Normand ; University of Alberta<br>Henson, Joseph; NIHR Leicester Biomedical Research Centre, Diabetes Research Centre<br>Chevalier, Stéphanie; McGill University, School of Human Nutrition<br>Redman, Emma; Leicester General Hospital<br>Chan, Deborah; Research Institute of the McGill University Health Centre<br>McCarthy, Matthew; Leicester General Hospital<br>Champagne, Julia; Research Institute of the McGill University Health Centre<br>Arsenyadis, Frank; Leicester General Hospital<br>Rees, Jordan; University of Alberta Faculty of Kinesiology Sport and Recreation<br>Da Costa, Deborah; Research Institute of the McGill University Health Centre<br>Gregg, Edward; Imperial College London<br>Yeung, Roseanne; University of Alberta<br>Hadjiconstantinou, Michelle; University of Leicester<br>Dattani, Abhishek; University of Leicester Department of Cardiovascular Sciences<br>Friedrich, Matthias G.; Courtois Cardiovascular Signature Centre, McGill University Health Centre<br>Khunti, Kamlesh; University of Leicester, Diabetes Research Centre<br>Rahme, Elham; McGill University Health Centre, Division of Clinical Epidemiology<br>Fortier, Isabel; Research Institute of the McGill University Health Centre<br>Prado, CM; University of Alberta<br>Sherman, Mark; Leicester Real World Evidence Unit, University of Leicester<br>Thompson, Richard ; University of Alberta, Department of Biomedical Engineering<br>Davies, Melanie; University of Leicester, Diabetes Research Centre<br>McCann, Gerry; University of Leicester Department of Cardiovascular Sciences,<br>Yates, Thomas; University of Leicester Diabetes Research Centre

Keywords:	DIABETES & ENDOCRINOLOGY, CARDIOLOGY, NUTRITION & DIETETICS

SCHOLARONE™
Manuscripts

Title: Remission of Type 2 Diabetes and Improved Diastolic Function by Combining Structured Exercise with Meal Replacement and Food Reintroduction among young adults: The RESET FOR REMISSION Randomized Controlled Trial Protocol

Authors:

Kaberi Dasgupta*[1], Normand Boulé[2], Joseph Henson[3], Stéphanie Chevalier[4], Emma Redman[3], Deborah Chan[1], Matthew McCarthy[3], Julia Champagne[1], Frank Arsenyadis[3], Jordan Rees[2], Deborah Da Costa[1], Edward Gregg[5], Roseanne O. Yeung[6], Michelle Hadjiconstantinou[3], Abhishek Dattani[7], Matthias G. Friedrich[8], Kamlesh Khunti[9], Elham Rahme[1], Isabel Fortier[1], Carla Prado[2], Mark Sherman[9], Richard Thompson[10], Melanie Davies[3], Gerry P. McCann[7], Thomas Yates*[3]

*Dr Dasgupta and Dr Yates are the Co-Lead investigators.

<u>Corresponding author</u>:

Kaberi Dasgupta, Professor of Medicine, McGill University

Centre for Outcomes Research and Evaluation, Research Institute of the McGill University Health Centre, 5252 Maisonneuve West Boulevard, Montreal, Quebec, Canada H4A 3S5

kaberi.dasgupta@mcgill.ca

[1] Centre for Outcomes Research and Evaluation, Research Institute of the McGill University Health Centre, 5252 Maisonneuve West Boulevard, Montreal, Quebec, Canada H4A 3S5; Department of Medicine, McGill University

[2] Faculty of Kinesiology, Sport, and Recreation 1-052 Li Ka Shing Centre for Health Research Innovation, University of Alberta, Edmonton, Alberta, Canada T6G 2H9

For peer review only

[3] Diabetes Research Centre, University of Leicester, Leicester General Hospital, Gwendolen Road, Leicester, United Kingdom LE5 4PW; NIHR Leicester Biomedical Research Centre, University Hospitals of Leicester NHS Trust and University of Leicester, Leicester, United Kingdom

[4]School of Human Nutrition, Macdonald Campus of McGill University, 21111 Lakeshore Road, Sainte-Anne-de-Bellevue, Quebec, Canada H9X 3V9

[5] School of Public Health, Imperial College London, Medical School Building, St Mary's Hospital, Norfolk Place, London, United Kingdom W2 1PG

[6] Division of Endocrinology & Metabolism, Department of Medicine, 9114- Clinical Sciences Building 11350 - 83 Avenue, Edmonton, Alberta, Canada T6G 2G3

[7]Department of Cardiovascular Sciences University of Leicester and the NIHR Leicester Biomedical Research Centre, Glenfield Hospital NHS Trust, Groby Rd, Leicester, United Kingdom LE3 9QP

[8] Courtois Cardiovascular Signature Centre, McGill University Health Centre, 1001 Decarie Boulevard, Montreal, Quebec, Canada H4A 3J1; Departments of Medicine and Diagnostic Radiology, McGill University

[9] Leicester Real World Evidence Unit, University of Leicester, Leicester General Hospital, Gwendolen Road, Leicester, United Kingdom LE5 4PW

[10] Department of Biomedical Engineering, 1098 Research Transition Facility, University of Alberta, Edmonton, Alberta, Canada T6G 2V2

Word Count: 3995 words

For peer review only - http://bmjopen.bmj.com/site/about/guidelines.xhtml

ABSTRACT

Introduction

Type 2 diabetes (T2DM) onset before 40 years of age has a magnified lifetime risk of cardiovascular disease. Diastolic dysfunction is its earliest cardiac manifestation. . Low energy diets incorporating meal replacement products can induce diabetes remission, but do not lead to improved diastolic function, unlike supervised exercise interventions. We are examining the impact of a combined low energy diet and supervised exercise intervention on T2DM remission, with peak early diastolic strain rate, a sensitive MRI-based measure, as a key secondary outcome.

Methods and Analysis

This prospective, randomized, two-arm, open-label, blinded-endpoint efficacy trial is being conducted in Montreal, Edmonton, and Leicester. We are enrolling 100 persons 18 to 40 years of age within 6 years' T2DM diagnosis, not on insulin therapy, and with obesity. During the intensive phase (12 weeks), active intervention participants adopt an 800 to 900 kcal/day low energy diet combining meal replacement products with some food, and receive supervised exercise training (aerobic and resistance), three times weekly. The maintenance phase (12 weeks) focuses on sustaining any weight loss and exercise practices achieved during the intensive phase; products and exercise supervision are tapered but reinstituted, as applicable, with weight regain and/or exercise reduction. The control arm receives standard care. The primary outcome is T2DM remission, (hemoglobin HbA1c of less than 6.5% at 24 weeks, without use of glucose lowering medications during maintenance.) Analyses will be by intention-to-treat with stratified Fisher's exact test statistics.

Ethics and Dissemination The trial is approved in Leicester (East Midlands – Nottingham Research Ethics Committee [21/EM/0026]), Montreal (McGill University Health Centre Research Ethics Board [RESET for remission / 2021-7148]), and Edmonton (University of

Alberta Health Research Ethics Board (Pro00101088). Findings will be shared widely (publications, presentations, press releases, social media platforms) and will inform an effectiveness trial.

Trial registration: ISRCTN15487120 https://doi.org/10.1186/ISRCTN15487120. Registered in accordance with World Health Organisation Trial Registration Data Set (version 1.3.1). Date assigned June 22, 2021.

Funding: Medical Research Council (grant number MR/T031816/1) of the United Kingdom; Canadian Institutes of Health Research (grant number UCD-170584; Institutes of Musculoskeletal Research and of Nutrition, Metabolism & Diabetes); John R McConnell Foundation (grant number n/a)

Key words: diabetes reversal, early-onset diabetes, heart failure, intensive lifestyle intervention, health behaviour change, obesity, weight loss, fitness, type 2 diabetes

For peer review only

Strengths and Limitations

- The active intervention combines a low energy meal replacement-based diabetes remission strategy with a structured and supervised exercise intervention to assess impacts on remission, providing insights on potential synergies. Previous diabetes remission trials have not combined this type of exercise intervention with a low energy meal replacement-based approach.
- The combination of aerobic and resistance training is a key strength, with potential to optimize glycemic, metabolic, fitness, muscle mass, and cardiac outcomes.
- RESET cannot distinguish the impacts of the individual dietary and exercise components but will provide data that will inform decisions as to whether individual elements should be compared against a combined intervention.

For peer review only - http://bmjopen.bmj.com/site/about/guidelines.xhtml

- Our focus on young adults with type 2 diabetes is deliberate but recruitment will be challenging as they do not constitute the bulk of adults with type 2 diabetes and thus must be contacted through a wide-reaching recruitment strategy enriched by social media.
- Our trial moves beyond a strict focus on diabetes remission as defined by glycemia and antihyperglycemic medication use to a broader focus of interventions on MRI-based measures of cardiac health, fitness, and other important health metrics in diabetes.

BACKGROUND

The Da Qing type 2 diabetes (T2DM) prevention trial's 30-year follow-up demonstrated each year without T2DM translates into fewer cardiovascular disease (CVD) events (1). This is particularly salient in early-onset T2DM, which, when compared to later-onset T2DM (2) and even type 1 diabetes, (3) has a more aggressive phenotype (4). Younger adults represent at least 16% of T2DM internationally (5). Even temporary T2DM remission could lead to fewer diabetes-related complications during what are supposed to be the most productive years.

Health behaviour-based remission trials to date have focused on glucose-lowering below T2DM thresholds without medications, through reduced energy intake (6-9). Among adults 45 to 76 years old, the Look AHEAD trial implemented a 1200 to 1800 kcal diet including some meal replacements; remission was 11.5% at 1 year and 7.3% at 4 years (10). Among adults averaging 53 years of age and within 6 years of diagnosis, the DiRECT trial (11-13) evaluated an 800-kcal meal replacement diet for 3 to 5 months followed by a food-based weight maintenance diet. It demonstrated 46% remission at 1 year (12) and over 30% at 2 years (13). In younger adults within 3 years of diagnosis, the DIADEM-I trial achieved over 50% remission at 1 year (14). None consistently incorporated more intensive supervised

For peer review only - http://bmjopen.bmj.com/site/about/guidelines.xhtml

exercise nor included resistance training. Our RESET for REMISSION trial is addressing this knowledge gap, focusing on younger adults.

Supervised exercise interventions in T2DM improve glycaemia , despite minimal weight loss (15). Regular exercise attenuates diet-induced lean mass loss while enhancing fitness (16, 17); in Look AHEAD, increased fitness predicted T2DM remission (10). Exercise also impacts cardiac function (18, 19). Concentric remodelling typifies diabetic heart disease (20), with isolated diastolic dysfunction as its earliest manifestation (21), more prevalent in younger adults with T2DM compared to weight matched and lean controls (22, 23). In the DIASTOLIC trial (24), our UK team members compared low energy meal replacement diets against thrice weekly supervised aerobic exercise training over 12 weeks among young adults with T2DM. The diet arm achieved 70% remission without impact on diastolic function; the exercise arm had little remission impact but improved peak early diastolic strain rate (24), a sensitive MRI-based diastolic function measure.

Our RESET for REMISSION trial combines low energy diet and supervised exercise approaches in younger adults with T2DM. We hypothesize these will enhance heart health and have synergistic impacts on remission, with the addition of a resistance component to an aerobic exercise regimen, designed to enhance insulin-sensitizing effects (25).

Aims

Among adults 18 to 40 years of age with obesity, within 6 years of a T2DM diagnosis, and not on insulin therapy, we will quantify the remission efficacy of a 12-week low energy diet combined with supervised exercise, followed by a 12-week phase of weight and exercise maintenance. We define T2DM remission as a haemoglobin A1c (HbA1c) value less than 6.5% (48 mmol/mol) at 24 weeks, without use of antihyperglycemic medications during the prior 12 weeks, concordant with the international consensus definition. (6-9)

For peer review only - http://bmjopen.bmj.com/site/about/guidelines.xhtml

Through comprehensive cardiovascular magnetic resonance (CMR), we are also assessing circumferential peak early diastolic strain rate (Figure 2), a sensitive measure (22) with excellent test-retest reproducibility (26); end-diastolic mass to volume ratio, a marker of concentric left ventricular remodelling (27); and measures of aortic distensibility, a key determinant of concentric remodelling in T2DM (27). We will also ascertain impacts on a range of other measures (Table 1) of insulin resistance, fitness, adiposity, cardiorenal parameters, diet, physical activity, mood, and quality of life. We will evaluate participant perspectives (Appendix 1). Our results will guide the design of a longer term effectiveness trial.

Table 1. Outcomes

	Week 12	Week 24
**PRIMARY OUTCOME**		
Diabetes remission		X
**KEY SECONDARY OUTCOMES**		
Other remission, glycemic, and insulin resistance measures		
Diabetes remission	X	
Hemoglobin A1c, fasting glucose and insulin, Homeostatic Model Assessment for Insulin Resistance	X	X
Main CMR measures		
Left ventricular peak early diastolic strain rate (circumferential and longitudinal, MRI)		X
End-diastolic mass to volume ratio		X
Main fitness measure		
VO_2 peak	X	X
Main adiposity and lean mass measures		
Total tissue, fat, and lean mass (DXA)	X	X
Weight and BMI	X	X
Cardiometabolic indicators		
Hypertension remission, systolic and diastolic blood pressure, heart rate	X	X
Total cholesterol, HDL, LDL, triglycerides	X	X
**OTHER SECONDARY OUTCOMES**		
Renal function measures		
Creatinine and estimated glomerular filtration rate, Urine albumin to creatinine ratio	X	X

For peer review only - http://bmjopen.bmj.com/site/about/guidelines.xhtml
For peer review only

Hepatic function measures		
Aspartate aminotransferase, alanine aminotransferase, gamma glutamyl transferase, bilirubin	X	X
Depression, anxiety, and distress		
Hospital Anxiety and Depression Scale and Diabetes Distress Scale	X	X
Indirect calorimetry		
Resting metabolic rate	X	X
Additional cardiac and aortic MRI-based measures		
Longitudinal and circumferential measures of systolic strain, end systolic volume, ejection fraction, mean T1 time	X	X
Cross-sectional areas and distensibility of ascending and descending aortae	X	X
Additional measures of lean mass and adiposity		
Neck, hip, and waist circumference; Appendicular lean mass (DXA)	X	X
Visceral adipose tissue, pancreatic and liver fat percentages, subcutaneous adipose (MRI)		X
Dietary variables		
Total energy and macronutrients (protein, carbohydrates, lipids)	X	X
Sugars, starch, fibre; saturated, monounsaturated, polyunsaturated fats; cholesterol, alcohol	X	X
Accelerometer-based physical activity measures and sleep (daily average)		
Steps, overall acceleration, intensity gradient metric; Minutes for each of sedentary, light, and moderate to vigorous physical activity	X	X
Sleep time, duration of night, sleep efficiency (sleep time/duration of night)	X	X
Other exercise stress test measures		
VCO_2 peak, maximum gradient achieved	X	X
**Tertiary outcomes**		
Bone measures		
Total bone mineral density and bone mineral content (DXA)	X	X
Physical function		
Handgrip strength, Short Physical Performance Battery, Dyspnoea scale	X	X
Overall health state	X	X
EQ- 5D-5L score (EuroQuol group 5-dimensional 5-level questionnaire), WHO Disability Assessment Schedule 2.0	X	X

169

170 **Methods**

171 **Design**

172 Prospective, randomised, two-arm, open-label, blinded-endpoint (PROBE) efficacy trial

173 **Setting**

For peer review only - http://bmjopen.bmj.com/site/about/guidelines.xhtml

Leicester (Leicester Diabetes Centre), United Kingdom; Montreal (Research Institute of the McGill University Health Centre- Centre for Outcomes Research and Evaluation, Canadian coordinating site), Canada; Edmonton (Alberta Diabetes Institute – University of Alberta), Canada. The first participant was recruited on September 24, 2021; the trial will continue to June 1, 2024.

Eligibility Criteria

Adults with T2DM diagnosed less than 6 years previously and not on insulin therapy, who have a HbA1c value between 6 and 10% if taking other glucose-lowering medication or between 6.5 and 10% if not taking any glucose-lowering medications. Eligibility criteria are detailed in Table 2.

Table 2. Inclusion and exclusion criteria

**Inclusion criteria**	
Age	18 to 40 years, inclusive
Type 2 diabetes	Physician diagnosis more than three months and less than six years previously
Hemoglobin A1c	6.5% to 10%, inclusive if not taking glucose-lowering medication; 6 to 10% if taking glucose-lowering medication
Body mass index	30 kg/m^2 to 45 kg/m^2, inclusive if White or Indigenous[1]<br>27 kg/m^2 to 45 kg/m^2, inclusive if other background, including mixed
Weight stability	Weight changes of less than 5 kg over the prior 6 months
Walking ability	Able to walk without assists and to participate in structured exercise training requiring the lower limbs
Capacity	Able to understand written and spoken English and/or French<br>Able to provide informed consent
Willingness	Willing to be randomized and able to participate<br>Willing to attend supervised exercise sessions, if so randomized<br>Willing to adopt low energy diet, including abstinence from alcohol, if so randomized<br>Willing to self-monitor glucose and blood pressure at the required frequency, if randomized to the low energy diet plus supervised exercise arm
**Exclusion criteria**	
Other diabetes types	Type 1 diabetes<br>Gestational diabetes<br>Monogenic diabetes

For peer review only - http://bmjopen.bmj.com/site/about/guidelines.xhtml

Poorly controlled blood pressure	Resting systolic blood pressure greater than 150 mmHg or resting diastolic blood pressure greater than 90 mmHg diastolic
Weight loss interventions	Currently participating in a weight reduction program in addition to routine care<br>Previous bariatric surgery
Medications	Insulin therapy[2]<br>Use of licensed weight loss medications<br>Significant changes in glucose lowering medications in the prior 3 months, as judged by study physicians<br>Steroids by mouth or injection
Self-reported allergies to components of meal replacement products	Milk protein, sunflower oil, soy, wheat, eggs, peanuts, tree nuts, or sesame seeds
Dietary practices	Dietary practices that prohibit the use of meal replacement products
Pregnancy & Lactation	Pregnancy<br>Lactation<br>Planning to become pregnant in the next 8 months
Eating disorder	Self-reported or diagnosed
Substance abuse	Alcohol, drugs
Estimated glomerular filtration rate	Less than 60 $ml.min^{-1}$ per $1.73m^2$
Retinopathy	Receiving or requiring active treatment for retinopathy
Clinically manifest vascular disease	Myocardial infarction<br>Stroke<br>Peripheral vascular disease
Other cardiac disease	Heart failure<br>Atrial fibrillation<br>Pacemaker<br>Implantable cardioverter defibrillator (ICD)
Other conditions that could impact weight and/or safety	Active malignancy or other chronic disease
Run-in phase	Failure to complete at least 5 or requested 7 days of accelerometer wear<br>Failure to complete a food diary for three weekdays and one weekend day

[1] Term for the original peoples of North America and their descendants; includes First Nations, Inuit, and Métis peoples

[2] An exception is made for women who are on insulin therapy in case of pregnancy occurrence because of insulin's established safety profile in pregnancy, rather than because of

For peer review only - http://bmjopen.bmj.com/site/about/guidelines.xhtml

inability to control glycemia on oral agents alone. If these women are willing and able to use a reliable form of contraception, they may be enrolled.

RECRUITMENT

Recruitment is primarily through clinics within the collaborative networks of participating centres and publicity through social media and other forms of media.

Run-in period

Candidates wear an accelerometer for 7 consecutive days (wrist) and complete a 4-day food diary (three weekdays and one weekend day). Five 5 days of accelerometer wear and the 4-day food diary are required to move beyond the run-in period.

For peer review only

MEASUREMENTS

In-person evaluations occur before randomization, at intervention period midpoint (12 weeks +/- 1 week; no MRI study), and at intervention period end (24 weeks +/- 2 weeks) (Figure 1; Table 1).

Type 2 diabetes remission and related measures

The primary remission outcome requires a HbA1c below 6.5% at intervention period end, without glucose-lowering medication during the prior 12 weeks (6-9). Venous blood is sampled for HbA1c (high-performance liquid chromatography) measurement. Other related outcomes include HbA1c, insulin resistance (HOMA-IR), and midpoint remission.

MRI defined cardiac structure and function

Using the 3-Tesla platform (28) (Figure 2), we acquire cardiac cine images with retrospective electrocardiographic gating and an 18-channel phased-array cardiac receiver coil covering the left ventricle from base to apex (8 mm slice thickness, 2 mm gap with temporal resolution <50 ms and reconstructed to 30 phases). Using cmr42 software (CircleCardiovascular Imaging, Canada), we assess end-diastolic and end-systolic volumes, ejection fraction, myocardial mass, peak diastolic filling rate, systolic global longitudinal and circumferential

strain, (simplified) long axis strain, and circumferential and longitudinal peak early diastolic strain rates (Figure 3).

We quantify aortic stiffness, at the pulmonary artery bifurcation with simultaneous blood pressure recording, using Java Image Manipulation (Xinapse Software, Essex, UK). (27) We characterize myocardial tissue using a modified look-locker inversion-recovery sequence for a mid-level native T1 map. High native T1 time, and is a surrogate marker of diffuse interstitial fibrosis.

Adiposity and fat and lean mass

We measure weight (nearest 0.1 kg) and height, waist, hip and neck circumferences (nearest 0.1 cm) and use DXA (GE Lunar iDXA) for total fat and lean mass (Table 1). We acquire MRI images for visceral, subcutaneous, hepatic, and pancreatic fat using the chemical shift encoded (DIXON) approach. Taylor's twin cycle hypothesis (29) emphasizes hepatic fat accumulation in T2DM development, leading to insulin resistance along with pancreatic fat accumulation that impairs beta-cell function.

Optional MRI sequences

Time permitting, optional sequences include simultaneous PROton Density Fat Fraction Imaging and Water T1-Mapping with Low B1+ Sensitivity (PROFIT1) of the liver (fibrosis), thigh (skeletal muscle volume), and heart (intra-myocardial fat) (30). In Montreal, and time permitting at other sites, we perform oxygen sensitive CMR with hyperventilation and breath-holding, enabling ischaemia/microvascular dysfunction detection without contrast. (31) In Leicester, participants receive manganese contrast infusion, with repeated T1 mapping every 2.5 min for 30 min, to assess calcium handling. (32)

Resting metabolic rate, fitness, and strength

For peer review only - http://bmjopen.bmj.com/site/about/guidelines.xhtml

Following overnight fast, we conduct indirect calorimetry (ventilated hood system) for resting metabolic rate. We perform fixed speed treadmill exercise testing with increasing gradient (1% each minute) and a rolling average of 10 breaths for oxygen consumption and carbon dioxide production. The test continues to volitional exhaustion; 100% of age-predicted maximum heart rate (85% if using beta-blocker medication) plus Respiratory Exchange Ratio ≥ 1.15; or ECG changes or symptoms of concern. We assess peak oxygen consumption, heart rate, and blood pressure. The peak force output of handgrip is determined across three measures in each hand. We apply the Short Physical Performance Battery (33), WHODAS 2.0 questionnaire (34) and the Medical Research Council dyspnoea scale (mMRC dyspnoea scale) (35).

Diet, physical activity, and sleep

The four-day food diary is repeated at intermediary and final evaluations for daily intakes (Table 1). In addition to run-in, participants wear an accelerometer for a 7 to 14-day period proximate to the intermediary and final assessments. Accelerometer data is captured at 30 Hz (open source R program GGIR) (36) for daily averages of steps, overall acceleration, and intensity gradient metric, and total time at sedentary, light, and moderate to vigorous intensities. Overnight wear permits capture of sleep time, night duration, and their ratio (sleep efficiency).

Cardiometabolic profile

We assess seated blood pressure (automated sphygmomanometer, averaged systolic and diastolic 5 sequential measures). We sample blood for lipid profiles, creatinine, electrolytes, and liver function tests; and urine for the albumin to creatinine ratio. Among those without RAAS inhibition for albuminuria, we assess hypertension remission at intermediary and final assessments (blood pressure values of 130/80 mm Hg without use of antihypertensive agents during prior 12 weeks.)

For peer review only - http://bmjopen.bmj.com/site/about/guidelines.xhtml

Demographic factors, mental well-being, and quality of life

Participants complete questionnaires on sex and gender, date of birth, ethnicity, employment, income, education, and marital status; anxiety and depression (Hospital Anxiety and Depression scale (37)); diabetes-related distress (Diabetes Distress Scale (DDS) (38)); and quality of life (EQ-5D-5L (39)).

Data gathering for process evaluation

Through a qualitative descriptive approach, we will explore experiences of intervention arm participants (40). We will use maximum variation sampling (41) to capture those who did and did not achieve remission and variations in age, sex, ethnicity, and location. Our individual audio recorded and transcribed in-depth interviews (Appendix 1) are informed by the Normalisation Process Theory framework (42) and COM-B (Capability, Opportunity, Motivation - Behaviour) (43). Sampling will continue until saturation of themes is achieved.(44)

RANDOMIZATION AND ALLOCATION CONCEALMENT

We randomize participants individually, stratified by country and sex, in blocks of variable size. An independent statistician developed the randomization sequences uploaded into the Research Electronic Data Capture system. A researcher blinded to the sequence unveils group allocation through an auto-generated randomization button on REDCap.

EXPERIMENTAL GROUP

Full meal replacement diet for two weeks

Dietitian review: Participants meet with the dietitian to discuss the first two weeks of the intervention which include only *Optifast* products (*Nestlé*), totalling 800 to 900 kcal/day (30% protein, 50% carbohydrate, 20% fat). To minimize constipation, participants drink at least 2 litres of calorie-free fluid each day, and receive a fibre-based laxative, to use as

For peer review only - http://bmjopen.bmj.com/site/about/guidelines.xhtml

needed. They receive a digital body weighing scale and record weight weekly. They meet with the dietitian again in weeks 1 and 2 (virtually or in-person, as preferred).

Physician review: The study physician meets with the intervention arm participant to discuss antihyperglycemic and antihypertensive medication withdrawal, (45) given immediate lowering of glucose values and blood pressure with the dietary intervention. Glucagon-like peptide 1 receptors are stopped one week prior to the low energy diet. All other antihyperglycemic medications are stopped the day that the diet is started. Except for angiotensin converting enzyme inhibitors or angiotensin receptor blockers prescribed for albuminuria (i.e., RAAS blockade), participants stop antihypertensive medications on the day that the diet is initiated.

At weeks 1 and 2 follow-up (virtual or in-person), the physician reviews participant-recorded glucose and blood pressure data. Participants perform capillary blood glucose testing daily before breakfast and, on one day prior to physician follow-up, a 7-point glucose profile (measures before and two hours after food or meal replacement consumption and at bedtime). Antihyperglycemic medications are not considered for reintroduction unless fasting morning capillary glucose is frequently 10 mmol/l or higher. Using the home blood pressure monitor provided, participants measure seated blood pressure each morning, after a 5-minute rest period (second of two sequential measures). Antihypertensive agents are not be reintroduced unless systolic blood pressure values are generally 165 mmHg or higher, as in the DiRECT trial (46). RAAS blockade may be interrupted with symptomatic hypotension.

Participants who may become pregnant are required to use reliable contraception. This is discussed, with referral as needed.

Partial meal replacement low energy diet and supervised exercise (weeks 3 to 12 inclusive)

For peer review only - http://bmjopen.bmj.com/site/about/guidelines.xhtml

Exercise physiologist: Starting at week 3, an exercise physiologist supervises two weekly sessions with aerobic and resistance components, at a designated facility. A third session is aerobic only, on site or elsewhere, as preferred, with self-monitoring (heart rate monitor and/or physical activity tracker provided). Participants increase exercise intensity and duration over time, with a goal of 60-minute sessions.

Aerobic exercise on-site includes brisk treadmill walking (60-80% of the maximum stress test heart rate). There is a high intensity interval walking option (47), to maximize improvements in fitness, insulin sensitivity (48) and lean mass preservation (49, 50). With walking challenges (e.g., plantar fasciitis), cycle ergometer may be offered.

Resistance training with machines or free weights includes one to three sets of four upper body exercises (bench press, seated row, shoulder press, and pull down), three leg exercises (leg press, extension, flexion), and exercises targeting stability, function, and posture, with a goal of 8-12 repetitions per set. At 12 repetitions with good technique, the prescribed resistance is increased. Participants are instructed in resistance band use, in preparation for the maintenance phase.

Dietary component: Participants shift to partial meal replacement at week 3, with 800-900 kcal daily on non-exercise days and an additional 150-200 kcal from meal replacement products on exercise days. In addition to meal replacement products, the dietary 'real food' components are the equivalent of 125 ml of milk (or non-dairy alternative), 90 g of protein from foods (meat, fish, egg or alternative), 1 portion of fruit and 2 portions of non-starchy vegetables each day. Because the variety of *Optifast* products are more limited in Canada than in the UK, *ProtiDiet* (soups, oatmeal, and bars) are incorporated. Participants meet with the dietitian at weeks 4 and 8. The low energy diet is continued until week 12 and/or ideal

For peer review only - http://bmjopen.bmj.com/site/about/guidelines.xhtml

weight (corresponding to BMI of 25 kg/m^2 for white participants and those of Indigenous origin and 23 kg/m^2 in participants of non-white or mixed background.)

Physician review: Participants meet with the study physician at weeks 4 and 8. The systolic blood pressure threshold to consider antihypertensive reintroduction at this stage is 140 mmHg, as in DiRECT (46).

Maintenance phase of intervention

Dietary component: By week 12's end, the dietitian and participant create an individualized plan for tapering meal replacement products, meeting again at weeks 14, 16, 18, and 21. The goal is to maintain any weight loss achieved in weeks 1 through 12. Meal replacement product tapering may be slowed, and product intake may even be increased again, in the event of weight regain.

Exercise component: Using monitoring tools, participants aim for 150 minutes of moderate to vigorous physical activity weekly, including three 30-minute dedicated sessions at 60 to 80% of the maximum heart rate achieved during the intermediary exercise stress test. Participants may use the study exercise facility or engage in home-based or community based exercise such as brisk walking and resistance training facilitated by bands. Participants have virtual or telephone contact with exercise physiologists at weeks 14, 16, 18 and 21. Those with less than 60 minutes of planned aerobic exercise and/or less than one resistance training session per week are asked to return for additional supervised sessions.

Physician monitoring: The study physician meets with participants at weeks 14 and 18. The thresholds for medication adjustment are as described previously. The HbA1c value at the intermediary evaluation may also be used to guide treatment.

For peer review only

376 **End of intervention**

377 Following final evaluations, intervention team members each meet with the participant to
378 discuss strategies to maintain weight loss and exercise patterns. The study physician makes
379 recommendations for medication management to the participant's usual treating physician, in
380 conformity with national diabetes management guidelines and the participant's medication
381 reimbursement plan regulations.

382

383 **CONTROL GROUP**

384 The control group receives care from their usual physician, in accordance with National
385 Institute for Health and Care Excellence (NICE) (51) and Diabetes Canada (52) guidelines.
386 Following final evaluations, control arm participants will be offered a 12-week low energy
387 diet intervention followed by a 4-week food reintroduction period.

388

389 **MONITORING FOR ADVERSE EVENTS**

390 Intervention team members enquire about adverse events at each visit. Participants are asked
391 to contact trial staff between visits to report any concerns. Serious adverse events will be
392 reported within 7 days to all Research Ethics Boards and the Data Monitoring Committee
393 which may seek a formal adjudication of relatedness by physicians blinded to the treatment
394 arm and who have not interacted with the study participants in question.

395

396 More common adverse events in low energy diet trials (12, 53) include constipation (18 to
397 47%), headache (8%), dizziness (4 to 32%), fatigue (11 to 25%), and thirst (6%).
398 Constipation responds to fibre-containing laxatives and fluid intake, and other symptoms
399 resolve over time. In one high intensity aerobic training trial, (54) 16% experienced fracture
400 or lower extremity muscle cramping and/or muscle, ligament, or tendon strain; 5.2%
401 experienced chest pain, difficulty breathing, dizziness, or loss of consciousness. In a trial with
402 a combined resistance and aerobic exercise arm (16), 12.5% had one of shoulder injury, left

For peer review only - http://bmjopen.bmj.com/site/about/guidelines.xhtml

knee pain, spinal stenosis exacerbation, or hip pain; one participant experienced atrial fibrillation.

WITHDRAWAL

Interventions and evaluations are halted in the event of intolerance to the study intervention or an adverse event that precludes participation, prolonged or serious hospital admission, the development of a serious co-morbid condition such as active malignancy, or pregnancy. We will document reasons for participant-initiated withdrawal, if specified.

COVID-19 RESTRICTIONS AND ADAPTATIONS

If COVID-19 restrictions are resumed, exercise physiologist led sessions using video conferencing may be used to replace some in-person supervised exercise sessions, using heart rate monitors, self-monitoring devices, and resistance bands. Any remaining in-person intervention visits or outcome measurement sessions will be undertaken using personal protective equipment following clinical guidelines that are current at the time of practice.

SAMPLE SIZE

In the DIRECT trial, remission was 46% at one year (vs. 4% in control arm; odds ratio 19.7) (12). Even higher remission was observed over the shorter term in other studies (55). We initially allowed for a minimum 45% remission at 24 weeks in the intervention group and up to a 5% remission rate in the control group. Using a Fisher exact test with 90% power, a significance of 0.05, and a 1:1 ratio between intervention and control groups, we estimated needing 56 individuals to complete the trial (control = 28, intervention n = 28). Allowing for up to 30% drop-out, we planned to recruit 80 individuals in total (40 UK, 40 Canada) in our successful application for funding to the Medical Research Council and the Canadian Institutes of Health Research.

For peer review only

We subsequently obtained additional funding from the Canadian JR McConnell Foundation, and increased recruitment goals in Canada from 40 to 60 participants (40 UK, 60 Canada). With an overall sample size of 100 (40 UK, 60 Canada), if 70 (70%) complete the trial, we will have 90% power to detect a minimum 35% remission at 24 weeks in the intervention group and up to a 5% remission rate in the control group.

Retention of at least 35 participants in each arm (70 total) will provide over 80% power to detect a difference in mean peak early diastolic strain rate of 0.10 s^{-1} difference, equivalent to the exercise effect in the DIASTOLIC trial, (24) assuming that the common standard deviation is 0.144 as in the DIASTOLIC trial and using a two group t-test with a 5% two-sided significance level.

STATISTICAL ANALYSES

We will apply intention-to-treat principles for the primary outcome, assuming that remission of diabetes did not occur if information is not available. Since we anticipate a small number with remission in the control arm, we will use stratified Fisher's exact test statistics (i.e., stratification by country and by sex). If numbers permit, logistic odds ratios with 95% confidence intervals (exact method) will also be performed to further quantify the efficacy of intervention over the control group, after adjusting for factors used to stratify the randomization. Subgroup analyses for the primary outcome will be performed by country, sex, and the degree of weight loss (<5%, 5 to 10%, 10 to 15%, $\geq$15% as examined in the DiRECT trial (12), collapsing categories as needed, and by exercise adherence (<50%, >50% of supervised sessions attended). In a per-protocol analysis, we will restrict the intervention group to those who achieved at least 10% weight loss and adhered to at least two thirds of prescribed exercise sessions.

For peer review only - http://bmjopen.bmj.com/site/about/guidelines.xhtml

Secondary outcomes will be analysed using generalised linear modelling with an appropriate distribution and link function, adjusted for baseline value and factors used to stratify the randomization (country and sex), with data reported as mean intervention effect (change in intervention minus change in control) with 95% confidence intervals. Missing baseline data will be handled using the indicator method, so cases are not dropped where follow-up data is present without baseline.

PROCESS EVALUATION ANALYSES

At least two trained team members will independently code transcripts for themes (56), continually refining existing codes and identifying new ones. We will use Dedoose Version 7.0.23 and NVivo analysis software to facilitate data coding/organization.

DATA MANAGEMENT

Data management is facilitated by institutional REDCap systems, with access controlled through Active Directory Technology. Data and images shared across participating centres use secure data transfer systems with all identifiable information removed. Within REDCap, we have derived plausible ranges for all outcome measures; values outside these ranges are automatically flagged and verified for accuracy.

DISCUSSION

Low energy diets incorporating meal replacements can achieve T2DM remission through weight loss, (12-14) but may reduce lean mass (16, 24). Exercise can preserve lean mass (16, 24) and directly lower insulin resistance (25, 48, 57-59). Combined aerobic and resistance training yields additive glucose-lowering benefits (25). Regular exercise reduces CVD events (60-63) and improves MRI-based measures of heart health (24). RESET for REMISSION will quantify remission of T2DM in young adults achieved through a combined low energy diet and supervised aerobic and resistance training intervention and will also ascertain

For peer review only - http://bmjopen.bmj.com/site/about/guidelines.xhtml

changes in diastolic function, lean mass, and other factors that may impact long term outcomes.

There is equipoise with respect to the combined effects of the dietary and exercise intervention on weight change. Some studies suggest appetite stimulation with exercise (64, 65), while others suggest better appetite regulation (66). Intervention arm participants must acclimate to a low energy diet and travel to an exercise centre for supervision. Despite low energy diet challenges, DiRECT trial active arm participants had improved quality-of-life measures compared to control arm participants and regular exercise has demonstrated favourable effects on mood (67, 68).

While young persons with T2DM stand to gain the most from remission, (2), their daily life demands may render adherence challenging. We have endeavoured to mitigate this by integrating virtual communication tools for monitoring and follow-up where possible. Recruitment itself may be challenging in these younger individuals, as they remain fewer in number than middle-aged and older individuals with T2DM (5). We are therefore collaborating with a wide network of practices and drawing on other diabetes research cohorts in which participants have provided permission to be contacted for trials. The international diabetes remission panel underscored the importance of evaluating the impacts of nonglycemic measures during remission. (6-9) RESET evaluates not only lipid profiles and MRI measures of pancreatic, hepatic, and visceral fat but also sensitive MRI-based indicators of heart health for a comprehensive picture of intervention impacts. Equally importantly, we seek to understand impacts on quality of life, diabetes distress, and mood.

The Canada-UK collaboration that underpins our trial will allow a wider sharing of knowledge and perspectives than in a single-country study, challenging all to learn from best practices in each jurisdiction. The DiRECT trial led to an ongoing 5000 person pilot by the

For peer review only - http://bmjopen.bmj.com/site/about/guidelines.xhtml

National Health Service in the UK for prescribing meal replacements as part of routine T2DM management (51). The visibility of the current trial and the Canada-UK partnership could be an important step towards considering implementing such an intervention with integration of exercise in Canadian health care jurisdictions. Indeed, a future trial may seek partners beyond these two countries, to move from a demonstration of efficacy to one of effectiveness across a wide range of settings, and a plan for implementation. This is a critical journey not only to meet the glycemic definitions of diabetes remission but also to establish its potential myocardial and vascular effects.

SPONSORS

The study is sponsored by the University of Leicester in the UK (RGOsponsor@leicester.ac.uk) and the Research Institute of the McGill University Health Centre in Canada (cinzia.raponi@muhc.mcgill.ca) who will ensure the study is conducted according to Good Clinical Practice and that all contractual, governance, ethics and regulatory processes are followed.

TRIAL STEERING COMMITTEE

This committee is responsible for the overall oversight of the trial and will review and approve protocol amendments, any sub-study proposals, review recruitment rates, protocol adherence, retention, compliance, safety issues, planned analyses and reports, and act on recommendations of the Data Monitoring Committee. The Trial Steering Committee includes an independent Chair (Jason Gill, University of Glasgow), an independent clinician (Alice Cheng, University of Toronto), the Nominated Principal Investigators (Kaberi Dasgupta, McGill University & Thomas Yates, University of Leicester), the key site investigator in Edmonton (Normand Boulé, University of Alberta), and the trial statistician (Elham Rahme, McGill University). The Trial Steering Committee's operations are described in its charter (Appendix 2).

For peer review only - http://bmjopen.bmj.com/site/about/guidelines.xhtml

DATA MONITORING COMMITTEE

The Data Monitoring Committee is responsible for the interests and safety of the participants and its main role will be to make advisory recommendations to the Trial Steering Committee. It includes an independent clinician (Andrew Farmer, Nuffield Department of Primary Care Health Sciences, University of Oxford), an independent chair (Janusz Kaczorowski, University of Montreal) and independent statistician (Stephen Sharp, University of Cambridge). The Data Monitoring committee is independent from the sponsors and operates in conformity with a charter (Appendix 3).

PUBLIC AND PATIENT PARTNER INVOLVEMENT AND ENGAGEMENT GROUP

This group is providing feedback on recruitment, evaluation, and retention strategies and alternatives. They will be involved in interpretation of findings and knowledge dissemination efforts, including manuscript co-authorship, presentations, and interviews. The partners in Canada include Josette Spencer, Mark Marcinkiewicz, and Sylvie Lauzon, who is Executive Director of Diabetes Quebec. Patricia Kearns is a patient partner trainer who assisted with training of the patient partners. Alastair Masters is a patient partner based in the UK.

DISSEMINATION

We will publish the results of this trial in peer-reviewed journals and disseminate further in the scientific community through educational and conference presentations and social media targeting health professionals and the scientific community. We will share findings with the general public through press releases, media interviews, and social media forums. The trial patient partners and participants will be invited to assist with knowledge sharing, including media interviews and op-eds.

DECLARATIONS

For peer review only

Ethics approvals and consent to participate

The trial has been approved in Leicester (East Midlands – Nottingham Research Ethics Committee [21/EM/0026]), Montreal (McGill University Health Centre Research Ethics Board [RESET for remission / 2021-7148]), and Edmonton (University of Alberta Health Research Ethics Board (Pro00101088). Trial candidates will be informed of all study procedures, with opportunity for questions and reflection. We will underscore confidentiality, voluntariness and freedom to withdraw from the trial at any time point. A trained member of the research team, named on the delegation of authority log, will obtain written informed consent will from trial candidates so that they may become participants. The Trial Steering Committee will review any proposed amendments to approved study documentation and these will then be submitted to the relevant Research Ethics Boards for re-approval prior to implementation. Any participant who wishes to make a complaint may do so through the standard National Health Service complaint system in Leicester, the McGill University Health Centre Ombudsman in Montreal, or the Health Research Ethics Board in Edmonton, as appropriate.

We will maintain an audit trail throughout the lifetime of the trial and all related documentation will be made available for sponsor monitoring and/or any external audits, as by ethics' committees. To maintain participant confidentiality, each participant will be assigned a unique identification number that will be used, without nominal information, on case report forms and other trial documents. Most trial materials will be stored electronically in password-protected files on secure country-specific servers. The few documents in paper format will include the study identification number without nominal information and will be held in a locked cabinet accessible only to the research team at each trial site. We will hold participant identifiable information such as name and address in a trial site-specific, separate locked archive. All research data generated by the study will be stored for 25 years following trial completion, after which it will be destroyed.

For peer review only - http://bmjopen.bmj.com/site/about/guidelines.xhtml

591

592 **Consent for publication**

593 Not applicable

594 **Availability of data and materials**

595 Following the publication of the primary papers and all ancillary analyses by the research
596 team, datasets will be available from the principal investigators (UK: Prof. Tom Yates -
597 ty20@leicester.ac.uk) or (Canada: Prof. Kaberi Dasgupta - kaberi.dasgupta@mcgill.ca),
598 contingent on the agreement of both Principal investigators and the associated Research
599 Ethics Boards, on reasonable request. Any publications stemming from these data will be
600 reported on group-level only so that individual participant anonymity is maintained.

601

602 **Competing interests and disclosures**

603 MGF is board member, shareholder, and consultant of Circle Cardiovascular Imaging
604 Inc. Through the University Hospitals of Leicester NHS Trust, Co-investigator GM has a
605 research agreement with Circle Cardiovascular Imaging Inc. We will be using software from
606 this company to analyze the cardiac MRI images that we will obtain. K.K. has received
607 honoraria from AstraZeneca, Boehringer Ingelheim, Eli Lilly, Janssen, Merck Sharp &
608 Dohme, Novartis, Novo Nordisk, Sanofi, Takeda, Servier, and Pfizer and research support
609 from AstraZeneca, Boehringer Ingelheim, Eli Lilly, Merck Sharp & Dohme, Novartis, Novo
610 Nordisk, Sanofi, and Pfizer. MJD has acted as consultant, advisory board member and
611 speaker for Novo Nordisk, Sanofi, Lilly and Boehringer Ingelheim, an advisory board
612 member and speaker for AstraZeneca, an advisory board member for Janssen, Lexicon,
613 Servier and Gilead Sciences Ltd and as a speaker for Napp Pharmaceuticals, Mitsubishi
614 Tanabe Pharma Corporation and Takeda Pharmaceuticals International Inc. She has received
615 grants in support of investigator and investigator initiated trials from Novo Nordisk, Sanofi-
616 Aventis, Lilly, Boehringer Ingelheim, Astrazeneca and Janssen. TY has received investigator
617 initiated funding for obesity-related research from AstraZeneca.

Funding

The primary funders of this trial are the Medical Research Council (MR/T031816/1) of the United Kingdom and the Canadian Institutes of Health Research (UCD-170584; Institutes of Musculoskeletal Research and of Nutrition, Metabolism & Diabetes), organizations that have contributed equally to this endeavour. Additional funding in Canada was obtained from the John R McConnell Foundation to support additional enrolment in Canada, as previously described. The sponsors and funders have no direct role in study design, data collection, management, analysis, interpretation of data, report writing or publications that arise from the trial.

Authors' contributions

Kaberi Dasgupta and Thomas Yates are the Principal Investigators of the trial and led the design and writing of this protocol, with critical input from Co-Investigators Normand Boulé and Joseph Henson, particularly related to the exercise intervention and fitness and strength evaluations; Stéphanie Chevalier, Emma Baldry, Frank Arsenyadis, and Carla Prado, experts in nutrition and body composition measures; Gerry McCann, Matthias Friedrich, and Richard Thompson, experts in MRI-based assessments of cardiac function and ectopic fat; Deborah Da Costa and Michelle Hadjiconstantinou, related to health coaching and process evaluation; Matthew McCarthy, Deborah Chan, Julia Champagne, and Jordan Rees as related to trial logistics and procedures; Melanie Davies, Mark Sherman, and Roseanne Yeung, with important input on medication management; Edward Gregg and Kamlesh Khunti, for comments on recruitment and overall procedures; Elham Rahme, as related to statistical considerations; and Isabel Fortier, with respect to the data management plan.

Acknowledgements

Not applicable

For peer review only - http://bmjopen.bmj.com/site/about/guidelines.xhtml

Abbreviations

AE – Adverse Event

BMI – Body Mass Index

CI – Confidence Interval

CMR – Cardiac magnetic resonance

COM-B - Capability, Opportunity, Motivation - Behaviour

DDS – Diabetes Distress Scale

DXA – Dual-energy X-Ray Absorptiometry

ECG - Electrocardiography

EQ-5D-5L – European QoL -5 Dimensions

HbA1c – Hemoglobin A1c (glycated haemoglobin)

MRI – Magnetic Resonance Imaging

PROBE - Prospective, randomised, open-label, blinded-endpoint trial

PROFIT1 - PROton Density Fat Fraction Imaging and Water T1-Mapping with Low B1+ Sensitivity

REDCap – Research Electronic Data Capture System

ScAT - Subcutaneous Abdominal Adipose Tissue

T2DM – Type 2 Diabetes Mellitus

UK – United Kingdom

VAT - Visceral Adipose Tissue

WHODAS – World Health Organisation Disability Assessment Schedule questionnaire

REFERENCES

For peer review only

For peer review only - http://bmjopen.bmj.com/site/about/guidelines.xhtml

1. Gong Q, Zhang P, Wang J, Ma J, An Y, Chen Y, et al. Morbidity and mortality after lifestyle intervention for people with impaires glucose tolerance: 30-year results of the Da Qing diabetes prevention outcome study. Lancet Diabetes Endocrinol. 2019;7(6):452-61.
2. Bjornstad P, Drews KL, Caprio S, Gubitosi-Klug R, Nathan DM, Tesfaldet B, et al. Long-Term Complications in Youth-Onset Type 2 Diabetes. N Engl J Med. 2021;385(5):416-26.
3. Dabelea D, Stafford JM, Mayer-Davis EJ, D'Agostino R, Jr., Dolan L, Imperatore G, et al. Association of Type 1 Diabetes vs Type 2 Diabetes Diagnosed During Childhood and Adolescence With Complications During Teenage Years and Young Adulthood. JAMA. 2017;317(8):825-35.
4. Lascar N, Brown J, Pattison H, Barnett A, Bailey C, Bellary S. Type 2 diabetes in adolescents and young adults. Lancet Diabetes Endocrinol. 2018;6(1):69-80.
5. Federation ID. Diabetes Atlas, 9th edition [updated 2019. Available from: http://www.idf.org/diabetesatlas.
6. Riddle MC, Cefalu WT, Evans PH, Gerstein HC, Nauck MA, Oh WK, et al. Consensus Report: Definition and Interpretation of Remission in Type 2 Diabetes. Diabetes Care. 2021. Aug 30:dci210034.
7. Riddle MC, Cefalu WT, Evans PH, Gerstein HC, Nauck MA, Oh WK, et al. Consensus report: Definition and interpretation of remission in type 2 diabetes. Diabet Med. 2021:e14669.
8. Riddle MC, Cefalu WT, Evans PH, Gerstein HC, Nauck MA, Oh WK, et al. Consensus Report: Definition and Interpretation of Remission in Type 2 Diabetes. J Clin Endocrinol Metab. 2021. Aug 30:dgab585.
9. Riddle MC, Cefalu WT, Evans PH, Gerstein HC, Nauck MA, Oh WK, et al. Consensus report: definition and interpretation of remission in type 2 diabetes. Diabetologia. 2021;64(11):2359-66.
10. Gregg EW, Chen H, Wagenknecht LE, Clark JM, Delahanty LM, Bantle J, et al. Association of an intensive lifestyle intervention with remission of type 2 diabetes. JAMA. 2012;308(23):2489-96.
11. Leslie WS, Ford I, Sattar N, Hollingsworth KG, Adamson A, Sniehotta FF, et al. The Diabetes Remission Clinical Trial (DiRECT): protocol for a cluster randomised trial. BMC Fam Pract. 2016;17:20.
12. Lean ME, Leslie WS, Barnes AC, Brosnahan N, Thom G, McCombie L, et al. Primary care-led weight management for remission of type 2 diabetes (DiRECT): an open-label, cluster-randomised trial. Lancet. 2018;391(10120):541-51.
13. Lean MEJ, Leslie WS, Barnes AC, Brosnahan N, Thom G, McCombie L, et al. Durability of a primary care-led weight-management intervention for remission of type 2 diabetes: 2-year results of the DiRECT open-label, cluster-randomised trial. Lancet Diabetes Endocrinol. 2019;7(5):344-55.
14. Taheri S, Zaghloul H, Chagoury O, Elhadad S, Ahmed SH, El Khatib N, et al. Effect of intensive lifestyle intervention on bodyweight and glycaemia in early type 2 diabetes (DIADEM-I): an open-label, parallel-group, randomised controlled trial. Lancet Diabetes Endocrinol. 2020;8(6):477-89.
15. Boule NG, Haddad E, Kenny GP, Wells GA, Sigal RJ. Effects of exercise on glycemic control and body mass in type 2 diabetes mellitus: A meta-analysis of controlled clinical trials. JAMA. 2001;286(10):1218-27.
16. Villareal DT, Aguirre L, Gurney AB, Waters DL, Sinacore DR, Colombo E, et al. Aerobic or Resistance Exercise, or Both, in Dieting Obese Older Adults. N Engl J Med. 2017;376(20):1943-55.
17. Yates T, Henson J, Sargeant J, King JA, Ahmad E, Zaccardi F, et al. Exercise, Pharmaceutical Therapies and Type 2 Diabetes: Looking beyond Glycemic Control to Whole Body Health and Function. Translational Medicine and Exercise Prescription. 2021;1(1):33-42.

For peer review only - http://bmjopen.bmj.com/site/about/guidelines.xhtml

18. Anand V, Garg S, Garg J, Bano S, Pritzker M. Impact of exercise training on cardiac function among patients with type 2 diabetes: a systematic review and meta-analysis. Journal of cardiopulmonary rehabilitation and prevention. 2018;38(6):358-65.

19. Verboven M, Van Ryckeghem L, Belkhouribchia J, Dendale P, Eijnde B, Hansen D, et al. Effect of exercise intervention on cardiac function in type 2 diabetes mellitus: a systematic review. Sports Medicine 2019;49(2):255-68.

20. Levelt E, Gulsin G, Neubauer S, McCann G. MECHANISMS IN ENDOCRINOLOGY: Diabetic cardiomyopathy: Pathophysiology and potential metabolic interventions state of the art review. Eur J Endocrinol. 2018;178(4):R127-R39.

21. Boonman-de Winter L, Rutten F, Cramer M, Landman M, Liem A, Rutten G, et al. High prevalence of previously unknown heart failure and left ventricular dysfunction in patients with type 2 diabetes. Diabetologia. 2012;55(8):2154-62.

22. Khan J, Wilmot E, Leggate M, Singh A, Yates T, Nimmo M, et al. Subclinical diastolic dysfunction in young adults with type 2 diabetes mellitus: A multiparametric contrast-enhanced cardiovascular magnetic resonance pilot study assessing potential mechanisms. European heart journal cardiovascular Imaging. 2014;15(11):1263-9.

23. Wilmot EG, Leggate M, Khan JN, Yates T, Gorely T, Bodicoat DH, et al. Type 2 diabetes mellitus and obesity in young adults: The extreme phenotype with early cardiovascular dysfunction. Diabet Med. 2014;31(7):794-8.

24. Gulsin GS, Swarbrick DJ, Athithan L, Brady EM, Henson J, Baldry E, et al. Effects of Low-Energy Diet or Exercise on Cardiovascular Function in Working-Age Adults With Type 2 Diabetes: A Prospective, Randomized, Open-Label, Blinded End Point Trial. Diabetes Care. 2020;43(6):1300-10.

25. Sigal RJ, Kenny GP, Boulé NG, Wells GA, Prud'homme D, Fortier M, et al. Effects of aerobic training, resistance training, or both on glycemic control in type 2 diabetes: a randomized trial. Ann Intern Med. 2007;147(6):357-69.

26. Graham-Brown M, Gulsin G, Parke K, Wormleighton J, Lai F, Athithan L, et al. A comparison of the reproducibility of two cine-derived strain software programmes in disease states. Eur J Radiol. 2019;113:51-8.

27. Gulsin GS, Swarbrick DJ, Hunt WH, Levelt E, Graham-Brown MPM, Parke KS, et al. Relation of Aortic Stiffness to Left Ventricular Remodeling in Younger Adults With Type 2 Diabetes. Diabetes. 2018;67(7):1395-400.

28. Singh A, Greenwood JP, Berry C, Dawson DK, Hogrefe K, Kelly DJ, et al. Comparison of exercise testing and CMR measured myocardial perfusion reserve for predicting outcome in asymptomatic aortic stenosis: the PRognostic Importance of MIcrovascular Dysfunction in Aortic Stenosis (PRIMID AS) Study. Eur Heart J. 2017;38(16):1222-9.

29. Taylor R, Al-Mrabeh A, Sattar N. Understanding the mechanisms of reversal of type 2 diabetes. Lancet Diabetes Endocrinol. 2019;7(9):726-36.

30. Thompson RB, Chow K, Mager D, Pagano JJ, Grenier J. Simultaneous proton density fat-fraction and R2∗ imaging with water-specific T(1) mapping (PROFIT(1)): application in liver. Magnetic resonance in medicine. 2021;85(1):223-38.

31. Roubille F, Fischer K, Guensch DP, Tardif JC, Friedrich MG. Impact of hyperventilation and apnea on myocardial oxygenation in patients with obstructive sleep apnea - An oxygenation-sensitive CMR study. J Cardiol. 2017;69(2):489-94.

32. Spath NB, Singh T, Papanastasiou G, Kershaw L, Baker AH, Janiczek RL, et al. Manganese-enhanced magnetic resonance imaging in dilated cardiomyopathy and hypertrophic cardiomyopathy. European heart journal cardiovascular Imaging. 2020. Nov 17:jeaa273.

33. Pavasini R, Guralnik J, Brown J, di Bari M, Cesari M, Landi F, et al. Short physical performance battery and all-cause mortality: Systematic review and meta-analysis. BMC Med. 2016;14(1):215-016-0763-7.

34. World Health Organisation: WHO disability assessment schedule 2.0 (WHODAS 2.0) [updated 2018. Available from: https://www.who.int/classifications/icf/whodasii/en/.

For peer review only - http://bmjopen.bmj.com/site/about/guidelines.xhtml

35. Mahler D, Wells C. Evaluation of clinical methods for rating dyspnea. Chest. 1988;93(3):580-6.

36. Migueles JH, Rowlands AV, Huber F, Sabia S, van Hees VT. GGIR: A Research Community–Driven Open Source R Package for Generating Physical Activity and Sleep Outcomes From Multi-Day Raw Accelerometer Data Journal for the Measurement of Physical Behaviour. 2019;2(3):188-96.

37. Zigmond A, Snaith R. The hospital anxiety and depression scale. Acta Psychiatr Scand. 1983;67(6):361-70.

38. Polonsky W, Fisher L, Earles J, Dudl R, Lees J, Mullan J, et al. Assessing psychosocial distress in diabetes: Development of the diabetes distress scale. Diabetes Care. 2005;28(3):626-31.

39. Herdman M, Gudex C, Lloyd A, Janssen M, Kind P, Parkin D, et al. Development and preliminary testing of the new five-level version of EQ-5D (EQ-5D-5L). Qual Life Res. 2011;20(10):1727-36.

40. Sandelowski M. What's in a name? Qualitative description revisited. Res Nurs Health. 2010;33(1):77-84.

41. Palinkas L, Horwitz S, Green C, Wisdom J, Duan N, Hoagwood K. Purposeful sampling for qualitative data collection and analysis in mixed method implementation research. Adm Policy Ment Health. 2015;42(5):533-44.

42. Murray E, Treweek S, Pope C, MacFarlane A, Ballini L, Dowrick C, et al. Normalisation process theory: A framework for developing, evaluating and implementing complex interventions. BMC Med. 2010;8:63-7015-8-63.

43. Michie S, van Stralen MM, West R. The behaviour change wheel: a new method for characterising and designing behaviour change interventions. Implement Sci. 2011;6:42.

44. Braun V, Clarke V. Using thematic analysis in psychology. Qualitative Research in Psychology. 2006;3(2):77-101.

45. Baldry EL, Davies MJ, Khunti K, Webb DR. Pragmatic management of low-energy diets in people with type 2 diabetes in primary care: a decision aid for clinicians. Diabet Med. 2020;37(5):747-51.

46. Leslie WS, Ali E, Harris L, Messow CM, Brosnahan NT, Thom G, et al. Antihypertensive medication needs and blood pressure control with weight loss in the Diabetes Remission Clinical Trial (DiRECT). Diabetologia. 2021;64(9):1927-38.

47. Terada T FA, Chahal BS, Bell GJ, McCargar LJ, Boulé NG. Feasibility and preliminary efficacy of high intensity interval training in type 2 diabetes. Diabetes Res Clin Pract. 2013;99(2):120-9.

48. Jelleyman C, Yates T, O'Donovan G, Gray LJ, King JA, Khunti K, et al. The effects of high-intensity interval training on glucose regulation and insulin resistance: a meta-analysis. Obes Rev. 2015;16(11):942-61.

49. Francois M, Little J. Effectiveness and safety of high-intensity interval training in patients with type 2 diabetes. Diabetes Spectr. 2015;28(1):39-44.

50. Cassidy S, Thoma C, Houghton D, Trenell M. High-intensity interval training: A review of its impact on glucose control and cardiometabolic health. Diabetologia. 2017;60(1):7-23.

51. Type 2 diabetes in adults: management NICE guideline [NG28] 2015 [updated 2021. Available from: https://www.nice.org.uk/guidance/ng28.

52. Guidelines DCCP. 2020 [Available from: http://guidelines.diabetes.ca/cpg.

53. Astbury NM, Aveyard P, Nickless A, Hood K, Corfield K, Lowe R, et al. Doctor Referral of Overweight People to Low Energy total diet replacement Treatment (DROPLET): pragmatic randomised controlled trial. Bmj. 2018;362:k3760.

54. Ross R, Hudson R, Stotz PJ, Lam M. Effects of exercise amount and intensity on abdominal obesity and glucose tolerance in obese adults: a randomized trial. Ann Intern Med. 2015;162(5):325-34.

For peer review only

For peer review only - http://bmjopen.bmj.com/site/about/guidelines.xhtml

55. Parretti H, Jebb S, Johns D, Lewis A, Christian-Brown A, Aveyard P. Clinical effectiveness of very-low-energy diets in the management of weight loss: a systematic review and meta-analysis of randomized controlled trials. Obes Rev. 2016;17(3):225-34.
56. Brooks J, McCluskey S, Turley E, King N. The utility of template analysis in qualitative psychology research. Qual Res Psychol. 2015;12(2):202-22.
57. Yates T, Davies M, Gorely T, Bull F, Khunti K. Effectiveness of a pragmatic education program designed to promote walking activity in individuals with impaired glucose tolerance: a randomized controlled trial. Diabetes Care. 2009;32(8):1404-10.
58. Dasgupta K, Rosenberg E, Joseph L, Cooke AB, Trudeau L, Bacon SL, et al. Physician step prescription and monitoring to improve ARTERial health (SMARTER): A randomized controlled trial in patients with type 2 diabetes and hypertension. Diabetes Obes Metab. 2017;19(5):695-704.
59. Boulé NG, Haddad E, Kenny GP, Wells GA, Sigal RJ. Effects of exercise on glycemic control and body mass in type 2 diabetes mellitus: a meta-analysis of controlled clinical trials. Jama. 2001;286(10):1218-27.
60. Paffenbarger RS, Jr., Hyde RT, Wing AL, Hsieh CC. Physical activity, all-cause mortality, and longevity of college alumni. N Engl J Med. 1986;314(10):605-13.
61. Hu FB, Willett WC, Li T, Stampfer MJ, Colditz GA, Manson JE. Adiposity as compared with physical activity in predicting mortality among women. N Engl J Med. 2004;351(26):2694-703.
62. Florido R, Kwak L, Lazo M, Nambi V, Ahmed HM, Hegde SM, et al. Six-Year Changes in Physical Activity and the Risk of Incident Heart Failure: ARIC Study. Circulation. 2018;137(20):2142-51.
63. Moholdt T, Lavie CJ, Nauman J. Sustained Physical Activity, Not Weight Loss, Associated With Improved Survival in Coronary Heart Disease. J Am Coll Cardiol. 2018;71(10):1094-101.
64. Beaulieu K, Blundell JE, van Baak MA, Battista F, Busetto L, Carraça EV, et al. Effect of exercise training interventions on energy intake and appetite control in adults with overweight or obesity: A systematic review and meta-analysis. Obes Rev. 2021;22 Suppl 4(Suppl 4):e13251.
65. Cummings DE, Purnell JQ, Frayo RS, Schmidova K, Wisse BE, Weigle DS. A preprandial rise in plasma ghrelin levels suggests a role in meal initiation in humans. Diabetes. 2001;50(8):1714-9.
66. Heden TD, Liu Y, Kanaley JA. Impact of Exercise Timing on Appetite Regulation in Individuals with Type 2 Diabetes. Med Sci Sports Exerc. 2016;48(2):182-9.
67. Aylett E, Small N, Bower P. Exercise in the treatment of clinical anxiety in general practice - a systematic review and meta-analysis. BMC health services research. 2018;18(1):559.
68. Schuch FB, Vancampfort D, Firth J, Rosenbaum S, Ward PB, Silva ES, et al. Physical Activity and Incident Depression: A Meta-Analysis of Prospective Cohort Studies. Am J Psychiatry. 2018;175(7):631-48.

For peer review only

FIGURE 1. TRIAL SCHEMATIC

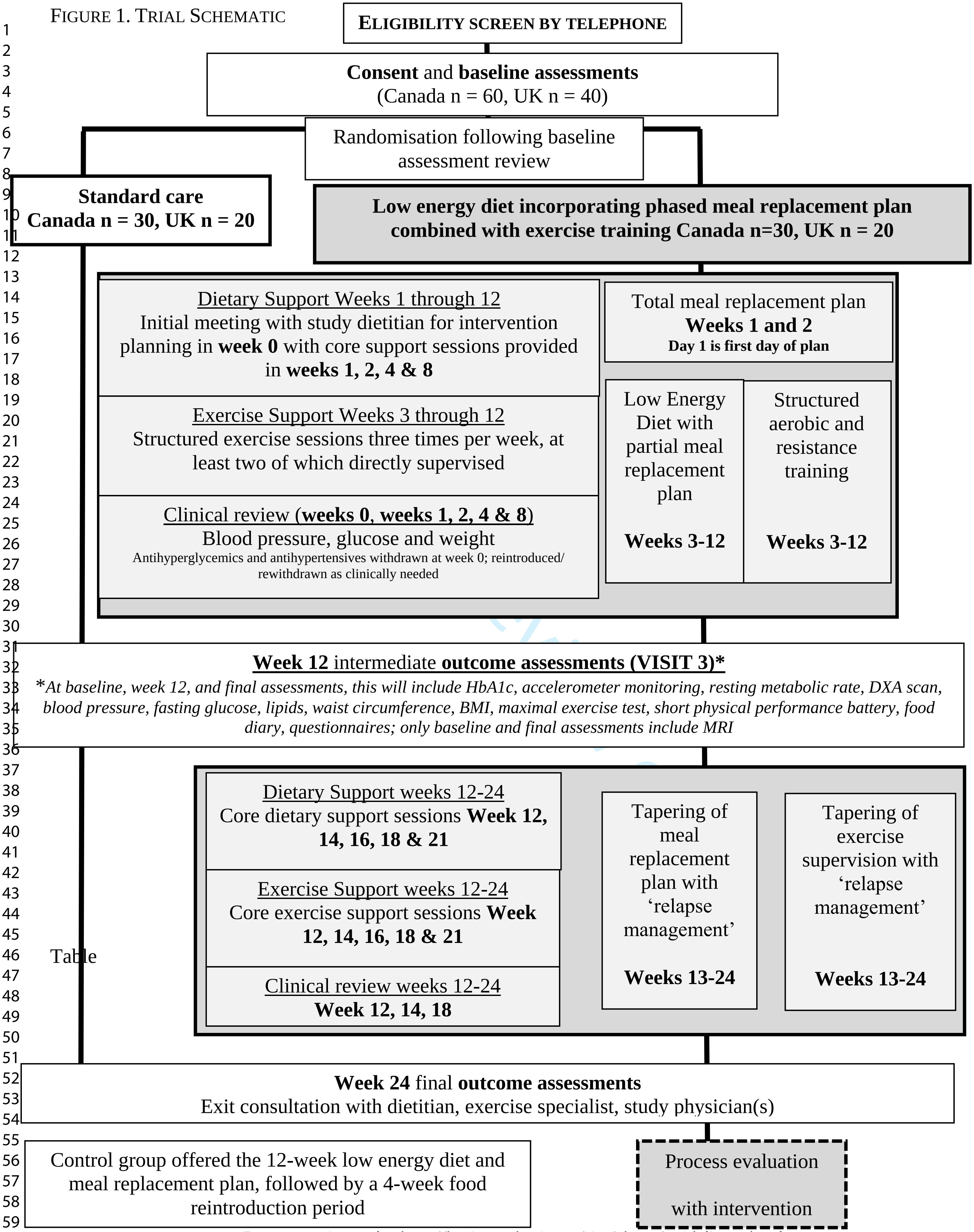

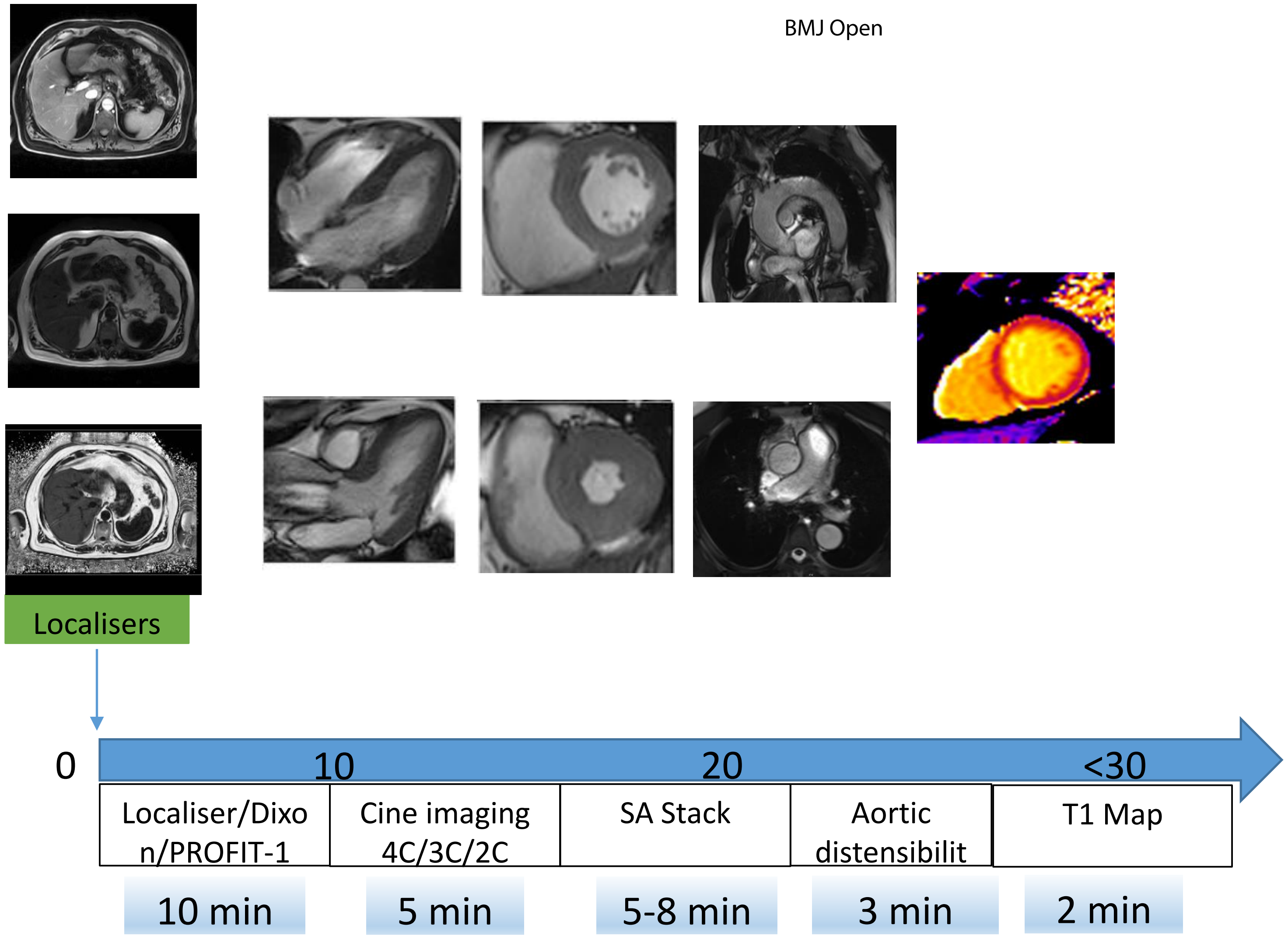

Figure 2: Core MRI protocol of the RESET FOR REMISSION trial.

Legend: The core protocol can be completed in under 30 minutes and comprises: localisers, fat-water imaging (Dixon) which allow assessment of subcutaneous and visceral adiposity (optional PROFIT-1 which adjusts liver fat measurement for fibrosis); cardiac cine imaging comprises long axes and complete coverage of the left ventricle and perpendicular to the thoracic aorta allowing calculation of myocardial volumes, mass, ejection fraction, strain/strain rates (see figure 3) and aortic distensibility; finally native T1 mapping in a single midventricular slice will be acquired as a surrogate marker of myocardial fibrosis.

Abbreviations: PROFIT1: simultaneous proton density fat fraction imaging and water T1-mapping with low B1+ sensitivity; 4C, 4-chamber; 3C, 3-chamber; 2C, 2-chamber. SA, short-axis.

For peer review only - http://bmjopen.bmj.com/site/about/guidelines.xhtml

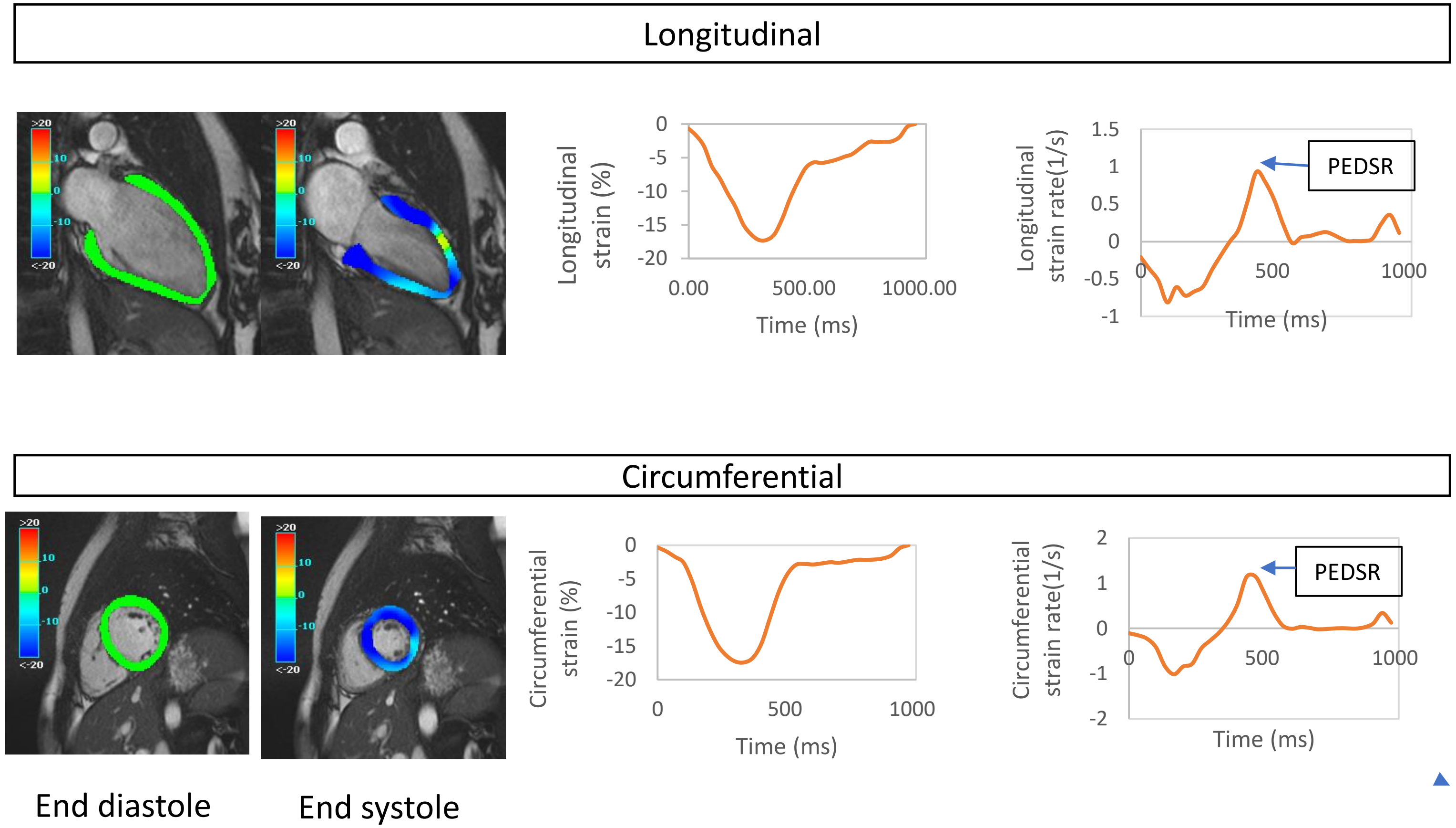

Figure 3: A typical example of strain analysis from cardiac MRI cine imaging.

Top panel 2 chamber view which allows derivation of longitudinal strain/strain rate; bottom panel mid ventricular short axis from which circumferential strain and strain rate is derived. Left: images showing color representations of longitudinal and circumferential strain at end-diastole and end-systole, with green showing 0%, light blue showing -10% and dark blue showing -20% strain (more negative indicating higher strain). Middle: graphs showing global longitudinal strain and global circumferential strain. Right: graphs showing longitudinal and circumferential strain rate throughout the cardiac cycle.

Abbreviations: PEDSR= peak early diastolic strain rate, a sensitive measure of diastolic relaxation and a key secondary outcome

Appendix 1. Interview guide for in-depth interviews with intervention arm participants at completion of final evaluations

This topic guide is a flexible tool and may be revised as new areas of interest arise during the process of data collection. The wording of the question is for guidance only and can be varied to suit the natural style of the interviewer and the level of understanding of the participant. This topic guide will be adapted to use for those who are involved in this study.

The interviewer will be a trial team member who has not interacted with the participant during the course of the trial. The interview may be conducted in person, by video teleconference, or by telephone, as preferred by the participant.

Introductory remarks

The interviewer will iterate that the purpose of the interview is to:

- Explore the participant's knowledge, views and experience of the RESET trial interventions

Participants will be assured that:

- They are not being 'tested' – there are no right or wrong answers.
- They are free to say as much or as little as they want.
- Their specific comments will be anonymously quoted in manuscripts and reports. Individual comments will not be shared with anyone external to the research team.

Recording will be started after introductory remarks.

Questions to discuss

1. What made you want to participate in this study?

For peer review only

2. When you started, did you feel you were you prepared to invest time and effort to make the intervention work?

3. When you were assigned to the 'active intervention' group, what was your understanding of how the intervention could benefit you?

4. What are your thoughts about this programme overall? Probe: What did you think about the format? The length of the sessions? What did you find easy to do? What was difficult?

5. Were you able to attend the sessions? Probe: if participants respond no, probe reasons for not being able to attend.

6. Was the venue convenient for the exercise training? (i.e. easy to attend) If not, what would have helped to make the venue convenient to you?

7. Dietary component:

   7a) What was your experience with the dietary support sessions? Prompt: meal replacement products, dietary support sessions, limited amounts of food

   7b) How did you find the reintroduction to food? Prompt: questions around pace, was it manageable, did they require more support etc.

   7c) Can you tell us if you felt the sessions helped in any way to manage your diet? Probe in what way do you feel this has helped, or has not helped?

For peer review only

8. Exercise component:

8a) You took part in exercise training (combined aerobic and resistance activities) – Can you tell me a little bit about this? Prompt: difficulty, convenience, motivation, challenges in combination with diet

8b) How well were you able to exercise independently once the close supervision of the first 12 weeks ended? Prompt: Challenges, need for return to supervision

8c) Can you tell us if you felt the sessions helped in any way to manage your exercise training? Probe: in what way do you feel this has helped, or has not helped?

9. Medication adjustments, glucose and blood pressure:

9a) How did you feel about stopping some medications at the beginning of the program? Probe: What concerns, if any, did you have?

9b) How did you feel about your glucose levels and blood pressure levels during the program? Probe: Did you feel that you were well-monitored during this period?

10. Did you make any changes in your eating? Probe: If yes, when you were making your diet changes, did you feel you were making a difference to your health?

11. Did you make any changes in your exercise? Probe: If yes, when you were making your exercise changes, did you feel you were making a difference to your health?

12. What skills do you feel you have taken from taking part in the intervention?

13. Diet component

13a) Were there any tasks or time constraints that would influence your ability in making changes to your diet? Elaborate

For peer review only

13b) What factors would you say influenced whether you were able to maintain your diet plan?

14. Exercise component

14a) Were there any tasks or time constraints that would influence your ability in making changes to you exercise? Elaborate

14b) What factors would you say influenced whether you were able to maintain your exercise plan?

15. Did you ever discuss the programme with your family members or friends? Probe: What did they think of your participation? Were they supportive? Concerned? Were they sceptical?

16. Would your family members or friends influence what you did as part of the programme? If so, to what extent? Prompt: i.e. was their influence positive? Was it negative?

17. Do you feel the intervention affected your relationships in any way? Prompt: i.e. by not eating regular food based meals with the family

18. If you made changes to your eating or physical activity, how important do you think it is for you to maintain these changes to your health? (elaborate why this is or isn't important to the participants)

For peer review only

19. How optimistic are you about maintaining these changes to your health? Prompt: if not optimistic, explore the reasons why

20. If there was additional support available in the intervention, what would this be?

21. What would you do differently if you were asked to take part again?

22. Do you have any suggestions for improving the programme?

For peer review only

Centre universitaire de santé McGill McGill University Health Centre

Centre universitaire de santé McGill McGill University Health Centre

X HME MCH X HGM MGH X HRV RVH
X HNM MNH X ITM MCI X CL LC

* F M U - 9 9 9 7 *

INFORMATION AND CONSENT FORM

**Research Study Title:**	REMISSION OF DIABETES AND IMPROVED DIASTOLIC FUNCTION BY COMBINING STRUCTURED EXERCISE WITH MEAL REPLACEMENT AND FOOD REINTRODUCTION IN ENGLAND AND CANADA: THE **RESET FOR REMISSION** TRIAL
**Protocol number:**	2021-7148
**Researcher responsible for the research study:**	Dr. Kaberi Dasgupta, Professor of Medicine and Physician, MUHC; Scientist, Research Institute of the MUHC
**Co-Investigators:**	Dr. Stéphanie Chevalier, Associate Professor of Medicine and Scientist, Research Institute of the MUHC<br>Dr. Deborah Da Costa, Associate Professor of Medicine and Scientist, Research Institute of the MUHC<br>Dr. Elham Rahme, Associate Professor of Medicine and Scientist, Research Institute of the MUHC<br>Dr. Matthias G. Friedrich, Professor of Medicine and Radiology and Physician, MUHC<br>Dr. Isabel Fortier, Assistant Professor of Medicine, MUHC; Scientist, Research Institute of the MUHC<br>Dr. Mark Sherman Associate Professor of Medicine and Physician, MUHC
**Funding source:**	The Canadian Institutes of Health Research (CIHR)
**Sponsor:**	The Research Institute of the MUHC

INTRODUCTION

We are inviting you to take part in this research study because you were diagnosed with Type 2 diabetes less than 6 years ago.

However, before you accept to take part in this study and sign this information and consent form, please take the time to read, understand and carefully examine the following information. You may also want to discuss this study with your doctor, a family member or a close friend.

We invite you to speak to the researcher responsible for this study ("the researcher") or to other

For peer review only

For peer review only - http://bmjopen.bmj.com/site/about/guidelines.xhtml

members of the research team and ask them any questions you may have about this study. Please also ask a member of the research team about any parts of this consent form you do not understand.

BACKGROUND

More and more people are getting type 2 diabetes. They are also getting it at younger ages (aged between 18-40 years) than in the past. This is worrying because heart, kidney and physical fitness problems related to diabetes can therefore also happen at an earlier age than in the past.

Past studies show that diets with a low number of calories can make blood sugar levels go back to normal without diabetes medications in people who have had type 2 diabetes for just a few years. This is called 'reversing diabetes.' A low calorie diet means eating a lot less than normal for some weeks often with special shakes and meal bars, with the guidance of a dietitian. Once people have lost enough weight, they can start to eat more regular food again while trying to keep off the weight they may have lost.

Exercise, such as walking and simple muscle building activities, can also make your blood sugar levels better and help your heart and muscles work more efficiently. Putting a low calorie diet *together* with a supervised exercise program has not been studied before to look at how well it works in reversing diabetes and improving how the heart and muscles work.

PURPOSE OF THE RESEARCH STUDY

The main goal of this study is to see whether combining exercise and a low calorie diet can reverse diabetes and make the heart and muscles work better.

This study is a randomized trial. This means that if you agree to participate in this study, you will be randomly assigned to one of two groups based on chance, like rolling a dice or flipping a coin. The two groups in this trial are:

- Group 1: **usual standard care** (that means continuing to be treated as you are right now)
- Group 2: a combination of an **exercise program and a low calorie diet**.

Neither you nor the researchers can choose the group to which you will be assigned. We will not know in which group you will be assigned until the trial begins. Both groups will have a series of tests and questionnaires at the beginning, middle, and at the end of the study, so we can compare the groups and what happens to them.

For this research study, we will recruit 30 to 35 people in Montreal. They will be men and women, between 18 and 40 years of age.

DESCRIPTION OF THE RESEARCH PROCEDURES

This research study will take place at the McGill University Health Centre. During the study, the research staff may communicate with you via e-mail, cell phone texting (standard text message rates apply) or video call.

For peer review only

For peer review only - http://bmjopen.bmj.com/site/about/guidelines.xhtml

Your participation in this research project will last 6 to 7 months and will include on-line questionnaires and at least 5 visits for testing (both Group 1 and Group 2). For those who are in the Group 2 (structured exercise with low calorie diet group), there will be more visits for counselling and supervision. Before we ask you to answer questionnaires or schedule visits, the study doctor will first go through your medical records and may talk to your doctor to make sure that you are eligible for the study. If you are eligible, we will ask you to complete the steps below.

1. Duration and number of visits

You will first complete an on-line questionnaire, an on-line 4-day food diary, and 1 week of physical activity monitoring with a device worn on your wrist. A paper-based alternative to the diary and questionnaires is also available, if you prefer. If you complete these procedures, you will be scheduled for the first two visits (visit 1 and visit 2 described in the table below). The study doctor will review the results of the first two visits to confirm that it is okay for you to continue the research. If it's okay, then we will do the randomization (like 'flipping a coin') to decide which of the two study groups you will be in.

2. Description of the trial groups and related procedures

GROUP 1- Standard care

If you are in the standard care group, you will receive your normal diabetes care. You will also be given a leaflet about the benefits of diet and exercise. You will come back for VISITS 3, 4, and 5, as described above. If you complete all visits, **you will be offered the low calorie diet at the end of the trial, in thanks for your participation.**

GROUP 2- Low calorie diet and structured exercise

Glucose and blood pressure medications and study physician supervision

We will stop your diabetes and most blood pressure medications at the beginning of the diet and exercise program. This is because the low calorie diet and the structured exercise will probably lower your blood sugar and blood pressure quite a lot. To be safe, we have to stop the medications.

A study doctor will see you at the beginning of the study and will stay in contact with you throughout the 24 weeks to monitor your blood sugar and blood pressure and decide with you if any medications need to be restarted. You will be asked to monitor your blood glucose more often. For study purposes, we will lend you a home blood pressure monitor and a weighing scale and provide you with glucometer strips if you do not have enough to measure your glucose levels at home.

You will measure your glucose levels, blood pressure, and body weight at home, and report these back to the study physician, dietitian, and exercise specialist, as they instruct. In general, you will be asked to check your glucose level every day before your first meal and 7 times the day before each time you meet with the study doctor (before and 2 hours after each 'meal' and at bedtime), your blood pressure when you wake up in the morning, and your body weight at least once each week. You will be measuring your glucose levels during the study with the usual way of pricking your finger with a lancet, placing a drop of blood on a test strip, and inserting the strip into a glucometer. You will measure your blood pressure with the monitor that we lend you during the study. You will sit comfortably with your back supported, attach the blood pressure cuff (like a band) on your upper arm, and rest for about 5 minutes. You will

For peer review only - http://bmjopen.bmj.com/site/about/guidelines.xhtml

then press a button to start the reading. Once the reading is done, you will push the button for a second reading, and this is the reading you will write down. You will write the blood pressure (first number and second number), body weight, and glucose values into an online diary that you and the study physician, dietitian, and exercise physiologist will be able to access. If you prefer, we can give you a paper diary instead. We will give you a fibre-based laxative (Metamucil) to take when needed in case you become constipated while on the low calorie diet described below.

We emphasize that it is very important not to become pregnant during the study. With this in mind, if you are female, the study physician will review your use of reliable contraception throughout the study. If you become pregnant, all study interventions will be stopped.

<u>The low calorie diet</u>
This diet will be a major change in your life for 12 weeks. You have to be aware of what is involved. You will have regular support from a study dietitian. This will be face to face or through telephone calls or virtual meetings.

Part 1 (2 weeks). You will have a “total dietary replacement” where you only consume special shakes and lots of water. The shakes will give you around 900 calories each day. All of the shakes will be free of charge and will be give to you the first time you meet the dietitian, which will be face-to-face at least the first time.

Part 2 (10 weeks). After the first two weeks, you will eat one meal with regular food each day. The rest of what you eat will be meal replacement products like the shakes you had the first two weeks. To give you more variety, on top of shakes, you will also get special soups and bars. On most of the days each week (4 out of 7 days), you will still have about 900 calories each day (all of the meal products and the one meal each day all considered together).

During this ‘part 2’ time, you will also be exercising 3 days each week. On the days that you exercise, you will have some extra meal replacement product so on those days you will be taking in a bit more than 900 calories.

You and the dietitian will figure out together what kinds of meals you will eat to make sure that you are not taking in more calories than the limit set by the study. She will work with you to pick foods that you prefer. She will also help you figure out what combination of shakes, bars, and soups you might like best.

Part 3 (12 weeks). At this point, you will probably have lost weight and we will now try to help you keep it off. Even if you have not lost weight, we will help you transition back to a healthy food-based diet. The dietitian will help you bring back normal foods. She will calculate about how many calories you will need every day to keep your weight the same as it is at 12 weeks. We will support you to figure out portion sizes and food choices. The goal is to help you keep off the weight you have lost. If you gain weight, she will reintroduce or increase some meal replacement products and remove some regular meals from your plan, for a period of time.

For peer review only - http://bmjopen.bmj.com/site/about/guidelines.xhtml

<u>The structured exercise program</u>

The exercise program will start after your first two weeks of shakes. We don't want to overwhelm you with too many changes at once, so we will start the exercise program at the same time that you get one food meal back in your diet at week 3.

Just as the dietitian supervises you in the low calorie diet part, an exercise specialist (exercise physiologist) will supervise you in the exercise part. The sessions are free of charge.

Part 1 – During the first 2 weeks when you are having the shakes only diet, you will continue with your usual activities.

Part 2 -- During the next 10 weeks you will exercise, you will have two supervised exercise sessions at the PERFORM Centre, located at 7272 Sherbrooke Street West. You will take part in lots of different exercises designed to increase your fitness and strength. The planned exercise will take into account your level of fitness and ability. You will use a treadmill, free weights, bands, and weight machines. The exercise specialist will help you use these safely and effectively. The specialist will design a third session for you to do each week. This 'unsupervised' session can be done outdoors, at another gym, or at home but you will also have the option to come to PERFORM for this, to access the equipment and facilities. Please note, however, that if there are any government-mandated COVID restrictions in place, the PERFORM gym will not be available for unsupervised exercise. You will wear a Fitbit device that we will lend you so that you and we can monitor your unsupervised exercise and activities of daily living.

Part 3 – During the next 12 weeks, you will exercise on your own. The goal is for you to keep exercising at the level and amount that you have been doing in part 2. The exercise specialist will make a plan with you and will be checking in with you by phone, text, or e-mail. If you are having difficulty or are not exercising regularly, you will be asked to return for some supervised sessions. When you are exercising on your own during these 12 weeks, you will still have the option of exercising at the PERFORM Centre. As we state above, however, if there are any government-mandated COVID restrictions in place, the PERFORM gym will not be available for unsupervised exercise. You will wear a Fitbit device that we will lend you to track your aerobic exercise and physical activity.

Additional glucose sensors

As we talked about above, you will be testing your blood sugar levels at least once a day at home and 7 times on the day before you have a visit with the study doctor. You and the study doctor will know all of these numbers. On top of that, we will measure your glucose levels throughout the day using a special device called a continuous blood glucose monitor (CGM). The study physician won't use the CGM numbers to guide you; she will stick to the numbers you measure yourself at home. We will use the CGM numbers for research analysis ('research math') to understand overall the patterns of blood sugar change in the two study groups. The research team will download and store these numbers for analyses.

We will show you how to place the CGM sensor on the skin of your upper arm. It has a small filament that is inserted under your skin with a small needle (less than 1 cm long). The needle is then removed and only the flexible filament remains under your skin. We will show you how to tape the sensor to

For peer review only

For peer review only - http://bmjopen.bmj.com/site/about/guidelines.xhtml

your arm. You can shower with it. Once in place, you do not need to do anything else with it. The CGM sensor measures your blood glucose continuously and stores values every 15 minutes.

We will ask you to wear a CGM sensor at 4 different time points, each time for two weeks. These are the 4 time blocks:
(1) The week before you start the low energy diet and the first week of the low energy diet;
(2) The second week of the low energy diet and the first week supervised exercise is added;
(3) The 11th and 12th weeks after you start the diet, when we should start seeing the strongest effects of what you are doing;
(4) The 23rd and 24th weeks when you are finishing up the study.

Each time you put on one of these sensors, you will mail it back to us after two weeks in a stamped envelope we will give you or you will bring it to the PERFORM centre where we will collect it from you.

3. Tests and procedures
After you agree to join the study, we will ask you to do the following:

Accelerometer to monitor your activity & sleep) for 7 days	We will mail you a small machine to wear on your wrist called a research accelerometer. This measures how much time you spend sleeping, sitting and moving. It can be worn all day and night. We will ask you to wear it for 7 days. You will then mail it back to us in the postage paid envelope that we will give you.
Food diary	We will explain to you how to log onto an online food diary or mail you a paper one to complete. We will ask you to track what you eat over 4 days (three weekdays and one weekend day). If you complete a paper diary, you will mail it back with the accelerometer. This will help you understand what your food habits are.
Questionnaire – done at home	We will ask you to fill out an online questionnaire (or paper version, if you prefer). You can reach our study staff by phone or email to answer any questions you have. You also have the option of answering the questions by phone and the staff can fill in the answers for you.<br>Examples of what the questions will be about are feeling anxious or depressed, how well you function in your day to day life, history of illness in your family, your cultural or ethnic background, any trouble breathing, how much schooling you completed, the kind of work you do if you are employed, and whether or not you have a partner. We will also ask female participants about previous pregnancies. We estimate that these questions will take about 20 minutes to complete.

If you do not complete the above procedures, you will not be invited to continue in the study. Once you complete the above procedures at home, you will be scheduled for some face-to-face visits for evaluation.

For peer review only

For peer review only - http://bmjopen.bmj.com/site/about/guidelines.xhtml

The study doctor or a member of the research team will conduct the following tests and procedures:

DESCRIPTION OF STUDY PROCEDURES	
**Procedures at visit 1, 3 and 4**	**Description**
Preparation for visits 1, 3, and 4 (fasting)	To make sure we have accurate results on the tests we are doing, we ask you not to exercise or drink alcohol for two days before you come to see us.<br>We also have to ask you to fast and not smoke the night and following early morning (about 10 hours total) before you come in to have accurate results on blood tests.<br>Do not take your diabetes medications until we give you a snack later in the morning before the exercise test.<br>You can take the other medications that you would normally take at that time of day
Urine collection	We will ask you to go to the washroom to empty your bladder into a container that we will give you. We will use the urine sample to check how your kidneys are working. If you are a woman who is able to become pregnant, we will do a urine test for pregnancy before you start participating in the study. We will ask you to put on a hospital gown.
Height, weight, neck, waist and hip measures	We will ask you change into a hospital gown. We will weigh you (in the gown *without* shoes) and measure your neck, waist, hip and height.
Indirect calorimetry	This test will tell us how much energy you use when you are still. It reflects the amount of energy your body uses to keep your blood flowing and your organs working. You will lie still on a table under a clear hood, staying awake. A machine connected to the hood will measure how much oxygen you are breathing in and how much carbon dioxide you are breathing out.
Review of your medications	You will bring in a list of your medications (with doses) or the medication containers themselves. These will be noted down by study staff. They will ask you some more questions about your medical history and current health.
Blood pressure	We will ask you to sit down quietly for 5 minutes. We will then take one measure from the left arm and then the right arm. Then six measures will be taken 1-minute apart in the arm that had the higher value on the first measurement.
Bioelectrical Impedance Analysis (BIA) test	This test will estimate your body fat and 'lean' mass (muscle) by running a small electrical current through the body that you will not feel. You will stand on the machine and hold onto the bars.
Blood collection	We will take some blood (about 34 mL or roughly 7 teaspoons) to measure the amount of sugar and fat in your blood and other blood tests related to type 2 diabetes and the heart.
Fitness test	We will give you a snack (or if this is visit 3, a lunch) before the fitness test.

For peer review only

For peer review only - http://bmjopen.bmj.com/site/about/guidelines.xhtml

	You will be walking/running on a treadmill. The speed will remain the same but the incline of the treadmill will slowly increase. We will ask you to keep going as long as you can to figure out how fit you are. It will feel feel like climbing up a hill that gradually becomes steeper and steeper. You will be asked to breathe through a mask in order to collect and measure the carbon dioxide you breathe out and the oxygen you breathe in.
**Procedures at visit 2 and 5**	
Preparation for Visits 2 and 5	Please do not smoke, consume caffeine-containing beverages or foods (coffee, tea, cocoa, chocolate, "energy drink" or cola) for 12 hours before MRI visit and do not eat for 4 hours before the MRI Visit. If you are on a type of medication for blood pressure called a beta-blocker, they study doctor will ask you to not take it 12 hours before the MRI.
MRI	Magnetic Resonance Imaging (MRI) is an imaging method that uses a magnetic field with radio frequency waves, which allows doctors to see your organs inside your body. Before your scan, we will figure out if you have been taking a medication called a beta-blocker for high blood pressure; if yes, on the day of the scan, we will ask you to delay taking it until after the can. During the scan, you will lie on your back inside the scanner. The inside of the machine is quite small. Some people may start to feel claustrophobic. When the scanner is working, it makes loud buzzing sounds. Therefore, you will wear headphones. We will use the scanner to take pictures of the heart. We will also measure how much fat you have in and around your organs. The MRI will produce images and the computer will collect this information. Some images will require for you to hold your breath for a short period of time (2-15sec) and the technologist will give you specific instructions. It is very important that you don't move when the picture is taken, as this can blur the pictures. Your heart, blood pressure, and blood oxygen levels will be closely monitored during the test. Electrocardiogram (ECG) electrodes (stickers) will be placed on your chest. A blood pressure cuff will be placed around your upper arm. A pulse oximeter (a small device to measure the amount of oxygen in your blood) will be attached to one of your fingers.
DXA (Dual Energy X-ray Absorptiometry) scan	This test uses X-rays to measure the overall amount of fat and muscle you have in your body. We will also ask you to stay as still as possible. You will lie on a padded table. There will be a machine that makes X-rays below the table. There will be a moveable machine 'arm' above the table that detects the X-rays. The arm will move across your body above you.
Physical function tests	After a snack, you will do a few tests that will measure hand grip strength, balance, time it will take you to walk 4 metres and time it takes for you to rise from a chair.

For peer review only - http://bmjopen.bmj.com/site/about/guidelines.xhtml

The schedule of procedures for each visit is listed below:

	For everyone in the study (Group 1 and Group 2)					
**Procedure**	**Prior to visit 1**	**Visit 1**	**Visit 2**	**Visit 3**	**Visit 4**	**Visit 5**
**Duration of in-person visit (hrs:min)**	**At home**	**4:00**	**2:10**	**5:30**	**4:30**	**2:10**
**Timeline**			Done within 14 days of visit 1	12 weeks after visit 1	24 weeks after visit 1	24 weeks after visit 1
Urine test		X		X	X	
Height, weight, neck, waist and hip measures		X		X	X	
Indirect calorimetry test		X		X	X	
Pregnancy test (if necessary)		X		X	X	
Medication list review		X		X	X	
Blood pressure		X		X	X	
Bioelectrical Impedance Analysis (BIA)		X		X	X	
Fasting, blood test		X		X	X	
Snack given to participant		X			X	
Lunch given to participant				X		
Fitness test		X		X	X	
Wear a device to monitor your activity & sleep	X			X	X	
Food diary	X			X	X	
Online questionnaire (at-home)	X			X	X	
MRI			X			X
DXA			X	X		X
Snack given to participant			X	X		X
Physical function test			X	X		X

**For those in the Group 2 (low calorie diet and exercise group)**													
**Week #**	**0**	**1**	**2**	**3**	**4**	**8**	**10**	**12**	**14**	**16**	**18**	**21**	**24**
Session with study physician or health care professional	x	x	x		x	x		x	x		x		x
Blood pressure, weight and fasting glucose measure		x	x		x	x		x	x		x		
Blood glucose monitoring (finger prick testing)	x	x	x	x	x	x	x	x	x	x	x	x	x
Sessions with the dietitian	x	x	x		x	x		x	x	x	x	x	x
Supervised exercise training*				x	x	x	x	x					
Home-based exercise**									x	x	x	x	x
CGM sensor	x	x	x	x				x***					x***

For peer review only

For peer review only - http://bmjopen.bmj.com/site/about/guidelines.xhtml

*= At least two supervised exercise sessions weekly from weeks 3 to 12.
** At least one unsupervised/home-based exercise session weekly from weeks 3 to 12 and three sessions weekly or equivalent from weeks 13 through 24
*** Each time you will wear it for two weeks (weeks 11 and 12; weeks 23 and 24)

BENEFITS ASSOCIATED WITH THE RESEARCH STUDY

You may or may not personally benefit from your participation in this research project. However, we hope that the study results will contribute to the advancement of scientific knowledge in the study field and help us find better treatments for patients.

RISKS ASSOCIATED WITH THE RESEARCH STUDY

A possible risk associated with this study is a breach of confidentiality or use of your personal information by a third party. To limit this risk, we will take the steps to protect your confidentiality described in the Confidentiality section, below.

1. **Risks associated with the research intervention (group 2 only)**

Physical activity and exercise: With exercise, there are risks of injury to joints, bones, and muscles. If you wind up in the low energy diet and structured exercise group, during the supervised exercise sessions, you will receive advice on how to be safely active. You will participate in some exercises and may try out some gym equipment. Becoming physically active and exercising may sometimes lead to injury or bring to light health problems that were not obvious. You must inform study personnel and physician of any symptoms of shortness of breath, chest discomfort, or other pain. The study physician may have to further evaluate you. Our exercise specialists will ask you about any joint, muscle, or back problems that you may have. They will help you to tailor your exercise accordingly. It is your responsibility to follow their advice and inform them of any concerns you may have.

Low energy diet: In past studies, the low energy diet has led to symptoms like constipation (18 to 47%), dizziness (4 to 32%), fatigue (11 to 25%), thirst (6%), and/or headache (8%). These tend to get better with fibre-based laxatives and time. We will be giving you a fibre-based laxative to use as needed. It is also important to drink as much water as needed when on the diet.

Stopping medications: We will be stopping your diabetes and blood pressure medications at the beginning of the diet and exercise program. This will help to prevent drops in blood pressure and/or glucose that would happen if the low energy diet were combined with medications. Diet and exercise may lead to low blood sugar and low blood pressure, but this is unlikely when not taking medication. We will monitor your blood sugar and blood pressure levels with you during the study to see if at any point medications need to be restarted.

2. **Risks associated with research procedures.**

Blood tests: The taking of blood samples may cause some discomfort, fainting, formation of a small blood clot or swelling of the vein on surrounding tissue, bleeding from the puncture site, and /or rarely an infection. There is a possibility that you may faint. However, precautions will be taken to ensure your safety should this occur.

For peer review only

For peer review only - http://bmjopen.bmj.com/site/about/guidelines.xhtml

Treadmill testing: As with any type of strenuous activity, there is a some risk of a serious event (e.g. heart attack, hypoglycemia) during the exercise tests that you will perform during the study. If at any time during the exercise tests you do not wish to continue for any reason, you may stop exercising. A health professional and physician will always be available during the treadmill test and emergency equipment will always be available in case of a serious event.

Body composition DXA scans: These procedures like X-rays use radiation energy to form images of your body and provide your doctor with other clinical information. Radiation can cause cell damage that may, after many years or decades, turn cancerous. We are all at risk of developing cancer during our lifetime. The normal risk is that this will happen to about 50% of people at some point in their life. Taking part in this study will add only a very small chance of this happening to you.

MRI: We will not perform an MRI on pregnant women. The magnetic field of the MRI may pose a risk to the developing fetus. The MRI table, on which you will lie on, will slide inside the MRI scanner. Less than 5% of people have anxiety or a feeling of claustrophobia because of the small space that they are in during the test. The scanner will emit radio frequencies that generate a loud noise, which will be lessened by the headphones we will give you to wear. During the exam, you can talk to the technologist or you can ask to stop the exam at any time. About 10% of people have headache or nausea related to the strong magnetic field of the MRI; these are usually mild and they go away. Some people may also experience a sensation of heat, tingling in the body, nausea and discomfort due to the loud noise.

Questionnaires: Some questions may upset some participants. For example, we will ask you about use of drugs and alcohol, past history of miscarriage, and symptoms of pain, anxiety, and depression. Please let us know if this upsets you and how we can help to support you. We may be able to help you find online resources or places to seek counselling, if necessary. We could also assist you in communicating any issues to your doctor.

Glucose sensors: There is a low risk of infection or bruising from the insertion of the sensor. These could last for a few days. The skin of some individuals is sensitive to the adhesive and can get red or itchy when the CGM is attached. There may be redness in the area where the tape was applied but this will usually disappear after a few days.

RISKS ASSOCIATED WITH PREGNANCY

The approach we are using has not been tested for safety in pregnant women. Therefore, it is important not to become pregnant during the study. In addition, if you are sexually active, and could become pregnant you must use a medically accepted contraceptive method throughout your participation in the study.

The medically accepted contraceptive methods are oral contraception, hormonal implants, hormonal patches, IUD, diaphragm and spermicide, cervical cap with spermicide, and condom with spermicide. The study doctor or the research team will discuss your contraceptive method with you to ensure that it is medically accepted.

If you suspect that you have become pregnant during your participation in the research study, you must inform the study staff immediately. The study doctor and staff will help arrange testing and

For peer review only

For peer review only - http://bmjopen.bmj.com/site/about/guidelines.xhtml

referral, as appropriate. If you are in Group 2, we will stop the low energy diet and supervised exercise program. For either Group 1 or Group 2, we will not do the other test procedures like MRI or treadmill testing.

VOLUNTARY PARTICIPATION AND THE RIGHT TO WITHDRAW

Your participation in this research project is voluntary. Therefore, you may refuse to participate. You may also withdraw from the project at any time, without giving any reason, by informing the study doctor or a member of the research team.

Your decision not to participate in the study, or to withdraw from it, will have no impact on the quality of care and services to which you are otherwise entitled, or on your relationship with the study doctor or clinical team.

The study doctor, the Research Ethics Board, the funding agency, or the Sponsor may put an end to your participation without your consent. This may happen if new findings or information indicate that participation is no longer in your interest, if you do not follow study instructions, or if there are administrative reasons to terminate the project.

If you withdraw or are withdrawn from the study, the information and biological material already collected for the study will be stored, analyzed and used to ensure the integrity of the study.

Any new findings that could influence your decision to stay in the research project will be shared with you as soon as possible.

CONFIDENTIALITY

During your participation in this study, the study doctor and their team will collect and record information about you in a study file. They will only collect information required to meet the scientific goals of the study.

The study file may include information from your medical chart, including your identity, concerning your past and present state of health, your lifestyle, as well as the results of the tests, exams, and procedures that you will undergo during this research project. Your research file could also contain other information, such as your name, sex, date of birth and ethnic origin.

All the information collected during the research project will remain strictly confidential to the extent provided by law. You will only be identified by a code number. The key to the code linking your name to your study file will be kept by the study doctor.

To ensure your safety, a copy of this information and consent form will be placed in your medical chart. As a result, any person or company to whom you give access to your medical chart will have access to this information.

For peer review only - http://bmjopen.bmj.com/site/about/guidelines.xhtml

We are conducting this study in collaboration with researchers in the United Kingdom (UK) and Canada. We may share the data collected with them, without any identifying information. This is so we can analyse the results from all the sites participating in this study. This will be done in such a way that you cannot be recognised from it. For example, shared data will not include names, addresses or dates of birth.

The study data will be stored for 25 years after completion of the trial.

The data may be published or shared during scientific meetings; however, precautions will be taken to ensure that it will not be possible to identify you.

You have the right to consult your study file in order to verify the information gathered, and to have it corrected if necessary.

However, in order to protect the scientific integrity of the research project, accessing certain information before the project is ended may require that you be withdrawn from the study.

For auditing purposes, the research study files which could include documents that may identify you may be examined by a person mandated by the study sponsor, the institution, or the Research Ethics Board. All these individuals and organizations adhere to policies on confidentiality.

INCIDENTAL FINDINGS

Material incidental findings are findings made in the course of the study that may have significant impacts on your current or future wellbeing or that of your family members. A material incidental finding concerning you in the course of this research will be communicated to you and to a health professional of your choice.

FUNDING OF THE RESEARCH PROJECT

The researcher and the institution have received funding from the Canadian Institutes of Health Research to conduct this research project.

COMPENSATION

You will receive an amount of $10 per **visit to the Glen hospital** (to cover parking and public transit costs) for each of the 5 study-related visits (see Table titled ***For everyone in the study (Group 1 and Group 2)*****).**

For Group 2, parking costs at PERFORM will be paid directly by the study team to the PERFORM centre. The meal replacements and supervised exercise sessions (where applicable) will be offered to you for free for the duration of this research study.

SHOULD YOU SUFFER ANY HARM

Should you suffer harm of any kind due to a procedure related to the research study, you will receive the appropriate care and services required by your state of health.

By agreeing to participate in this research project, you are not waiving any of your legal rights nor discharging the study doctor, the sponsor or the institution, of their civil and professional responsibilities.

For peer review only - http://bmjopen.bmj.com/site/about/guidelines.xhtml

For peer review only

WHAT ABOUT COVID-19?

Your safety and that of our research team will be paramount. The study will comply with the latest recommendations and laws. This includes physical distancing and the use of personal protective equipment. If things change during the course of the study, we may need to change how we run the study. This may mean greater use of remote consultations and monitoring. The amount of exercise done at home may also need to be increased.

CLINICAL TRIAL REGISTRATION

A description of this clinical trial will be available on http://www.ClinicalTrials.gov. This Website will not include information that can identify you. At most, the Website will include a summary of the results. You can search this Website at any moment.

CONTACT INFORMATION

If you have questions or if you have a problem you think may be related to your participation in this research study, or if you would like to withdraw, you may communicate with:

- The Principal Investigator, Dr. Kaberi Dasgupta, at the McGill University Health Centre, at 514-934-1934, then 1 then ext. 44715; or
- The main research coordinator of the study, Ms. Debbie Chan, at 514-934-1934 then 1 then ext. 44835 or by email at debbie.chan@rimuhc.ca

For any question concerning your rights as a research participant taking part in this study, or if you have comments, or wish to file a complaint, you may communicate with McGill University Health Centre Ombudsman: 514-934-1934, then 1 then ext. 35655

OVERVIEW OF ETHICAL ASPECTS OF THE RESEARCH

The McGill University Health Centre Research Ethics Board reviewed this research and is responsible for monitoring the study.

For peer review only

For peer review only - http://bmjopen.bmj.com/site/about/guidelines.xhtml

SIGNATURES

Signature of the participant

I have reviewed the information and consent form. Both the research study and the information and consent form were explained to me. My questions were answered, and I was given sufficient time to make a decision. After reflection, I consent to participate in this research study in accordance with the conditions stated above.

I authorize the research study team to have access to my medical record for the purposes of this study.

I authorize the study doctor to inform my treating physician that I am taking part in this study.

I authorize the doctor in charge of this research study to communicate with me directly to ask if I am interested in participating in other research

I understand that my doctor may be contacted to provide information about my health status to the research team for confirmation of eligibility.

Name and contact information of treating physician: ______________________________

Physician's Address: _________________________________

Physician's phone number:________-_________-____________

I understand that the study doctor will send my treating physician health information if it will be useful for my care.

____

Name of participant Signature Date (dd/mmm/yyyy)

Signature of the person obtaining consent

I have explained the research study and the terms of this information and consent form to the research participant, and I answered all his/her questions.

____

Name of the person obtaining consent Signature Date (dd/mmm/yyyy)

For peer review only

For peer review only - http://bmjopen.bmj.com/site/about/guidelines.xhtml

DMC Charter for R4R | Author: Thomas Yates and Kaberi Dasgupta

Date: 15/01/2021 | Version: 1.0

RESET 4 REMISSION Trial DMC Charter

**1. INTRODUCTION**	
Name (and sponsor's ID) of trial plus ISRCTN and/or EUDRACT number	REmission of diabetes and improved diastolic function by combining Structured Exercise with meal replacemenT and food reintroduction: THE RESET FOR REMISSION TRIAL<br>Sponsor: University of Leicester and McGill University<br>Registration number: *To be included*
Objectives of trial, including interventions being investigated	**Primary objective**<br>The aim of this efficacy study is to investigate whether combining a low energy diet incorporating the phased use of meal replacement products, with structured exercise training leads to remission of T2DM in younger adults (18-40 years) over a 24-week period, in comparison with usual care. Remission is defined as an hba1c value less than 6.5% at 24 weeks and no antihyperglycemic medications during weeks 13 through 24 weeks of the 24 week study period.<br><br>**Secondary objectives**<br>Key secondary objectives will examine the effects of the combined intervention on cardiovascular and functional health, particularly MRI-assessed diastolic function and cardiometabolic risk factors such as lipid profile and peripheral blood pressure, along with cardiorespiratory fitness, physical function, lean mass and basal metabolic rate. We will also undertake a process evaluation, to ensure the findings from our research can be used to inform how the interventional components are refined and translated.<br><br>Figure 1 shows a flow chart of the trial design.
Outline of scope of charter	The purpose of this document is to describe membership, terms of reference, roles, responsibilities, authority, decision-making and relationships of the independent Data Monitoring Committee (DMC) for the RESET 4 REMISSION trial. This will include the timing of meetings, methods of providing information to and from the DMC, frequency and format of meetings and relationships with other trial committees such as the Trial Steering Committee (TSC).
**2. ROLES AND RESPONSIBILITIES**	
A broad statement of the aims of the Data Monitoring Committee	To safeguard the interests of trial participants, assess the safety of the interventions during the trial, and monitor the overall conduct of the clinical trial.
Terms of reference	The DMC should receive and review the progress and accruing data of this trial and provide advice on the conduct of the trial to the Trial Steering Committee.
Specific roles of DMC	To undertake reviews of the trial's progress, including updated figures on recruitment, data quality, and main outcomes and safety data, by:<br>• assess data completeness (and by so doing encourage collection of high-quality data)<br>• monitor recruitment figures and losses to follow-up<br>• monitor compliance with the protocol by participants and investigators<br>• monitor evidence for any potential treatment harm (e.g., Serious Adverse Events or deaths)

For peer review only - http://bmjopen.bmj.com/site/about/guidelines.xhtml

RESET 4 REMISSION Trial DMC Charter

	• suggest additional data analyses as part of the review of the statistical analysis plan<br>• advise on protocol modifications suggested by investigators or sponsors (e.g., to inclusion criteria, trial endpoints, or sample size)<br>• monitor planned sample size assumptions<br>• monitor continuing appropriateness of participant information sheets<br>• monitor compliance with previous DMC recommendations<br>• assess the impact and relevance of external evidence as requested by the TSC, Trial Sponsors (University of Leicester and Research Institute of the McGill University Health Centre [RI-MUHC]) or Trial Funders (The UK Medical Research Council [MRC] and The Canadian Institutes of Health Research [CIHR])<br>• if funding is required above the level originally requested, the DMC may be asked by the Principal (Chief) Investigators, TSC, Trial Sponsors or Trial Funders to provide advice and, where appropriate, information on the data gathered to date in a way that will not compromise the trial.
**3. BEFORE OR EARLY IN THE TRIAL**	
Whether the DMC will have input into the protocol	All potential DMC members should read the protocol/outline before agreeing to join the committee. Before recruitment begins the trial will have undergone review by the funders (UK Medical Research Council and Canadian Institutes of Health Research), the sponsors (University of Leicester and RI-MUHC), and other relevant trial committees including that of research ethics committees. Therefore, if a potential DMC member has major reservations about the trial (e.g., the protocol or the logistics) they should report these to the Chief Investigators and may decide not to accept their invitation to join. DMC members should be constructively critical of the ongoing trial, but also supportive of aims and methods of the trial.
Whether the DMC will meet before the start of the trial	It is recommended that, if possible, the DMC meets before the trial starts or early in the course of the trial, to discuss the protocol, the trial, any analysis plan, future meetings, and to have the opportunity to clarify any aspects with the principal investigators.
Any issues specific to the disease under study	The study population comprises younger (18 – 40 years) adults with type 2 diabetes. These individuals are at risk of developing chronic (e.g., cardiovascular disease) or acute (e.g. hyperglycaemia) complications related to their diabetes. The protocol lists the possible adverse events of study interventions within this population.
Any specific regulatory issues	None
Any other issues specific to the treatment under study	None
Whether members of the DMC will have a contract	DMC members will not be asked to formally sign a contract but should formally register their agreement to join the group by confirming; (1) that they agree to be a member of the DMC and (2) that they agree with the contents of this Charter. Any potential competing interests, real or potential, should be declared at the same time using a short competing interest form (displayed in **Annex 1**), which should be completed and returned by the DMC members to the trial coordinating centre. DMC members should sign and maintain this log of potential competing interests.

For peer review only - http://bmjopen.bmj.com/site/about/guidelines.xhtml

RESET 4 REMISSION Trial DMC Charter

**4. COMPOSITION**	
Membership and size of the DMC	Members of the DMC (including the DMC Chair) should be independent of the trial. The definition of independent is as follows:<br>• Not part of the same institution as any of the applicants or members of the project team<br>• Not part of the same institution that is acting as a recruitment or investigative centre<br>• Not related to any of the applicants or members of the project team<br>• For the chair only – not an applicant on a rival proposal<br>The membership will consist of three individuals including two clinicians experienced in the clinical area and one statistician. Members have been chosen because they are experienced in trials and/or the disease area.<br>The members of the DMC for the RESET 4 REMISSION Trial are:<br>(1) **Dr Janusz Kaczorowski** *(Chair)*, Professor of Family and Emergency Medicine, University of Montreal<br>(2) **Mr Stephen Sharp** (*Statistician*), MRC Epidemiology Unit, University of Cambridge School of Clinical Medicine<br>(3) **Dr Andrew Farmer** (*Clinical Representative*), Professor of General Practice, Nuffield Department of Primary Care Health Sciences, University of Oxford
The Chair, how they are chosen and the Chair's role. (Likewise, if relevant, the vice-Chairman)	The chair is Dr. Janusz Kaczorowski (Professor and Research Director in the Department of Family and Emergency Medicine – University of Montreal) who is serving on the DMC. The Chair is expected to facilitate and summarise discussions. There is no vice-Chair.
The responsibilities of the DMC statistician	The DMC membership includes a statistician (Mr Stephen Sharp) whose role it is to provide independent statistical expertise and to further guide the other DMC members through the report. The DMC statistician is not expected to prepare the DMC report.
The responsibilities of the trial statistician	The trial statistician (Dr Elham Rahme) will produce (or oversee the production of) the report to the DMC and will participate in DMC meetings, guide the DMC through the report, may participate in DMC discussions and, on some occasions, taking notes.
The responsibilities of the trial office team	The trial management team (e.g., Trial Managers/Coordinators) will input to the production of the DMC report.
The responsibilities of the PI and other members of the Trial Management Group (TMG)	The PI's (Professor Thomas Yates and Professor Kaberi Dasgupta), may be asked, and should be available, to attend open sessions of the DMC meeting. The other TMG members will not usually be expected to attend but can attend open sessions when necessary (see Section 6 regarding 'Organisation of DMC Meetings' below).
**5. RELATIONSHIPS**	
Relationships with Principal Investigators, other trial committees (eg Trial Steering Committee (TSC) or Executive Committee), sponsor and regulatory bodies	The DMC are advisory to the Trial Steering Committee (which includes the PI's). The TSC is the executive body for the trial.
Clarification of whether the DMC is	The DMC is advisory to the Trial Steering Committee. The TSC is the executive

For peer review only - http://bmjopen.bmj.com/site/about/guidelines.xhtml

RESET 4 REMISSION Trial DMC Charter

advisory (make recommendations) or executive (make decisions)	body for the trial.
Payments to DMC members	Members will be reimbursed for reasonable travel costs and other expenses incurred, but it is anticipated that the meetings with be virtual. No other payments or rewards would be given to professional members.
The need for DMC members to disclose information about any competing interests	Competing interests should be disclosed. These are not restricted to financial matters – involvement in other trials or intellectual investment could be relevant. Although members may well be able to act objectively despite such connections, complete disclosure enhances credibility (See **Annex 1** towards end of DMC Charter document).<br><br>DMC members should not use interim results to inform trading in pharmaceutical shares, and careful consideration should be given to trading in stock of companies with competing products. Changes in declarations of real or potential competing interests should be documented within the minutes at the start of each meeting.
**6. ORGANISATION OF DMC MEETINGS**	
Expected frequency of DMC meetings	The DMC should meet 6-monthly to annually, or more often as appropriate either in person or via teleconference. Some trial issues may need to be dealt with between meetings, by phone or by email. DMC members should be prepared for such instances. Additional *ad hoc* meetings will be scheduled with the occurrence of any severe adverse event including death, hospitalization, or emergency room consultation. During these meetings, reports from the PIs will be reviewed, regarding the SAE in question and the relatedness to trial procedures. The DSC will then determine whether further adjudication is warranted and what type of expertise is required; in these instances, two physicians from the relevant research site will be invited to review the event and medical records and comment on relatedness. If there is a difference of opinion, a third physician will be invited to review. The physicians may be trial investigators but they must be blinded to the trial arm and cannot have interacted with the participant during the course of the trial.<br><br>Regularly-scheduled meetings should be timed approximately 1 month prior to TSC meetings, so that reports can be fed to the TSC accordingly.
Whether meetings will be face-to-face or by teleconference	Given the international context, the default will be to hold meetings over videoconference. Effort will be made to ensure that all members can attend.
How DMC meetings will be organised, especially regarding open and closed sessions, including who will be present in each session	Upon request by the DMC, the format will be an open session throughout where all parts of the report are discussed and there is DMC discussion. The DMC members can ask for a closed session at any point in the DMC (± trial statistician at discretion of the DMC chair).
**7. TRIAL DOCUMENTATION AND PROCEDURES TO ENSURE CONFIDENTIALITY AND PROPER COMMUNICATION**	
Intended content of material to be available in sessions	Accumulating information relating to recruitment and retention, and data quality (e.g., data return rates, treatment compliance) will be presented by intervention arm. No formal interim analyses are planned A log of adverse events will be presented at each meeting and discussed. Outcome data will not be presented. However, if the DMC has any concerns related to safety, pooled outcome data may be presented, and, in discussion with the TSC, unblinding may be permitted.

For peer review only - http://bmjopen.bmj.com/site/about/guidelines.xhtml

RESET 4 REMISSION Trial DMC Charter

Will the DMC be blinded to the treatment allocation	The DMC will not be blinded to recruitment data by treatment arm. No formal interim analyses are planned. The DMC will remain blinded unless safety concerns arise. However, primary and secondary outcome data will be based on pooled data, and the DMC will remain blinded unless unblinding is requested related to concerns about safety.
Who will see the accumulating data	There are no planned interim analyses. The accumulating data presented to the DMC by intervention arm will relate only to attrition and to safety. If the DMC deems that safety concerns then unblinding may occur as indicated above. Interim data and analyses by intervention/control group (and the deliberations of the DMC) should be available only to those present in the DMC meetings when the discussions occurred: i.e., only members of the DMC, the trial statistician and other members of the trial team, as agreed by the DMC. DMC members must not share confidential information with people outside the DMC.
Who will be responsible for identifying and circulating external evidence (e.g., from other trials/ systematic reviews)	Identification and circulation of external evidence (e.g., from other trials/ systematic reviews) is not the responsibility of the DMC members. It is the responsibility of the TMG (Trial Management Group). However, the DMC should continue to be made aware of other data that may impact the trial.
To whom the DMC will communicate the decisions/ recommendations that are reached	The DMC will report its recommendations via the chair in writing to the Trial Steering Committee (TSC, please see **Annex 2**). This should be sent in time for consideration at a TSC meeting. If the trial is to continue largely unchanged then it is often useful for the report from the DMC to include a summary paragraph suitable for trial promotion purposes.
Whether reports to the DMC be available before the meeting or only at/during the meeting	It is planned that the DMC will receive data monitoring reports (i.e., recruitment, attrition, numbers in various study stages, numbers completed) from the TMG at least 2 weeks before scheduled meetings.
What will happen to the confidential papers after the meeting	The DMC members should store the papers safely after each meeting so they may check the next report against them. After the trial is reported, the DMC members should destroy all interim reports.

8. DECISION MAKING

What decisions/recommendations will be open to the DMC	Possible recommendations to the TSC could include:<br>• No action needed; trial continues as planned<br>• Early stopping due, for example, to clear harm of a treatment or external evidence (this should generally involve a recommendation from the DMC to unblind the TSC to this data)<br>• Modifying target recruitment, or pre-analysis follow-up, based on any change to the assumptions underlying the original trial sample size calculation (but not on any emerging differences)<br>• Sanctioning and/or proposing protocol changes<br><br>Ultimately, decisions will be made by the TSC, as the DMC's role is advisory.
The role of formal statistical methods, specifically which methods will be used and whether they will be used as guidelines or rules	The DMC and TSC will be asked to comment on and approve the trial statistical analysis plan (SAP) before database lock.
How decisions or recommendations will be reached within the DMC	Every effort should be made to achieve consensus. The role of the DMC Chair is to summarise discussions and encourage consensus; therefore, it is usually best for the Chair to give their own opinion last.

For peer review only - http://bmjopen.bmj.com/site/about/guidelines.xhtml

RESET 4 REMISSION Trial DMC Charter

	It is important that the implications (e.g., ethical, statistical, practical, financial) for the trial be considered before any decision is made. Only appointed members will be entitled to vote and the chair will have a casting vote.
When the DMC is quorate for decision-making	Effort should be made for all DMC members to attend. The TMG will try to ensure that a date is chosen to enable this. Members who cannot attend in person should be encouraged to attend by teleconference. If, at short notice, any DMC members cannot attend at all then the DMC may still meet if at least two members will be present (including the Chair). If the DMC is considering recommending major action after such a meeting, the DMC Chair should talk with the absent members as soon after the meeting as possible to check whether they agree with such action. If they do not, a further teleconference should be arranged with the full DMC.
Can DMC members who cannot attend the meeting input	If the report is circulated before the meeting, DMC members who will not be able to attend the meeting may pass comments to the DMC Chair for consideration during the discussions.
What happens to members who do not attend meetings	If a member does not attend a meeting, it should be ensured that the member is available for the next meeting. If a member does not attend a second meeting, they should be asked if they wish to remain part of the DMC. If a member does not attend a third meeting, they should be replaced.
Whether different weight will be given to different endpoints (e.g., safety/efficacy)	This study is unlikely to result in safety issues, therefore greater weight should be given to efficacy considerations.
Any specific issues relating to the trial design that might influence the proceedings, eg cluster trials, equivalence trials, multi-arm trials	This is a 2-arm trial, occurring in the UK (Leicester) and Canada (Montreal, Edmonton). It is likely that the TSC/ DMC will need to consider issues specific to UK or Canadian sites in equivalent trials.
**9. REPORTING**	
To whom will the DMC report their recommendations/decisions, and in what form	The DMC Chair should report in writing to the Chair of the Trial Steering Committee, usually within 3 weeks after the meeting. Unless the DMC is recommending that the trial protocol be changed in some way, the letter to the TSC should not reveal any confidential information. An example of a letter from a DMC to the TSC recommending no action is presented in **Annex 2**. Additionally, the letter should be copied to the PIs, trial statistician and trial managers.
Whether minutes of the meeting be made and, if so, by whom and where they will be kept	Minutes including key points and actions will be made by a member of the trials' office team (to be decided prior to each meeting). This will include details of whether any competing interests have arisen/changed for any attendees since the previous meeting. The draft minutes will be initially circulated for comment to all DMC members who were present at the meeting. The DMC Chair will then sign off the final version of minutes. The final signed version will then be sent to all members, the sponsor, the funder and stored in the trial master file.
What will be done if there is disagreement between the DMC and the body to which it reports	The TSC has ultimate responsibility for the trial and assumes primacy. However, the TSC should report to the DMC regarding how they have acted upon the DMC's recommendations. If the DMC has serious problems or concerns with the TSC decision, a joint meeting of the TSC and DMC should be held. The information to be shown would depend upon the action proposed and each committee's concerns. The meeting would be Chaired by

For peer review only - http://bmjopen.bmj.com/site/about/guidelines.xhtml

RESET 4 REMISSION Trial DMC Charter

	a senior member of the trials office staff or an external expert who is not directly involved with the trial.
**10. AFTER THE TRIAL**	
Publication of results	The PIs have responsibility that trial results will be published in a correct and timely manner. The TSC is the committee that should oversee this process.
The information about the DMC that will be included in published trial reports	DMC members will be named and their affiliations listed in the main report, unless they explicitly request otherwise.
Whether the DMC will have the opportunity to approve publications, especially with respect to reporting of any DMC recommendation regarding termination of a trial	The DMC members should be given at least 2 weeks to read and comment on any draft publications that report the primary outcome measure and/or details of the DMC. This may be done simultaneously to other groups reviewing the draft manuscript (e.g. Trial Steering Committee, trial investigators).
Any constraints on DMC members divulging information about their deliberations after the trial has been published	The DMC should not discuss confidential issues from their involvement in the trial until 12 months after the primary trial results have been published, unless permission is agreed with the TSC. The TSC will also decide when it is appropriate for TMG and DMC members to trade in related stocks.

For peer review only

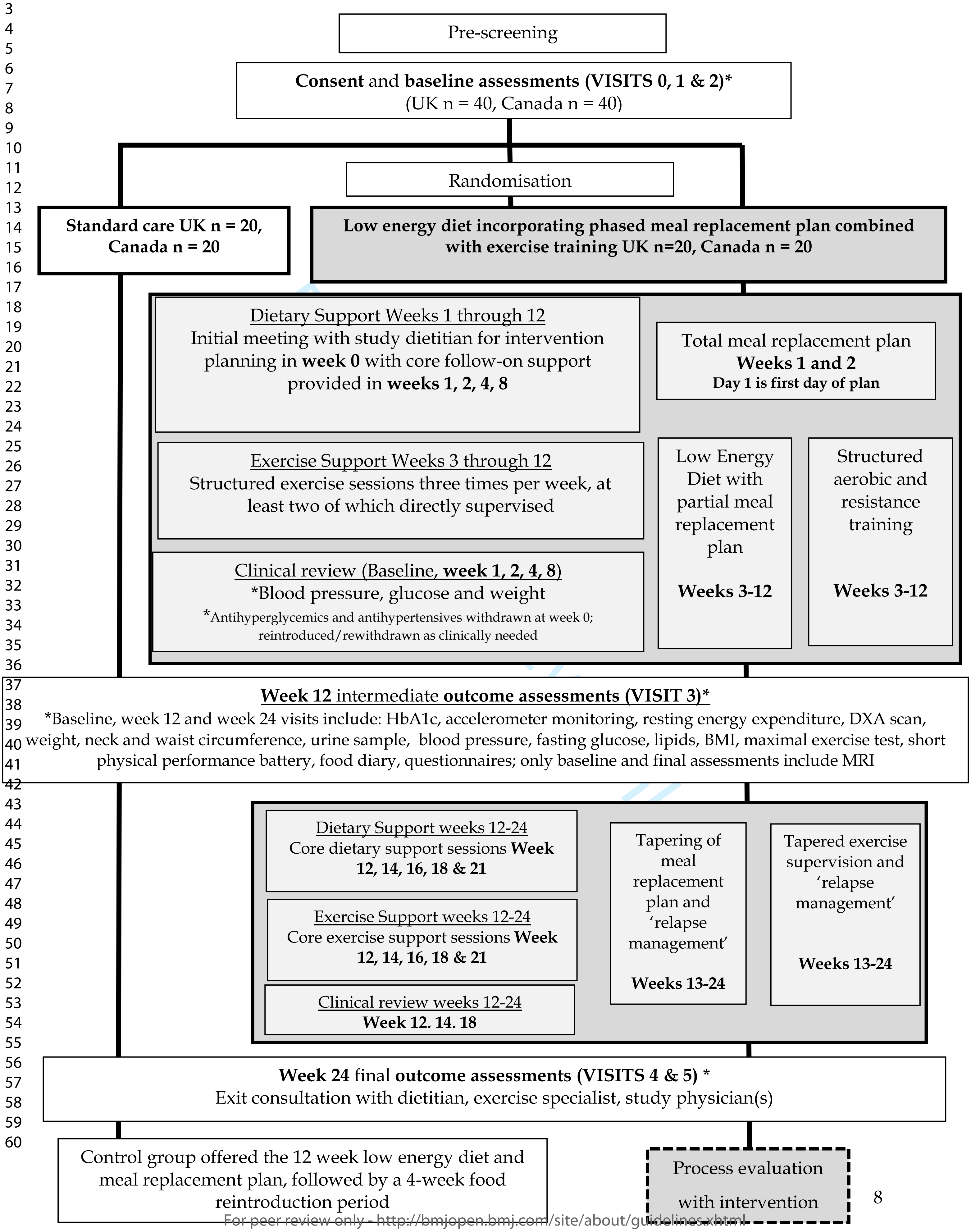

For peer review only - http://bmjopen.bmj.com/site/about/guidelines.xhtml

Annex 1: Suggested competing interests form

RESET 4 REMISSION Trial (Sponsor: University of Leicester and McGill University): Agreement to join the Data Monitoring Committee as an independent member and disclosure of potential competing interests

Please complete the following document and return to the Trials Office

(Please initial box to agree)

- ☐ I have read and understood the DMC Charter version 1.0, dated 15th March 2021
- ☐ I agree to join the Data Monitoring Committee for this trial as an independent member
- ☐ I agree to treat all sensitive trial data and discussions confidentially

The avoidance of any perception that independent members of a DMC may be biased in some fashion is important for the credibility of the decisions made by the DMC and for the integrity of the trial.

Potential competing interests should be disclosed via the trials office. In many cases simple disclosure up front should be sufficient. Otherwise, the (potential) independent DMC member should remove the conflict or stop participating in the DMC. **Table 1** lists potential competing interests.

- ☐ **No,** I have no potential competing interests to declare
- ☐ **Yes,** I have potential competing interests to declare (please detail below)

Please provide details of any potential competing interests:

____

____

____

Name: ____________________________

Signed: ___________________________ Date: ______________

Table 1: Potential competing interests for independent members

- Stock ownership in any commercial companies involved
- Stock transaction in any commercial company involved (if previously holding stock)
- Consulting arrangements with the Sponsor/Funder
- Ongoing advisory role to a company providing drugs to the trial
- Frequent speaking engagements on behalf of the intervention
- Career tied up in a product or technique assessed by trial
- Hands-on participation in the trial
- Involvement in the running of the trial
- Emotional involvement in the trial
- Intellectual conflict e.g. strong prior belief in the trial's experimental arm
- Involvement in regulatory issues relevant to the trial procedures
- Investment (financial or intellectual) or career tied up in competing products
- Involvement in the writing up of the main trial results in the form of authorship

For peer review only

Annex 2: Suggested report from DMC to TSC where no recommendations are being made

[Insert date]

To: Professor XX (Chair of Trial Steering Committee)

Dear Professor XX,

The Data Monitoring Committee (DMC) for the RESET 4 REMISSION Trial met on *[meeting date]* to review its progress and interim accumulating data. *[List members] attended* the meeting and reviewed the report.

We congratulate the trial organisers and collaborators on the progress and conduct of the trial and the presentation of the data. The trial question remains important and, on the basis of the data reviewed at this stage, we recommend continuation of the trial according to the current version of the protocol *[specify protocol version number and date]* with no changes.

We shall next review the progress and data *[provide approximate timing]*

Yours sincerely,

Professor XX
Chair of Data Monitoring Committee

On behalf of the DMC (all members listed below)

DMC members:
(1) xx
(2) xx
(3) xx

For peer review only

TSC Charter for R4R

Author: Kaberi Dasgupta and Thomas Yates

Date: 13/01/2021

Version: 1.0

RESET FOR REMISSION TRIAL TSC CHARTER

1. INTRODUCTION

Name (and sponsor's ID) of trial plus ISRCTN and/or EUDRACT number	REmission of diabetes and improved diastolic function by combining Structured Exercise with meal replacemenT and food reintroduction: THE RESET FOR REMISSION TRIAL<br>Sponsor: University of Leicester and McGill University<br>Registration number: *To be included*
Objectives of trial, including interventions being investigated	**Primary objective**<br>The aim of this efficacy study is to investigate whether combining a low energy diet incorporating the phased use of meal replacement products, with structured exercise training leads to remission of T2DM in younger adults (18-40 years) over a 24 week period. Remission is defined as an HbA1c value less than 6.5% without antihyperglycemic medications during weeks 13 through 24 weeks of the 24 week study period.<br>**Secondary objectives**<br>Key secondary objectives will examine the effects of the combined intervention on cardiovascular and functional health, particularly MRI-assessed diastolic function and cardiometabolic risk factors such as lipid profile and peripheral blood pressure, along with cardiorespiratory fitness, physical function, lean mass and basal metabolic rate. We will also undertake a process evaluation, to ensure the findings from our research can be used to inform how the interventional components are refined and translated.<br>Figure 1 shows a flow chart of the trial design.
Outline of scope of charter	The purpose of this document is to describe the membership, terms of reference, roles, responsibilities, authority, decision-making and relationships of the Trial Steering Committee (TSC) for this trial, including the timing of meetings, methods of providing information to and from the TSC, frequency and format of meetings and relationships with other trial committees.

2. ROLES AND RESPONSIBILITIES

A broad statement of the aims of the TSC	To act as the oversight body for the Reset For Remission Trial on behalf of the Sponsor/Funder.
Terms of reference	The role of the TSC is to provide oversight for the trial. It should also provide advice, through its Chair, to the CIs (PIs), the Sponsors (University of Leicester and Research Institute of the McGill University Health Centre), the Funders (The Canadian Institutes of Health Research [CIHR] and the UK Medical Research Council [MRC]), and the Host Institution on all appropriate aspects of the trial but with a particular focus on:<br>• Progress of the trial, adherence to the protocol, participant safety and the consideration of new information of relevance to the research question.

For peer review only - http://bmjopen.bmj.com/site/about/guidelines.xhtml

RESET FOR REMISSION TRIAL TSC CHARTER

	• The rights, safety and well-being of the trial participants are the most important considerations and should prevail over the interests of science and society.<br>• To ensure appropriate ethical and other approvals are obtained in line with the project plan<br>• To review proposals for substantial protocol amendments and provide advice to the sponsor and funder regarding approvals of such amendments, where appropriate.<br>• To provide advice to the investigators on all aspects of the trial.
Specific roles of TSC	The specific roles of the TSC as a body will be to:<br>• provide expert oversight of the trial<br>• make decisions as to the future continuation (or otherwise) of the trial<br>• monitor recruitment rates and encourage the Trial Management Group (TMG) to develop strategies to deal with any recruitment problems<br>• review regular reports of the trial from the trials coordinators (sent on behalf of the TMG)<br>• receive letters of feedback from the DMC (Data Monitoring Committee) and consider their recommendations<br>• assess the impact and relevance of any accumulating external evidence (provided by the TMG)<br>• monitor follow-up rates and review strategies from TMG to deal with problems<br>• ensure sites that are complying with the protocol<br>• approve major amendments to the protocol<br>• approve any proposals by the TMG concerning any change to the design of the trial, including additional sub studies<br>• oversee the timely reporting of trial results<br>• comment on the statistical analysis plan<br>• comment on the publication policy<br>• comment on the main trial manuscript<br>• approve and comment on any abstracts and presentations of any results *during* the running of the trial<br>• approve external or early internal requests for release of data or subsets of data or samples including clinical data and stored biological samples<br>It is the responsibility of individual members of the TSC to maintain confidentiality of all trial information that is not already in the public domain.
**3. BEFORE OR EARLY IN THE TRIAL**	
TSC input into the protocol	All potential independent TSC members should have opportunity to comment on the protocol as early as possible. If a potential independent TSC member has major reservations about the trial (e.g., the protocol, the logistics, ethical concerns) they should report these to the TMG and may decide not to accept the invitation to join. TSC members should be constructively critical of the protocol, but also supportive of aims and methods of the trial.
Any issues specific to the disease under study	The study population comprises younger (18 – 40 years) adults with type 2 diabetes. These individuals are at risk of developing chronic (e.g. cardiovascular disease) or acute (e.g. hyperglycaemia) complications related

For peer review only - http://bmjopen.bmj.com/site/about/guidelines.xhtml

TSC Charter for R4R

Author: Kaberi Dasgupta and Thomas Yates

Date: 13/01/2021

Version: 1.0

RESET FOR REMISSION TRIAL TSC CHARTER

	to diabetes. The protocol lists the possible adverse events of study interventions within this population
Any specific regulatory issues	None
Any other issues specific to the treatment under study	None
Whether members of the TSC will have a contract	TSC members will not be asked to formally sign a contract but should formally register their agreement to join the group by confirming (1) that they agree to be a member of the TSC and (2) that they agree with the contents of this Charter. Any potential competing interests should be declared at the same time. Members should complete and return the form in Annex 1 or 2 by email. Any observers (attendees who are not members) will sign a confidentiality agreement on the first occasion they attend a meeting (Annex 3).
**4. COMPOSITION**	
Membership and size of the TSC	Apart from the studies PIs (Dasgupta, Yates), the members of the TSC, including the Chair, should be independent of the trial. Non-independent members will also be part of the TSC. The definition of independent is as follows:<br>• Not part of the same institution as any of the applicants or members of the project team<br>• Not part of the same institution that is acting as a recruitment or investigative centre<br>• Not related to any of the applicants or members of the project team<br>• For the chair only – not an applicant on a rival proposal<br><br>The membership will consist of three individuals including two clinicians experienced in the clinical area and one statistician. Members have been chosen because they are experienced in trials and/or the disease area.<br>The members of the TSC for this trial are:<br>(1) **Professor Jason Gill** (Chair), Professor of Cardiometabolic Health, University of Glasgow<br>(2) **Dr Alice Cheng** (Clinical representative), Physician and Associate Professor of Medicine, University of Toronto<br>(3) Dr Elham Rahme (Trial statistician)<br>(4) Professor Kaberi Dasgupta and Prof Tom Yates, PIs<br><br>Only appointed members will be entitled to vote. The PIs or their delegates are allocated one vote in total.
The Chair	The chair is Prof Jason Gill (Professor of Cardiometabolic Health at the University of Glasgow) who is serving on the TSC. The Chair is expected to facilitate and summarise discussions. There is no vice-Chair.

For peer review only - http://bmjopen.bmj.com/site/about/guidelines.xhtml

RESET FOR REMISSION TRIAL TSC CHARTER

	The Chair is directly answerable to the Sponsor and Funders. The Chair's responsibilities include:<br>• Arranging, along with the PIs and trials office, an inaugural meeting to set up a schedule of meetings to align with the project plan<br>• Being familiar with relevant guidance documents and with the role of the DMC<br>• Providing an independent, experienced opinion if conflicts arise between the needs of the research team, the funder, the sponsor, the participating organisations and/or any other agencies<br>• Leading the TSC to provide regular, impartial oversight of the trial, especially to identify and pre-empt problems<br>• Ensuring that changes to the protocol are debated and endorsed by the TSC, letters of endorsement should be made available to the project team when requesting approval from the funder and sponsor for matters such as substantive changes to protocol<br>• Being available to provide independent advice as required, not just when TSC meetings are scheduled<br>• Commenting on any extension requests and, where appropriate, providing a letter of recommendation to accompany such a request<br>• Commenting in detail, when appropriate, regarding the continuation or termination of the project
The responsibilities of the trial office team	The trials office team will produce a short report on the trial before each meeting of the TSC. A template for the report will be followed.
The responsibilities of the PI and other members of the Trial Management Group (TMG)	The PIs is an important member of the TSC and no major decisions should be made without their involvement.
The responsibilities of the observers	Additional observers may be in attendance through (parts of) the TSC meetings in order to provide input on behalf of the trials office, the trial's Sponsor/Funder or to provide specific relevant expertise.
**5. RELATIONSHIPS**	
Advisory and executive bodies	The TSC is the oversight body (see *Roles and responsibilities* above). All substantial issues regarding the trial must go to the TSC for consideration. The DMC is advisory to the TSC.
Payments to TSC members	Members will be reimbursed for reasonable travel costs and other expenses incurred. No other payments or rewards would be given to professional members.
The need for TSC members to disclose information about any competing interests	Competing interests should be disclosed. These are not restricted to financial matters – involvement in other trials or intellectual investment could be relevant. Although members may well be able to act objectively despite such connections, complete disclosure enhances credibility (See Annex 1).<br>TSC members should not use interim results to inform trading in pharmaceutical shares, and careful consideration should be given to trading

For peer review only - http://bmjopen.bmj.com/site/about/guidelines.xhtml

TSC Charter for R4R

Author: Kaberi Dasgupta and Thomas Yates

Date: 13/01/2021

Version: 1.0

RESET FOR REMISSION TRIAL TSC CHARTER

	in stock of companies with competing products. Changes in declarations of real or potential competing interests should be minuted at the start of each meeting.
**6. ORGANISATION OF TSC MEETINGS**	
Expected frequency of TSC meetings	The TSC will meet at 6-monthly to annually depending on need as determined by the Chair, either in person or via teleconference. At the request of the TSC, interim meetings, in person or by teleconference, will be organised. Some trial issues may need to be dealt with between meetings, by phone or by email. TSC members should be prepared for such instances.<br>It is expected that the TSC will meet approximately one month after the DMC.
Attendance of TSC members at meetings	Given the international context, the default will be to hold meetings over videoconference. Effort will be made to ensure that all members can attend. At least one PI must attend all meeting. If, at short notice, any TSC members cannot attend then the TSC may still meet if at least two independent members will be present, along with a PI. There may be occasions when the Trial Sponsors or Funders will wish to organise and administer these meetings.
How TSC meetings will be organised, especially regarding open and closed sessions, including who will be present in each session	Presence will be usually limited to the TSC members, and observers from the TMG. Other attendees may be invited for all or part of the meeting by the TSC Chair. Meeting will be organised by Trials office, in coordination with the PIs and Chair.
Input from TSC members who cannot attend the meeting	If the report is circulated before the meeting, TSC members who will not be able to attend the meeting may pass comments to the TSC Chair or trials office contact for consideration during the discussions.
What happens to independent members who do not attend meetings	If an independent member does not attend a meeting or provide comments when requested between meetings, it should be ensured that the independent member is available for the next meeting. If an independent member does not attend the next meeting or provide comments when next requested, they should be asked if they wish to remain part of the TSC. If an independent member does not attend a third meeting, strong consideration should be given to replacing this member.
**7. TRIAL DOCUMENTATION AND PROCEDURES TO ENSURE CONFIDENTIALITY AND PROPER COMMUNICATION**	
Intended content of material to be considered during meetings	A short report will be prepared by the trial coordinators following a standard template. This will report on accrual and any matters affecting the trial. Additionally, the material may include a report *from* the DMC, requests *from* the TMG or draft publications. No trial outcome measure data will be presented by arm unless explicitly authorised by the DMC (e.g., safety outcomes). If specifically requested by the TSC, accrual, compliance with follow-up and adherence to treatment may be presented by site.
Whether reports to the TSC will be available before the meeting or only at/during the meeting	It is planned that the TSC will receive the report at least 1 week and preferably at least 2 weeks before any meetings.
Responsibility for identifying and	Identification and circulation of external evidence (e.g., from other trials/

For peer review only - http://bmjopen.bmj.com/site/about/guidelines.xhtml

TSC Charter for R4R
Author: Kaberi Dasgupta and Thomas Yates
Date: 13/01/2021
Version: 1.0

RESET FOR REMISSION TRIAL TSC CHARTER

circulating external evidence (e.g. from other trials/ systematic reviews)	systematic reviews) is not the responsibility of the TSC members; it is a responsibility of the TMG. However, the TSC should continue to be made aware of other data that may impact on a trial.
What will happen to the papers after the meeting	TSC members will be expected to securely store copies of the reports to and from the TSC, agenda and minutes, as well as copies of communications between meetings so they may check the next report against them. All documentation should be considered confidential. After the trial is reported, the TSC members should destroy all interim reports. The trials office will keep a central record of all minutes, reports and correspondence by the TSC.
**8. DECISION MAKING**	
What decisions will be open to the TSC	Based on recommendations from the DMC, possible decisions include:-<br>• No action needed; trial continues as planned.<br>• Early stopping due, for example, to clear harm of a treatment or external evidence (this should generally involve a recommendation from the DMC to unblind the TSC to this data).<br>• Modifying target recruitment, or pre-analysis follow-up, based on any change to the assumptions underlying the original trial sample size calculation (but not on any emerging differences).<br>• Sanctioning and/or proposing protocol changes.<br><br>Based on other factors, possible decisions include the decisions above and:<br>• Censuring centres for poor data quality.<br>• Approving proposed protocol amendments or new trial sub-studies.<br>• Approving requests for early release of (subsets of) data.<br>• Approving presentation of results during the trial or soon after closure.<br>• Approval of new centres or strategies to improve recruitment or follow-up.
The role of formal statistical methods	The TSC will be asked to comment on and approve trial statistical analysis plan (SAP) before database lock.
How decisions or recommendations will be reached within the TSC	Every effort should be made to achieve consensus. The role of the Chair is to summarise discussions and encourage consensus; therefore, it is usually best for the Chair to give their own opinion last.<br>It is important that the implications (e.g., ethical, statistical, practical, financial) for the trial be considered before any decision is made.
When the TSC is quorate for decision-making	At least two independent members of the TSC should be present including the Chair, along with at last one PI
Any specific issues relating to the trial design that might influence the proceedings, eg cluster trials, equivalence trials, multi-arm trials	This is a 2-arm trial, occurring in the UK (Leicester) and Canada (Montreal, Edmonton). It is likely that the TSC will need to consider issues specific to UK or Canadian sites
**9. REPORTING**	
To whom will the TSC report their recommendations/decisions, and in	Reports will be returned to the TMG who will be responsible for implementing any actions resulting. The TSC may also provide feedback to

For peer review only - http://bmjopen.bmj.com/site/about/guidelines.xhtml

TSC Charter for R4R

Author: Kaberi Dasgupta and Thomas Yates

Date: 13/01/2021

Version: 1.0

RESET FOR REMISSION TRIAL TSC CHARTER	
what form	the DMC and, where appropriate, to the Sponsors or Funders.
Whether minutes of the meeting be made and, if so, by whom and where they will be kept	Notes of key points and actions will be made by a member of the trial management team. This will include details of whether potential competing interests have changed for any attendees since the previous meeting. The draft minutes will be initially circulated for comment to those TSC members who were present at the meeting. The TSC Chair will sign off the final version of minutes or notes. The final version will then be sent to all members, the sponsor, the funder and the trial master file.
What will be done if there is disagreement between the TSC and other trial committees	The TSC is the oversight body for the trial. However, the TSC should have good reason before deciding not to accept requests from the TMG and recommendations from the DMC. If there are serious problems or concerns with the TSC decision following a DMC recommendation, a joint meeting of the TSC and DMC should be held. The information to be shown would depend upon the action proposed and each committee's concerns. The meeting would be Chaired by a senior member of the trial management staff or an external expert who is not directly involved with the trial.
**10. AFTER THE TRIAL**	
Publication of results	The TSC will oversee the timely analysis, writing up and publication of the main trial results. The independent members of the TSC will have the opportunity to read and comment on the proposed main publications of trial data prior to submission and abstracts and presentations during the trial. This review may be concurrent to that of the trial investigators and DMC.
The information about the TSC that will be included in published trial reports	TSC members will be named and their affiliations listed in the main report, unless they explicitly request otherwise.
Any constraints on TSC members divulging information about their deliberations after the trial has been published	The TSC may discuss issues from their involvement in the trial 12 months after the primary trial results have been published, or sooner if permission is agreed with the other trial committees and trials office. Similarly, the TSC will decide when it is appropriate for TMG and DMC members to trade in related stocks.

For peer review only

TSC Charter for R4R

Author: Kaberi Dasgupta and Thomas Yates

Date: 13/01/2021

Version: 1.0

Figure 1: Trial Design

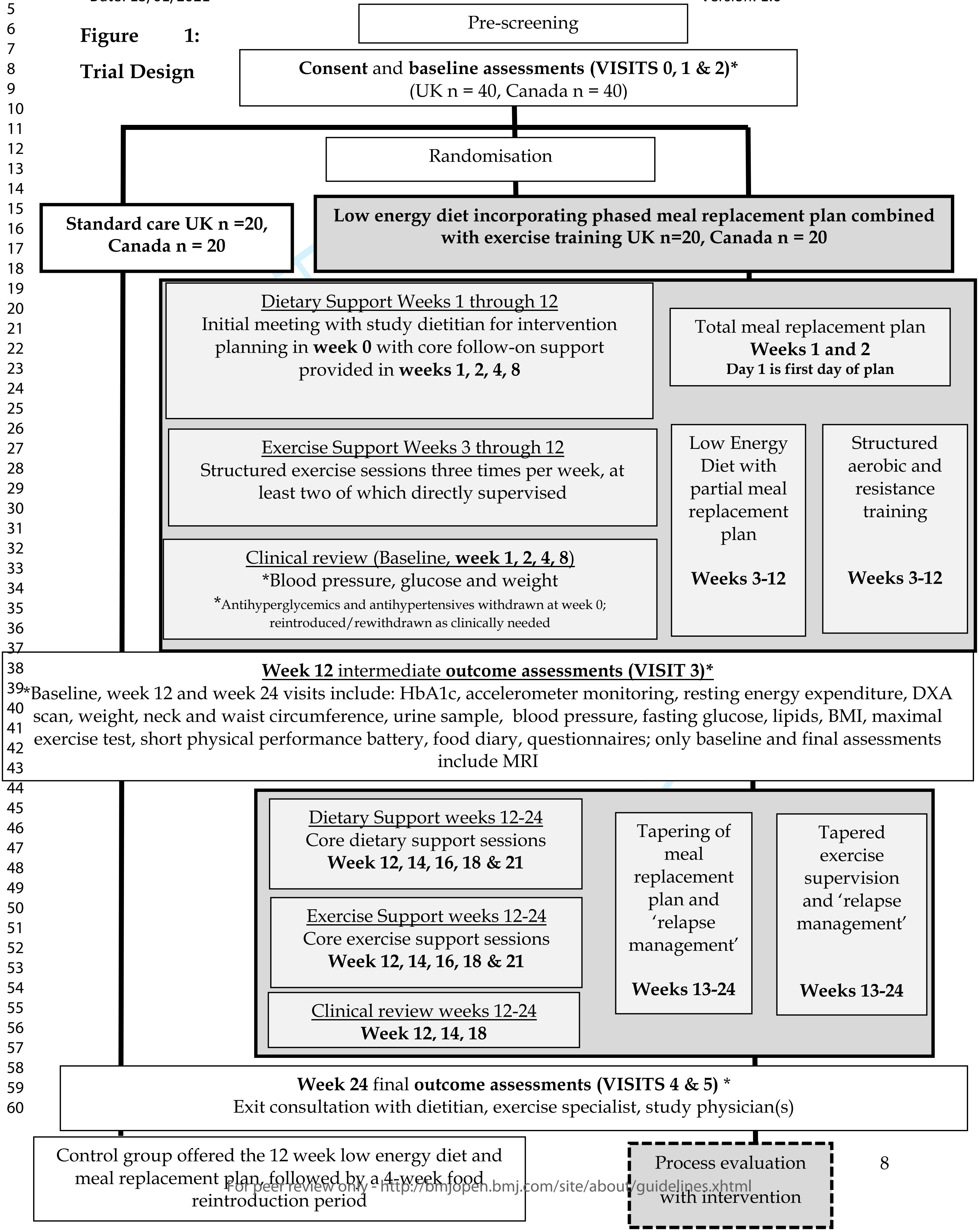

For peer review only - http://bmjopen.bmj.com/site/about/guidelines.xhtml

TSC Charter for R4R Author: Kaberi Dasgupta and Thomas Yates

Date: 13/01/2020 Version: 1.0

Annex 1: Agreement and competing interests form for independent members

RESET FOR REMISSION TRIAL: Agreement to join the Trial Steering Committee as an independent member and disclosure of potential competing interests

Please complete the following document and return to the Trials Office

(Please initial box to agree)

☐	I have read and understood the TSC Charter version 1.0, dated (will insert date pending REB approval)
☐	I agree to join the Trial Steering Committee for this trial as an independent member
☐	I agree to treat all sensitive trial data and discussions confidentially

The avoidance of any perception that independent members of a TSC may be biased in some fashion is important for the credibility of the decisions made by the TSC and for the integrity of the trial.

Potential competing interests should be disclosed via the trials office. In many cases simple disclosure up front should be sufficient. Otherwise, the (potential) independent TSC member should remove the conflict or stop participating in the TSC. **Table 1** lists potential competing interests.

☐ **No,** I have no potential competing interests to declare
☐ **Yes,** I have potential competing interests to declare (please detail below)

Please provide details of any potential competing interests:

____
____
____

Name: ______________________________

Signed: _____________________________ Date: _______________

Table 1: Potential competing interests for independent members

- Stock ownership in any commercial companies involved
- Stock transaction in any commercial company involved (if previously holding stock)
- Consulting arrangements with the Sponsor/Funder
- Ongoing advisory role to a company providing products to the trial
- Frequent speaking engagements on behalf of the intervention
- Career tied up in a product or technique assessed by trial
- Hands-on participation in the trial
- Involvement in the running of the trial
- Emotional involvement in the trial
- Intellectual conflict e.g., strong prior belief in the trial's experimental arm
- Involvement in regulatory issues relevant to the trial procedures
- Investment (financial or intellectual) or career tied up in competing products

For peer review only

- Involvement in the writing up of the main trial results in the form of authorship

Annex 2: Agreement and competing interests form for non-independent members

RESET FOR REMISSION TRIAL: Agreement to join the Trial Steering Committee as a non-independent member and disclosure of potential competing interests

Please complete the following document and return to the Trials Office.

(Please initial box to agree)

☐ I have read and understood the TSC Charter version 1.0, dated 24th April 2013

☐ I agree to join the Trial Steering Committee for this trial as a non-independent member

☐ I agree to treat all sensitive trial data and discussions confidentially

The avoidance of any perception that members of a TSC may be biased in some undisclosed fashion is important for the credibility of the decisions made by the TSC and for the integrity of the trial.

Possible competing interests should be disclosed via the trials office. In many cases simple disclosure up front should be sufficient. Otherwise, the (potential) independent TSC member should remove the conflict or stop participating in the TSC. **Table 1** lists potential competing interests.

☐ **No,** I have no competing interests to declare other than involvement in the trial

☐ **Yes,** I have competing interests to declare (please detail below)

Please provide details of any competing interests:

____

____

____

Name: ________________________________

Signed: ________________________________ Date: ________________

Table 1: Potential competing interests for non-independent members

- Stock ownership in any commercial companies involved
- Stock transaction in any commercial company involved (if previously holding stock)
- Consulting arrangements with the Sponsor/Funder
- Ongoing advisory role to a company providing drugs to the trial
- Frequent speaking engagements on behalf of the intervention
- Intellectual conflict e.g., strong prior belief in the trial's experimental arm
- Involvement in regulatory issues relevant to the trial procedures

For peer review only

- Investment (financial or intellectual) in competing products

Annex 3: Agreement and confidentiality agreement for observers

<u>RESET FOR REMISSION TRIAL:</u> Agreement to attend the Trial Steering Committee and treat all information confidentially

Please complete the following document and return to the Trials Office.

(Please initial box to agree)

☐	I have received a copy of the TSC Charter version 1.0 dated 24th April 2013
☐	I agree to attend the Trial Steering Committee meeting on ___/____/______
☐	I agree to treat as confidential any sensitive information gained during this meeting unless explicitly permitted

Name: ______________________________

Signed: _____________________________ Date: _______________

For peer review only

Annex 4: Document History

Version	Date	Who	Comments
1.0	24 April 2013	DM	First draft

For peer review only

STANDARD PROTOCOL ITEMS: RECOMMENDATIONS FOR INTERVENTIONAL TRIALS

SPIRIT 2013 Checklist: Recommended items to address in a clinical trial protocol and related documents*

**Section/item**	**Item No**	**Description**	**Page Number that item is reported**
**Administrative information**			
Title	1	Descriptive title identifying the study design, population, interventions, and, if applicable, trial acronym	**1**
Trial registration	2a	Trial identifier and registry name. If not yet registered, name of intended registry	**4**
	2b	All items from the World Health Organization Trial Registration Data Set	**4**
Protocol version	3	Date and version identifier	**4**
Funding	4	Sources and types of financial, material, and other support	**29**
Roles and responsibilitie s	5a	Names, affiliations, and roles of protocol contributors	**1 and 29**
	5b	Name and contact information for the trial sponsor	**25**
	5c	Role of study sponsor and funders, if any, in study design; collection, management, analysis, and interpretation of data; writing of the report; and the decision to submit the report for publication, including whether they will have ultimate authority over any of these activities	**29**
	5d	Composition, roles, and responsibilities of the coordinating centre, steering committee, endpoint adjudication committee, data management team, and other individuals or groups overseeing the trial, if applicable (see Item 21a for data monitoring committee)	**25-26**<br><br>**DMC and TSC appendix**

For peer review only - http://bmjopen.bmj.com/site/about/guidelines.xhtml

**Introduction**			
Background and rationale	6a	Description of research question and justification for undertaking the trial, including summary of relevant studies (published and unpublished) examining benefits and harms for each intervention	**5-6**
	6b	Explanation for choice of comparators	**5-6**
Objectives	7	Specific objectives or hypotheses	**6-7**
Trial design	8	Description of trial design including type of trial (eg, parallel group, crossover, factorial, single group), allocation ratio, and framework (eg, superiority, equivalence, noninferiority, exploratory)	**9**
**Methods: Participants, interventions, and outcomes**			
Study setting	9	Description of study settings (eg, community clinic, academic hospital) and list of countries where data will be collected. Reference to where list of study sites can be obtained	**9-10**
Eligibility criteria	10	Inclusion and exclusion criteria for participants. If applicable, eligibility criteria for study centres and individuals who will perform the interventions (eg, surgeons, psychotherapists)	**10-12 Table 2**
Interventions	11a	Interventions for each group with sufficient detail to allow replication, including how and when they will be administered	**16-20**
	11b	Criteria for discontinuing or modifying allocated interventions for a given trial participant (eg, drug dose change in response to harms, participant request, or improving/worsening disease)	**20-21**
	11c	Strategies to improve adherence to intervention protocols, and any procedures for monitoring adherence (eg, drug tablet return, laboratory tests)	**24**
	11d	Relevant concomitant care and interventions that are permitted or prohibited during the trial	**16-20**
Outcomes	12	Primary, secondary, and other outcomes, including the specific measurement variable (eg, systolic blood pressure), analysis metric (eg, change from baseline, final value, time to event), method of aggregation (eg, median, proportion), and time point for each outcome. Explanation of the clinical relevance of chosen efficacy and harm outcomes is strongly recommended	**6-9** Table 1<br><br>**13-16** (table summary)

For peer review only - http://bmjopen.bmj.com/site/about/guidelines.xhtml

Participant timeline	13	Time schedule of enrolment, interventions (including any run-ins and washouts), assessments, and visits for participants. A schematic diagram is highly recommended (see Figure)	**13** (Figure 1)
Sample size	14	Estimated number of participants needed to achieve study objectives and how it was determined, including clinical and statistical assumptions supporting any sample size calculations	**21 - 22**
Recruitment	15	Strategies for achieving adequate participant enrolment to reach target sample size	**13 and 24**
**Methods: Assignment of interventions (for controlled trials)**			
Allocation:			
Sequence generation	16a	Method of generating the allocation sequence (eg, computer-generated random numbers), and list of any factors for stratification. To reduce predictability of a random sequence, details of any planned restriction (eg, blocking) should be provided in a separate document that is unavailable to those who enrol participants or assign interventions	**16**
Allocation concealment mechanism	16b	Mechanism of implementing the allocation sequence (eg, central telephone; sequentially numbered, opaque, sealed envelopes), describing any steps to conceal the sequence until interventions are assigned	**16**
Implementation	16c	Who will generate the allocation sequence, who will enrol participants, and who will assign participants to interventions	**16**
Blinding (masking)	17a	Who will be blinded after assignment to interventions (eg, trial participants, care providers, outcome assessors, data analysts), and how	**16**
	17b	If blinded, circumstances under which unblinding is permissible, and procedure for revealing a participant's allocated intervention during the trial	**N/A**

For peer review only

**Methods: Data collection, management, and analysis**			
Data collection methods	18a	Plans for assessment and collection of outcome, baseline, and other trial data, including any related processes to promote data quality (eg, duplicate measurements, training of assessors) and a description of study instruments (eg, questionnaires, laboratory tests) along with their reliability and validity, if known. Reference to where data collection forms can be found, if not in the protocol	**13-16**
	18b	Plans to promote participant retention and complete follow-up, including list of any outcome data to be collected for participants who discontinue or deviate from intervention protocols	**21,22 and 24**
Data management	19	Plans for data entry, coding, security, and storage, including any related processes to promote data quality (eg, double data entry; range checks for data values). Reference to where details of data management procedures can be found, if not in the protocol	**23**
Statistical methods	20a	Statistical methods for analysing primary and secondary outcomes. Reference to where other details of the statistical analysis plan can be found, if not in the protocol	**22 - 23**
	20b	Methods for any additional analyses (eg, subgroup and adjusted analyses)	**22 – 23**
	20c	Definition of analysis population relating to protocol non-adherence (eg, as randomised analysis), and any statistical methods to handle missing data (eg, multiple imputation)	**22 - 23**
**Methods: Monitoring**			
Data monitoring	21a	Composition of data monitoring committee (DMC); summary of its role and reporting structure; statement of whether it is independent from the sponsor and competing interests; and reference to where further details about its charter can be found, if not in the protocol. Alternatively, an explanation of why a DMC is not needed	**26**<br>**DMC appendix**
	21b	Description of any interim analyses and stopping guidelines, including who will have access to these interim results and make the final decision to terminate the trial	**DMC appendix**

For peer review only

Harms	22	Plans for collecting, assessing, reporting, and managing solicited and spontaneously reported adverse events and other unintended effects of trial interventions or trial conduct	**20-21**
Auditing	23	Frequency and procedures for auditing trial conduct, if any, and whether the process will be independent from investigators and the sponsor	**27-28**<br>**DMC appendix**
**Ethics and dissemination**			
Research ethics approval	24	Plans for seeking research ethics committee/institutional review board (REC/IRB) approval	**3-4**
Protocol amendments	25	Plans for communicating important protocol modifications (eg, changes to eligibility criteria, outcomes, analyses) to relevant parties (eg, investigators, REC/IRBs, trial participants, trial registries, journals, regulators)	**20**<br>**DMC appendix**<br>**TSC appendix**
Consent or assent	26a	Who will obtain informed consent or assent from potential trial participants or authorised surrogates, and how (see Item 32)	**27-28**
	26b	Additional consent provisions for collection and use of participant data and biological specimens in ancillary studies, if applicable	**28**
Confidentiality	27	How personal information about potential and enrolled participants will be collected, shared, and maintained in order to protect confidentiality before, during, and after the trial	**27-28**
Declaration of interests	28	Financial and other competing interests for principal investigators for the overall trial and each study site	**28-29**
Access to data	29	Statement of who will have access to the final trial dataset, and disclosure of contractual agreements that limit such access for investigators	**27-28**<br>**TSC and DMC appendix**
Ancillary and post-trial care	30	Provisions, if any, for ancillary and post-trial care, and for compensation to those who suffer harm from trial participation	**See consent form**<br>**20**
Dissemination policy	31a	Plans for investigators and sponsor to communicate trial results to participants, healthcare professionals, the public, and other relevant groups (eg, via publication, reporting in results databases, or other data sharing arrangements), including any publication restrictions	**26**

For peer review only - http://bmjopen.bmj.com/site/about/guidelines.xhtml

	31b	Authorship eligibility guidelines and any intended use of professional writers	**N/A**
	31c	Plans, if any, for granting public access to the full protocol, participant-level dataset, and statistical code	**27**
**Appendices**			
Informed consent materials	32	Model consent form and other related documentation given to participants and authorised surrogates	**See appendix**
Biological specimens	33	Plans for collection, laboratory evaluation, and storage of biological specimens for genetic or molecular analysis in the current trial and for future use in ancillary studies, if applicable	**N/A**

*It is strongly recommended that this checklist be read in conjunction with the SPIRIT 2013 Explanation & Elaboration for important clarification on the items. Amendments to the protocol should be tracked and dated. The SPIRIT checklist is copyrighted by the SPIRIT Group under the Creative Commons "Attribution-NonCommercial-NoDerivs 3.0 Unported" license.

For peer review only

BMJ Open

Remission of Type 2 Diabetes and Improved Diastolic Function by Combining Structured Exercise with Meal Replacement and Food Reintroduction among young adults: The RESET FOR REMISSION Randomized Controlled Trial Protocol

Journal:	*BMJ Open*
Manuscript ID	bmjopen-2022-063888.R1
Article Type:	Protocol
Date Submitted by the Author:	29-Aug-2022

Complete List of Authors:	Dasgupta, Kaberi; Research Institute of the McGill University Health Centre, Department of Medicine, Faculty of Medicine<br>Boulé , Normand ; University of Alberta<br>Henson, Joseph; NIHR Leicester Biomedical Research Centre, Diabetes Research Centre<br>Chevalier, Stéphanie; McGill University, School of Human Nutrition<br>Redman, Emma; Leicester General Hospital<br>Chan, Deborah; Research Institute of the McGill University Health Centre<br>McCarthy, Matthew; Leicester General Hospital<br>Champagne, Julia; Research Institute of the McGill University Health Centre<br>Arsenyadis, Frank; Leicester General Hospital<br>Rees, Jordan; University of Alberta Faculty of Kinesiology Sport and Recreation<br>Da Costa, Deborah; Research Institute of the McGill University Health Centre<br>Gregg, Edward; Imperial College London<br>Yeung, Roseanne; University of Alberta<br>Hadjiconstantinou, Michelle; University of Leicester<br>Dattani, Abhishek; University of Leicester Department of Cardiovascular Sciences<br>Friedrich, Matthias G.; Courtois Cardiovascular Signature Centre, McGill University Health Centre<br>Khunti, Kamlesh; University of Leicester, Diabetes Research Centre<br>Rahme, Elham; McGill University Health Centre, Division of Clinical Epidemiology<br>Fortier, Isabel; Research Institute of the McGill University Health Centre<br>Prado, CM; University of Alberta<br>Sherman, Mark; Leicester Real World Evidence Unit, University of Leicester<br>Thompson, Richard ; University of Alberta, Department of Biomedical Engineering<br>Davies, Melanie; University of Leicester, Diabetes Research Centre<br>McCann, Gerry; University of Leicester Department of Cardiovascular Sciences,<br>Yates, Thomas; University of Leicester Diabetes Research Centre

**Primary Subject Heading**:	Diabetes and endocrinology
Secondary Subject Heading:	Cardiovascular medicine
Keywords:	DIABETES & ENDOCRINOLOGY, CARDIOLOGY, NUTRITION & DIETETICS

SCHOLARONE™
Manuscripts

Title: Remission of Type 2 Diabetes and Improved Diastolic Function by Combining Structured Exercise with Meal Replacement and Food Reintroduction among young adults: The RESET FOR REMISSION Randomized Controlled Trial Protocol

Authors:

Kaberi Dasgupta*[1], Normand Boulé[2], Joseph Henson[3], Stéphanie Chevalier[4], Emma Redman[3], Deborah Chan[1], Matthew McCarthy[3], Julia Champagne[1], Frank Arsenyadis[3], Jordan Rees[2], Deborah Da Costa[1], Edward Gregg[5], Roseanne O. Yeung[6], Michelle Hadjiconstantinou[3], Abhishek Dattani[7], Matthias G. Friedrich[8], Kamlesh Khunti[9], Elham Rahme[1], Isabel Fortier[1], Carla M. Prado[10], Mark Sherman[9], Richard Thompson[11], Melanie Davies[3], Gerry P. McCann[7], Thomas Yates*[3]

*Dr Dasgupta and Dr Yates are the Co-Lead investigators and Co-corresponding authors. They contributed equally to this work.

[1] Centre for Outcomes Research and Evaluation, Research Institute of the McGill University Health Centre, 5252 Maisonneuve West Boulevard, Montreal, Quebec, Canada H4A 3S5; Department of Medicine, McGill University

[2] Faculty of Kinesiology, Sport, and Recreation 1-052 Li Ka Shing Centre for Health Research Innovation, University of Alberta, Edmonton, Alberta, Canada T6G 2H9

[3] Diabetes Research Centre, University of Leicester, Leicester General Hospital, Gwendolen Road, Leicester, United Kingdom LE5 4PW; NIHR Leicester Biomedical Research Centre, University Hospitals of Leicester NHS Trust and University of Leicester, Leicester, United Kingdom

For peer review only

[4]School of Human Nutrition, Macdonald Campus of McGill University, 21111 Lakeshore Road, Sainte-Anne-de-Bellevue, Quebec, Canada H9X 3V9

[5] School of Public Health, Imperial College London, Medical School Building, St Mary's Hospital, Norfolk Place, London, United Kingdom W2 1PG

[6] Division of Endocrinology & Metabolism, Department of Medicine, 9114- Clinical Sciences Building 11350 - 83 Avenue, Edmonton, Alberta, Canada T6G 2G3

[7]Department of Cardiovascular Sciences University of Leicester and the NIHR Leicester Biomedical Research Centre, Glenfield Hospital NHS Trust, Groby Rd, Leicester, United Kingdom LE3 9QP

[8] Courtois Cardiovascular Signature Centre, McGill University Health Centre, 1001 Decarie Boulevard, Montreal, Quebec, Canada H4A 3J1; Departments of Medicine and Diagnostic Radiology, McGill University

[9] Leicester Real World Evidence Unit, University of Leicester, Leicester General Hospital, Gwendolen Road, Leicester, United Kingdom LE5 4PW

[10] Department of Agricultural, Food and Nutritional Science, University of Alberta, Edmonton, Alberta, Canada T6G 2V2

[11] Department of Biomedical Engineering, 1098 Research Transition Facility, University of Alberta, Edmonton, Alberta, Canada T6G 2V2

Word Count: 4473 words

For peer review only - http://bmjopen.bmj.com/site/about/guidelines.xhtml

ABSTRACT

Introduction

Type 2 diabetes (T2DM) onset before 40 years of age has a magnified lifetime risk of cardiovascular disease. Diastolic dysfunction is its earliest cardiac manifestation. Low energy diets incorporating meal replacement products can induce diabetes remission, but do not lead to improved diastolic function, unlike supervised exercise interventions. We are examining the impact of a combined low energy diet and supervised exercise intervention on T2DM remission, with peak early diastolic strain rate, a sensitive Magnetic Resonance Imaging (MRI)-based measure, as a key secondary outcome.

Methods and Analysis

This prospective, randomized, two-arm, open-label, blinded-endpoint efficacy trial is being conducted in Montreal, Edmonton, and Leicester. We are enrolling 100 persons 18 to 40 years of age within 6 years' T2DM diagnosis, not on insulin therapy, and with obesity. During the intensive phase (12 weeks), active intervention participants adopt an 800 to 900 kcal/day low energy diet combining meal replacement products with some food, and receive supervised exercise training (aerobic and resistance), three times weekly. The maintenance phase (12 weeks) focuses on sustaining any weight loss and exercise practices achieved during the intensive phase; products and exercise supervision are tapered but reinstituted, as applicable, with weight regain and/or exercise reduction. The control arm receives standard care. The primary outcome is T2DM remission, (hemoglobin HbA1c of less than 6.5% at 24 weeks, without use of glucose lowering medications during maintenance.) Analyses will be by intention-to-treat with stratified Fisher's exact test statistics.

Ethics and Dissemination The trial is approved in Leicester (East Midlands – Nottingham Research Ethics Committee [21/EM/0026]), Montreal (McGill University Health Centre

Research Ethics Board [RESET for remission / 2021-7148]), and Edmonton (University of Alberta Health Research Ethics Board (Pro00101088). Findings will be shared widely (publications, presentations, press releases, social media platforms) and will inform an effectiveness trial.

Trial registration: ISRCTN15487120 https://doi.org/10.1186/ISRCTN15487120. Registered in accordance with World Health Organisation Trial Registration Data Set (version 1.3.1). Date assigned June 22, 2021.

Key words: diabetes reversal, early-onset diabetes, heart failure, intensive lifestyle intervention, health behaviour change, obesity, weight loss, fitness, type 2 diabetes

For peer review only

Strengths and Limitations

- RESET will evaluate diabetes remission alongside MRI-assessed diastolic function, an early indicator of the adverse impacts of type 2 diabetes, following a low energy diet and supervised exercise
- RESET will not distinguish the impacts of the individual dietary and exercise components
- We are focusing on young adults with type 2 diabetes, a group at elevated risk for diabetes complications
- Recruitment will be challenging due to competing responsibilities and lower numbers of young persons with type 2 diabetes compared to older age groups

BACKGROUND

The global prevalence of diabetes over 9% and is expect to surpass 10% by 2030. (1) More than 90% is type 2 diabetes (T2DM).Diabetes is diagnosed in over 8% of Canadians and 6%

For peer review only - http://bmjopen.bmj.com/site/about/guidelines.xhtml

of persons in the UK. More than 90% is type 2 diabetes (T2DM). The Da Qing type 2 diabetes prevention trial's 30-year follow-up demonstrated each year without T2DM translates into fewer cardiovascular disease (CVD) events (2). T2DM remission is emerging as a potential goal in early-onset disease, defined as an A1C below diagnostic thresholds without glucose lowering medications.

Health behaviour-based remission trials to date have focused on meal-replacement facilitated lowering of energy intake combined with exercise counselling (3-6). The Look AHEAD (Action for Health in Diabetes) trial implemented a 1200 to 1800 kcal diet and an exercise target of 175 minutes/week. Its primary aim was reducing T2DM-related complications; remission, a secondary outcome, was 11.5% at 1 year and 7.3% at 4 years (7); increased fitness predicted T2DM remission (7). The DiRECT trial (Diabetes REmission Clinical Trial) (8-10) focussed on remission within 6 years of a T2DM diagnosis through an 800-kcal meal replacement diet for 3 to 5 months followed by a food-based weight maintenance diet. Participants received a step counter and advised to increase their steps. Remission was 46% remission at 1 year (9) and over 30% at 2 years (10). The DIADEM-I trial (Diabetes Intervention Accentuating Diet and Enhancing Metabolism) also focussed on remission, among adults within 3 years of diagnosis through a dietary strategy similar to DiRECT but more regular exercise counselling, incorporating both time and step goals as well as a goal of resistance training twice per week. DIADEM-I achieved over 60% remission at 1 year (11).

Our RESET for REMISSION trial combines a meal replacement-facilitated dietary intervention with a *supervised* exercise intervention. Such interventions in T2DM demonstrated greater glycemic lowering than unsupervised counselling-based approaches, (12) with A1C lowering independent of weight loss (13) and direct cardiac benefits in T2DM (14, 15). Isolated diastolic dysfunction as the earliest manifestation (16) of the concentric

For peer review only

For peer review only - http://bmjopen.bmj.com/site/about/guidelines.xhtml

remodelling that typifies diabetic heart disease (17). In the DIASTOLIC trial (18) (Diabetes Interventional Assessment of Slimming or Training tO Lessen Inconspicuous Cardiovascular Dysfunction), our UK team members compared low energy meal replacement diets against thrice weekly supervised aerobic exercise training over 12 weeks among young adults with T2DM to examine impacts on cardiovascular function, with remission as a secondary outcome. Seventy percent of those in the diet arm achieved 70% remission, without impact on diastolic function; the exercise arm had little impact on remission but improved peak early diastolic strain rate (18), a sensitive MRI-based diastolic function measure. In RESET for REMISSION, we will combine the dietary and exercise interventions and assess impacts not only on remission but also on MRI-based measures of cardiac function, another novel aspect of our trial. Moreover, in contrast to DIASTOLIC, we will combine supervised aerobic and resistance training to optimise lean mass preservation (19) with weight loss and the potential for reductions in A1C than those observed for supervised aerobic exercise alone. (19) (20) We hypothesize that this combination of a low energy meal replacement based diet and supervision for both aerobic and resistance training will have synergistic impacts on remission, enhance heart health, preserve lean mass, and improve fitness (20) (19, 21).

Diastolic dysfunction is more prevalent in younger adults with T2DM compared to weight matched and lean controls (22, 23). Compared to later-onset T2DM (24) and even type 1 diabetes, (25) young-onset T2DM has a more aggressive phenotype (26). Younger adults represent at least 16% of T2DM internationally (27). LookAHEAD studied persons 45 to 76 years of age and the average age in DiRECT was 53 years. DIADEM-I in contrast targeted adults 18 to 50 years of age. RESET for REMISSION will focus on those 18 to 45 years of age in cities in Canada and the UK. Even temporary T2DM remission could lead to fewer diabetes-related complications during what are supposed to be the most productive years. Even temporary T2DM remission could lead to fewer diabetes-related complications during what are supposed to be the most productive years.

For peer review only - http://bmjopen.bmj.com/site/about/guidelines.xhtml

AIMS

Among adults 18 to 40 years of age with obesity, within 6 years of a T2DM diagnosis, and not on insulin therapy, we will quantify the remission efficacy of a 12-week low energy diet combined with supervised exercise, followed by a 12-week phase of weight and exercise maintenance. We define T2DM remission as a haemoglobin A1c (HbA1c) value less than 6.5% (48 mmol/mol) at 24 weeks, without use of antihyperglycemic medications during the prior 12 weeks, concordant with the international consensus definition. (3-6)

Through comprehensive cardiovascular magnetic resonance (CMR), we are also assessing circumferential peak early diastolic strain rate, a sensitive measure (22) with excellent test-retest reproducibility (28); end-diastolic mass to volume ratio, a marker of concentric left ventricular remodelling (29); and measures of aortic distensibility, a key determinant of concentric remodelling in T2DM (29). We will also ascertain impacts on a range of other measures (Table 1) of insulin resistance, fitness, adiposity, cardiorenal parameters, diet, physical activity, mood, and quality of life. We will evaluate participant perspectives (Appendix 1). Our results will guide the design of a longer-term effectiveness trial.

For peer review only

Table 1. Outcomes

	Week 12	Week 24
**PRIMARY OUTCOME**		
Diabetes remission		X
**KEY SECONDARY OUTCOMES**		
Other remission, glycemic, and insulin resistance measures		
Diabetes remission	X	
Hemoglobin A1c, fasting glucose and insulin, Homeostatic Model Assessment for Insulin Resistance	X	X

For peer review only - http://bmjopen.bmj.com/site/about/guidelines.xhtml

Main CMR measures		
Left ventricular peak early diastolic strain rate (circumferential and longitudinal, MRI)		X
End-diastolic mass to volume ratio		X
Main fitness measure		
VO_2 peak	X	X
Main fat and lean mass measures		
Total fat and lean soft tissue mass (DXA)	X	X
Weight and BMI	X	X
Cardiometabolic indicators		
Hypertension remission, systolic and diastolic blood pressure, heart rate	X	X
Total cholesterol, HDL, LDL, triglycerides	X	X
**OTHER SECONDARY OUTCOMES**		
Renal function measures		
Creatinine and estimated glomerular filtration rate	X	X
Urine albumin to creatinine ratio	X	X
Hepatic function measures		
Aspartate aminotransferase, alanine aminotransferase, gamma glutamyl transferase, bilirubin	X	X
Depression, anxiety, and distress		
Hospital Anxiety and Depression Scale and Diabetes Distress Scale	X	X
Indirect calorimetry		
Resting metabolic rate	X	X
Additional cardiac and aortic MRI-based measures		

For peer review only

Longitudinal and circumferential measures of systolic strain, end systolic volume, ejection fraction, mean T1 time		X
Cross-sectional areas and distensibility of ascending and descending aortae		X
Additional measures of muscle mass and adiposity		
Neck, hip, and waist circumference	X	X
Visceral adipose tissue, pancreatic and liver fat percentages, subcutaneous adipose tissue, muscle mass (MRI)		X
Dietary variables		
Total energy and macronutrient intake (protein, carbohydrates, lipids)	X	X
Selected carbohydrate types (total sugars, starch, fibre), selected lipid types (saturated, monounsaturated, polyunsaturated, cholesterol), alcohol	X	X
Accelerometer-based physical activity measures and sleep (daily average)		
Steps, overall acceleration, and intensity gradient metric	X	X
Minutes for each of sedentary, light, and moderate to vigorous physical activity	X	X
Sleep time, duration of night, sleep efficiency (sleep time/duration of night)	X	X
Other exercise stress test measures		
VCO_2 peak, maximum gradient achieved	X	X
**Tertiary outcomes**		
Bone measures		
Total bone mineral density and bone mineral content (DXA)	X	X
Physical function		
Handgrip strength	X	X
Short Physical Performance Battery	X	X
Dyspnoea scale	X	X

For Peer review only

For peer review only - http://bmjopen.bmj.com/site/about/guidelines.xhtml

Overall health state		
EQ-5D-5L score (EuroQuol group 5-dimensional 5-level questionnaire)	X	X
WHO Disability Assessment Schedule 2.0	X	X
Process Evaluation		
Acceptability, feasibility, facilitators, barriers and adherence to the intervention will be determined through interviews		X

METHODS

Design

Prospective, randomised, two-arm, open-label, blinded-endpoint (PROBE) efficacy trial

Setting

Leicester (Leicester Diabetes Centre), United Kingdom; Montreal (Research Institute of the McGill University Health Centre- Centre for Outcomes Research and Evaluation, Canadian coordinating site), Canada; Edmonton (Alberta Diabetes Institute – University of Alberta), Canada.

Trial time frame

We recruited the first participant on September 24, 2021; we plan to complete recruitment by June 30, 2024 and interventions and final evaluations by January 31, 2025,

Eligibility Criteria

Adults with T2DM 18 to 45 years of age who were diagnosed less than 6 years previously, are not on insulin therapy, and who have a HbA1c value between 6 and 10% if taking other glucose-lowering medication or between 6.5 and 10% if not taking any glucose-lowering medications. Eligibility criteria are detailed in Table 2.

Table 2. Inclusion and exclusion criteria

For peer review only - http://bmjopen.bmj.com/site/about/guidelines.xhtml
For peer review only

Inclusion criteria	
Age	18 to 45 years, inclusive
Type 2 diabetes	Physician diagnosis more than three months and less than six years previously
Hemoglobin A1c	6.5% to 10%, inclusive if not taking glucose-lowering medication; 6 to 10% if taking glucose-lowering medication
Body mass index	• 30 kg/m^2 to 45 kg/m^2, inclusive if White or Indigenous[1]<br>• 27 kg/m^2 to 45 kg/m^2, inclusive if other background, including mixed
Weight stability	Weight changes of less than 5 kg over the prior 6 months
Walking ability	Able to walk without assists and to participate in structured exercise training requiring the lower limbs
Capacity	• Able to understand written and spoken English and/or French<br>• Able to provide informed consent
Willingness	• Willing to be randomized and able to participate<br>• Willing to attend supervised exercise sessions, if so randomized<br>• Willing to adopt low energy diet, including abstinence from alcohol, if so randomized<br>• Willing to self-monitor glucose and blood pressure at the required frequency, if randomized to the low energy diet plus supervised exercise arm
**Exclusion criteria**	
Other diabetes types	• Type 1 diabetes

For peer review only - http://bmjopen.bmj.com/site/about/guidelines.xhtml

	• Gestational diabetes<br>• Monogenic diabetes
Poorly controlled blood pressure	Resting systolic blood pressure greater than 150 mmHg or resting diastolic blood pressure greater than 90 mmHg diastolic
Weight loss interventions	• Currently participating in a weight reduction program in addition to routine care<br>• Previous bariatric surgery
Medications	• Insulin therapy[2]<br>• Use of licensed weight loss medications<br>• Significant changes in glucose lowering medications in the prior 3 months, as judged by study physicians<br>• Steroids by mouth or injection
Self-reported allergies to components of meal replacement products	Milk protein and/or other relevant allergies
Dietary practices	Dietary practices that prohibit the use of meal replacement products
Pregnancy & Lactation	• Pregnancy<br>• Lactation<br>• Planning to become pregnant in the next 8 months
Eating disorder	Self-reported or diagnosed
Substance abuse	Alcohol, drugs
Estimated glomerular filtration rate	Less than 60 $ml.min^{-1}$ per $1.73m^2$
Retinopathy	Receiving or requiring active treatment for retinopathy

For peer review only

Clinically manifest vascular disease	• Myocardial infarction<br>• Stroke<br>• Peripheral vascular disease
Other cardiac disease	• Heart failure<br>• Atrial fibrillation<br>• Pacemaker<br>• Implantable cardioverter defibrillator (ICD)
Other conditions that could impact weight and/or safety	Active malignancy or other chronic disease
Run-in phase	• Failure to complete at least 5 or requested 7 days of accelerometer wear<br>• Failure to complete a food diary for three weekdays and one weekend day

[1] Term for the original peoples of North America and their descendants; includes First Nations, Inuit, and Métis peoples

[2] An exception is made for women who are on insulin therapy in case of pregnancy occurrence because of insulin's established safety profile in pregnancy, rather than because of inability to control glycemia on oral agents alone. If these women are willing and able to use a reliable form of contraception, they may be enrolled.

RECRUITMENT

Recruitment is primarily through clinics within the collaborative networks of participating centres and publicity through social media and other forms of media.

Run-in period

For peer review only - http://bmjopen.bmj.com/site/about/guidelines.xhtml

Candidates wear an accelerometer for 7 consecutive days (wrist) and complete a 4-day food diary (three weekdays and one weekend day). Five 5 days of accelerometer wear and the 4-day food diary are required to move beyond the run-in period.

MEASUREMENTS

In-person evaluations occur before randomization, at intervention period midpoint (12 weeks +/- 1 week; no MRI study), and at intervention period end (24 weeks +/- 2 weeks) (Figure 1; Table 1).

Type 2 diabetes remission and related measures

The primary remission outcome requires a HbA1c below 6.5% at intervention period end, without glucose-lowering medication during the prior 12 weeks (3-6). Venous blood is sampled for HbA1c (high-performance liquid chromatography) measurement. Other related outcomes include HbA1c, insulin resistance (HOMA-IR), and midpoint remission.

MRI defined cardiac structure and function

Using the 3-Tesla platform (30) (Figure 2), we acquire cardiac cine images with retrospective electrocardiographic gating and an 18-channel phased-array cardiac receiver coil covering the left ventricle from base to apex (8 mm slice thickness, 2 mm gap with temporal resolution <50 ms and reconstructed to 30 phases). Using cmr42 software (CircleCardiovascular Imaging, Canada), we assess end-diastolic and end-systolic volumes, ejection fraction, myocardial mass, peak diastolic filling rate, systolic global longitudinal and circumferential strain, (simplified) long axis strain, and circumferential and longitudinal peak early diastolic strain rates (Figure 3).

We quantify aortic stiffness, at the pulmonary artery bifurcation with simultaneous blood pressure recording, using Java Image Manipulation (Xinapse Software, Essex, UK). (29) We characterize myocardial tissue using a modified look-locker inversion-recovery sequence for

For peer review only

a mid-level native T1 map. High native T1 time, and is a surrogate marker of diffuse interstitial fibrosis.

Adiposity and lean measurements

We measure weight (nearest 0.1 kg) and height, waist, hip and neck circumferences (nearest 0.1 cm) and use DXA (GE Lunar iDXA) for total fat and lean mass (Table 1). We acquire MRI images for visceral, subcutaneous, hepatic, and pancreatic adipose tissue using the chemical shift encoded (DIXON) approach. Taylor's twin cycle hypothesis (31) emphasizes hepatic fat accumulation in T2DM development, leading to insulin resistance along with pancreatic fat accumulation that impairs beta-cell function.

Optional MRI sequences

Time permitting, optional sequences include simultaneous PROton Density Fat Fraction Imaging and Water T1-Mapping with Low B1+ Sensitivity (PROFIT1) of the liver (fibrosis), thigh (skeletal muscle volume), and heart (intra-myocardial fat) (32). In Montreal, and time permitting at other sites, we perform oxygen sensitive CMR with hyperventilation and breath-holding, enabling ischaemia/microvascular dysfunction detection without contrast. (33) In Leicester, participants receive manganese contrast infusion, with repeated T1 mapping every 2.5 min for 30 min, to assess calcium handling. (34)

Resting metabolic rate, fitness, and strength

Following overnight fast, we conduct indirect calorimetry (ventilated hood system) for resting metabolic rate. We perform fixed speed treadmill exercise testing with increasing gradient (1% each minute) and a rolling average of 10 breaths for oxygen consumption and carbon dioxide production. The test continues to volitional exhaustion; 100% of age-predicted maximum heart rate (85% if using beta-blocker medication) plus Respiratory Exchange Ratio ≥ 1.15; or ECG changes or symptoms of concern. We assess peak oxygen

For peer review only - http://bmjopen.bmj.com/site/about/guidelines.xhtml

For peer review only

consumption, heart rate, and blood pressure. The peak force output of handgrip is determined across three measures in each hand. We apply the Short Physical Performance Battery (35), WHODAS 2.0 questionnaire (36) and the Medical Research Council dyspnoea scale (mMRC dyspnoea scale) (37).

Diet, physical activity, and sleep

The four-day food diary is repeated at intermediary and final evaluations for daily intakes (Table 1). In addition to run-in, participants wear an accelerometer for a 7 to 14-day period proximate to the intermediary and final assessments. Accelerometer data is captured at 30 Hz (open source R program GGIR) (38) for daily averages of steps, overall acceleration, and intensity gradient metric, and total time at sedentary, light, and moderate to vigorous intensities. Overnight wear permits capture of sleep time, night duration, and their ratio (sleep efficiency).

Cardiometabolic profile

We assess seated blood pressure (automated sphygmomanometer, averaged systolic and diastolic 5 sequential measures). We sample blood for lipid profiles, creatinine, electrolytes, and liver function tests; and urine for the albumin to creatinine ratio. Among those without RAAS inhibition for albuminuria, we assess hypertension remission at intermediary and final assessments (blood pressure values of 130/80 mm Hg without use of antihypertensive agents during prior 12 weeks.)

Demographic factors, mental well-being, and quality of life

Participants complete questionnaires on sex and gender, date of birth, ethnicity, employment, income, education, and marital status; anxiety and depression (Hospital Anxiety and Depression scale (39)); diabetes-related distress (Diabetes Distress Scale (DDS) (40)); and quality of life (EQ-5D-5L (41)).

Intervention arm continuous blood glucose monitoring

For peer review only - http://bmjopen.bmj.com/site/about/guidelines.xhtml

To quantify the pattern of blood glucose responses to the intervention in detail, continuous glucose monitoring (CGM) devices will be worn for 7 to 14 days preintervention, at intervention onset, midintervention and at the end of intervention, with computation of average glucose level , glucose variability (standard deviation (SD) and coefficient of variation (CV)) and time within, above, and below range.

Data gathering for process evaluation

Through a qualitative descriptive approach, we will explore experiences of intervention arm participants (42). We will use maximum variation sampling (43) to capture those who did and did not achieve remission and variations in age, sex, ethnicity, and location. Our individual audio recorded and transcribed in-depth interviews (Appendix 1) are informed by the Normalisation Process Theory framework (44) and COM-B (Capability, Opportunity, Motivation - Behaviour) (45). Sampling will continue until saturation of themes is achieved.(46)

RANDOMIZATION AND ALLOCATION CONCEALMENT

We randomize participants individually, stratified by country and sex, in blocks of variable size. An independent statistician developed the randomization sequences uploaded into the Research Electronic Data Capture system. A researcher blinded to the sequence unveils group allocation through an auto-generated randomization button on REDCap.

EXPERIMENTAL GROUP

Full meal replacement diet for two weeks

Dietitian review: Participants meet with the dietitian to discuss the first two weeks of the intervention which include only *Optifast* products (*Nestlé*), totalling 800 to 900 kcal/day (30% protein, 50% carbohydrate, 20% fat). To minimize constipation, participants drink at least 2 litres of calorie-free fluid each day, and receive a fibre-based laxative, to use as

For peer review only - http://bmjopen.bmj.com/site/about/guidelines.xhtml

needed. They receive a digital body weighing scale and record weight weekly. They meet with the dietitian again in weeks 1 and 2 (virtually or in-person, as preferred).

Physician review: The study physician meets with the intervention arm participant to discuss antihyperglycemic and antihypertensive medication withdrawal, (47) given immediate lowering of glucose values and blood pressure with the dietary intervention. Glucagon-like peptide 1 receptors are stopped one week prior to the low energy diet. All other antihyperglycemic medications are stopped the day that the diet is started. Except for angiotensin converting enzyme inhibitors or angiotensin receptor blockers prescribed for albuminuria (i.e., RAAS blockade), participants stop antihypertensive medications on the day that the diet is initiated.

At weeks 1 and 2 follow-up (virtual or in-person), the physician reviews participant-recorded glucose and blood pressure data. Participants perform capillary blood glucose testing daily before breakfast and, on one day prior to physician follow-up, a 7-point glucose profile (measures before and two hours after food or meal replacement consumption and at bedtime). Antihyperglycemic medications are not considered for reintroduction unless fasting morning capillary glucose is frequently 10 mmol/l or higher. Using the home blood pressure monitor provided, participants measure seated blood pressure each morning, after a 5-minute rest period (second of two sequential measures). Antihypertensive agents are not be reintroduced unless systolic blood pressure values are generally 165 mmHg or higher, as in the DiRECT trial (48). RAAS blockade may be interrupted with symptomatic hypotension.

Participants who may become pregnant are required to use reliable contraception. This is discussed, with referral as needed.

Partial meal replacement low energy diet and supervised exercise (weeks 3 to 12 inclusive)

For peer review only - http://bmjopen.bmj.com/site/about/guidelines.xhtml

Exercise physiologist: Starting at week 3, an exercise physiologist supervises two weekly sessions with aerobic and resistance components, at a designated facility. A third session is aerobic only, on site or elsewhere, as preferred, with self-monitoring (heart rate monitor and/or physical activity tracker provided). Participants increase exercise intensity and duration over time, with a goal of 60-minute sessions.

Aerobic exercise on-site includes brisk treadmill walking (60-80% of the maximum stress test heart rate). There is a high intensity interval walking option (49), to maximize improvements in fitness, insulin sensitivity (50) and lean mass preservation (51, 52). With walking challenges (e.g., plantar fasciitis), cycle ergometer may be offered.

Resistance training with machines or free weights includes one to three sets of four upper body exercises (bench press, seated row, shoulder press, and pull down), three leg exercises (leg press, extension, flexion), and exercises targeting stability, function, and posture, with a goal of 8-12 repetitions per set. At 12 repetitions with good technique, the prescribed resistance is increased. Participants are instructed in resistance band use, in preparation for the maintenance phase.

Dietary component: Participants shift to partial meal replacement at week 3, with 800-900 kcal daily on non-exercise days and an additional 150-200 kcal from meal replacement products on exercise days. In addition to meal replacement products, the dietary 'real food' components are the equivalent of 125 ml of milk (or non-dairy alternative), careful attention to adequate protein intake with up to 90 g of protein from foods (meat, fish, egg or alternative), 1 portion of fruit and 2 portions of non-starchy vegetables each day. Because the variety of *Optifast* products are more limited in Canada than in the UK, *ProtiDiet* (soups, oatmeal, and bars) are incorporated. Participants meet with the dietitian at weeks 4 and 8. The low energy diet is continued until week 12 and/or ideal weight (corresponding to BMI of 25

For peer review only

For peer review only - http://bmjopen.bmj.com/site/about/guidelines.xhtml

kg/m² for white participants and those of Indigenous origin and 23 kg/m² in participants of non-white or mixed background.)

Physician review: Participants meet with the study physician at weeks 4 and 8. The systolic blood pressure threshold to consider antihypertensive reintroduction at this stage is 140 mmHg, as in DiRECT (48).

Maintenance phase of intervention

Dietary component: By week 12's end, the dietitian and participant create an individualized plan for tapering meal replacement products, meeting again at weeks 14, 16, 18, and 21. The goal is to maintain any weight loss achieved in weeks 1 through 12. Meal replacement product tapering may be slowed, and product intake may even be increased again, in the event of weight regain.

Exercise component: Using monitoring tools, participants aim for 150 minutes of moderate to vigorous physical activity weekly, including three 30-minute dedicated sessions at 60 to 80% of the maximum heart rate achieved during the intermediary exercise stress test. Participants may use the study exercise facility or engage in home-based or community based exercise such as brisk walking and resistance training facilitated by bands. Participants have virtual or telephone contact with exercise physiologists at weeks 14, 16, 18 and 21. Those with less than 60 minutes of planned aerobic exercise and/or less than one resistance training session per week are asked to return for additional supervised sessions.

Physician monitoring: The study physician meets with participants at weeks 14 and 18. The thresholds for medication adjustment are as described previously. The HbA1c value at the intermediary evaluation may also be used to guide treatment.

For peer review only - http://bmjopen.bmj.com/site/about/guidelines.xhtml

End of intervention

Following final evaluations, intervention team members each meet with the participant to discuss strategies to maintain weight loss and exercise patterns. The study physician makes recommendations for medication management to the participant's usual treating physician, in conformity with national diabetes management guidelines and the participant's medication reimbursement plan regulations.

CONTROL GROUP

The control group receives care from their usual physician, in accordance with National Institute for Health and Care Excellence (NICE) (53) and Diabetes Canada (54) guidelines. Following final evaluations, control arm participants will be offered a 12-week low energy diet intervention followed by a 4-week food reintroduction period.

MONITORING FOR ADVERSE EVENTS

Intervention team members enquire about adverse events at each visit. Participants are asked to contact trial staff between visits to report any concerns. Serious adverse events will be reported within 7 days to all Research Ethics Boards and the Data Monitoring Committee which may seek a formal adjudication of relatedness by physicians blinded to the treatment arm and who have not interacted with the study participants in question.

More common adverse events in low energy diet trials (9, 55) include constipation (18 to 47%), headache (8%), dizziness (4 to 32%), fatigue (11 to 25%), and thirst (6%). Constipation responds to fibre-containing laxatives and fluid intake, and other symptoms resolve over time. In one high intensity aerobic training trial, (56) 16% experienced fracture or lower extremity muscle cramping and/or muscle, ligament, or tendon strain; 5.2% experienced chest pain, difficulty breathing, dizziness, or loss of consciousness. In a trial with a combined resistance and aerobic exercise arm (19), 12.5% had one of shoulder injury, left

For peer review only - http://bmjopen.bmj.com/site/about/guidelines.xhtml

knee pain, spinal stenosis exacerbation, or hip pain; one participant experienced atrial fibrillation.

WITHDRAWAL

Interventions and evaluations are halted in the event of intolerance to the study intervention or an adverse event that precludes participation, prolonged or serious hospital admission, the development of a serious co-morbid condition such as active malignancy, or pregnancy. We will document reasons for participant-initiated withdrawal, if specified.

COVID-19 RESTRICTIONS AND ADAPTATIONS

If COVID-19 restrictions are resumed, exercise physiologist led sessions using video conferencing may be used to replace some in-person supervised exercise sessions, using heart rate monitors, self-monitoring devices, and resistance bands. Any remaining in-person intervention visits or outcome measurement sessions will be undertaken using personal protective equipment following clinical guidelines that are current at the time of practice.

SAMPLE SIZE

In the DIRECT trial, remission was 46% at one year (vs. 4% in control arm; odds ratio 19.7) (9). Even higher remission was observed over the shorter term in other studies (57). We initially allowed for a minimum 45% remission at 24 weeks in the intervention group and up to a 5% remission rate in the control group. Using a Fisher exact test with 90% power, a significance of 0.05, and a 1:1 ratio between intervention and control groups, we estimated needing 56 individuals to complete the trial (control = 28, intervention n = 28). Allowing for up to 30% drop-out, we planned to recruit 80 individuals in total (40 UK, 40 Canada) in our successful application for funding to the Medical Research Council and the Canadian Institutes of Health Research.

For peer review only - http://bmjopen.bmj.com/site/about/guidelines.xhtml
For peer review only

We subsequently obtained additional funding from the Canadian JR McConnell Foundation, and increased recruitment goals in Canada from 40 to 60 participants (40 UK, 60 Canada). With an overall sample size of 100 (40 UK, 60 Canada), if 70 (70%) complete the trial, we will have 90% power to detect a minimum 35% remission at 24 weeks in the intervention group and up to a 5% remission rate in the control group.

Retention of at least 35 participants in each arm (70 total) will provide over 80% power to detect a difference in mean peak early diastolic strain rate of 0.10 s^{-1} difference, equivalent to the exercise effect in the DIASTOLIC trial (see BACKGROUND), (18) assuming that the common standard deviation is 0.144 as in the DIASTOLIC trial and using a two group t-test with a 5% two-sided significance level.

STATISTICAL ANALYSES

Our primary outcome measures the proportion of remission in the intervention arm relative to the control arm. We will apply intention-to-treat principles for the primary outcome, assuming that remission of diabetes did not occur if information is not available. Based on the DiRECT and DIADEM-I trials, we estimate that remission counts in the control arm could be less than 2. We will therefore use stratified Fisher's exact test statistics (i.e., stratification by country and by sex) if remission counts are low in the control arm but consider Pearson chi-square test, if appropriate. If numbers permit, logistic odds ratios with 95% confidence intervals (exact method) will also be performed to further quantify the efficacy of intervention over the control group, after adjusting for factors used to stratify the randomization (country, age, or sex); we will not have missing values for these covariates. Subgroup analyses for the primary outcome will be performed by country, sex, and the degree of weight loss (<5%, 5 to 10%, 10 to 15%, ≥15% as examined in the DiRECT trial (9), collapsing categories as needed, and by exercise adherence (<50%, >50% of supervised sessions attended). In a per-protocol analysis, we will restrict the intervention group to those

For peer review only - http://bmjopen.bmj.com/site/about/guidelines.xhtml

who achieved at least 10% weight loss and adhered to at least two thirds of prescribed exercise sessions.

We will analyse dichotomous secondary outcomes (e.g., diabetes remission at 12 weeks, hypertension remission at 12 and 24 weeks) as described for the primary outcome. We will analyse continuous key secondary outcomes (e.g., left ventricular peak early diastolic strain rate, lean soft tissue mass) through linear regression or log-linear regression, as appropriate, with stratification by country and sex.

PROCESS EVALUATION ANALYSES

At least two trained team members will independently code transcripts for themes (58), continually refining existing codes and identifying new ones. We will use Dedoose Version 7.0.23 and NVivo analysis software to facilitate data coding/organization.

DATA MANAGEMENT

Data management is facilitated by institutional REDCap systems, with access controlled through Active Directory Technology. Data and images shared across participating centres use secure data transfer systems with all identifiable information removed. Within REDCap, we have derived plausible ranges for all outcome measures; values outside these ranges are automatically flagged and verified for accuracy.

DISCUSSION

Low energy diets incorporating meal replacements can achieve T2DM remission through weight loss, (9-11) but may reduce lean mass (18, 19). Exercise can preserve lean mass (18, 19) and directly lower insulin resistance (20, 50, 59-61). Combined aerobic and resistance training yields additive glucose-lowering benefits (20). Regular exercise reduces CVD events (62-65) and improves MRI-based measures of heart health (18). RESET for REMISSION

For peer review only

will quantify remission of T2DM in young adults achieved through a combined low energy diet and supervised aerobic and resistance training intervention and will also ascertain changes in diastolic function, lean mass, and other factors that may impact long term outcomes.

Since funding was awarded for RESET for REMISSION, the literature has continued to evolve, with publication of the DIADEM-I trial, which targeted remission in younger adults less than 50 years of age of West Asian or North African ancestry. DIADEM-I combined a low energy diet (800-820 kcal/day) with exercise prescription from a trained professional centred on walking activities and self-monitoring tools, with resistance exercises introduced as the intervention progressed. Whilst this trial did not include supervised exercise training, the objective measurement of physical activity, or cardiac function and structure, it does provide robust evidence that a low energy diet combined with physical activity can be combined in younger adults with T2DM whilst leading to an equivalent level of remission as has been shown in older populations, as in DiRECT.

While young persons with T2DM stand to gain the most from remission, (24), their daily life demands may render recruitment and adherence challenging. We have endeavoured to mitigate this by integrating virtual communication tools for monitoring and follow-up where possible. Recruitment itself may be challenging in these younger individuals, as they remain fewer in number than middle-aged and older individuals with T2DM (27). We are therefore collaborating with a wide network of practices and drawing on other diabetes research cohorts in which participants have provided permission to be contacted for trials.
The international diabetes remission panel underscored the importance of evaluating the impacts of nonglycemic measures during remission. (3-6) RESET evaluates not only lipid profiles and MRI measures of pancreatic, hepatic, and visceral fat but also sensitive MRI-

For peer review only - http://bmjopen.bmj.com/site/about/guidelines.xhtml

based indicators of heart health for a comprehensive picture of intervention impacts. Equally importantly, we seek to understand impacts on quality of life, diabetes distress, and mood.

The Canada-UK collaboration that underpins our trial will allow a wider sharing of knowledge and perspectives than in a single-country study, challenging all to learn from best practices in each jurisdiction. The DiRECT trial led to an ongoing 5000 person pilot by the National Health Service in the UK for prescribing meal replacements as part of routine T2DM management (53). The visibility of the current trial and the Canada-UK partnership could be an important step towards considering implementing such an intervention with integration of exercise in Canadian health care jurisdictions. Indeed, a future trial may seek partners beyond these two countries, to move from a demonstration of efficacy to one of effectiveness across a wide range of settings, and a plan for implementation. This is a critical journey not only to meet the glycemic definitions of diabetes remission but also to establish its potential myocardial and vascular effects.

SPONSORS

The study is sponsored by the University of Leicester in the UK (RGOsponsor@leicester.ac.uk) and the Research Institute of the McGill University Health Centre in Canada (cinzia.raponi@muhc.mcgill.ca) who will ensure the study is conducted according to Good Clinical Practice and that all contractual, governance, ethics and regulatory processes are followed.

TRIAL STEERING COMMITTEE

This committee is responsible for the overall oversight of the trial and will review and approve protocol amendments, any sub-study proposals, review recruitment rates, protocol adherence, retention, compliance, safety issues, planned analyses and reports, and act on recommendations of the Data Monitoring Committee. The Trial Steering Committee includes

For peer review only - http://bmjopen.bmj.com/site/about/guidelines.xhtml

an independent Chair (Jason Gill, University of Glasgow), an independent clinician (Alice Cheng, University of Toronto), the Nominated Principal Investigators (Kaberi Dasgupta, McGill University & Thomas Yates, University of Leicester), the key site investigator in Edmonton (Normand Boulé, University of Alberta), and the trial statistician (Elham Rahme, McGill University). The Trial Steering Committee's operations are described in its charter (Appendix 2).

DATA MONITORING COMMITTEE

The Data Monitoring Committee is responsible for the interests and safety of the participants and its main role will be to make advisory recommendations to the Trial Steering Committee. It includes an independent clinician (Andrew Farmer, Nuffield Department of Primary Care Health Sciences, University of Oxford), an independent chair (Janusz Kaczorowski, University of Montreal) and independent statistician (Stephen Sharp, University of Cambridge). The Data Monitoring committee is independent from the sponsors and operates in conformity with a charter (Appendix 3).

PATIENT AND PUBLIC INVOLVEMENT

This group will provide feedback throughout the trial on recruitment, evaluation, and retention strategies and alternatives. They will be involved in interpretation of findings and knowledge dissemination efforts, including manuscript co-authorship, presentations, and interviews. The partners in Canada include Josette Spencer, Mark Marcinkiewicz, and Sylvie Lauzon, who is Executive Director of Diabetes Quebec. Patricia Kearns is a patient partner trainer who assisted with training of the patient partners. Alastair Masters is a patient partner based in the UK.

DISSEMINATION

We will publish the results of this trial in peer-reviewed journals and disseminate further in the scientific community through educational and conference presentations and social media

For peer review only - http://bmjopen.bmj.com/site/about/guidelines.xhtml

targeting health professionals and the scientific community. We will share findings with the general public through press releases, media interviews, and social media forums. The trial patient partners and participants will be invited to assist with knowledge sharing, including media interviews and op-eds.

DECLARATIONS

Ethics approvals and consent to participate

The trial has been approved in Leicester (East Midlands – Nottingham Research Ethics Committee [21/EM/0026]), Montreal (McGill University Health Centre Research Ethics Board [RESET for remission / 2021-7148]), and Edmonton (University of Alberta Health Research Ethics Board (Pro00101088). Trial candidates will be informed of all study procedures, with opportunity for questions and reflection. We will underscore confidentiality, voluntariness and freedom to withdraw from the trial at any time point. A trained member of the research team, named on the delegation of authority log, will obtain written informed consent will from trial candidates so that they may become participants. The Trial Steering Committee will review any proposed amendments to approved study documentation and these will then be submitted to the relevant Research Ethics Boards for re-approval prior to implementation. Any participant who wishes to make a complaint may do so through the standard National Health Service complaint system in Leicester, the McGill University Health Centre Ombudsman in Montreal, or the Health Research Ethics Board in Edmonton, as appropriate.

We will maintain an audit trail throughout the lifetime of the trial and all related documentation will be made available for sponsor monitoring and/or any external audits, as by ethics' committees. To maintain participant confidentiality, each participant will be assigned a unique identification number that will be used, without nominal information, on case report forms and other trial documents. Most trial materials will be stored electronically

For peer review only

in password-protected files on secure country-specific servers. The few documents in paper format will include the study identification number without nominal information and will be held in a locked cabinet accessible only to the research team at each trial site. We will hold participant identifiable information such as name and address in a trial site-specific, separate locked archive. All research data generated by the study will be stored for 25 years following trial completion, after which it will be destroyed.

Consent for publication

Not applicable

Availability of data and materials

Following the publication of the primary papers and all ancillary analyses by the research team, datasets will be available from the principal investigators (UK: Prof. Tom Yates - ty20@leicester.ac.uk) or (Canada: Prof. Kaberi Dasgupta - kaberi.dasgupta@mcgill.ca), contingent on the agreement of both Principal investigators and the associated Research Ethics Boards, on reasonable request. Any publications stemming from these data will be reported on group-level only so that individual participant anonymity is maintained.

Competing interests and disclosures

MGF is board member, shareholder, and consultant of Circle Cardiovascular Imaging Inc. Through the University Hospitals of Leicester NHS Trust, Co-investigator GM has a research agreement with Circle Cardiovascular Imaging Inc. We will be using software from this company to analyze the cardiac MRI images that we will obtain. K.K. has received honoraria from AstraZeneca, Boehringer Ingelheim, Eli Lilly, Janssen, Merck Sharp & Dohme, Novartis, Novo Nordisk, Sanofi, Takeda, Servier, and Pfizer and research support from AstraZeneca, Boehringer Ingelheim, Eli Lilly, Merck Sharp & Dohme, Novartis, Novo Nordisk, Sanofi, and Pfizer. MJD has acted as consultant, advisory board member and speaker for Novo Nordisk, Sanofi, Lilly and Boehringer Ingelheim, an advisory board

For peer review only

member and speaker for AstraZeneca, an advisory board member for Janssen, Lexicon, Servier and Gilead Sciences Ltd and as a speaker for Napp Pharmaceuticals, Mitsubishi Tanabe Pharma Corporation and Takeda Pharmaceuticals International Inc. She has received grants in support of investigator and investigator initiated trials from Novo Nordisk, Sanofi-Aventis, Lilly, Boehringer Ingelheim, Astrazeneca and Janssen. TY has received investigator initiated funding for obesity-related research from AstraZeneca.

Funding

The primary funders of this trial are the Medical Research Council (MR/T031816/1) of the United Kingdom and the Canadian Institutes of Health Research (UCD-170584; Institutes of Musculoskeletal Research and of Nutrition, Metabolism & Diabetes), organizations that have contributed equally to this endeavour. Additional funding in Canada was obtained from the John R McConnell Foundation to support additional enrolment in Canada, as previously described. The sponsors and funders have no direct role in study design, data collection, management, analysis, interpretation of data, report writing or publications that arise from the trial. For the Canadian sites, some capillary glucose meters and strips were donated by Roche Diabetes Care (Canada) and continuous glucose monitoring supplies by Dexcom (San Diego, CA).

For peer review only

Authors' contributions

Kaberi Dasgupta and Thomas Yates are the Principal Investigators of the trial and led the design and writing of this protocol, with critical input from Co-Investigators Normand Boulé and Joseph Henson, particularly related to the exercise intervention and fitness and strength evaluations; Stéphanie Chevalier, Emma Redman, Frank Arsenyadis, and Carla Prado, experts in nutrition and body composition measures; Gerry McCann, Matthias Friedrich, Richard Thompson and Abhishek Dattani, experts in MRI-based assessments of cardiac function and ectopic fat; Deborah Da Costa and Michelle Hadjiconstantinou, related to health

For peer review only - http://bmjopen.bmj.com/site/about/guidelines.xhtml

coaching and process evaluation; Matthew McCarthy, Deborah Chan, Julia Champagne, and Jordan Rees as related to trial logistics and procedures; Melanie Davies, Mark Sherman, and Roseanne Yeung, with important input on medication management; Edward Gregg and Kamlesh Khunti, for comments on recruitment and overall procedures; Elham Rahme, as related to statistical considerations; and Isabel Fortier, with respect to the data management plan.

Acknowledgements

Not applicable

For peer review only

Abbreviations

AE – Adverse Event

BMI – Body Mass Index

CI – Confidence Interval

CMR – Cardiac magnetic resonance

COM-B - Capability, Opportunity, Motivation - Behaviour

DDS – Diabetes Distress Scale

DXA – Dual-energy X-Ray Absorptiometry

ECG - Electrocardiography

EQ-5D-5L – European QoL -5 Dimensions

HbA1c – Hemoglobin A1c (glycated haemoglobin)

MRI – Magnetic Resonance Imaging

PROBE - Prospective, randomised, open-label, blinded-endpoint trial

PROFIT1 - PROton Density Fat Fraction Imaging and Water T1-Mapping with Low B1+ Sensitivity

REDCap – Research Electronic Data Capture System

ScAT - Subcutaneous Abdominal Adipose Tissue

For peer review only - http://bmjopen.bmj.com/site/about/guidelines.xhtml

T2DM – Type 2 Diabetes Mellitus

UK – United Kingdom

VAT - Visceral Adipose Tissue

WHODAS – World Health Organisation Disability Assessment Schedule questionnaire

REFERENCES

1. Saeedi P, Petersohn I, Salpea P, Malanda B, Karuranga S, Unwin N, et al. Global and regional diabetes prevalence estimates for 2019 and projections for 2030 and 2045: Results from the International Diabetes Federation Diabetes Atlas, 9(th) edition. Diabetes Res Clin Pract. 2019;157:107843.
2. Gong Q, Zhang P, Wang J, Ma J, An Y, Chen Y, et al. Morbidity and mortality after lifestyle intervention for people with impaires glucose tolerance: 30-year results of the Da Qing diabetes prevention outcome study. Lancet Diabetes Endocrinol. 2019;7(6):452-61.
3. Riddle MC, Cefalu WT, Evans PH, Gerstein HC, Nauck MA, Oh WK, et al. Consensus Report: Definition and Interpretation of Remission in Type 2 Diabetes. Diabetes Care. 2021. Aug 30:dci210034.
4. Riddle MC, Cefalu WT, Evans PH, Gerstein HC, Nauck MA, Oh WK, et al. Consensus report: Definition and interpretation of remission in type 2 diabetes. Diabet Med. 2021:e14669.
5. Riddle MC, Cefalu WT, Evans PH, Gerstein HC, Nauck MA, Oh WK, et al. Consensus Report: Definition and Interpretation of Remission in Type 2 Diabetes. J Clin Endocrinol Metab. 2021. Aug 30:dgab585.
6. Riddle MC, Cefalu WT, Evans PH, Gerstein HC, Nauck MA, Oh WK, et al. Consensus report: definition and interpretation of remission in type 2 diabetes. Diabetologia. 2021;64(11):2359-66.
7. Gregg EW, Chen H, Wagenknecht LE, Clark JM, Delahanty LM, Bantle J, et al. Association of an intensive lifestyle intervention with remission of type 2 diabetes. JAMA. 2012;308(23):2489-96.
8. Leslie WS, Ford I, Sattar N, Hollingsworth KG, Adamson A, Sniehotta FF, et al. The Diabetes Remission Clinical Trial (DiRECT): protocol for a cluster randomised trial. BMC Fam Pract. 2016;17:20.
9. Lean ME, Leslie WS, Barnes AC, Brosnahan N, Thom G, McCombie L, et al. Primary care-led weight management for remission of type 2 diabetes (DiRECT): an open-label, cluster-randomised trial. Lancet. 2018;391(10120):541-51.
10. Lean MEJ, Leslie WS, Barnes AC, Brosnahan N, Thom G, McCombie L, et al. Durability of a primary care-led weight-management intervention for remission of type 2 diabetes: 2-year results of the DiRECT open-label, cluster-randomised trial. Lancet Diabetes Endocrinol. 2019;7(5):344-55.
11. Taheri S, Zaghloul H, Chagoury O, Elhadad S, Ahmed SH, El Khatib N, et al. Effect of intensive lifestyle intervention on bodyweight and glycaemia in early type 2 diabetes (DIADEM-I): an open-label, parallel-group, randomised controlled trial. Lancet Diabetes Endocrinol. 2020;8(6):477-89.

For peer review only

For peer review only - http://bmjopen.bmj.com/site/about/guidelines.xhtml

12. Umpierre D, Ribeiro PA, Kramer CK, Leitão CB, Zucatti AT, Azevedo MJ, et al. Physical activity advice only or structured exercise training and association with HbA1c levels in type 2 diabetes: a systematic review and meta-analysis. Jama. 2011;305(17):1790-9.

13. Boule NG, Haddad E, Kenny GP, Wells GA, Sigal RJ. Effects of exercise on glycemic control and body mass in type 2 diabetes mellitus: A meta-analysis of controlled clinical trials. JAMA. 2001;286(10):1218-27.

14. Anand V, Garg S, Garg J, Bano S, Pritzker M. Impact of exercise training on cardiac function among patients with type 2 diabetes: a systematic review and meta-analysis. Journal of cardiopulmonary rehabilitation and prevention. 2018;38(6):358-65.

15. Verboven M, Van Ryckeghem L, Belkhouribchia J, Dendale P, Eijnde B, Hansen D, et al. Effect of exercise intervention on cardiac function in type 2 diabetes mellitus: a systematic review. Sports Medicine 2019;49(2):255-68.

16. Boonman-de Winter L, Rutten F, Cramer M, Landman M, Liem A, Rutten G, et al. High prevalence of previously unknown heart failure and left ventricular dysfunction in patients with type 2 diabetes. Diabetologia. 2012;55(8):2154-62.

17. Levelt E, Gulsin G, Neubauer S, McCann G. MECHANISMS IN ENDOCRINOLOGY: Diabetic cardiomyopathy: Pathophysiology and potential metabolic interventions state of the art review. Eur J Endocrinol. 2018;178(4):R127-R39.

18. Gulsin GS, Swarbrick DJ, Athithan L, Brady EM, Henson J, Baldry E, et al. Effects of Low-Energy Diet or Exercise on Cardiovascular Function in Working-Age Adults With Type 2 Diabetes: A Prospective, Randomized, Open-Label, Blinded End Point Trial. Diabetes Care. 2020;43(6):1300-10.

19. Villareal DT, Aguirre L, Gurney AB, Waters DL, Sinacore DR, Colombo E, et al. Aerobic or Resistance Exercise, or Both, in Dieting Obese Older Adults. N Engl J Med. 2017;376(20):1943-55.

20. Sigal RJ, Kenny GP, Boulé NG, Wells GA, Prud'homme D, Fortier M, et al. Effects of aerobic training, resistance training, or both on glycemic control in type 2 diabetes: a randomized trial. Ann Intern Med. 2007;147(6):357-69.

21. Yates T, Henson J, Sargeant J, King JA, Ahmad E, Zaccardi F, et al. Exercise, Pharmaceutical Therapies and Type 2 Diabetes: Looking beyond Glycemic Control to Whole Body Health and Function. Translational Medicine and Exercise Prescription. 2021;1(1):33-42.

22. Khan J, Wilmot E, Leggate M, Singh A, Yates T, Nimmo M, et al. Subclinical diastolic dysfunction in young adults with type 2 diabetes mellitus: A multiparametric contrast-enhanced cardiovascular magnetic resonance pilot study assessing potential mechanisms. European heart journal cardiovascular Imaging. 2014;15(11):1263-9.

23. Wilmot EG, Leggate M, Khan JN, Yates T, Gorely T, Bodicoat DH, et al. Type 2 diabetes mellitus and obesity in young adults: The extreme phenotype with early cardiovascular dysfunction. Diabet Med. 2014;31(7):794-8.

24. Bjornstad P, Drews KL, Caprio S, Gubitosi-Klug R, Nathan DM, Tesfaldet B, et al. Long-Term Complications in Youth-Onset Type 2 Diabetes. N Engl J Med. 2021;385(5):416-26.

25. Dabelea D, Stafford JM, Mayer-Davis EJ, D'Agostino R, Jr., Dolan L, Imperatore G, et al. Association of Type 1 Diabetes vs Type 2 Diabetes Diagnosed During Childhood and Adolescence With Complications During Teenage Years and Young Adulthood. JAMA. 2017;317(8):825-35.

26. Lascar N, Brown J, Pattison H, Barnett A, Bailey C, Bellary S. Type 2 diabetes in adolescents and young adults. Lancet Diabetes Endocrinol. 2018;6(1):69-80.

27. Federation ID. Diabetes Atlas, 9th edition [updated 2019. Available from: http://www.idf.org/diabetesatlas.

28. Graham-Brown M, Gulsin G, Parke K, Wormleighton J, Lai F, Athithan L, et al. A comparison of the reproducibility of two cine-derived strain software programmes in disease states. Eur J Radiol. 2019;113:51-8.

For peer review only - http://bmjopen.bmj.com/site/about/guidelines.xhtml

29. Gulsin GS, Swarbrick DJ, Hunt WH, Levelt E, Graham-Brown MPM, Parke KS, et al. Relation of Aortic Stiffness to Left Ventricular Remodeling in Younger Adults With Type 2 Diabetes. Diabetes. 2018;67(7):1395-400.

30. Singh A, Greenwood JP, Berry C, Dawson DK, Hogrefe K, Kelly DJ, et al. Comparison of exercise testing and CMR measured myocardial perfusion reserve for predicting outcome in asymptomatic aortic stenosis: the PRognostic Importance of MIcrovascular Dysfunction in Aortic Stenosis (PRIMID AS) Study. Eur Heart J. 2017;38(16):1222-9.

31. Taylor R, Al-Mrabeh A, Sattar N. Understanding the mechanisms of reversal of type 2 diabetes. Lancet Diabetes Endocrinol. 2019;7(9):726-36.

32. Thompson RB, Chow K, Mager D, Pagano JJ, Grenier J. Simultaneous proton density fat-fraction and R2∗ imaging with water-specific T(1) mapping (PROFIT(1)): application in liver. Magnetic resonance in medicine. 2021;85(1):223-38.

33. Roubille F, Fischer K, Guensch DP, Tardif JC, Friedrich MG. Impact of hyperventilation and apnea on myocardial oxygenation in patients with obstructive sleep apnea - An oxygenation-sensitive CMR study. J Cardiol. 2017;69(2):489-94.

34. Spath NB, Singh T, Papanastasiou G, Kershaw L, Baker AH, Janiczek RL, et al. Manganese-enhanced magnetic resonance imaging in dilated cardiomyopathy and hypertrophic cardiomyopathy. Eur Heart J Cardiovasc Imaging 2020. Nov 17:jeaa273.

35. Pavasini R, Guralnik J, Brown J, di Bari M, Cesari M, Landi F, et al. Short physical performance battery and all-cause mortality: Systematic review and meta-analysis. BMC Med. 2016;14(1):215-016-0763-7.

36. World Health Organisation: WHO disability assessment schedule 2.0 (WHODAS 2.0) [updated 2018. Available from: https://www.who.int/classifications/icf/whodasii/en/.

37. Mahler D, Wells C. Evaluation of clinical methods for rating dyspnea. Chest. 1988;93(3):580-6.

38. Migueles JH, Rowlands AV, Huber F, Sabia S, van Hees VT. GGIR: A Research Community–Driven Open Source R Package for Generating Physical Activity and Sleep Outcomes From Multi-Day Raw Accelerometer Data Journal for the Measurement of Physical Behaviour. 2019;2(3):188-96.

39. Zigmond A, Snaith R. The hospital anxiety and depression scale. Acta Psychiatr Scand. 1983;67(6):361-70.

40. Polonsky W, Fisher L, Earles J, Dudl R, Lees J, Mullan J, et al. Assessing psychosocial distress in diabetes: Development of the diabetes distress scale. Diabetes Care. 2005;28(3):626-31.

41. Herdman M, Gudex C, Lloyd A, Janssen M, Kind P, Parkin D, et al. Development and preliminary testing of the new five-level version of EQ-5D (EQ-5D-5L). Qual Life Res. 2011;20(10):1727-36.

42. Sandelowski M. What's in a name? Qualitative description revisited. Res Nurs Health. 2010;33(1):77-84.

43. Palinkas L, Horwitz S, Green C, Wisdom J, Duan N, Hoagwood K. Purposeful sampling for qualitative data collection and analysis in mixed method implementation research. Adm Policy Ment Health. 2015;42(5):533-44.

44. Murray E, Treweek S, Pope C, MacFarlane A, Ballini L, Dowrick C, et al. Normalisation process theory: A framework for developing, evaluating and implementing complex interventions. BMC Med. 2010;8:63-7015-8-63.

45. Michie S, van Stralen MM, West R. The behaviour change wheel: a new method for characterising and designing behaviour change interventions. Implement Sci. 2011;6:42.

46. Braun V, Clarke V. Using thematic analysis in psychology. Qualitative Research in Psychology. 2006;3(2):77-101.

47. Baldry EL, Davies MJ, Khunti K, Webb DR. Pragmatic management of low-energy diets in people with type 2 diabetes in primary care: a decision aid for clinicians. Diabet Med. 2020;37(5):747-51.

For peer review only

For peer review only - http://bmjopen.bmj.com/site/about/guidelines.xhtml

48. Leslie WS, Ali E, Harris L, Messow CM, Brosnahan NT, Thom G, et al. Antihypertensive medication needs and blood pressure control with weight loss in the Diabetes Remission Clinical Trial (DiRECT). Diabetologia. 2021;64(9):1927-38.
49. Terada T FA, Chahal BS, Bell GJ, McCargar LJ, Boulé NG. Feasibility and preliminary efficacy of high intensity interval training in type 2 diabetes. Diabetes Res Clin Pract. 2013;99(2):120-9.
50. Jelleyman C, Yates T, O'Donovan G, Gray LJ, King JA, Khunti K, et al. The effects of high-intensity interval training on glucose regulation and insulin resistance: a meta-analysis. Obes Rev. 2015;16(11):942-61.
51. Francois M, Little J. Effectiveness and safety of high-intensity interval training in patients with type 2 diabetes. Diabetes Spectr. 2015;28(1):39-44.
52. Cassidy S, Thoma C, Houghton D, Trenell M. High-intensity interval training: A review of its impact on glucose control and cardiometabolic health. Diabetologia. 2017;60(1):7-23.
53. Type 2 diabetes in adults: management NICE guideline [NG28] 2015 [updated 2021. Available from: https://www.nice.org.uk/guidance/ng28.
54. Guidelines DCCP. 2020 [Available from: http://guidelines.diabetes.ca/cpg.
55. Astbury NM, Aveyard P, Nickless A, Hood K, Corfield K, Lowe R, et al. Doctor Referral of Overweight People to Low Energy total diet replacement Treatment (DROPLET): pragmatic randomised controlled trial. Bmj. 2018;362:k3760.
56. Ross R, Hudson R, Stotz PJ, Lam M. Effects of exercise amount and intensity on abdominal obesity and glucose tolerance in obese adults: a randomized trial. Ann Intern Med. 2015;162(5):325-34.
57. Parretti H, Jebb S, Johns D, Lewis A, Christian-Brown A, Aveyard P. Clinical effectiveness of very-low-energy diets in the management of weight loss: a systematic review and meta-analysis of randomized controlled trials. Obes Rev. 2016;17(3):225-34.
58. Brooks J, McCluskey S, Turley E, King N. The utility of template analysis in qualitative psychology research. Qual Res Psychol. 2015;12(2):202-22.
59. Yates T, Davies M, Gorely T, Bull F, Khunti K. Effectiveness of a pragmatic education program designed to promote walking activity in individuals with impaired glucose tolerance: a randomized controlled trial. Diabetes Care. 2009;32(8):1404-10.
60. Dasgupta K, Rosenberg E, Joseph L, Cooke AB, Trudeau L, Bacon SL, et al. Physician step prescription and monitoring to improve ARTERial health (SMARTER): A randomized controlled trial in patients with type 2 diabetes and hypertension. Diabetes Obes Metab. 2017;19(5):695-704.
61. Boulé NG, Haddad E, Kenny GP, Wells GA, Sigal RJ. Effects of exercise on glycemic control and body mass in type 2 diabetes mellitus: a meta-analysis of controlled clinical trials. Jama. 2001;286(10):1218-27.
62. Paffenbarger RS, Jr., Hyde RT, Wing AL, Hsieh CC. Physical activity, all-cause mortality, and longevity of college alumni. N Engl J Med. 1986;314(10):605-13.
63. Hu FB, Willett WC, Li T, Stampfer MJ, Colditz GA, Manson JE. Adiposity as compared with physical activity in predicting mortality among women. N Engl J Med. 2004;351(26):2694-703.
64. Florido R, Kwak L, Lazo M, Nambi V, Ahmed HM, Hegde SM, et al. Six-Year Changes in Physical Activity and the Risk of Incident Heart Failure: ARIC Study. Circulation. 2018;137(20):2142-51.
65. Moholdt T, Lavie CJ, Nauman J. Sustained Physical Activity, Not Weight Loss, Associated With Improved Survival in Coronary Heart Disease. J Am Coll Cardiol. 2018;71(10):1094-101.

For peer review only - http://bmjopen.bmj.com/site/about/guidelines.xhtml

FIGURE 1. TRIAL SCHEMATIC

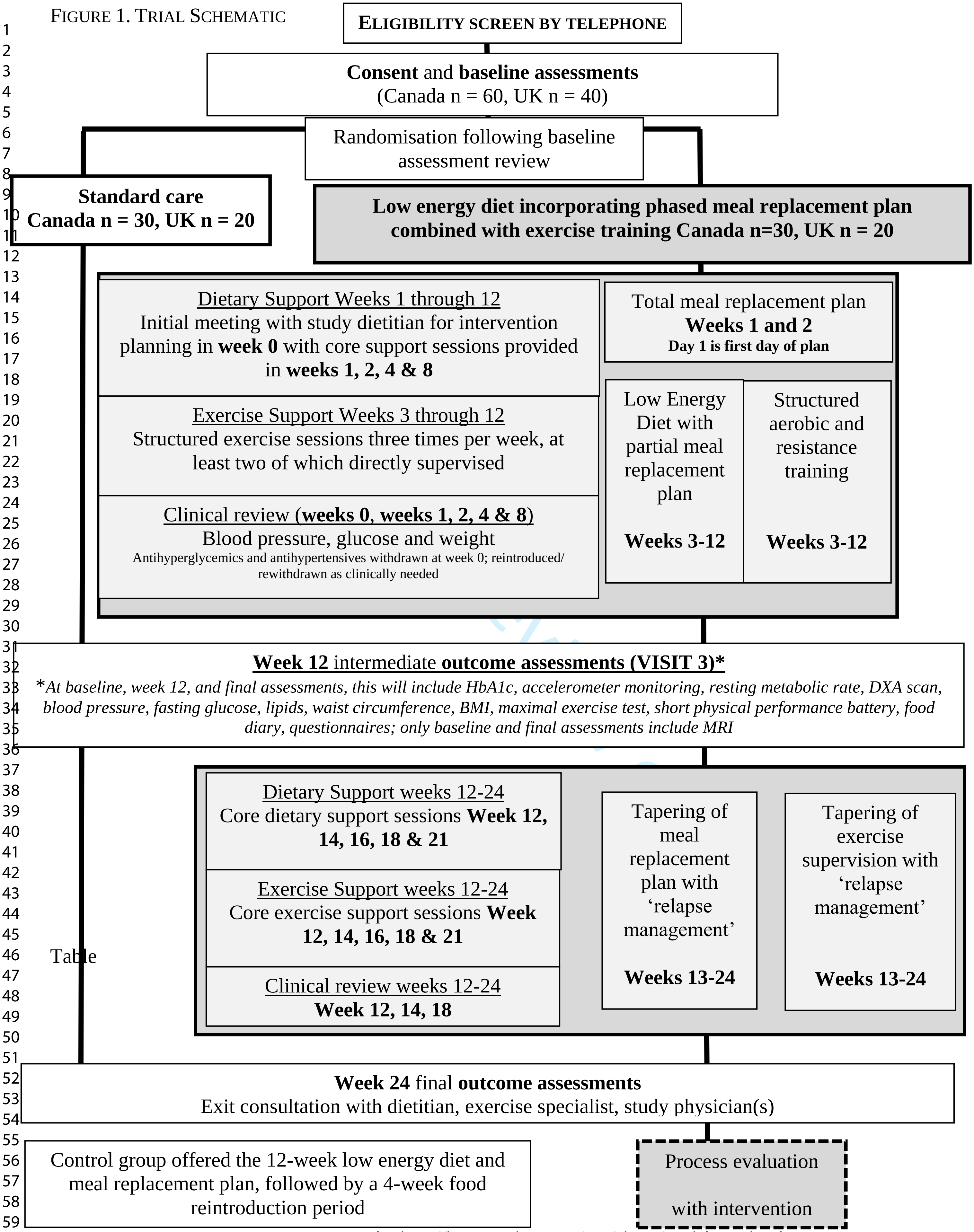

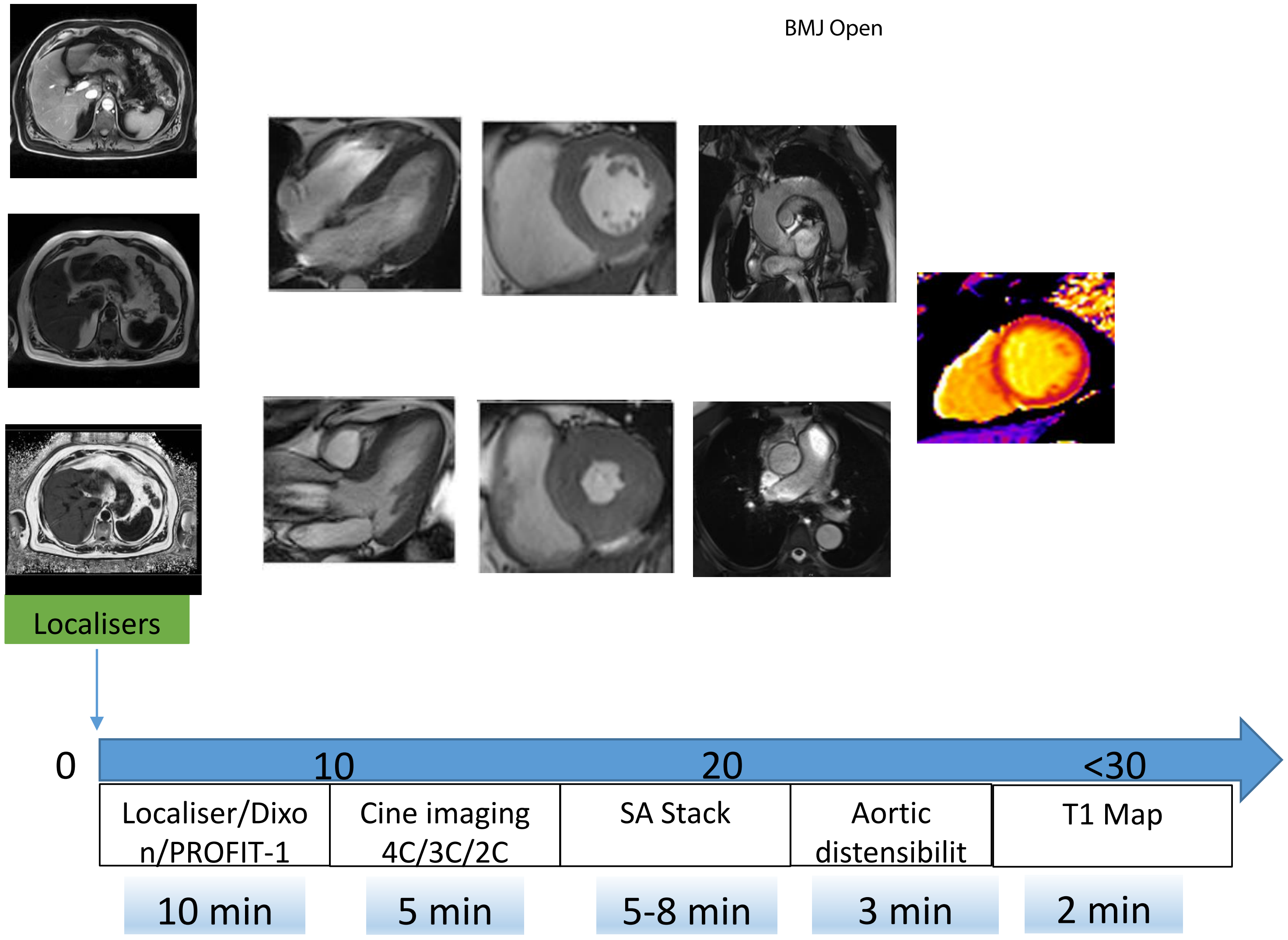

Figure 2: Core MRI protocol of the RESET FOR REMISSION trial.

Legend: The core protocol can be completed in under 30 minutes and comprises: localisers, fat-water imaging (Dixon) which allow assessment of subcutaneous and visceral adiposity (optional PROFIT-1 which adjusts liver fat measurement for fibrosis); cardiac cine imaging comprises long axes and complete coverage of the left ventricle and perpendicular to the thoracic aorta allowing calculation of myocardial volumes, mass, ejection fraction, strain/strain rates (see figure 3) and aortic distensibility; finally native T1 mapping in a single midventricular slice will be acquired as a surrogate marker of myocardial fibrosis.

Abbreviations: PROFIT1: simultaneous proton density fat fraction imaging and water T1-mapping with low B1+ sensitivity; 4C, 4-chamber; 3C, 3-chamber; 2C, 2-chamber. SA, short-axis.

For peer review only - http://bmjopen.bmj.com/site/about/guidelines.xhtml

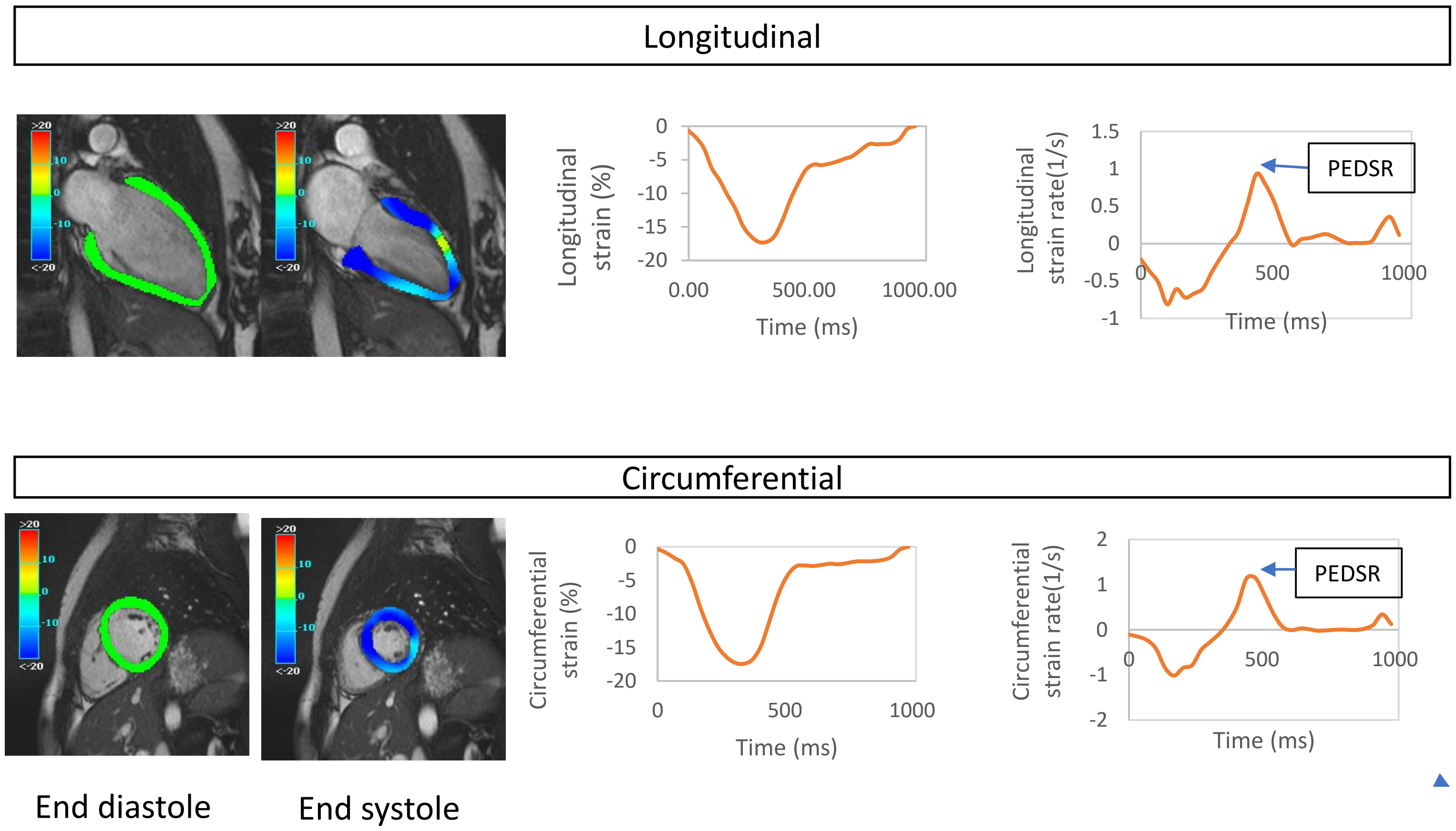

Figure 3: A typical example of strain analysis from cardiac MRI cine imaging.

Top panel 2 chamber view which allows derivation of longitudinal strain/strain rate; bottom panel mid ventricular short axis from which circumferential strain and strain rate is derived. Left: images showing color representations of longitudinal and circumferential strain at end-diastole and end-systole, with green showing 0%, light blue showing -10% and dark blue showing -20% strain (more negative indicating higher strain). Middle: graphs showing global longitudinal strain and global circumferential strain. Right: graphs showing longitudinal and circumferential strain rate throughout the cardiac cycle.

Abbreviations: PEDSR= peak early diastolic strain rate, a sensitive measure of diastolic relaxation and a key secondary outcome

For peer review only - http://bmjopen.bmj.com/site/about/guidelines.xhtml

Appendix 1. Interview guide for in-depth interviews with intervention arm participants at completion of final evaluations

This topic guide is a flexible tool and may be revised as new areas of interest arise during the process of data collection. The wording of the question is for guidance only and can be varied to suit the natural style of the interviewer and the level of understanding of the participant. This topic guide will be adapted to use for those who are involved in this study.

The interviewer will be a trial team member who has not interacted with the participant during the course of the trial. The interview may be conducted in person, by video teleconference, or by telephone, as preferred by the participant.

Introductory remarks

The interviewer will iterate that the purpose of the interview is to:

- Explore the participant's knowledge, views and experience of the RESET trial interventions

Participants will be assured that:

- They are not being 'tested' – there are no right or wrong answers.
- They are free to say as much or as little as they want.
- Their specific comments will be anonymously quoted in manuscripts and reports. Individual comments will not be shared with anyone external to the research team.

Recording will be started after introductory remarks.

Questions to discuss

1. What made you want to participate in this study?

For peer review only

2. When you started, did you feel you were you prepared to invest time and effort to make the intervention work?

3. When you were assigned to the ‘active intervention’ group, what was your understanding of how the intervention could benefit you?

4. What are your thoughts about this programme overall? Probe: What did you think about the format? The length of the sessions? What did you find easy to do? What was difficult?

5. Were you able to attend the sessions? Probe: if participants respond no, probe reasons for not being able to attend.

6. Was the venue convenient for the exercise training? (i.e. easy to attend) If not, what would have helped to make the venue convenient to you?

7. Dietary component:

   7a) What was your experience with the dietary support sessions? Prompt: meal replacement products, dietary support sessions, limited amounts of food

   7b) How did you find the reintroduction to food? Prompt: questions around pace, was it manageable, did they require more support etc.

   7c) Can you tell us if you felt the sessions helped in any way to manage your diet? Probe in what way do you feel this has helped, or has not helped?

For peer review only

8. Exercise component:

8a) You took part in exercise training (combined aerobic and resistance activities) – Can you tell me a little bit about this? Prompt: difficulty, convenience, motivation, challenges in combination with diet

8b) How well were you able to exercise independently once the close supervision of the first 12 weeks ended? Prompt: Challenges, need for return to supervision

8c) Can you tell us if you felt the sessions helped in any way to manage your exercise training? Probe: in what way do you feel this has helped, or has not helped?

9. Medication adjustments, glucose and blood pressure:

9a) How did you feel about stopping some medications at the beginning of the program? Probe: What concerns, if any, did you have?

9b) How did you feel about your glucose levels and blood pressure levels during the program? Probe: Did you feel that you were well-monitored during this period?

10. Did you make any changes in your eating? Probe: If yes, when you were making your diet changes, did you feel you were making a difference to your health?

11. Did you make any changes in your exercise? Probe: If yes, when you were making your exercise changes, did you feel you were making a difference to your health?

12. What skills do you feel you have taken from taking part in the intervention?

13. Diet component

13a) Were there any tasks or time constraints that would influence your ability in making changes to your diet? Elaborate

For peer review only

13b) What factors would you say influenced whether you were able to maintain your diet plan?

14. Exercise component

14a) Were there any tasks or time constraints that would influence your ability in making changes to you exercise? Elaborate

14b) What factors would you say influenced whether you were able to maintain your exercise plan?

15. Did you ever discuss the programme with your family members or friends? Probe: What did they think of your participation? Were they supportive? Concerned? Were they sceptical?

16. Would your family members or friends influence what you did as part of the programme? If so, to what extent? Prompt: i.e. was their influence positive? Was it negative?

17. Do you feel the intervention affected your relationships in any way? Prompt: i.e. by not eating regular food based meals with the family

18. If you made changes to your eating or physical activity, how important do you think it is for you to maintain these changes to your health? (elaborate why this is or isn't important to the participants)

For peer review only

19. How optimistic are you about maintaining these changes to your health? Prompt: if not optimistic, explore the reasons why

20. If there was additional support available in the intervention, what would this be?

21. What would you do differently if you were asked to take part again?

22. Do you have any suggestions for improving the programme?

For peer review only

Centre universitaire de santé McGill — McGill University Health Centre

Centre universitaire de santé McGill — McGill University Health Centre

X HME MCH X HGM MGH X HRV RVH
X HNM MNH X ITM MCI X CL LC

* F M U - 9 9 9 7 *

INFORMATION AND CONSENT FORM

Research Study Title: REMISSION OF DIABETES AND IMPROVED DIASTOLIC FUNCTION BY COMBINING STRUCTURED EXERCISE WITH MEAL REPLACEMENT AND FOOD REINTRODUCTION IN ENGLAND AND CANADA: THE **RESET FOR REMISSION** TRIAL

Protocol number: 2021-7148

Researcher responsible for the research study: Dr. Kaberi Dasgupta, Professor of Medicine and Physician, MUHC; Scientist, Research Institute of the MUHC

Co-Investigators:
Dr. Stéphanie Chevalier, Associate Professor of Medicine and Scientist, Research Institute of the MUHC
Dr. Deborah Da Costa, Associate Professor of Medicine and Scientist, Research Institute of the MUHC
Dr. Elham Rahme, Associate Professor of Medicine and Scientist, Research Institute of the MUHC
Dr. Matthias G. Friedrich, Professor of Medicine and Radiology and Physician, MUHC
Dr. Isabel Fortier, Assistant Professor of Medicine, MUHC; Scientist, Research Institute of the MUHC
Dr. Mark Sherman Associate Professor of Medicine and Physician, MUHC

Funding source: The Canadian Institutes of Health Research (CIHR)

Sponsor: The Research Institute of the MUHC

INTRODUCTION

We are inviting you to take part in this research study because you were diagnosed with Type 2 diabetes less than 6 years ago.

However, before you accept to take part in this study and sign this information and consent form, please take the time to read, understand and carefully examine the following information. You may also want to discuss this study with your doctor, a family member or a close friend.

We invite you to speak to the researcher responsible for this study ("the researcher") or to other

For peer review only - http://bmjopen.bmj.com/site/about/guidelines.xhtml

members of the research team and ask them any questions you may have about this study. Please also ask a member of the research team about any parts of this consent form you do not understand.

BACKGROUND

More and more people are getting type 2 diabetes. They are also getting it at younger ages (aged between 18-40 years) than in the past. This is worrying because heart, kidney and physical fitness problems related to diabetes can therefore also happen at an earlier age than in the past.

Past studies show that diets with a low number of calories can make blood sugar levels go back to normal without diabetes medications in people who have had type 2 diabetes for just a few years. This is called 'reversing diabetes.' A low calorie diet means eating a lot less than normal for some weeks often with special shakes and meal bars, with the guidance of a dietitian. Once people have lost enough weight, they can start to eat more regular food again while trying to keep off the weight they may have lost.

Exercise, such as walking and simple muscle building activities, can also make your blood sugar levels better and help your heart and muscles work more efficiently. Putting a low calorie diet *together* with a supervised exercise program has not been studied before to look at how well it works in reversing diabetes and improving how the heart and muscles work.

PURPOSE OF THE RESEARCH STUDY

The main goal of this study is to see whether combining exercise and a low calorie diet can reverse diabetes and make the heart and muscles work better.

This study is a randomized trial. This means that if you agree to participate in this study, you will be randomly assigned to one of two groups based on chance, like rolling a dice or flipping a coin. The two groups in this trial are:

- Group 1: **usual standard care** (that means continuing to be treated as you are right now)
- Group 2: a combination of an **exercise program and a low calorie diet**.

Neither you nor the researchers can choose the group to which you will be assigned. We will not know in which group you will be assigned until the trial begins. Both groups will have a series of tests and questionnaires at the beginning, middle, and at the end of the study, so we can compare the groups and what happens to them.

For this research study, we will recruit 30 to 35 people in Montreal. They will be men and women, between 18 and 40 years of age.

DESCRIPTION OF THE RESEARCH PROCEDURES

This research study will take place at the McGill University Health Centre. During the study, the research staff may communicate with you via e-mail, cell phone texting (standard text message rates apply) or video call.

For peer review only - http://bmjopen.bmj.com/site/about/guidelines.xhtml

Your participation in this research project will last 6 to 7 months and will include on-line questionnaires and at least 5 visits for testing (both Group 1 and Group 2). For those who are in the Group 2 (structured exercise with low calorie diet group), there will be more visits for counselling and supervision. Before we ask you to answer questionnaires or schedule visits, the study doctor will first go through your medical records and may talk to your doctor to make sure that you are eligible for the study. If you are eligible, we will ask you to complete the steps below.

1. Duration and number of visits

You will first complete an on-line questionnaire, an on-line 4-day food diary, and 1 week of physical activity monitoring with a device worn on your wrist. A paper-based alternative to the diary and questionnaires is also available, if you prefer. If you complete these procedures, you will be scheduled for the first two visits (visit 1 and visit 2 described in the table below). The study doctor will review the results of the first two visits to confirm that it is okay for you to continue the research. If it's okay, then we will do the randomization (like 'flipping a coin') to decide which of the two study groups you will be in.

2. Description of the trial groups and related procedures

GROUP 1- Standard care

If you are in the standard care group, you will receive your normal diabetes care. You will also be given a leaflet about the benefits of diet and exercise. You will come back for VISITS 3, 4, and 5, as described above. If you complete all visits, **you will be offered the low calorie diet at the end of the trial, in thanks for your participation.**

GROUP 2- Low calorie diet and structured exercise

<u>Glucose and blood pressure medications and study physician supervision</u>

We will stop your diabetes and most blood pressure medications at the beginning of the diet and exercise program. This is because the low calorie diet and the structured exercise will probably lower your blood sugar and blood pressure quite a lot. To be safe, we have to stop the medications.

A study doctor will see you at the beginning of the study and will stay in contact with you throughout the 24 weeks to monitor your blood sugar and blood pressure and decide with you if any medications need to be restarted. You will be asked to monitor your blood glucose more often. For study purposes, we will lend you a home blood pressure monitor and a weighing scale and provide you with glucometer strips if you do not have enough to measure your glucose levels at home.

You will measure your glucose levels, blood pressure, and body weight at home, and report these back to the study physician, dietitian, and exercise specialist, as they instruct. In general, you will be asked to check your glucose level every day before your first meal and 7 times the day before each time you meet with the study doctor (before and 2 hours after each 'meal' and at bedtime), your blood pressure when you wake up in the morning, and your body weight at least once each week. You will be measuring your glucose levels during the study with the usual way of pricking your finger with a lancet, placing a drop of blood on a test strip, and inserting the strip into a glucometer. You will measure your blood pressure with the monitor that we lend you during the study. You will sit comfortably with your back supported, attach the blood pressure cuff (like a band) on your upper arm, and rest for about 5 minutes. You will

For peer review only - http://bmjopen.bmj.com/site/about/guidelines.xhtml

then press a button to start the reading. Once the reading is done, you will push the button for a second reading, and this is the reading you will write down. You will write the blood pressure (first number and second number), body weight, and glucose values into an online diary that you and the study physician, dietitian, and exercise physiologist will be able to access. If you prefer, we can give you a paper diary instead. We will give you a fibre-based laxative (Metamucil) to take when needed in case you become constipated while on the low calorie diet described below.

We emphasize that it is very important not to become pregnant during the study. With this in mind, if you are female, the study physician will review your use of reliable contraception throughout the study. If you become pregnant, all study interventions will be stopped.

<u>The low calorie diet</u>
This diet will be a major change in your life for 12 weeks. You have to be aware of what is involved. You will have regular support from a study dietitian. This will be face to face or through telephone calls or virtual meetings.

Part 1 (2 weeks). You will have a "total dietary replacement" where you only consume special shakes and lots of water. The shakes will give you around 900 calories each day. All of the shakes will be free of charge and will be give to you the first time you meet the dietitian, which will be face-to-face at least the first time.

Part 2 (10 weeks). After the first two weeks, you will eat one meal with regular food each day. The rest of what you eat will be meal replacement products like the shakes you had the first two weeks. To give you more variety, on top of shakes, you will also get special soups and bars. On most of the days each week (4 out of 7 days), you will still have about 900 calories each day (all of the meal products and the one meal each day all considered together).

During this 'part 2' time, you will also be exercising 3 days each week. On the days that you exercise, you will have some extra meal replacement product so on those days you will be taking in a bit more than 900 calories.

You and the dietitian will figure out together what kinds of meals you will eat to make sure that you are not taking in more calories than the limit set by the study. She will work with you to pick foods that you prefer. She will also help you figure out what combination of shakes, bars, and soups you might like best.

Part 3 (12 weeks). At this point, you will probably have lost weight and we will now try to help you keep it off. Even if you have not lost weight, we will help you transition back to a healthy food-based diet. The dietitian will help you bring back normal foods. She will calculate about how many calories you will need every day to keep your weight the same as it is at 12 weeks. We will support you to figure out portion sizes and food choices. The goal is to help you keep off the weight you have lost. If you gain weight, she will reintroduce or increase some meal replacement products and remove some regular meals from your plan, for a period of time.

For peer review only

For peer review only - http://bmjopen.bmj.com/site/about/guidelines.xhtml

<u>The structured exercise program</u>
The exercise program will start after your first two weeks of shakes. We don't want to overwhelm you with too many changes at once, so we will start the exercise program at the same time that you get one food meal back in your diet at week 3.

Just as the dietitian supervises you in the low calorie diet part, an exercise specialist (exercise physiologist) will supervise you in the exercise part. The sessions are free of charge.

Part 1 – During the first 2 weeks when you are having the shakes only diet, you will continue with your usual activities.

Part 2 -- During the next 10 weeks you will exercise, you will have two supervised exercise sessions at the PERFORM Centre, located at 7272 Sherbrooke Street West. You will take part in lots of different exercises designed to increase your fitness and strength. The planned exercise will take into account your level of fitness and ability. You will use a treadmill, free weights, bands, and weight machines. The exercise specialist will help you use these safely and effectively. The specialist will design a third session for you to do each week. This 'unsupervised' session can be done outdoors, at another gym, or at home but you will also have the option to come to PERFORM for this, to access the equipment and facilities. Please note, however, that if there are any government-mandated COVID restrictions in place, the PERFORM gym will not be available for unsupervised exercise. You will wear a Fitbit device that we will lend you so that you and we can monitor your unsupervised exercise and activities of daily living.

Part 3 – During the next 12 weeks, you will exercise on your own. The goal is for you to keep exercising at the level and amount that you have been doing in part 2. The exercise specialist will make a plan with you and will be checking in with you by phone, text, or e-mail. If you are having difficulty or are not exercising regularly, you will be asked to return for some supervised sessions. When you are exercising on your own during these 12 weeks, you will still have the option of exercising at the PERFORM Centre. As we state above, however, if there are any government-mandated COVID restrictions in place, the PERFORM gym will not be available for unsupervised exercise. You will wear a Fitbit device that we will lend you to track your aerobic exercise and physical activity.

Additional glucose sensors
As we talked about above, you will be testing your blood sugar levels at least once a day at home and 7 times on the day before you have a visit with the study doctor. You and the study doctor will know all of these numbers. On top of that, we will measure your glucose levels throughout the day using a special device called a continuous blood glucose monitor (CGM). The study physician won't use the CGM numbers to guide you; she will stick to the numbers you measure yourself at home. We will use the CGM numbers for research analysis ('research math') to understand overall the patterns of blood sugar change in the two study groups. The research team will download and store these numbers for analyses.

We will show you how to place the CGM sensor on the skin of your upper arm. It has a small filament that is inserted under your skin with a small needle (less than 1 cm long). The needle is then removed and only the flexible filament remains under your skin. We will show you how to tape the sensor to

For peer review only

For peer review only - http://bmjopen.bmj.com/site/about/guidelines.xhtml

your arm. You can shower with it. Once in place, you do not need to do anything else with it. The CGM sensor measures your blood glucose continuously and stores values every 15 minutes.

We will ask you to wear a CGM sensor at 4 different time points, each time for two weeks. These are the 4 time blocks:
(1) The week before you start the low energy diet and the first week of the low energy diet;
(2) The second week of the low energy diet and the first week supervised exercise is added;
(3) The 11th and 12th weeks after you start the diet, when we should start seeing the strongest effects of what you are doing;
(4) The 23rd and 24th weeks when you are finishing up the study.

Each time you put on one of these sensors, you will mail it back to us after two weeks in a stamped envelope we will give you or you will bring it to the PERFORM centre where we will collect it from you.

3. Tests and procedures

After you agree to join the study, we will ask you to do the following:

Accelerometer to monitor your activity & sleep) for 7 days	We will mail you a small machine to wear on your wrist called a research accelerometer. This measures how much time you spend sleeping, sitting and moving. It can be worn all day and night. We will ask you to wear it for 7 days. You will then mail it back to us in the postage paid envelope that we will give you.
Food diary	We will explain to you how to log onto an online food diary or mail you a paper one to complete. We will ask you to track what you eat over 4 days (three weekdays and one weekend day). If you complete a paper diary, you will mail it back with the accelerometer. This will help you understand what your food habits are.
Questionnaire – done at home	We will ask you to fill out an online questionnaire (or paper version, if you prefer). You can reach our study staff by phone or email to answer any questions you have. You also have the option of answering the questions by phone and the staff can fill in the answers for you.<br>Examples of what the questions will be about are feeling anxious or depressed, how well you function in your day to day life, history of illness in your family, your cultural or ethnic background, any trouble breathing, how much schooling you completed, the kind of work you do if you are employed, and whether or not you have a partner. We will also ask female participants about previous pregnancies. We estimate that these questions will take about 20 minutes to complete.

If you do not complete the above procedures, you will not be invited to continue in the study. Once you complete the above procedures at home, you will be scheduled for some face-to-face visits for evaluation.

For peer review only - http://bmjopen.bmj.com/site/about/guidelines.xhtml

The study doctor or a member of the research team will conduct the following tests and procedures:

DESCRIPTION OF STUDY PROCEDURES	
**Procedures at visit 1, 3 and 4**	**Description**
Preparation for visits 1, 3, and 4 (fasting)	To make sure we have accurate results on the tests we are doing, we ask you not to exercise or drink alcohol for two days before you come to see us.<br>We also have to ask you to fast and not smoke the night and following early morning (about 10 hours total) before you come in to have accurate results on blood tests.<br>Do not take your diabetes medications until we give you a snack later in the morning before the exercise test.<br>You can take the other medications that you would normally take at that time of day
Urine collection	We will ask you to go to the washroom to empty your bladder into a container that we will give you. We will use the urine sample to check how your kidneys are working. If you are a woman who is able to become pregnant, we will do a urine test for pregnancy before you start participating in the study. We will ask you to put on a hospital gown.
Height, weight, neck, waist and hip measures	We will ask you change into a hospital gown. We will weigh you (in the gown *without* shoes) and measure your neck, waist, hip and height.
Indirect calorimetry	This test will tell us how much energy you use when you are still. It reflects the amount of energy your body uses to keep your blood flowing and your organs working. You will lie still on a table under a clear hood, staying awake. A machine connected to the hood will measure how much oxygen you are breathing in and how much carbon dioxide you are breathing out.
Review of your medications	You will bring in a list of your medications (with doses) or the medication containers themselves. These will be noted down by study staff. They will ask you some more questions about your medical history and current health.
Blood pressure	We will ask you to sit down quietly for 5 minutes. We will then take one measure from the left arm and then the right arm. Then six measures will be taken 1-minute apart in the arm that had the higher value on the first measurement.
Bioelectrical Impedance Analysis (BIA) test	This test will estimate your body fat and 'lean' mass (muscle) by running a small electrical current through the body that you will not feel. You will stand on the machine and hold onto the bars.
Blood collection	We will take some blood (about 34 mL or roughly 7 teaspoons) to measure the amount of sugar and fat in your blood and other blood tests related to type 2 diabetes and the heart.
Fitness test	We will give you a snack (or if this is visit 3, a lunch) before the fitness test.

For peer review only

For peer review only - http://bmjopen.bmj.com/site/about/guidelines.xhtml

	You will be walking/running on a treadmill. The speed will remain the same but the incline of the treadmill will slowly increase. We will ask you to keep going as long as you can to figure out how fit you are. It will feel feel like climbing up a hill that gradually becomes steeper and steeper. You will be asked to breathe through a mask in order to collect and measure the carbon dioxide you breathe out and the oxygen you breathe in.
**Procedures at visit 2 and 5**	
Preparation for Visits 2 and 5	Please do not smoke, consume caffeine-containing beverages or foods (coffee, tea, cocoa, chocolate, “energy drink” or cola) for 12 hours before MRI visit and do not eat for 4 hours before the MRI Visit. If you are on a type of medication for blood pressure called a beta-blocker, they study doctor will ask you to not take it 12 hours before the MRI.
MRI	Magnetic Resonance Imaging (MRI) is an imaging method that uses a magnetic field with radio frequency waves, which allows doctors to see your organs inside your body. Before your scan, we will figure out if you have been taking a medication called a beta-blocker for high blood pressure; if yes, on the day of the scan, we will ask you to delay taking it until after the can. During the scan, you will lie on your back inside the scanner. The inside of the machine is quite small. Some people may start to feel claustrophobic. When the scanner is working, it makes loud buzzing sounds. Therefore, you will wear headphones. We will use the scanner to take pictures of the heart. We will also measure how much fat you have in and around your organs. The MRI will produce images and the computer will collect this information. Some images will require for you to hold your breath for a short period of time (2-15sec) and the technologist will give you specific instructions. It is very important that you don’t move when the picture is taken, as this can blur the pictures. Your heart, blood pressure, and blood oxygen levels will be closely monitored during the test. Electrocardiogram (ECG) electrodes (stickers) will be placed on your chest. A blood pressure cuff will be placed around your upper arm. A pulse oximeter (a small device to measure the amount of oxygen in your blood) will be attached to one of your fingers.
DXA (Dual Energy X-ray Absorptiometry) scan	This test uses X-rays to measure the overall amount of fat and muscle you have in your body. We will also ask you to stay as still as possible. You will lie on a padded table. There will be a machine that makes X-rays below the table. There will be a moveable machine ‘arm’ above the table that detects the X-rays. The arm will move across your body above you.
Physical function tests	After a snack, you will do a few tests that will measure hand grip strength, balance, time it will take you to walk 4 metres and time it takes for you to rise from a chair.

For peer review only - http://bmjopen.bmj.com/site/about/guidelines.xhtml

The schedule of procedures for each visit is listed below:

	For everyone in the study (Group 1 and Group 2)					
**Procedure**	**Prior to visit 1**	**Visit 1**	**Visit 2**	**Visit 3**	**Visit 4**	**Visit 5**
**Duration of in-person visit (hrs:min)**	**At home**	**4:00**	**2:10**	**5:30**	**4:30**	**2:10**
**Timeline**			Done within 14 days of visit 1	12 weeks after visit 1	24 weeks after visit 1	24 weeks after visit 1
Urine test		X		X	X	
Height, weight, neck, waist and hip measures		X		X	X	
Indirect calorimetry test		X		X	X	
Pregnancy test (if necessary)		X		X	X	
Medication list review		X		X	X	
Blood pressure		X		X	X	
Bioelectrical Impedance Analysis (BIA)		X		X	X	
Fasting, blood test		X		X	X	
Snack given to participant		X			X	
Lunch given to participant				X		
Fitness test		X		X	X	
Wear a device to monitor your activity & sleep	X			X	X	
Food diary	X			X	X	
Online questionnaire (at-home)	X			X	X	
MRI			X			X
DXA			X	X		X
Snack given to participant			X	X		X
Physical function test			X	X		X

**For those in the Group 2 (low calorie diet and exercise group)**													
**Week #**	**0**	**1**	**2**	**3**	**4**	**8**	**10**	**12**	**14**	**16**	**18**	**21**	**24**
Session with study physician or health care professional	x	x	x		x	x		x	x		x		x
Blood pressure, weight and fasting glucose measure		x	x		x	x		x	x		x		
Blood glucose monitoring (finger prick testing)	x	x	x	x	x	x	x	x	x	x	x	x	x
Sessions with the dietitian	x	x	x		x	x		x	x	x	x	x	x
Supervised exercise training*				x	x	x	x	x					
Home-based exercise**									x	x	x	x	x
CGM sensor	x	x	x	x				x***					x***

For peer review only - http://bmjopen.bmj.com/site/about/guidelines.xhtml

*= At least two supervised exercise sessions weekly from weeks 3 to 12.
** At least one unsupervised/home-based exercise session weekly from weeks 3 to 12 and three sessions weekly or equivalent from weeks 13 through 24
*** Each time you will wear it for two weeks (weeks 11 and 12; weeks 23 and 24)

BENEFITS ASSOCIATED WITH THE RESEARCH STUDY

You may or may not personally benefit from your participation in this research project. However, we hope that the study results will contribute to the advancement of scientific knowledge in the study field and help us find better treatments for patients.

RISKS ASSOCIATED WITH THE RESEARCH STUDY

A possible risk associated with this study is a breach of confidentiality or use of your personal information by a third party. To limit this risk, we will take the steps to protect your confidentiality described in the Confidentiality section, below.

1. **Risks associated with the research intervention (group 2 only)**

Physical activity and exercise: With exercise, there are risks of injury to joints, bones, and muscles. If you wind up in the low energy diet and structured exercise group, during the supervised exercise sessions, you will receive advice on how to be safely active. You will participate in some exercises and may try out some gym equipment. Becoming physically active and exercising may sometimes lead to injury or bring to light health problems that were not obvious. You must inform study personnel and physician of any symptoms of shortness of breath, chest discomfort, or other pain. The study physician may have to further evaluate you. Our exercise specialists will ask you about any joint, muscle, or back problems that you may have. They will help you to tailor your exercise accordingly. It is your responsibility to follow their advice and inform them of any concerns you may have.

Low energy diet: In past studies, the low energy diet has led to symptoms like constipation (18 to 47%), dizziness (4 to 32%), fatigue (11 to 25%), thirst (6%), and/or headache (8%). These tend to get better with fibre-based laxatives and time. We will be giving you a fibre-based laxative to use as needed. It is also important to drink as much water as needed when on the diet.

Stopping medications: We will be stopping your diabetes and blood pressure medications at the beginning of the diet and exercise program. This will help to prevent drops in blood pressure and/or glucose that would happen if the low energy diet were combined with medications. Diet and exercise may lead to low blood sugar and low blood pressure, but this is unlikely when not taking medication. We will monitor your blood sugar and blood pressure levels with you during the study to see if at any point medications need to be restarted.

2. **Risks associated with research procedures.**

Blood tests: The taking of blood samples may cause some discomfort, fainting, formation of a small blood clot or swelling of the vein on surrounding tissue, bleeding from the puncture site, and /or rarely an infection. There is a possibility that you may faint. However, precautions will be taken to ensure your safety should this occur.

For peer review only - http://bmjopen.bmj.com/site/about/guidelines.xhtml

Treadmill testing: As with any type of strenuous activity, there is a some risk of a serious event (e.g. heart attack, hypoglycemia) during the exercise tests that you will perform during the study. If at any time during the exercise tests you do not wish to continue for any reason, you may stop exercising. A health professional and physician will always be available during the treadmill test and emergency equipment will always be available in case of a serious event.

Body composition DXA scans: These procedures like X-rays use radiation energy to form images of your body and provide your doctor with other clinical information. Radiation can cause cell damage that may, after many years or decades, turn cancerous. We are all at risk of developing cancer during our lifetime. The normal risk is that this will happen to about 50% of people at some point in their life. Taking part in this study will add only a very small chance of this happening to you.

MRI: We will not perform an MRI on pregnant women. The magnetic field of the MRI may pose a risk to the developing fetus. The MRI table, on which you will lie on, will slide inside the MRI scanner. Less than 5% of people have anxiety or a feeling of claustrophobia because of the small space that they are in during the test. The scanner will emit radio frequencies that generate a loud noise, which will be lessened by the headphones we will give you to wear. During the exam, you can talk to the technologist or you can ask to stop the exam at any time. About 10% of people have headache or nausea related to the strong magnetic field of the MRI; these are usually mild and they go away. Some people may also experience a sensation of heat, tingling in the body, nausea and discomfort due to the loud noise.

Questionnaires: Some questions may upset some participants. For example, we will ask you about use of drugs and alcohol, past history of miscarriage, and symptoms of pain, anxiety, and depression. Please let us know if this upsets you and how we can help to support you. We may be able to help you find online resources or places to seek counselling, if necessary. We could also assist you in communicating any issues to your doctor.

Glucose sensors: There is a low risk of infection or bruising from the insertion of the sensor. These could last for a few days. The skin of some individuals is sensitive to the adhesive and can get red or itchy when the CGM is attached. There may be redness in the area where the tape was applied but this will usually disappear after a few days.

RISKS ASSOCIATED WITH PREGNANCY

The approach we are using has not been tested for safety in pregnant women. Therefore, it is important not to become pregnant during the study. In addition, if you are sexually active, and could become pregnant you must use a medically accepted contraceptive method throughout your participation in the study.

The medically accepted contraceptive methods are oral contraception, hormonal implants, hormonal patches, IUD, diaphragm and spermicide, cervical cap with spermicide, and condom with spermicide. The study doctor or the research team will discuss your contraceptive method with you to ensure that it is medically accepted.

If you suspect that you have become pregnant during your participation in the research study, you must inform the study staff immediately. The study doctor and staff will help arrange testing and

For peer review only

For peer review only - http://bmjopen.bmj.com/site/about/guidelines.xhtml

referral, as appropriate. If you are in Group 2, we will stop the low energy diet and supervised exercise program. For either Group 1 or Group 2, we will not do the other test procedures like MRI or treadmill testing.

VOLUNTARY PARTICIPATION AND THE RIGHT TO WITHDRAW

Your participation in this research project is voluntary. Therefore, you may refuse to participate. You may also withdraw from the project at any time, without giving any reason, by informing the study doctor or a member of the research team.

Your decision not to participate in the study, or to withdraw from it, will have no impact on the quality of care and services to which you are otherwise entitled, or on your relationship with the study doctor or clinical team.

The study doctor, the Research Ethics Board, the funding agency, or the Sponsor may put an end to your participation without your consent. This may happen if new findings or information indicate that participation is no longer in your interest, if you do not follow study instructions, or if there are administrative reasons to terminate the project.

If you withdraw or are withdrawn from the study, the information and biological material already collected for the study will be stored, analyzed and used to ensure the integrity of the study.

Any new findings that could influence your decision to stay in the research project will be shared with you as soon as possible.

CONFIDENTIALITY

During your participation in this study, the study doctor and their team will collect and record information about you in a study file. They will only collect information required to meet the scientific goals of the study.

The study file may include information from your medical chart, including your identity, concerning your past and present state of health, your lifestyle, as well as the results of the tests, exams, and procedures that you will undergo during this research project. Your research file could also contain other information, such as your name, sex, date of birth and ethnic origin.

All the information collected during the research project will remain strictly confidential to the extent provided by law. You will only be identified by a code number. The key to the code linking your name to your study file will be kept by the study doctor.

To ensure your safety, a copy of this information and consent form will be placed in your medical chart. As a result, any person or company to whom you give access to your medical chart will have access to this information.

For peer review only

For peer review only - http://bmjopen.bmj.com/site/about/guidelines.xhtml

We are conducting this study in collaboration with researchers in the United Kingdom (UK) and Canada. We may share the data collected with them, without any identifying information. This is so we can analyse the results from all the sites participating in this study. This will be done in such a way that you cannot be recognised from it. For example, shared data will not include names, addresses or dates of birth.

The study data will be stored for 25 years after completion of the trial.

The data may be published or shared during scientific meetings; however, precautions will be taken to ensure that it will not be possible to identify you.

You have the right to consult your study file in order to verify the information gathered, and to have it corrected if necessary.

However, in order to protect the scientific integrity of the research project, accessing certain information before the project is ended may require that you be withdrawn from the study.

For auditing purposes, the research study files which could include documents that may identify you may be examined by a person mandated by the study sponsor, the institution, or the Research Ethics Board. All these individuals and organizations adhere to policies on confidentiality.

INCIDENTAL FINDINGS

Material incidental findings are findings made in the course of the study that may have significant impacts on your current or future wellbeing or that of your family members. A material incidental finding concerning you in the course of this research will be communicated to you and to a health professional of your choice.

FUNDING OF THE RESEARCH PROJECT

The researcher and the institution have received funding from the Canadian Institutes of Health Research to conduct this research project.

COMPENSATION

You will receive an amount of $10 per **visit to the Glen hospital** (to cover parking and public transit costs) for each of the 5 study-related visits (see Table titled ***For everyone in the study (Group 1 and Group 2)***).

For Group 2, parking costs at PERFORM will be paid directly by the study team to the PERFORM centre. The meal replacements and supervised exercise sessions (where applicable) will be offered to you for free for the duration of this research study.

SHOULD YOU SUFFER ANY HARM

Should you suffer harm of any kind due to a procedure related to the research study, you will receive the appropriate care and services required by your state of health.

By agreeing to participate in this research project, you are not waiving any of your legal rights nor discharging the study doctor, the sponsor or the institution, of their civil and professional responsibilities.

For peer review only - http://bmjopen.bmj.com/site/about/guidelines.xhtml

WHAT ABOUT COVID-19?

Your safety and that of our research team will be paramount. The study will comply with the latest recommendations and laws. This includes physical distancing and the use of personal protective equipment. If things change during the course of the study, we may need to change how we run the study. This may mean greater use of remote consultations and monitoring. The amount of exercise done at home may also need to be increased.

CLINICAL TRIAL REGISTRATION

A description of this clinical trial will be available on http://www.ClinicalTrials.gov. This Website will not include information that can identify you. At most, the Website will include a summary of the results. You can search this Website at any moment.

CONTACT INFORMATION

If you have questions or if you have a problem you think may be related to your participation in this research study, or if you would like to withdraw, you may communicate with:

- The Principal Investigator, Dr. Kaberi Dasgupta, at the McGill University Health Centre, at 514-934-1934, then 1 then ext. 44715; or
- The main research coordinator of the study, Ms. Debbie Chan, at 514-934-1934 then 1 then ext. 44835 or by email at debbie.chan@rimuhc.ca

For any question concerning your rights as a research participant taking part in this study, or if you have comments, or wish to file a complaint, you may communicate with McGill University Health Centre Ombudsman: 514-934-1934, then 1 then ext. 35655

OVERVIEW OF ETHICAL ASPECTS OF THE RESEARCH

The McGill University Health Centre Research Ethics Board reviewed this research and is responsible for monitoring the study.

For peer review only

For peer review only - http://bmjopen.bmj.com/site/about/guidelines.xhtml

SIGNATURES

Signature of the participant

I have reviewed the information and consent form. Both the research study and the information and consent form were explained to me. My questions were answered, and I was given sufficient time to make a decision. After reflection, I consent to participate in this research study in accordance with the conditions stated above.

I authorize the research study team to have access to my medical record for the purposes of this study.

I authorize the study doctor to inform my treating physician that I am taking part in this study.

I authorize the doctor in charge of this research study to communicate with me directly to ask if I am interested in participating in other research

I understand that my doctor may be contacted to provide information about my health status to the research team for confirmation of eligibility.

Name and contact information of treating physician: ______________________________

Physician's Address: _________________________________

Physician's phone number:________-_________-____________

I understand that the study doctor will send my treating physician health information if it will be useful for my care.

____

Name of participant Signature Date (dd/mmm/yyyy)

Signature of the person obtaining consent

I have explained the research study and the terms of this information and consent form to the research participant, and I answered all his/her questions.

____

Name of the person obtaining consent Signature Date (dd/mmm/yyyy)

For peer review only - http://bmjopen.bmj.com/site/about/guidelines.xhtml

For peer review only

RESET 4 REMISSION Trial DMC Charter

**1. INTRODUCTION**	
Name (and sponsor's ID) of trial plus ISRCTN and/or EUDRACT number	REmission of diabetes and improved diastolic function by combining Structured Exercise with meal replacemenT and food reintroduction: THE RESET FOR REMISSION TRIAL<br>Sponsor: University of Leicester and McGill University<br>Registration number: *To be included*
Objectives of trial, including interventions being investigated	**Primary objective**<br>The aim of this efficacy study is to investigate whether combining a low energy diet incorporating the phased use of meal replacement products, with structured exercise training leads to remission of T2DM in younger adults (18-40 years) over a 24-week period, in comparison with usual care. Remission is defined as an hba1c value less than 6.5% at 24 weeks and no antihyperglycemic medications during weeks 13 through 24 weeks of the 24 week study period.<br><br>**Secondary objectives**<br>Key secondary objectives will examine the effects of the combined intervention on cardiovascular and functional health, particularly MRI-assessed diastolic function and cardiometabolic risk factors such as lipid profile and peripheral blood pressure, along with cardiorespiratory fitness, physical function, lean mass and basal metabolic rate. We will also undertake a process evaluation, to ensure the findings from our research can be used to inform how the interventional components are refined and translated.<br><br>Figure 1 shows a flow chart of the trial design.
Outline of scope of charter	The purpose of this document is to describe membership, terms of reference, roles, responsibilities, authority, decision-making and relationships of the independent Data Monitoring Committee (DMC) for the RESET 4 REMISSION trial. This will include the timing of meetings, methods of providing information to and from the DMC, frequency and format of meetings and relationships with other trial committees such as the Trial Steering Committee (TSC).
**2. ROLES AND RESPONSIBILITIES**	
A broad statement of the aims of the Data Monitoring Committee	To safeguard the interests of trial participants, assess the safety of the interventions during the trial, and monitor the overall conduct of the clinical trial.
Terms of reference	The DMC should receive and review the progress and accruing data of this trial and provide advice on the conduct of the trial to the Trial Steering Committee.
Specific roles of DMC	To undertake reviews of the trial's progress, including updated figures on recruitment, data quality, and main outcomes and safety data, by:<br>• assess data completeness (and by so doing encourage collection of high-quality data)<br>• monitor recruitment figures and losses to follow-up<br>• monitor compliance with the protocol by participants and investigators<br>• monitor evidence for any potential treatment harm (e.g., Serious Adverse Events or deaths)

For peer review only - http://bmjopen.bmj.com/site/about/guidelines.xhtml

RESET 4 REMISSION Trial DMC Charter

	• suggest additional data analyses as part of the review of the statistical analysis plan<br>• advise on protocol modifications suggested by investigators or sponsors (e.g., to inclusion criteria, trial endpoints, or sample size)<br>• monitor planned sample size assumptions<br>• monitor continuing appropriateness of participant information sheets<br>• monitor compliance with previous DMC recommendations<br>• assess the impact and relevance of external evidence as requested by the TSC, Trial Sponsors (University of Leicester and Research Institute of the McGill University Health Centre [RI-MUHC]) or Trial Funders (The UK Medical Research Council [MRC] and The Canadian Institutes of Health Research [CIHR])<br>• if funding is required above the level originally requested, the DMC may be asked by the Principal (Chief) Investigators, TSC, Trial Sponsors or Trial Funders to provide advice and, where appropriate, information on the data gathered to date in a way that will not compromise the trial.
**3. BEFORE OR EARLY IN THE TRIAL**	
Whether the DMC will have input into the protocol	All potential DMC members should read the protocol/outline before agreeing to join the committee. Before recruitment begins the trial will have undergone review by the funders (UK Medical Research Council and Canadian Institutes of Health Research), the sponsors (University of Leicester and RI-MUHC), and other relevant trial committees including that of research ethics committees. Therefore, if a potential DMC member has major reservations about the trial (e.g., the protocol or the logistics) they should report these to the Chief Investigators and may decide not to accept their invitation to join. DMC members should be constructively critical of the ongoing trial, but also supportive of aims and methods of the trial.
Whether the DMC will meet before the start of the trial	It is recommended that, if possible, the DMC meets before the trial starts or early in the course of the trial, to discuss the protocol, the trial, any analysis plan, future meetings, and to have the opportunity to clarify any aspects with the principal investigators.
Any issues specific to the disease under study	The study population comprises younger (18 – 40 years) adults with type 2 diabetes. These individuals are at risk of developing chronic (e.g., cardiovascular disease) or acute (e.g. hyperglycaemia) complications related to their diabetes. The protocol lists the possible adverse events of study interventions within this population.
Any specific regulatory issues	None
Any other issues specific to the treatment under study	None
Whether members of the DMC will have a contract	DMC members will not be asked to formally sign a contract but should formally register their agreement to join the group by confirming; (1) that they agree to be a member of the DMC and (2) that they agree with the contents of this Charter. Any potential competing interests, real or potential, should be declared at the same time using a short competing interest form (displayed in **Annex 1**), which should be completed and returned by the DMC members to the trial coordinating centre. DMC members should sign and maintain this log of potential competing interests.

For peer review only - http://bmjopen.bmj.com/site/about/guidelines.xhtml

RESET 4 REMISSION Trial DMC Charter

**4. COMPOSITION**	
Membership and size of the DMC	Members of the DMC (including the DMC Chair) should be independent of the trial. The definition of independent is as follows:<br>• Not part of the same institution as any of the applicants or members of the project team<br>• Not part of the same institution that is acting as a recruitment or investigative centre<br>• Not related to any of the applicants or members of the project team<br>• For the chair only – not an applicant on a rival proposal<br>The membership will consist of three individuals including two clinicians experienced in the clinical area and one statistician. Members have been chosen because they are experienced in trials and/or the disease area.<br>The members of the DMC for the RESET 4 REMISSION Trial are:<br>(1) **Dr Janusz Kaczorowski** *(Chair)*, Professor of Family and Emergency Medicine, University of Montreal<br>(2) **Mr Stephen Sharp** (*Statistician*), MRC Epidemiology Unit, University of Cambridge School of Clinical Medicine<br>(3) **Dr Andrew Farmer** (*Clinical Representative*), Professor of General Practice, Nuffield Department of Primary Care Health Sciences, University of Oxford
The Chair, how they are chosen and the Chair's role. (Likewise, if relevant, the vice-Chairman)	The chair is Dr. Janusz Kaczorowski (Professor and Research Director in the Department of Family and Emergency Medicine – University of Montreal) who is serving on the DMC. The Chair is expected to facilitate and summarise discussions. There is no vice-Chair.
The responsibilities of the DMC statistician	The DMC membership includes a statistician (Mr Stephen Sharp) whose role it is to provide independent statistical expertise and to further guide the other DMC members through the report. The DMC statistician is not expected to prepare the DMC report.
The responsibilities of the trial statistician	The trial statistician (Dr Elham Rahme) will produce (or oversee the production of) the report to the DMC and will participate in DMC meetings, guide the DMC through the report, may participate in DMC discussions and, on some occasions, taking notes.
The responsibilities of the trial office team	The trial management team (e.g., Trial Managers/Coordinators) will input to the production of the DMC report.
The responsibilities of the PI and other members of the Trial Management Group (TMG)	The PI's (Professor Thomas Yates and Professor Kaberi Dasgupta), may be asked, and should be available, to attend open sessions of the DMC meeting. The other TMG members will not usually be expected to attend but can attend open sessions when necessary (see Section 6 regarding 'Organisation of DMC Meetings' below).
**5. RELATIONSHIPS**	
Relationships with Principal Investigators, other trial committees (eg Trial Steering Committee (TSC) or Executive Committee), sponsor and regulatory bodies	The DMC are advisory to the Trial Steering Committee (which includes the PI's). The TSC is the executive body for the trial.
Clarification of whether the DMC is	The DMC is advisory to the Trial Steering Committee. The TSC is the executive

For peer review only - http://bmjopen.bmj.com/site/about/guidelines.xhtml

RESET 4 REMISSION Trial DMC Charter

advisory (make recommendations) or executive (make decisions)	body for the trial.
Payments to DMC members	Members will be reimbursed for reasonable travel costs and other expenses incurred, but it is anticipated that the meetings with be virtual. No other payments or rewards would be given to professional members.
The need for DMC members to disclose information about any competing interests	Competing interests should be disclosed. These are not restricted to financial matters – involvement in other trials or intellectual investment could be relevant. Although members may well be able to act objectively despite such connections, complete disclosure enhances credibility (See **Annex 1** towards end of DMC Charter document).<br><br>DMC members should not use interim results to inform trading in pharmaceutical shares, and careful consideration should be given to trading in stock of companies with competing products. Changes in declarations of real or potential competing interests should be documented within the minutes at the start of each meeting.
**6. ORGANISATION OF DMC MEETINGS**	
Expected frequency of DMC meetings	The DMC should meet 6-monthly to annually, or more often as appropriate either in person or via teleconference. Some trial issues may need to be dealt with between meetings, by phone or by email. DMC members should be prepared for such instances. Additional *ad hoc* meetings will be scheduled with the occurrence of any severe adverse event including death, hospitalization, or emergency room consultation. During these meetings, reports from the PIs will be reviewed, regarding the SAE in question and the relatedness to trial procedures. The DSC will then determine whether further adjudication is warranted and what type of expertise is required; in these instances, two physicians from the relevant research site will be invited to review the event and medical records and comment on relatedness. If there is a difference of opinion, a third physician will be invited to review. The physicians may be trial investigators but they must be blinded to the trial arm and cannot have interacted with the participant during the course of the trial.<br><br>Regularly-scheduled meetings should be timed approximately 1 month prior to TSC meetings, so that reports can be fed to the TSC accordingly.
Whether meetings will be face-to-face or by teleconference	Given the international context, the default will be to hold meetings over videoconference. Effort will be made to ensure that all members can attend.
How DMC meetings will be organised, especially regarding open and closed sessions, including who will be present in each session	Upon request by the DMC, the format will be an open session throughout where all parts of the report are discussed and there is DMC discussion. The DMC members can ask for a closed session at any point in the DMC (± trial statistician at discretion of the DMC chair).
**7. TRIAL DOCUMENTATION AND PROCEDURES TO ENSURE CONFIDENTIALITY AND PROPER COMMUNICATION**	
Intended content of material to be available in sessions	Accumulating information relating to recruitment and retention, and data quality (e.g., data return rates, treatment compliance) will be presented by intervention arm. No formal interim analyses are planned A log of adverse events will be presented at each meeting and discussed. Outcome data will not be presented. However, if the DMC has any concerns related to safety, pooled outcome data may be presented, and, in discussion with the TSC, unblinding may be permitted.

For peer review only - http://bmjopen.bmj.com/site/about/guidelines.xhtml

RESET 4 REMISSION Trial DMC Charter

Will the DMC be blinded to the treatment allocation	The DMC will not be blinded to recruitment data by treatment arm. No formal interim analyses are planned. The DMC will remain blinded unless safety concerns arise. However, primary and secondary outcome data will be based on pooled data, and the DMC will remain blinded unless unblinding is requested related to concerns about safety.
Who will see the accumulating data	There are no planned interim analyses. The accumulating data presented to the DMC by intervention arm will relate only to attrition and to safety. If the DMC deems that safety concerns then unblinding may occur as indicated above. Interim data and analyses by intervention/control group (and the deliberations of the DMC) should be available only to those present in the DMC meetings when the discussions occurred: i.e., only members of the DMC, the trial statistician and other members of the trial team, as agreed by the DMC. DMC members must not share confidential information with people outside the DMC.
Who will be responsible for identifying and circulating external evidence (e.g., from other trials/ systematic reviews)	Identification and circulation of external evidence (e.g., from other trials/ systematic reviews) is not the responsibility of the DMC members. It is the responsibility of the TMG (Trial Management Group). However, the DMC should continue to be made aware of other data that may impact the trial.
To whom the DMC will communicate the decisions/ recommendations that are reached	The DMC will report its recommendations via the chair in writing to the Trial Steering Committee (TSC, please see **Annex 2**). This should be sent in time for consideration at a TSC meeting. If the trial is to continue largely unchanged then it is often useful for the report from the DMC to include a summary paragraph suitable for trial promotion purposes.
Whether reports to the DMC be available before the meeting or only at/during the meeting	It is planned that the DMC will receive data monitoring reports (i.e., recruitment, attrition, numbers in various study stages, numbers completed) from the TMG at least 2 weeks before scheduled meetings.
What will happen to the confidential papers after the meeting	The DMC members should store the papers safely after each meeting so they may check the next report against them. After the trial is reported, the DMC members should destroy all interim reports.
**8. DECISION MAKING**	
What decisions/recommendations will be open to the DMC	Possible recommendations to the TSC could include:<br>• No action needed; trial continues as planned<br>• Early stopping due, for example, to clear harm of a treatment or external evidence (this should generally involve a recommendation from the DMC to unblind the TSC to this data)<br>• Modifying target recruitment, or pre-analysis follow-up, based on any change to the assumptions underlying the original trial sample size calculation (but not on any emerging differences)<br>• Sanctioning and/or proposing protocol changes<br><br>Ultimately, decisions will be made by the TSC, as the DMC's role is advisory.
The role of formal statistical methods, specifically which methods will be used and whether they will be used as guidelines or rules	The DMC and TSC will be asked to comment on and approve the trial statistical analysis plan (SAP) before database lock.
How decisions or recommendations will be reached within the DMC	Every effort should be made to achieve consensus. The role of the DMC Chair is to summarise discussions and encourage consensus; therefore, it is usually best for the Chair to give their own opinion last.

For peer review only - http://bmjopen.bmj.com/site/about/guidelines.xhtml

RESET 4 REMISSION Trial DMC Charter

	It is important that the implications (e.g., ethical, statistical, practical, financial) for the trial be considered before any decision is made. Only appointed members will be entitled to vote and the chair will have a casting vote.
When the DMC is quorate for decision-making	Effort should be made for all DMC members to attend. The TMG will try to ensure that a date is chosen to enable this. Members who cannot attend in person should be encouraged to attend by teleconference. If, at short notice, any DMC members cannot attend at all then the DMC may still meet if at least two members will be present (including the Chair). If the DMC is considering recommending major action after such a meeting, the DMC Chair should talk with the absent members as soon after the meeting as possible to check whether they agree with such action. If they do not, a further teleconference should be arranged with the full DMC.
Can DMC members who cannot attend the meeting input	If the report is circulated before the meeting, DMC members who will not be able to attend the meeting may pass comments to the DMC Chair for consideration during the discussions.
What happens to members who do not attend meetings	If a member does not attend a meeting, it should be ensured that the member is available for the next meeting. If a member does not attend a second meeting, they should be asked if they wish to remain part of the DMC. If a member does not attend a third meeting, they should be replaced.
Whether different weight will be given to different endpoints (e.g., safety/efficacy)	This study is unlikely to result in safety issues, therefore greater weight should be given to efficacy considerations.
Any specific issues relating to the trial design that might influence the proceedings, eg cluster trials, equivalence trials, multi-arm trials	This is a 2-arm trial, occurring in the UK (Leicester) and Canada (Montreal, Edmonton). It is likely that the TSC/ DMC will need to consider issues specific to UK or Canadian sites in equivalent trials.
**9. REPORTING**	
To whom will the DMC report their recommendations/decisions, and in what form	The DMC Chair should report in writing to the Chair of the Trial Steering Committee, usually within 3 weeks after the meeting. Unless the DMC is recommending that the trial protocol be changed in some way, the letter to the TSC should not reveal any confidential information. An example of a letter from a DMC to the TSC recommending no action is presented in **Annex 2**. Additionally, the letter should be copied to the PIs, trial statistician and trial managers.
Whether minutes of the meeting be made and, if so, by whom and where they will be kept	Minutes including key points and actions will be made by a member of the trials' office team (to be decided prior to each meeting). This will include details of whether any competing interests have arisen/changed for any attendees since the previous meeting. The draft minutes will be initially circulated for comment to all DMC members who were present at the meeting. The DMC Chair will then sign off the final version of minutes. The final signed version will then be sent to all members, the sponsor, the funder and stored in the trial master file.
What will be done if there is disagreement between the DMC and the body to which it reports	The TSC has ultimate responsibility for the trial and assumes primacy. However, the TSC should report to the DMC regarding how they have acted upon the DMC's recommendations. If the DMC has serious problems or concerns with the TSC decision, a joint meeting of the TSC and DMC should be held. The information to be shown would depend upon the action proposed and each committee's concerns. The meeting would be Chaired by

For peer review only - http://bmjopen.bmj.com/site/about/guidelines.xhtml

RESET 4 REMISSION Trial DMC Charter

	a senior member of the trials office staff or an external expert who is not directly involved with the trial.
**10. AFTER THE TRIAL**	
Publication of results	The PIs have responsibility that trial results will be published in a correct and timely manner. The TSC is the committee that should oversee this process.
The information about the DMC that will be included in published trial reports	DMC members will be named and their affiliations listed in the main report, unless they explicitly request otherwise.
Whether the DMC will have the opportunity to approve publications, especially with respect to reporting of any DMC recommendation regarding termination of a trial	The DMC members should be given at least 2 weeks to read and comment on any draft publications that report the primary outcome measure and/or details of the DMC. This may be done simultaneously to other groups reviewing the draft manuscript (e.g. Trial Steering Committee, trial investigators).
Any constraints on DMC members divulging information about their deliberations after the trial has been published	The DMC should not discuss confidential issues from their involvement in the trial until 12 months after the primary trial results have been published, unless permission is agreed with the TSC. The TSC will also decide when it is appropriate for TMG and DMC members to trade in related stocks.

For peer review only

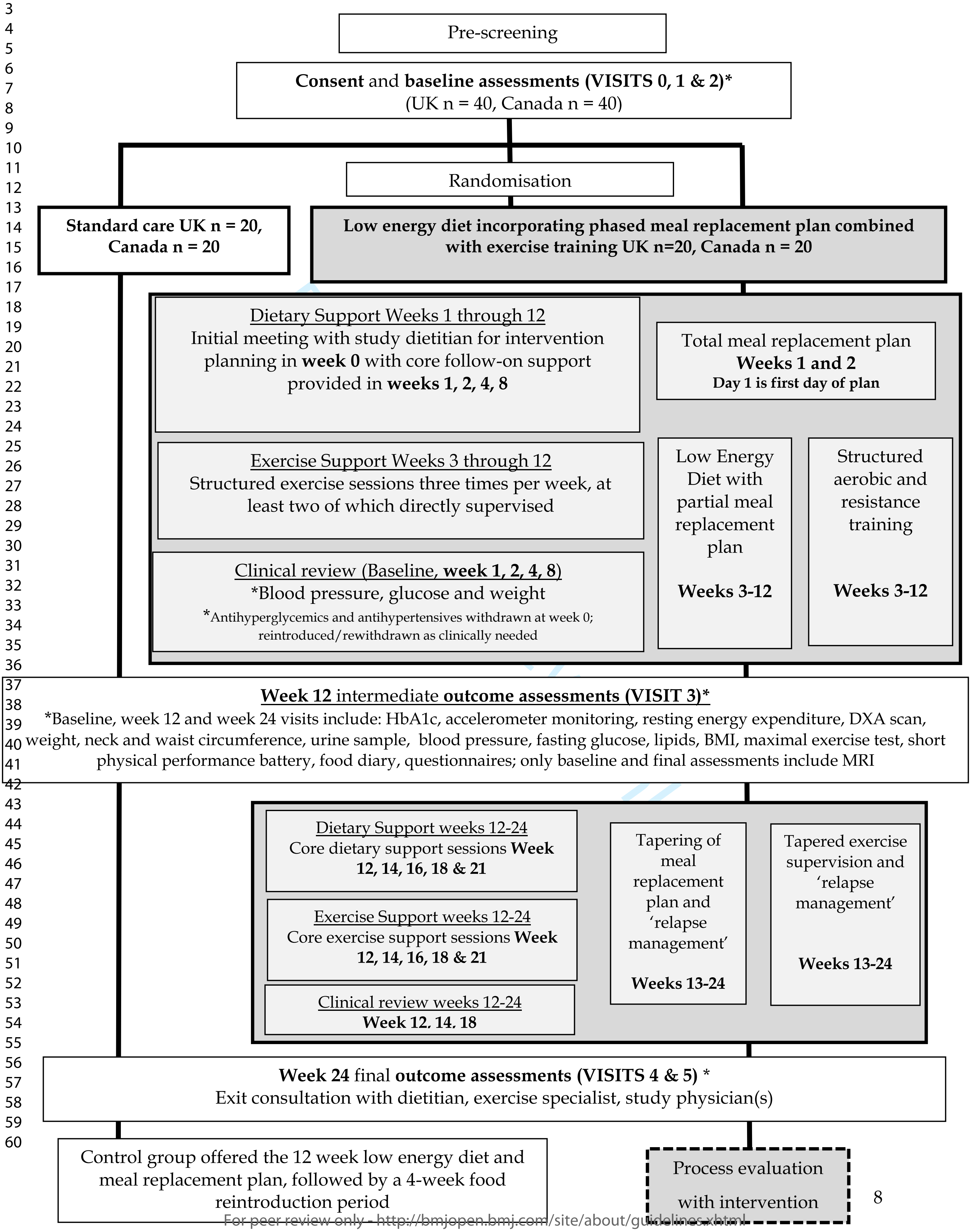

For peer review only - http://bmjopen.bmj.com/site/about/guidelines.xhtml

Annex 1: Suggested competing interests form

RESET 4 REMISSION Trial (Sponsor: University of Leicester and McGill University): Agreement to join the Data Monitoring Committee as an independent member and disclosure of potential competing interests

Please complete the following document and return to the Trials Office

(Please initial box to agree)

☐ I have read and understood the DMC Charter version 1.0, dated 15th March 2021

☐ I agree to join the Data Monitoring Committee for this trial as an independent member

☐ I agree to treat all sensitive trial data and discussions confidentially

The avoidance of any perception that independent members of a DMC may be biased in some fashion is important for the credibility of the decisions made by the DMC and for the integrity of the trial.

Potential competing interests should be disclosed via the trials office. In many cases simple disclosure up front should be sufficient. Otherwise, the (potential) independent DMC member should remove the conflict or stop participating in the DMC. **Table 1** lists potential competing interests.

☐ **No,** I have no potential competing interests to declare

☐ **Yes,** I have potential competing interests to declare (please detail below)

Please provide details of any potential competing interests:

____

____

____

Name: ____________________________

Signed: ___________________________ Date: ______________

Table 1: Potential competing interests for independent members

- Stock ownership in any commercial companies involved
- Stock transaction in any commercial company involved (if previously holding stock)
- Consulting arrangements with the Sponsor/Funder
- Ongoing advisory role to a company providing drugs to the trial
- Frequent speaking engagements on behalf of the intervention
- Career tied up in a product or technique assessed by trial
- Hands-on participation in the trial
- Involvement in the running of the trial
- Emotional involvement in the trial
- Intellectual conflict e.g. strong prior belief in the trial's experimental arm
- Involvement in regulatory issues relevant to the trial procedures
- Investment (financial or intellectual) or career tied up in competing products
- Involvement in the writing up of the main trial results in the form of authorship

For peer review only - http://bmjopen.bmj.com/site/about/guidelines.xhtml

Annex 2: Suggested report from DMC to TSC where no recommendations are being made

[Insert date]

To: Professor XX (Chair of Trial Steering Committee)

Dear Professor XX,

The Data Monitoring Committee (DMC) for the RESET 4 REMISSION Trial met on *[meeting date]* to review its progress and interim accumulating data. *[List members] attended* the meeting and reviewed the report.

We congratulate the trial organisers and collaborators on the progress and conduct of the trial and the presentation of the data. The trial question remains important and, on the basis of the data reviewed at this stage, we recommend continuation of the trial according to the current version of the protocol *[specify protocol version number and date]* with no changes.

We shall next review the progress and data *[provide approximate timing]*

Yours sincerely,

Professor XX
Chair of Data Monitoring Committee

On behalf of the DMC (all members listed below)

DMC members:
(1) xx
(2) xx
(3) xx

For peer review only

TSC Charter for R4R

Author: Kaberi Dasgupta and Thomas Yates

Date: 13/01/2021

Version: 1.0

RESET FOR REMISSION TRIAL TSC CHARTER

1. INTRODUCTION	
Name (and sponsor's ID) of trial plus ISRCTN and/or EUDRACT number	REmission of diabetes and improved diastolic function by combining Structured Exercise with meal replacemenT and food reintroduction: THE RESET FOR REMISSION TRIAL<br>Sponsor: University of Leicester and McGill University<br>Registration number: *To be included*
Objectives of trial, including interventions being investigated	**Primary objective**<br>The aim of this efficacy study is to investigate whether combining a low energy diet incorporating the phased use of meal replacement products, with structured exercise training leads to remission of T2DM in younger adults (18-40 years) over a 24 week period. Remission is defined as an HbA1c value less than 6.5% without antihyperglycemic medications during weeks 13 through 24 weeks of the 24 week study period.<br><br>**Secondary objectives**<br>Key secondary objectives will examine the effects of the combined intervention on cardiovascular and functional health, particularly MRI-assessed diastolic function and cardiometabolic risk factors such as lipid profile and peripheral blood pressure, along with cardiorespiratory fitness, physical function, lean mass and basal metabolic rate. We will also undertake a process evaluation, to ensure the findings from our research can be used to inform how the interventional components are refined and translated.<br><br>Figure 1 shows a flow chart of the trial design.
Outline of scope of charter	The purpose of this document is to describe the membership, terms of reference, roles, responsibilities, authority, decision-making and relationships of the Trial Steering Committee (TSC) for this trial, including the timing of meetings, methods of providing information to and from the TSC, frequency and format of meetings and relationships with other trial committees.
**2. ROLES AND RESPONSIBILITIES**	
A broad statement of the aims of the TSC	To act as the oversight body for the Reset For Remission Trial on behalf of the Sponsor/Funder.
Terms of reference	The role of the TSC is to provide oversight for the trial. It should also provide advice, through its Chair, to the CIs (PIs), the Sponsors (University of Leicester and Research Institute of the McGill University Health Centre), the Funders (The Canadian Institutes of Health Research [CIHR] and the UK Medical Research Council [MRC]), and the Host Institution on all appropriate aspects of the trial but with a particular focus on:<br>• Progress of the trial, adherence to the protocol, participant safety and the consideration of new information of relevance to the research question.

For peer review only - http://bmjopen.bmj.com/site/about/guidelines.xhtml

TSC Charter for R4R | Author: Kaberi Dasgupta and Thomas Yates
Date: 13/01/2021 | Version: 1.0

RESET FOR REMISSION TRIAL TSC CHARTER	
	• The rights, safety and well-being of the trial participants are the most important considerations and should prevail over the interests of science and society.<br>• To ensure appropriate ethical and other approvals are obtained in line with the project plan<br>• To review proposals for substantial protocol amendments and provide advice to the sponsor and funder regarding approvals of such amendments, where appropriate.<br>• To provide advice to the investigators on all aspects of the trial.
Specific roles of TSC	The specific roles of the TSC as a body will be to:<br>• provide expert oversight of the trial<br>• make decisions as to the future continuation (or otherwise) of the trial<br>• monitor recruitment rates and encourage the Trial Management Group (TMG) to develop strategies to deal with any recruitment problems<br>• review regular reports of the trial from the trials coordinators (sent on behalf of the TMG)<br>• receive letters of feedback from the DMC (Data Monitoring Committee) and consider their recommendations<br>• assess the impact and relevance of any accumulating external evidence (provided by the TMG)<br>• monitor follow-up rates and review strategies from TMG to deal with problems<br>• ensure sites that are complying with the protocol<br>• approve major amendments to the protocol<br>• approve any proposals by the TMG concerning any change to the design of the trial, including additional sub studies<br>• oversee the timely reporting of trial results<br>• comment on the statistical analysis plan<br>• comment on the publication policy<br>• comment on the main trial manuscript<br>• approve and comment on any abstracts and presentations of any results *during* the running of the trial<br>• approve external or early internal requests for release of data or subsets of data or samples including clinical data and stored biological samples<br>It is the responsibility of individual members of the TSC to maintain confidentiality of all trial information that is not already in the public domain.
**3. BEFORE OR EARLY IN THE TRIAL**	
TSC input into the protocol	All potential independent TSC members should have opportunity to comment on the protocol as early as possible. If a potential independent TSC member has major reservations about the trial (e.g., the protocol, the logistics, ethical concerns) they should report these to the TMG and may decide not to accept the invitation to join. TSC members should be constructively critical of the protocol, but also supportive of aims and methods of the trial.
Any issues specific to the disease under study	The study population comprises younger (18 – 40 years) adults with type 2 diabetes. These individuals are at risk of developing chronic (e.g. cardiovascular disease) or acute (e.g. hyperglycaemia) complications related

For peer review only - http://bmjopen.bmj.com/site/about/guidelines.xhtml

RESET FOR REMISSION TRIAL TSC CHARTER

	to diabetes. The protocol lists the possible adverse events of study interventions within this population
Any specific regulatory issues	None
Any other issues specific to the treatment under study	None
Whether members of the TSC will have a contract	TSC members will not be asked to formally sign a contract but should formally register their agreement to join the group by confirming (1) that they agree to be a member of the TSC and (2) that they agree with the contents of this Charter. Any potential competing interests should be declared at the same time. Members should complete and return the form in Annex 1 or 2 by email. Any observers (attendees who are not members) will sign a confidentiality agreement on the first occasion they attend a meeting (Annex 3).
**4. COMPOSITION**	
Membership and size of the TSC	Apart from the studies PIs (Dasgupta, Yates), the members of the TSC, including the Chair, should be independent of the trial. Non-independent members will also be part of the TSC. The definition of independent is as follows:<br>• Not part of the same institution as any of the applicants or members of the project team<br>• Not part of the same institution that is acting as a recruitment or investigative centre<br>• Not related to any of the applicants or members of the project team<br>• For the chair only – not an applicant on a rival proposal<br><br>The membership will consist of three individuals including two clinicians experienced in the clinical area and one statistician. Members have been chosen because they are experienced in trials and/or the disease area.<br>The members of the TSC for this trial are:<br>(1) **Professor Jason Gill** (Chair), Professor of Cardiometabolic Health, University of Glasgow<br>(2) **Dr Alice Cheng** (Clinical representative), Physician and Associate Professor of Medicine, University of Toronto<br>(3) Dr Elham Rahme (Trial statistician)<br>(4) Professor Kaberi Dasgupta and Prof Tom Yates, PIs<br><br>Only appointed members will be entitled to vote. The PIs or their delegates are allocated one vote in total.
The Chair	The chair is Prof Jason Gill (Professor of Cardiometabolic Health at the University of Glasgow) who is serving on the TSC. The Chair is expected to facilitate and summarise discussions. There is no vice-Chair.

For peer review only - http://bmjopen.bmj.com/site/about/guidelines.xhtml

TSC Charter for R4R Author: Kaberi Dasgupta and Thomas Yates

Date: 13/01/2021 Version: 1.0

RESET FOR REMISSION TRIAL TSC CHARTER

	The Chair is directly answerable to the Sponsor and Funders. The Chair's responsibilities include:<br>• Arranging, along with the PIs and trials office, an inaugural meeting to set up a schedule of meetings to align with the project plan<br>• Being familiar with relevant guidance documents and with the role of the DMC<br>• Providing an independent, experienced opinion if conflicts arise between the needs of the research team, the funder, the sponsor, the participating organisations and/or any other agencies<br>• Leading the TSC to provide regular, impartial oversight of the trial, especially to identify and pre-empt problems<br>• Ensuring that changes to the protocol are debated and endorsed by the TSC, letters of endorsement should be made available to the project team when requesting approval from the funder and sponsor for matters such as substantive changes to protocol<br>• Being available to provide independent advice as required, not just when TSC meetings are scheduled<br>• Commenting on any extension requests and, where appropriate, providing a letter of recommendation to accompany such a request<br>• Commenting in detail, when appropriate, regarding the continuation or termination of the project
The responsibilities of the trial office team	The trials office team will produce a short report on the trial before each meeting of the TSC. A template for the report will be followed.
The responsibilities of the PI and other members of the Trial Management Group (TMG)	The PIs is an important member of the TSC and no major decisions should be made without their involvement.
The responsibilities of the observers	Additional observers may be in attendance through (parts of) the TSC meetings in order to provide input on behalf of the trials office, the trial's Sponsor/Funder or to provide specific relevant expertise.
**5. RELATIONSHIPS**	
Advisory and executive bodies	The TSC is the oversight body (see *Roles and responsibilities* above). All substantial issues regarding the trial must go to the TSC for consideration. The DMC is advisory to the TSC.
Payments to TSC members	Members will be reimbursed for reasonable travel costs and other expenses incurred. No other payments or rewards would be given to professional members.
The need for TSC members to disclose information about any competing interests	Competing interests should be disclosed. These are not restricted to financial matters – involvement in other trials or intellectual investment could be relevant. Although members may well be able to act objectively despite such connections, complete disclosure enhances credibility (See Annex 1).<br>TSC members should not use interim results to inform trading in pharmaceutical shares, and careful consideration should be given to trading

For peer review only - http://bmjopen.bmj.com/site/about/guidelines.xhtml

TSC Charter for R4R

Author: Kaberi Dasgupta and Thomas Yates

Date: 13/01/2021

Version: 1.0

RESET FOR REMISSION TRIAL TSC CHARTER

	in stock of companies with competing products. Changes in declarations of real or potential competing interests should be minuted at the start of each meeting.
**6. ORGANISATION OF TSC MEETINGS**	
Expected frequency of TSC meetings	The TSC will meet at 6-monthly to annually depending on need as determined by the Chair, either in person or via teleconference. At the request of the TSC, interim meetings, in person or by teleconference, will be organised. Some trial issues may need to be dealt with between meetings, by phone or by email. TSC members should be prepared for such instances.<br>It is expected that the TSC will meet approximately one month after the DMC.
Attendance of TSC members at meetings	Given the international context, the default will be to hold meetings over videoconference. Effort will be made to ensure that all members can attend. At least one PI must attend all meeting. If, at short notice, any TSC members cannot attend then the TSC may still meet if at least two independent members will be present, along with a PI. There may be occasions when the Trial Sponsors or Funders will wish to organise and administer these meetings.
How TSC meetings will be organised, especially regarding open and closed sessions, including who will be present in each session	Presence will be usually limited to the TSC members, and observers from the TMG. Other attendees may be invited for all or part of the meeting by the TSC Chair. Meeting will be organised by Trials office, in coordination with the PIs and Chair.
Input from TSC members who cannot attend the meeting	If the report is circulated before the meeting, TSC members who will not be able to attend the meeting may pass comments to the TSC Chair or trials office contact for consideration during the discussions.
What happens to independent members who do not attend meetings	If an independent member does not attend a meeting or provide comments when requested between meetings, it should be ensured that the independent member is available for the next meeting. If an independent member does not attend the next meeting or provide comments when next requested, they should be asked if they wish to remain part of the TSC. If an independent member does not attend a third meeting, strong consideration should be given to replacing this member.
**7. TRIAL DOCUMENTATION AND PROCEDURES TO ENSURE CONFIDENTIALITY AND PROPER COMMUNICATION**	
Intended content of material to be considered during meetings	A short report will be prepared by the trial coordinators following a standard template. This will report on accrual and any matters affecting the trial. Additionally, the material may include a report *from* the DMC, requests *from* the TMG or draft publications. No trial outcome measure data will be presented by arm unless explicitly authorised by the DMC (e.g., safety outcomes). If specifically requested by the TSC, accrual, compliance with follow-up and adherence to treatment may be presented by site.
Whether reports to the TSC will be available before the meeting or only at/during the meeting	It is planned that the TSC will receive the report at least 1 week and preferably at least 2 weeks before any meetings.
Responsibility for identifying and	Identification and circulation of external evidence (e.g., from other trials/

For peer review only - http://bmjopen.bmj.com/site/about/guidelines.xhtml

TSC Charter for R4R | Author: Kaberi Dasgupta and Thomas Yates
Date: 13/01/2021 | Version: 1.0

RESET FOR REMISSION TRIAL TSC CHARTER

circulating external evidence (e.g. from other trials/ systematic reviews)	systematic reviews) is not the responsibility of the TSC members; it is a responsibility of the TMG. However, the TSC should continue to be made aware of other data that may impact on a trial.
What will happen to the papers after the meeting	TSC members will be expected to securely store copies of the reports to and from the TSC, agenda and minutes, as well as copies of communications between meetings so they may check the next report against them. All documentation should be considered confidential. After the trial is reported, the TSC members should destroy all interim reports. The trials office will keep a central record of all minutes, reports and correspondence by the TSC.
**8. DECISION MAKING**	
What decisions will be open to the TSC	Based on recommendations from the DMC, possible decisions include:-<br>• No action needed; trial continues as planned.<br>• Early stopping due, for example, to clear harm of a treatment or external evidence (this should generally involve a recommendation from the DMC to unblind the TSC to this data).<br>• Modifying target recruitment, or pre-analysis follow-up, based on any change to the assumptions underlying the original trial sample size calculation (but not on any emerging differences).<br>• Sanctioning and/or proposing protocol changes.<br><br>Based on other factors, possible decisions include the decisions above and:<br>• Censuring centres for poor data quality.<br>• Approving proposed protocol amendments or new trial sub-studies.<br>• Approving requests for early release of (subsets of) data.<br>• Approving presentation of results during the trial or soon after closure.<br>• Approval of new centres or strategies to improve recruitment or follow-up.
The role of formal statistical methods	The TSC will be asked to comment on and approve trial statistical analysis plan (SAP) before database lock.
How decisions or recommendations will be reached within the TSC	Every effort should be made to achieve consensus. The role of the Chair is to summarise discussions and encourage consensus; therefore, it is usually best for the Chair to give their own opinion last.<br>It is important that the implications (e.g., ethical, statistical, practical, financial) for the trial be considered before any decision is made.
When the TSC is quorate for decision-making	At least two independent members of the TSC should be present including the Chair, along with at last one PI
Any specific issues relating to the trial design that might influence the proceedings, eg cluster trials, equivalence trials, multi-arm trials	This is a 2-arm trial, occurring in the UK (Leicester) and Canada (Montreal, Edmonton). It is likely that the TSC will need to consider issues specific to UK or Canadian sites
**9. REPORTING**	
To whom will the TSC report their recommendations/decisions, and in	Reports will be returned to the TMG who will be responsible for implementing any actions resulting. The TSC may also provide feedback to

For peer review only - http://bmjopen.bmj.com/site/about/guidelines.xhtml

RESET FOR REMISSION TRIAL TSC CHARTER

what form	the DMC and, where appropriate, to the Sponsors or Funders.
Whether minutes of the meeting be made and, if so, by whom and where they will be kept	Notes of key points and actions will be made by a member of the trial management team. This will include details of whether potential competing interests have changed for any attendees since the previous meeting. The draft minutes will be initially circulated for comment to those TSC members who were present at the meeting. The TSC Chair will sign off the final version of minutes or notes. The final version will then be sent to all members, the sponsor, the funder and the trial master file.
What will be done if there is disagreement between the TSC and other trial committees	The TSC is the oversight body for the trial. However, the TSC should have good reason before deciding not to accept requests from the TMG and recommendations from the DMC. If there are serious problems or concerns with the TSC decision following a DMC recommendation, a joint meeting of the TSC and DMC should be held. The information to be shown would depend upon the action proposed and each committee's concerns. The meeting would be Chaired by a senior member of the trial management staff or an external expert who is not directly involved with the trial.
**10. AFTER THE TRIAL**	
Publication of results	The TSC will oversee the timely analysis, writing up and publication of the main trial results. The independent members of the TSC will have the opportunity to read and comment on the proposed main publications of trial data prior to submission and abstracts and presentations during the trial. This review may be concurrent to that of the trial investigators and DMC.
The information about the TSC that will be included in published trial reports	TSC members will be named and their affiliations listed in the main report, unless they explicitly request otherwise.
Any constraints on TSC members divulging information about their deliberations after the trial has been published	The TSC may discuss issues from their involvement in the trial 12 months after the primary trial results have been published, or sooner if permission is agreed with the other trial committees and trials office. Similarly, the TSC will decide when it is appropriate for TMG and DMC members to trade in related stocks.

For peer review only - http://bmjopen.bmj.com/site/about/guidelines.xhtml

TSC Charter for R4R

Author: Kaberi Dasgupta and Thomas Yates

Date: 13/01/2021

Version: 1.0

Figure 1: Trial Design

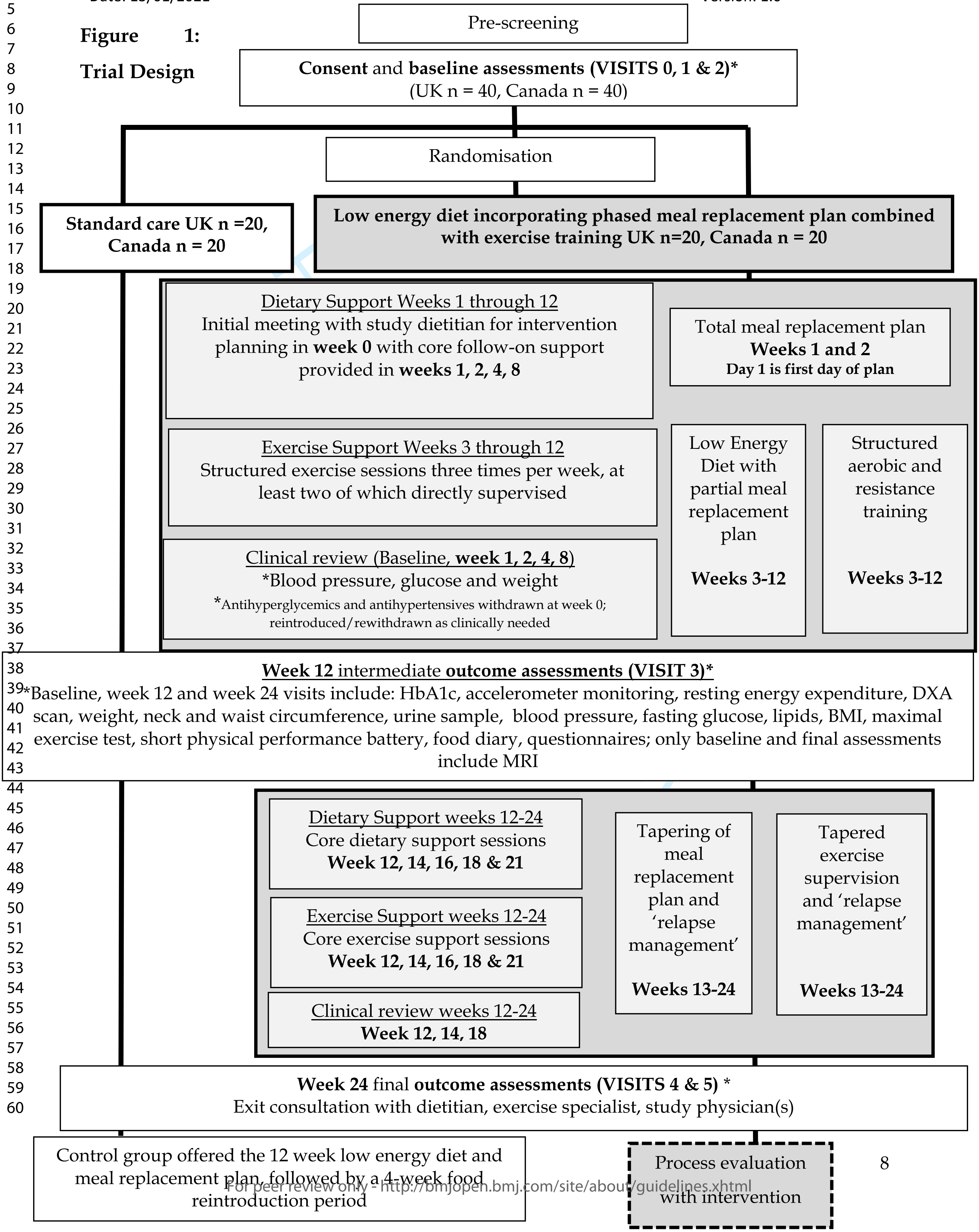

For peer review only - http://bmjopen.bmj.com/site/about/guidelines.xhtml

Annex 1: Agreement and competing interests form for independent members

RESET FOR REMISSION TRIAL: Agreement to join the Trial Steering Committee as an independent member and disclosure of potential competing interests

Please complete the following document and return to the Trials Office

(Please initial box to agree)

☐ I have read and understood the TSC Charter version 1.0, dated (will insert date pending REB approval)

☐ I agree to join the Trial Steering Committee for this trial as an independent member

☐ I agree to treat all sensitive trial data and discussions confidentially

The avoidance of any perception that independent members of a TSC may be biased in some fashion is important for the credibility of the decisions made by the TSC and for the integrity of the trial.

Potential competing interests should be disclosed via the trials office. In many cases simple disclosure up front should be sufficient. Otherwise, the (potential) independent TSC member should remove the conflict or stop participating in the TSC. **Table 1** lists potential competing interests.

☐ **No,** I have no potential competing interests to declare

☐ **Yes,** I have potential competing interests to declare (please detail below)

Please provide details of any potential competing interests:

____

____

____

Name: ______________________________

Signed: _____________________________ Date: _______________

Table 1: Potential competing interests for independent members

- Stock ownership in any commercial companies involved
- Stock transaction in any commercial company involved (if previously holding stock)
- Consulting arrangements with the Sponsor/Funder
- Ongoing advisory role to a company providing products to the trial
- Frequent speaking engagements on behalf of the intervention
- Career tied up in a product or technique assessed by trial
- Hands-on participation in the trial
- Involvement in the running of the trial
- Emotional involvement in the trial
- Intellectual conflict e.g., strong prior belief in the trial's experimental arm
- Involvement in regulatory issues relevant to the trial procedures
- Investment (financial or intellectual) or career tied up in competing products

For peer review only

- Involvement in the writing up of the main trial results in the form of authorship

Annex 2: Agreement and competing interests form for non-independent members

RESET FOR REMISSION TRIAL: Agreement to join the Trial Steering Committee as a non-independent member and disclosure of potential competing interests

Please complete the following document and return to the Trials Office.

(Please initial box to agree)

☐ I have read and understood the TSC Charter version 1.0, dated 24th April 2013

☐ I agree to join the Trial Steering Committee for this trial as a non-independent member

☐ I agree to treat all sensitive trial data and discussions confidentially

The avoidance of any perception that members of a TSC may be biased in some undisclosed fashion is important for the credibility of the decisions made by the TSC and for the integrity of the trial.

Possible competing interests should be disclosed via the trials office. In many cases simple disclosure up front should be sufficient. Otherwise, the (potential) independent TSC member should remove the conflict or stop participating in the TSC. **Table 1** lists potential competing interests.

☐ **No,** I have no competing interests to declare other than involvement in the trial

☐ **Yes,** I have competing interests to declare (please detail below)

Please provide details of any competing interests:

____

____

____

Name: ____________________________

Signed: ___________________________ Date: _______________

Table 1: Potential competing interests for non-independent members

- Stock ownership in any commercial companies involved
- Stock transaction in any commercial company involved (if previously holding stock)
- Consulting arrangements with the Sponsor/Funder
- Ongoing advisory role to a company providing drugs to the trial
- Frequent speaking engagements on behalf of the intervention
- Intellectual conflict e.g., strong prior belief in the trial's experimental arm
- Involvement in regulatory issues relevant to the trial procedures

For peer review only

TSC Charter for R4R

Author: Kaberi Dasgupta and Thomas Yates

Date: 13/01/2020

Version: 1.0

- Investment (financial or intellectual) in competing products

Annex 3: Agreement and confidentiality agreement for observers

RESET FOR REMISSION TRIAL: Agreement to attend the Trial Steering Committee and treat all information confidentially

Please complete the following document and return to the Trials Office.

(Please initial box to agree)

☐	I have received a copy of the TSC Charter version 1.0 dated 24th April 2013
☐	I agree to attend the Trial Steering Committee meeting on ___/____/______
☐	I agree to treat as confidential any sensitive information gained during this meeting unless explicitly permitted

Name: ______________________________

Signed: _____________________________ Date: _______________

For peer review only

Annex 4: Document History

Version	Date	Who	Comments
1.0	24 April 2013	DM	First draft

For peer review only

SPIRIT
STANDARD PROTOCOL ITEMS: RECOMMENDATIONS FOR INTERVENTIONAL TRIALS

SPIRIT 2013 Checklist: Recommended items to address in a clinical trial protocol and related documents*

Section/item	Item No	Description	Page Number that item is reported
**Administrative information**			
Title	1	Descriptive title identifying the study design, population, interventions, and, if applicable, trial acronym	**1**
Trial registration	2a	Trial identifier and registry name. If not yet registered, name of intended registry	**4**
	2b	All items from the World Health Organization Trial Registration Data Set	**4**
Protocol version	3	Date and version identifier	**4**
Funding	4	Sources and types of financial, material, and other support	**29**
Roles and responsibilitie s	5a	Names, affiliations, and roles of protocol contributors	**1 and 29**
	5b	Name and contact information for the trial sponsor	**25**
	5c	Role of study sponsor and funders, if any, in study design; collection, management, analysis, and interpretation of data; writing of the report; and the decision to submit the report for publication, including whether they will have ultimate authority over any of these activities	**29**
	5d	Composition, roles, and responsibilities of the coordinating centre, steering committee, endpoint adjudication committee, data management team, and other individuals or groups overseeing the trial, if applicable (see Item 21a for data monitoring committee)	**25-26**<br>**DMC and TSC appendix**

For peer review only

**Introduction**			
Background and rationale	6a	Description of research question and justification for undertaking the trial, including summary of relevant studies (published and unpublished) examining benefits and harms for each intervention	**5-6**
	6b	Explanation for choice of comparators	**5-6**
Objectives	7	Specific objectives or hypotheses	**6-7**
Trial design	8	Description of trial design including type of trial (eg, parallel group, crossover, factorial, single group), allocation ratio, and framework (eg, superiority, equivalence, noninferiority, exploratory)	**9**
**Methods: Participants, interventions, and outcomes**			
Study setting	9	Description of study settings (eg, community clinic, academic hospital) and list of countries where data will be collected. Reference to where list of study sites can be obtained	**9-10**
Eligibility criteria	10	Inclusion and exclusion criteria for participants. If applicable, eligibility criteria for study centres and individuals who will perform the interventions (eg, surgeons, psychotherapists)	**10-12 Table 2**
Interventions	11a	Interventions for each group with sufficient detail to allow replication, including how and when they will be administered	**16-20**
	11b	Criteria for discontinuing or modifying allocated interventions for a given trial participant (eg, drug dose change in response to harms, participant request, or improving/worsening disease)	**20-21**
	11c	Strategies to improve adherence to intervention protocols, and any procedures for monitoring adherence (eg, drug tablet return, laboratory tests)	**24**
	11d	Relevant concomitant care and interventions that are permitted or prohibited during the trial	**16-20**
Outcomes	12	Primary, secondary, and other outcomes, including the specific measurement variable (eg, systolic blood pressure), analysis metric (eg, change from baseline, final value, time to event), method of aggregation (eg, median, proportion), and time point for each outcome. Explanation of the clinical relevance of chosen efficacy and harm outcomes is strongly recommended	**6-9** Table 1 **13-16** (table summary)

For peer review only

Participant timeline	13	Time schedule of enrolment, interventions (including any run-ins and washouts), assessments, and visits for participants. A schematic diagram is highly recommended (see Figure)	**13** (Figure 1)
Sample size	14	Estimated number of participants needed to achieve study objectives and how it was determined, including clinical and statistical assumptions supporting any sample size calculations	**21 - 22**
Recruitment	15	Strategies for achieving adequate participant enrolment to reach target sample size	**13 and 24**
**Methods: Assignment of interventions (for controlled trials)**			
Allocation:			
Sequence generation	16a	Method of generating the allocation sequence (eg, computer-generated random numbers), and list of any factors for stratification. To reduce predictability of a random sequence, details of any planned restriction (eg, blocking) should be provided in a separate document that is unavailable to those who enrol participants or assign interventions	**16**
Allocation concealment mechanism	16b	Mechanism of implementing the allocation sequence (eg, central telephone; sequentially numbered, opaque, sealed envelopes), describing any steps to conceal the sequence until interventions are assigned	**16**
Implementation	16c	Who will generate the allocation sequence, who will enrol participants, and who will assign participants to interventions	**16**
Blinding (masking)	17a	Who will be blinded after assignment to interventions (eg, trial participants, care providers, outcome assessors, data analysts), and how	**16**
	17b	If blinded, circumstances under which unblinding is permissible, and procedure for revealing a participant's allocated intervention during the trial	**N/A**

For peer review only

Methods: Data collection, management, and analysis			
Data collection methods	18a	Plans for assessment and collection of outcome, baseline, and other trial data, including any related processes to promote data quality (eg, duplicate measurements, training of assessors) and a description of study instruments (eg, questionnaires, laboratory tests) along with their reliability and validity, if known. Reference to where data collection forms can be found, if not in the protocol	**13-16**
	18b	Plans to promote participant retention and complete follow-up, including list of any outcome data to be collected for participants who discontinue or deviate from intervention protocols	**21,22 and 24**
Data management	19	Plans for data entry, coding, security, and storage, including any related processes to promote data quality (eg, double data entry; range checks for data values). Reference to where details of data management procedures can be found, if not in the protocol	**23**
Statistical methods	20a	Statistical methods for analysing primary and secondary outcomes. Reference to where other details of the statistical analysis plan can be found, if not in the protocol	**22 - 23**
	20b	Methods for any additional analyses (eg, subgroup and adjusted analyses)	**22 – 23**
	20c	Definition of analysis population relating to protocol non-adherence (eg, as randomised analysis), and any statistical methods to handle missing data (eg, multiple imputation)	**22 - 23**
**Methods: Monitoring**			
Data monitoring	21a	Composition of data monitoring committee (DMC); summary of its role and reporting structure; statement of whether it is independent from the sponsor and competing interests; and reference to where further details about its charter can be found, if not in the protocol. Alternatively, an explanation of why a DMC is not needed	**26**<br>**DMC appendix**
	21b	Description of any interim analyses and stopping guidelines, including who will have access to these interim results and make the final decision to terminate the trial	**DMC appendix**

For peer review only

Harms	22	Plans for collecting, assessing, reporting, and managing solicited and spontaneously reported adverse events and other unintended effects of trial interventions or trial conduct	**20-21**
Auditing	23	Frequency and procedures for auditing trial conduct, if any, and whether the process will be independent from investigators and the sponsor	**27-28**<br>**DMC appendix**
**Ethics and dissemination**			
Research ethics approval	24	Plans for seeking research ethics committee/institutional review board (REC/IRB) approval	**3-4**
Protocol amendments	25	Plans for communicating important protocol modifications (eg, changes to eligibility criteria, outcomes, analyses) to relevant parties (eg, investigators, REC/IRBs, trial participants, trial registries, journals, regulators)	**20**<br>**DMC appendix**<br>**TSC appendix**
Consent or assent	26a	Who will obtain informed consent or assent from potential trial participants or authorised surrogates, and how (see Item 32)	**27-28**
	26b	Additional consent provisions for collection and use of participant data and biological specimens in ancillary studies, if applicable	**28**
Confidentiality	27	How personal information about potential and enrolled participants will be collected, shared, and maintained in order to protect confidentiality before, during, and after the trial	**27-28**
Declaration of interests	28	Financial and other competing interests for principal investigators for the overall trial and each study site	**28-29**
Access to data	29	Statement of who will have access to the final trial dataset, and disclosure of contractual agreements that limit such access for investigators	**27-28**<br>**TSC and DMC appendix**
Ancillary and post-trial care	30	Provisions, if any, for ancillary and post-trial care, and for compensation to those who suffer harm from trial participation	**See consent form**<br>**20**
Dissemination policy	31a	Plans for investigators and sponsor to communicate trial results to participants, healthcare professionals, the public, and other relevant groups (eg, via publication, reporting in results databases, or other data sharing arrangements), including any publication restrictions	**26**

For peer review only

	31b	Authorship eligibility guidelines and any intended use of professional writers	**N/A**
	31c	Plans, if any, for granting public access to the full protocol, participant-level dataset, and statistical code	**27**
**Appendices**			
Informed consent materials	32	Model consent form and other related documentation given to participants and authorised surrogates	**See appendix**
Biological specimens	33	Plans for collection, laboratory evaluation, and storage of biological specimens for genetic or molecular analysis in the current trial and for future use in ancillary studies, if applicable	**N/A**

*It is strongly recommended that this checklist be read in conjunction with the SPIRIT 2013 Explanation & Elaboration for important clarification on the items. Amendments to the protocol should be tracked and dated. The SPIRIT checklist is copyrighted by the SPIRIT Group under the Creative Commons "Attribution-NonCommercial-NoDerivs 3.0 Unported" license.

For peer review only
